# Piloting a psychosocial intervention for perinatal depression, the Thinking Healthy Programme–Peer delivered (THPP), in a primary care setting in Lilongwe District, Malawi

**DOI:** 10.1371/journal.pgph.0002128

**Published:** 2024-05-01

**Authors:** Mwawi Ng’oma, Najia Atif, Samantha Meltzer-Brody, Ellen Chirwa, Robert C. Stewart

**Affiliations:** 1 Department of Psychiatry and Mental Health, Kamuzu University of Health Sciences, Blantyre, Malawi; 2 Programs Department, St John of God Hospitaller Services, Lilongwe, Malawi; 3 Human Development Research Foundation, Islamabad, Pakistan; 4 Department of Psychiatry, University of North Carolina–Chapel Hill, Chapel Hill, North Carolina, United States of America; 5 Department of Reproductive Health, Kamuzu University of Health Sciences, Blantyre, Malawi; 6 Division of Psychiatry, University of Edinburgh, Edinburgh, United Kingdom; Tufts University School of Medicine, UNITED STATES

## Abstract

Despite the evidence for the effectiveness of psychosocial interventions for perinatal depression, their uptake is low in Low- and Middle-Income Countries. Reasons for this include the lack of contextually adapted interventions and mental health specialists to deliver them. This study aimed to test the acceptability and feasibility of a psychosocial intervention for perinatal depression, the *Thinking Healthy Programme–Peer Delivered*, adapted for use in rural Malawi. A multi-method evaluation of feasibility and acceptability of the intervention was conducted using a one-group pretest-posttest quasi-experimental design and an exploratory qualitative study. Pre-post intervention change in depression scores (paired t-test) and recruitment, retention and session adherence rates were calculated. Qualitative data were collected through 29 in-depth interviews (22 mothers and 7 peer volunteers) and 1 Focus Group Discussion (18 mothers). Thematic analysis approach was used to analyse qualitative data. Seven (7) out of 8 peer volunteers were successfully trained to deliver the intervention. A total of 31 pregnant women with Edinburgh Postnatal Depression Scale (EPDS) score ≥12 were offered intervention, of whom 24 were enrolled (recruitment rate 77.4%). Out of these 24 women, 22 completed the intervention (retention rate 91.6%). Mean difference between pre- and post-test EPDS scores one week after 8^th^ session was 7.59 (95% CI 4.98 to 10.19), p<0.001. Qualitative evaluation showed that the intervention was acceptable despite some challenges including stigma and issues around incentivization of peer volunteers. The Thinking Healthy Programme–Peer Delivered, adapted for use in Malawi, was feasible to deliver and acceptable to its target population. The intervention may be useful in management of perinatal depression in primary care settings in Malawi. However, definitive trials are needed to evaluate its effectiveness.

## Introduction

Globally, perinatal depression is a serious mental health condition with adverse impact on maternal and child health outcomes [1–6]. It is highly prevalent in Low- and Middle-Income Countries (LMICs). Pooled prevalence rates of up to 34% in the antenatal period and 25.8% in the postnatal period were reported in systematic reviews of studies conducted in LMICs [3, 7]. A recent systematic review of studies conducted in Malawi reported pooled prevalence of antenatal and postnatal depression of 17.1% and 19.8% respectively with an overall prevalance of perinatal depression of 18.9% [8]. A mother’s mental and psychological wellbeing is highly significant for her child’s wellbeing especially during the first 3 years when the child is most highly dependent on her care [9, 10]. In LMICs such as Malawi, most mothers have no access to appropriate mental health services, primarily due to lack of mental health trained staff and the poor availability of psychotropic medications, including antidepressants, at Primary Health Care (PHC) facilities [11–13].

There is ample evidence that in resource-constrained settings low-intensity psychological or psychosocial interventions can be effectively delivered by non-specialist/lay health workers with proper training, supervision and support [14, 15]. However, such interventions need to be culturally and contextually adapted to make them relevant to the needs of the target population [16] and to ensure their uptake [17]. Systematically adapted interventions have shown better outcomes compared with those implemented without adaptation [16, 18, 19]. In a pilot Randomized Controlled Trial (RCT) in Malawi, an adapted counseling intervention (the Friendship Bench) delivered to women living with HIV who screened positive for perinatal depression, improved maternal health outcomes. Women in the intervention arm showed higher depressive symptom reduction, better retention in HIV care, and there was a trend toward greater viral suppression, compared to the control group [20].

In a previous study, we adapted a Cognitive Behavioural Therapy (CBT) based psychosocial intervention for perinatal depression called the Thinking Healthy Programme–Peer delivered (THPP) for use in rural Malawi [21]. The adaptation process involved engaging participants similar to the intended population to review, critique and offer suggestions about role-played sessions of the Thinking Healthy Programme in theatre testing. Information collected through this process and topic experts’ reviews were used to make changes to the intervention manuals. The core elements of the THPP including developing empathetic relationships, psychoeducation, facilitating family support, behavioral activation and problem solving were retained in the adapted version. Major adaptations were made to language and the illustrations and stories that the THPP uses to help women reflect on their thinking, behaviour and feelings. We redesigned all illustrations, altered stories to fit the Malawi context, and simplified language where needed.

The THPP is an adaptation of the Thinking Healthy Programme (THP), simplified for use by peer volunteers [22]. The THP is recommended by the World Health Organisation (WHO) as first line psychosocial intervention for perinatal depression in primary and secondary health care settings [23]. Randomized controlled trials conducted in Pakistan [24] and India [25] showed THPP’s effectiveness in improving maternal health outcomes compared to enhanced usual care at 3 months postpartum. To our knowledge no such family-focussed intervention for perinatal depression has been tested in Malawi, in spite of evidence that positive social support is a protective factor for perinatal depression [26–28]. Previous and current trials of adapted interventions for perinatal depression in Malawi focused on perinatal women alone [20, 29].

In this multi-method study, we piloted an adapted THPP to evaluate its feasibility and acceptability to perinatal women with depression in communities surrounding Kabudula Community Hospital in rural Lilongwe District, Central Malawi.

## Methods

### Study setting and design

The study was conducted in communities surrounding Kabudula Community Hospital in rural Lilongwe district, Central Region of Malawi. Malawi is a landlocked country with an agriculture-based economy [30]. The majority of Malawians live below the poverty line [30]. Malawi has a youthful population with 49% within age 18 to 64 years out of the total population 17,563,749 as of the 2018 Malawi population and housing census [31]. The majority of the population (84%) reside in rural areas. The 2015–2016 Malawi Demographic and Health Survey showed lower levels of completed education for women compared to men; the fertility rate was 4.4 with adolescent pregnancies estimated at 29% [32]. Many women reported experiencing physical and sexual violence, with husbands or partners being the main perpetrators of physical violence [32]. HIV prevalence was 10.8% in women and 6.4% in men [32]. Although infant and maternal mortality rates were on a decrease at 42 deaths per every 1,000 live births and 439 deaths per 100,000 live births respectively, these still remained high compared to other African countries [32].

Kabudula Community Hospital is located about 56 km away from Lilongwe City. The main economic activity in the surrounding area is subsistence farming. The hospital caters for 124 villages [33]. It is one of the 2 community hospitals operating at primary level of care in Lilongwe District serving an estimated population of 44,334, with 10,197 women of childbearing age [34]. The facility provides maternal, child health and general adult health services. No maternal mental health interventions are provided at the hospital nor in surrounding communities.

A multi-method design was used to evaluate the feasibility and acceptability of the adapted THPP intervention. It had three components: (1) one-group pretest-posttest quasi-experimental design, powered to detect change in depression scores pre- and post-intervention; (2) quantitative measures of recruitment, retention and adherence of mothers in the intervention; and, (3), an exploratory descriptive qualitative study to explore participant perceptions on the acceptability and feasibility of the intervention [35].

### Intervention training and delivery procedures

#### Training of Trainers

The Principal Investigator (PI), 4 research assistants, and 5 health workers from Kabudula Community Hospital (3 nurse midwives working in antenatal and postnatal clinics and 2 environmental health officers) went through a Training of Trainers (TOT) programme consisting of 3 days of classroom training followed by fieldwork spread over 3 months. During fieldwork, each participant delivered the intervention under supervision to two mothers with depression from another part of Lilongwe District (Area 25 PHC clinic). This training and supervision were facilitated by THPP master trainers from Pakistan via video-conferencing between May to July 2020. All research tools including the THPP manuals, and interview guides used to collect qualitative data on perceived feasibility and acceptability of the THPP from perinatal women and peer volunteers through in-depth interviews and Focus Group Discussion (FGD), were pretested during the field work and necessary changes made.

#### Recruitment and training of peer volunteers

The peer volunteers were recruited by the PI in liaison with the village health committees and Kabudula Hospital’s environmental health staff. Community sensitization meetings were conducted to share information about the study with potential peer volunteers and those interested met with the respective village health committees. Criteria developed from findings of the formative qualitative study on perception of perinatal depression and health service needs was used in choosing peer volunteers [36]. A mature parous woman regarded as “respectable” in the community was identified in each of 8 randomly selected villages surrounding Kabudula Community Hospital. They had each attained a minimum of 8 years of education, i.e. Standard 8 (highest primary school class) or secondary level of education. All volunteers were able to read and write.

The training of the peer volunteers in THPP was spread over two weeks; one-week in the classroom followed by one-week of intensive supervised fieldwork (see training schedule S2 File for detailed topics). The peer volunteers’ competencies were assessed during each session using an adapted standardized tool, the Enhancing Assessment of Common Therapeutic factors [ENACT] [37, 38]. The ENACT uses a 3-point Likert scale (where a score of 1 = Needs improvement, 2 = Done partially and 3 = Done well) on 18 dimensions with a total score of 54; a higher score indicates greater mastery **(**the tool is available as S3 File). One peer volunteer was dropped after showing inadequate skills on repeated assessments and 7 peer volunteers were recruited into the study. Characteristics of the peer volunteers are shown in Table 1.

**Table 1:** Characteristics of the peer volunteers.

Peer volunteers (n = 7)		
Age		Median 36 years, (Range 32–51 years)
Marital status	Married (monogamous marriage)	5
	Married (polygamous marriage)	2
Number of Children		Median 3, (Range 3–6)
Level of education	Junior Certificate^1^	5
	Primary School Leaving Certificate^2^	2
Voluntary work experience	Health Programme	4
	Agriculture programme	1
	None	2

^1^Junior Certificate–successful completion of 8 years of primary education and 2 years of secondary education

2 Primary School Leaving Certificate–successful completion of 8 years of primary education

#### Recruitment of participants for the intervention

Recruitment of pregnant women was conducted over a period of 2 months, from September to October, 2020. Pregnant women were provided with information about the study at the Kabadula Community Hospital antenatal clinic waiting room as part of their routine health education sessions before clinic activities. All those interested were referred to the research team and assessed against inclusion and exclusion criteria. Inclusion criteria were: a pregnant woman aged 18 years and above, between 24–28 weeks gestation, and living in one of the 8 randomly selected villages (strata).We excluded women with psychosis and other disorders requiring urgent medical or psychiatric care. The study information sheet including the benefits and risks of participation was read word-for-word to the prospective participants. Those consenting to take part signed a written consent form (signature or thumb print verified by a witness). Following the consenting process, the women were screened for depression using a locally adapted and validated Chichewa version of EPDS [39] with a cutoff point of 12 until the required sample of 24 women (3 from each of the 8 villages) scoring 12 or more on the EPDS was reached.

The required minimum sample size to power the one-group pretest-posttest quasi-experimental study was calculated based on the formula for calculating sample size for single arm trials [40]: n = Z^2^p(1-p)/d^2^ (where Z = 1.96 at 95% confidence; p = proportion of women with postnatal depression; 1-p is the proportion of women without postnatal depression; d = effect size. For this study, therefore p = 0.14; 1-p = 0.86; d = 0.15. The effect size was estimated based on a previous study which reported that the THPP intervention was significantly effective in reducing depressive symptoms [41]). The calculated minimum sample size was 21 participants. Assuming a refusal rate of 15%, an additional 3 participants were added to the sample size for a total of 24 participants.

#### Intervention delivery

Pregnant women with depression recruited to the study were offered eight to ten individual home-based sessions and two group sessions from October, 2020 to May 2021. Four individual sessions were delivered during the last trimester of the pregnancy, and four to six sessions were delivered following childbirth between weeks 2 to 12 postpartum. The sessions were aimed at improving the mother’s personal health, her relationships with people around her, and her relationship with her child [22, 42]. The peer volunteers used the adapted THPP manuals with contextually relevant illustrations to help mothers reflect on their thinking processes, promote behavior activation through homework, and encourage family support. Problem-solving techniques were used during the sessions and while reviewing homework.

Fortnightly supportive supervision sessions of the peer volunteers were conducted by the PI, research assistants and environmental health staff. Individual intervention sessions were reviewed and participants requiring specialist care were referred to the hospital psychiatric nurse for management and further referral to a specialist hospital within the district.

### Quantitative evaluation

An adapted and validated Chichewa version of the EPDS [36, 39, 43–45], was used to screen women for symptoms of depression. Women scoring 12 or above on EPDS were eligible to participate in the study. For those who participated, EPDS was repeated one week after the 8^th^ session (the minimum number of sessions considered effective based on THPP trails conducted in Pakistan and India showing stronger evidence of the benefit of interventions at 3 months compared to 6 months) to compare pre- and post-intervention scores.

#### Pre-post intervention depression scores

Paired t-test was used to compare the changes in pretreatment and posttreatment depression scores.

#### Recruitment, adherence and retention

Recruitment, adherence and retention of participants in the intervention were determined by calculating the number of participants eligible for the intervention versus number enrolled (recruitment), number of sessions attended by each participant (adherence), and number of participants that completed the intervention as designed (retention). Quantitative data were analyzed using the Statistical Package for Social Sciences (SPSS) V.20.

### Qualitative evaluation

For the qualitative study exploring acceptability and perceived feasibility, in-depth interviews were conducted with 22 mothers who completed the intervention and seven peer volunteers. In addition, one Focus Group Discussion (FGD) was conducted with 18 of the 22 mothers to generate collective understanding of facilitators and barriers of the intervention. Interview guides were developed informed by the study objectives and literature review. Perinatal women’s satisfaction with the intervention, their experiences, possible benefits, and peer volunteers’ experiences in delivering the intervention were explored. To evaluate perceived feasibility of implementing THPP, a guide with elements adapted from a Structured Assessment of Feasibility (SAFE) tool [46] was used. The SAFE was designed by Kings College, London for use in the UK National Health Service (NHS) to assess the feasibility of implementing complex intervention within mental health services.

A pilot-test of the interview guides was conducted during research staff and peer volunteers’ field work at Area 25 PHC clinic on 10 participants, who were not part of the current study. Necessary adjustments were made to the interview guides through an iterative process until researcher were satisfied with the guides’ performance (The interview guides are available as S4 and S5 Files).

The Principal Investigator (PI) and research assistants interviewed the mothers and facilitated the FGD while an independent interviewer, who did not participate in the pilot study, interviewed the peer volunteers to avoid any biases. All interviews were conducted in designated offices at Kubudula Community Hospital; they lasted for 30 minutes to an hour, while FGD took 1 hour 15 minutes. All interviews were audio recorded and observational notes were taken.

Thematic analysis approach was used to analyze qualitative data [47]. Audiotaped interviews were transcribed verbatim and translated into English. The PI reviewed all audio recordings against the transcripts and reviewed observational notes to become familiar with the data. Data were then imported into NVivo Release 1 for coding and analysis. Using codes deductively developed from the interview guides, the PI and one researcher double coded 2 transcripts in NVivo and through an iterative process generated a final code book, reviewing the codes, merging some, removing others and adding emerging ones. The PI coded the rest of the transcripts and organized a summary of the data in Microsoft Excel sheet with reference to a particular transcript, code, emerging themes and participants’ quotes.

### Ethical consideration

Ethical Clearance for this study was obtained from College of Medicine Research and Ethics Committee (COMREC), Approval certificate number P.09/19/2804. Permission to conduct the study at Kabudula Community Hospital was obtained from Lilongwe District Health Office (DHO). Written consent (signature or thumb print verified by a witness) was obtained from the participants before commencement of the study. The researchers read out the information sheets word-for-word to all potential participants confidentially. The information sheets contained information on study procedures (recruitment, intervention and assessments), expectations, benefits of participating in the study, possible risks and risk mitigation. An opportunity was given to participants to ask questions and those who showed interest gave consent. Psychosocial support and counselling were offered to all mothers in this study including referral if needed.

## Results

### Feasibility of recruitment

Out of 270 who were approached, 166 pregnant women were screened for depression using the locally validated version of the EPDS. Thirty-one pregnant women scored above cut-off on the EPDS and were eligible for recruitment into the intervention. Of these 31 eligible women, 24 were recruited into the intervention giving a recruitment rate of 77.4%. The median age of the women was 24 years, ranging between 18 and 36; 18 were married and 6 divorced/separated with the median number of children being 2 ranging between 0 and 8.

### Retention and adherence

**Fig 1:**
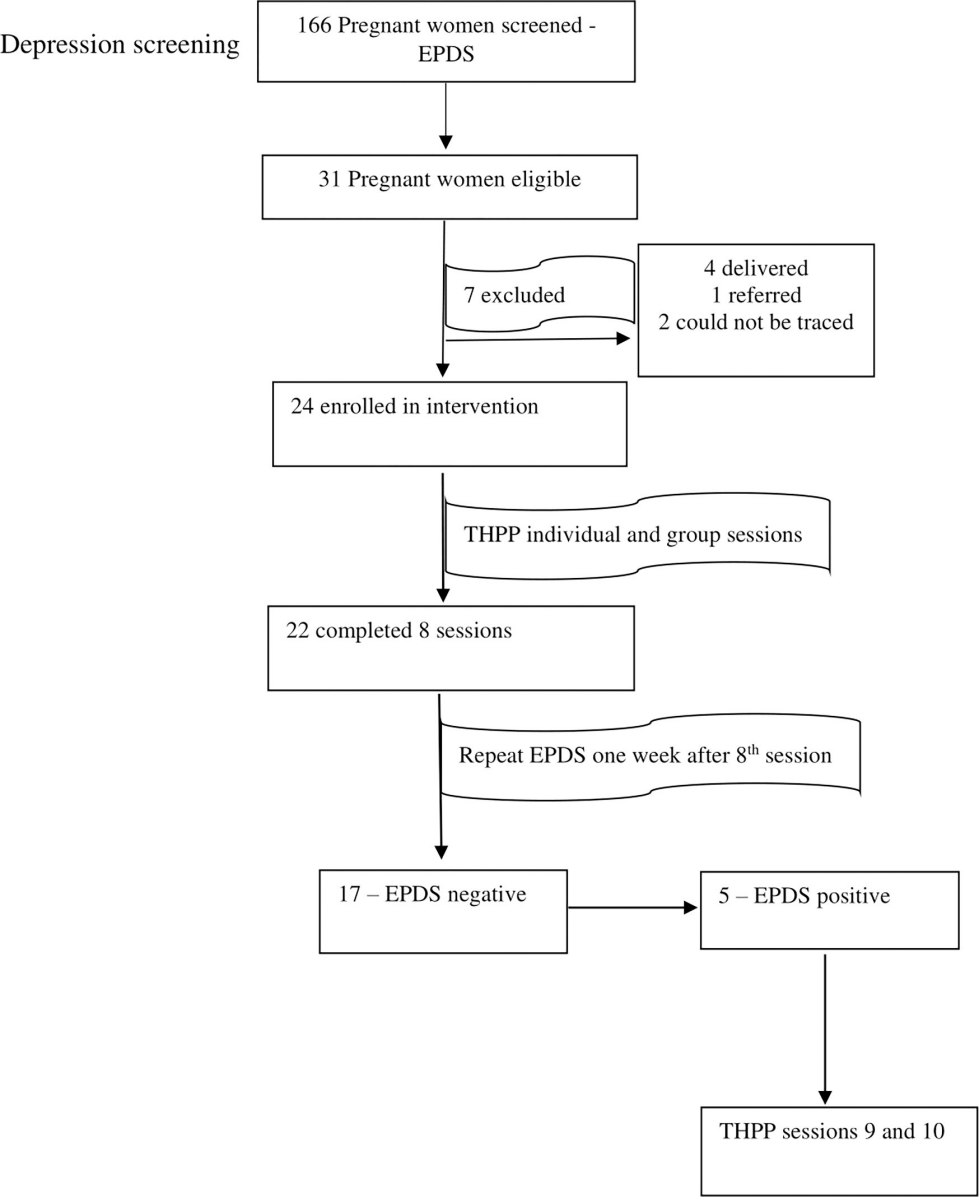
Recruitment and retention of participants in the pilot study.

Of the 24 women recruited, 22 completed the intervention, the required 8 sessions (a retention rate of 91.6%). Of the 22 women, 5 (who had persistently high EPDS scores after 8 sessions) attended 10 individual sessions. There was 100% adherence to the sessions. See Fig 1.

### Change in depressive symptoms pre-post intervention

The EPDS was administered at baseline and one week after the 8^th^ session for 22 mothers. A paired *t*-test analysis showed a significant reduction in the mean EPDS scores from baseline to endline assessment: baseline mean score 13.73 SD = 3.62, post THPP mean score 6.00 SD = 5.46, mean difference = 7.59 (95% CI 4.98 to 10.19), p<0.001.

### Perceived acceptability and feasibility of THPP

A total of eight main themes and 20 subthemes were generated from analysis of the qualitative data. These are summarized in Table 2. The themes/subthemes are presented supported by selected participant’s excerpts.

**Table 2:** Themes and subthemes.

Area/Code	Theme	Subthemes
Training	Sufficiency of training	Adequate and helpful Short duration Undesirable training venue
Feasibility of delivery	Difficulties in the process	Challenges to initiate Challenges moving from one step of THPP to the other Mother’s complicated issues
	Positive experiences	Illustrations facilitated delivery Participant’s satisfaction Unexpected personal benefits
	Facilitators	Home delivery Good rapport Family involvement/support
	Barriers	Stigma Insufficient incentives
Perceived acceptability	Satisfaction with THPP	Personal improvement Improved family relation and support
	Satisfaction with Peer volunteers	Peer volunteers skills and attributes Peer volunteers support
	Barriers to perceived impact	Poverty Lack of support

#### Area 1: Training

*Sufficiency of training*. The majority of the peer volunteers found the THPP training adequate and helpful however, some peer volunteers especially those who struggled to revise content at home, felt that a longer training was needed.

**PV#6** *“…Few training days…we were operating from our homes during the cultivation time*, *after the training we would just drop the books and go back to the fields… limited time to go through the books*…*”*

#### Area 2: Perceived feasibility of THPP delivery

*Difficult experiences during THPP delivery*. Some difficulties were reported during delivery of the THPP sessions. Some peer volunteers initially found delivering the introductory session challenging. Over time, they gradually gained more experience, which increased their confidence:

**PV#5** *“The introduction session was difficult*…*the intervention was new…meeting the family for the first time*. *I did not know where and how to start but the rest were easy”*.

A few peer volunteers found challenging to deliver intervention to mothers who were experiencing complex issues and had chronic symptoms while one mother stated that the intervention was complex initially:

**PV#4** *“When participant had lots of issues*, *it became difficult to move from one step to another*. *It took me some time to understand and successfully deliver it to high need participants…”*

**IDI Mother#11** *“the only problem I had was interpreting some terms used*,*… I just tried to figure out the meaning on my own…”*.

However, perceived support and good collaboration with the research and hospital staff was deemed helpful in managing the challenges faced in implementation:

**PV#6** *“… the good part was we were not alone in the programme we met research and hospital staff often*, *they were helping us how to go about the sessions and how to deal with issues”*.

**PV#2** *“…We worked very well with health workers…*.*if faced with challenges*, *we were asking them”*.

*Positive experiences during THPP delivery*. The THPP sessions are structured and use illustrations. Most peer volunteers reported that while structure simplified the sessions’ delivery, the contextually relevant illustrations helped to explain things better and make it more relatable to the mothers:

**PV#6** “*…it was easy to explain to women through the pictures…even those who did not go to school understood it*, *even the old mother in-laws were able to understand the lessons through the pictures”*.

The perceived satisfaction from mothers and their families were reported to be source of encouragement to the peer volunteers.

*Unexpected personal benefits*. All peer volunteers reported to have benefited from the intervention. Talking about the personal gains, some mentioned improved problem-solving skills, interpersonal relationships and social support, while others gained respect in their communities and feelings of contentment to see the impact:

**PV#6** *“It was very prestigious for us to be known as counsellors in our communities*. *We also felt proud to see healthy babies born during the program with our counselling”*.

*Facilitators to THPP delivery*. Home-based mode was perceived convenient for providers and recipients. No traveling was required for mothers and their families, and the flexibility in meeting times encouraged participants and their families’ engagement:

**IDI Mother #9** “…*Receiving the counseling at home was good…the counselor would come and find us* [family members] *all there*. *If we would have been invited to the clinic*, *they would not be able to come because of transport issues”*.

*Barriers to THPP delivery*. Some participants, peer volunteers and mothers reported HIV/AIDs stigma as a potential barrier to the THPP delivery and access. The stigma resulted from peer volunteers being perceived as Anti-Retroviral Therapy (ART) providers, who provide community care to HIV positive women. This raised concern among a few mothers:

**IDI Mother #2** “…*they were saying the counsellor was dispersing ARVs…my partner was consistently discussing with our landlord*. *She was telling him that he should quiz me…*, *if they continued talking I would have stopped…to avoid tarnishing my image”*

Another barrier to the THPP delivery reported by the peer volunteers was their dissatisfaction with the stipend they were receiving:

**PV#2** *“*…*Much as we were volunteers*, *we were supposed to be paid a certain amount of money*, *which would at least have contributed towards the cost of farming”*.

Likewise, some mothers felt that monitory support would have helped them to implement what was suggested by their peer volunteers.

**IDI Mother #8** *“I feel that apart from the counseling*, *we should be given a certain support so we can implement what we are learning…like money (to improve diet and for social activities)”*.

Furthermore, lack of partners’ involvement and support were identified as barriers to accomplish between session tasks. Most mothers suggested providing material resources/handouts for the partners to improve their engagement:

**PV#2** *“there were some partners who seemed to be very busy and never attended the sessions…mostly those in polygamous marriages*…*”*

#### Area 3: Perceived Acceptability of the THPP

*Satisfaction with the intervention*. Most mothers reported being satisfied with receiving the intervention. They felt that the intervention had equipped them with self-help skills, and had helped them to identify the root cause of their problems. Furthermore, mothers found their families/partners involvement valuable as it helped improving their interpersonal relationship and support:

**IDI Mother #7** *“At first I was experiencing thoughts of harming myself and my unborn baby*, *this intervention has helped me a lot in making me feel better…*, *I would not have breastfed my baby*, *if I would not have received it*,*… …”*

**IDI Mother#3** *“…The counselling helped my family to change the way they relate to me… They started supporting me”*.

The intervention stimulated a lot of interest in the community, in both pregnant and non-pregnant couples:

**IDI Mother #15** *“It is very helpful and it is unfortunate that only pregnant women are targeted… there are also other married people*, *not pregnant that want to benefit from this intervention”*.

*Satisfaction with the peer volunteers*. Most mothers were satisfied with the support from peer volunteers and with their sensitivity in discussing confidential issues:

**IDI#16** *“I was able to express my opinions…each time there was something I was not comfortable sharing in front of others; she would provide me with the opportunity to discuss it in privacy”*.

The peer volunteers were viewed as knowledgeable, skilled and well mannered. The rapport they established with the mothers created a conducive environment for discussions:

**IDI Mother#7** *“… The counselor’s openness helped me to open up and I was assisted*… *I changed because of this intervention…sometimes we suffer in silence because we are not able to confide in someone”*.

## Discussion

This pilot study was conducted to evaluate the feasibility and acceptability of the adapted THPP in a PHC setting in Central Malawi. We found that THPP could be successfully delivered by peer volunteers in spite of some implementation challenges and was well received by mothers and their families. We found that it was feasible to recruit participants to the intervention with good retention and adherence to sessions. Mothers had lower depression scores following the intervention. However, as this was a small-scale uncontrolled pilot study, these changes cannot be attributed solely to the intervention.

The peer volunteers found delivery of the intervention to mothers and their families appropriate and acceptable. Delivering the home-based intervention was convenient, facilitated adherence to the intervention and involvement of family members residing within same village. Additionally, the intervention’s agenda of optimal child development and linking the infant’s wellbeing with the mother’s [22] enhanced mothers’ and their family’s interest in the intervention and reduced stigma evidenced by the high recruitment and retention rate.

The adapted THPP was accepted by most mothers who found the intervention relatable and beneficial in improving their wellbeing. Culturally appropriate interventions have shown usefulness and relevance and are easily accepted [48]. Factors contributing to the intervention’s acceptability in the current study included the use of culturally and contextually relevant illustrations, metaphors and stories, and peer volunteers positive attributes as well the similarity of their demographic and psychosocial characteristics to the intervention recipients. Furthermore, setting culturally acceptable tasks in collaboration with the mothers and their family members was perceived as beneficial.

While most peer volunteers found the training useful, some felt that, due to their lack of experience in mental health work, longer duration of training would have been more appropriate. These findings are in line with recommendations for longer duration of training and more frequent supervisions to ensure peer volunteers’ competency and quality of the intervention delivery [48]. In this current study, the shorter duration of training was compensated for with weekly supervision sessions and support from the research and hospital staff, which enabled most peer volunteers to deliver their sessions with ease. Successful task sharing requires appropriate training, supportive supervision of lay health workers by specialized staff and incentivization [17, 49]. Training and supervision not only prepare the lay workers for their role but also acts as a motivator [50].

Challenges such as mothers’ poverty, lack of food, chronicity of their depression and lack of their partner’s support affected delivery of the intervention, particularly completion of assigned tasks. Similar findings were reported in a systematic review of content and delivery of psychological interventions for perinatal depression by non-specialist health workers in LMICs [48]; and in studies conducted in Uganda, Ethiopia and South Africa [51–53]. Addressing these social economic problems can enable mothers to benefit fully from the intervention.

Stigma related to mental disorders including depression is another known barrier to accessing maternal mental health care [49, 52]. Although in the current study the stigma was related to misconception about peer volunteers being ART delivery agents, it still affected some families. Similar findings were reported in a study conducted in South Africa where clinic-based interventions were preferred to avoid stigma associated with HIV related home based care [54]. These misconceptions might have resulted from lack of awareness about the programme in the community. Successful initiation and delivery of interventions in the community require community buy-in and support through raising their awareness and engagement [49].

Another potential barrier identified in this study was lack of peer volunteers’ and the mothers’ incentivization. Although the peer volunteers received monetary compensation for each session, some expressed their dissatisfaction with the amount and were in favor of formal remuneration. There is evidence that material and monetary incentives are important motivating factors for community health workers and that lower than expected incentives can negatively affect motivation [55, 56]. In other studies, lack of financial compensation, basic resources and recognition from specialist health workers greatly demotivated Health Surveillance Assistants in Malawi [57] and volunteers in Uganda, respectively [58]. Although in our study, the intervention was delivered in a rural community, where volunteerism provides an opportunity for gaining work experience and was traditionally perceived desirable, new experience is showing that expectations are shifting from in-kind benefit and recognition to monetary benefits [59]. Similarly, mothers and their families expressed an expectation of some support in the form of food and money. Similar findings are reported from other studies where participants’ unmet expectations affected both participants and providers’ motivation [53, 60]. Appropriate incentivization is paramount for continued engagement and retention of volunteers’ in the programmes [50].

Despite the dissatisfaction expressed by some peer volunteers over a lack of adequate incentivization, the majority of peer volunteers found their role prestigious and the experience useful for their personal growth and development. The peer volunteers’ helping role was valued and respected in their communities, which boosted their self-esteem. Evidence originated from other LMICs such as Ethiopia, Kenya, Indonesia, Malawi, Mozambique and Uganda also indicated that such personal gains were strong motivators for both salaried and voluntary community health workers [50, 55].

### Strengths and limitations

Triangulation of methods, use of both quantitative and qualitative study designs, multiple data collecting methods and study participants, was a major strength of this study. Collection of data from perinatal women and peer volunteers and use of in-depth interviews and FGD enriched the data collected. The high adherence and retention rate of mothers and peer volunteers in the intervention reflects the perceived relevance of the intervention to the participants (following the careful process of adaptation), and the high quality supportive supervision of the peer volunteers. Similarly, the home-based model of delivery enabled involvement of family members as intended, and enhanced adherence.

Another methodological strength was the use of a validated depression screening measure, the EPDS, previously used in antenatal clinics in Malawi with accurate results [39, 61] to recruit participants and assess depression scores after the intervention.

Limitations in the methodology include the large size of the FGD following completion of the intervention, in which 18 mothers participated. This may have affected the depth of discussions and the willingness of participants to actively participate. Secondly, the views of other family members including partners were not sought despite their involvement in the intervention. Another limitation was the exclusion of pregnant women below 18 years of age. This population is considered special population with specific needs and might require specific intervention. Finally, this study was conducted only in Lilongwe District; therefore, the study results may not be generalizable to other parts of Malawi with differing languages and cultural practices.

## Conclusion and recommendations

The adapted THPP proved feasible and acceptable in a PHC care setting in Lilongwe, Malawi; individual, community and implementation challenges were successfully overcome. Overall, there was good retention and adherence to the intervention and peers were deemed valuable providers of the intervention. Although the current study showed a reduction in depression scores post-intervention, no conclusions can be made about the effectiveness of adapted THPP for the treatment of perinatal depression in the absence of a control group. Therefore, we recommend a randomised controlled trial to test the effectiveness of THPP in Malawi. Similarly future studies should consider incorporating and evaluating longer duration training and continued evaluation of peer volunteers’ competencies, prolonged stakeholder engagement to address stigma and misconceptions and a review of peer volunteers incentives for sustenance of motivation and commitment.

## Supporting information

S1 File THP-TOT Malawi schedule 27–29 May20. (DOCX)

S2 File THPP volunteer training programme. (DOCX)

S3 File Enhancing assessment of common therapeutic ENACT scale. (DOC)

S4 File Interview guide_peer volunteers. (DOCX)

S5 File Interview guide_perinatal women. (DOCX)

S6 File EPDS pre and post test scores after 8 and 10 sessions. (XLS)

S7 File THPP acceptability report_coded transcripts. (DOCX)

S8 File THPP feasibility report_coded transcripts. (DOCX)
