# Supplementary material for: Piloting a psychosocial intervention for perinatal depression, the Thinking Healthy Programme–Peer delivered (THPP), in a primary care setting in Lilongwe District, Malawi
Source: PLOS Glob Public Health. 2024 May 1;4(5):e0002128. doi: 10.1371/journal.pgph.0002128 (PMC11062519; doi:10.1371/journal.pgph.0002128)
Supplement: S1 File — (DOCX) [file pgph.0002128.s001.docx]

**Training of the Trainers**

Thinking Healthy Programme – *Peer delivered*

*Date: 27^th^– 29^th^ May ‘20*

***Zoom link:*** <https://us02web.zoom.us/j/81213024965>

**Trainers**

**Dr Najia Atif**

Consultant Psychologist & Senior Research Fellow

Human Development Research Foundation, Pakistan

Honorary Research Fellow

Lancashire Care NHS Foundation Trust, UK

Email: [najia.atif@hdrfoundation.org](mailto:najia.atif@hdrfoundation.org)

**Huma Nasir**

Research Fellow

Human Development Research Foundation, Pakistan

Email: [huma.nasir@hdrfoundation.org](mailto:huma.nasir@hdrfoundation.org)

| **Training day 1** | |
| --- | --- |
| Malawi Standard Time | Contents of the training day |
| 9:00 am – 9:15 am | Welcome and Introductions |
| 9:15am – 10:00 am | Overview of the Thinking Healthy Programme Peer Delivered (THPP) |
| 10:00 am – 10:45 am | Introduction to the Bernal’s framework for adaptation of the THPP to local context |
| 10:45 am – 11:30 am | Perinatal depression, its risk factors and impact |
| 11:30am – 12:00 noon | Tea break |
| 12:00 – 12:15 pm | Introduction to the Cognitive Behavior Therapy (CBT) strategies and its use in THPP |
| 12:15 pm – 1:00 pm | Essential Skill 1 for the delivery of THPP |
| 1:00 pm – 1:45 pm | Lunch break |
| 1:45 pm – 3:45 pm | Essential Skills 2 & 3 for the delivery of THPP |
| 3:45 pm – 4:00pm | Comments and Feedback |
| **Training day 2** | |
| 9:00 am – 9:15 am | Review of training day 1 |
| 9:15 am – 11:15 am | Essential Skills 4 & 5 for the delivery of THPP |
| 11:15 am – 11:45 am | Tea break |
| 11:45 am – 1:00 pm | Understand the content and delivery mechanisms of the THPP individual sessions |
| 1:00 pm – 1:45 pm | Lunch break |
| 1:45 pm – 3:45 pm | Practising delivering the individual sessions through conducting role plays |
| 3:45 pm – 4:00pm | Comments and Feedback |
| **Training day 3** | |
| 9:00 am – 9:15 am | Review of training day 2 |
| 9:15 am – 11:15 am | Understanding the contents and the delivery mechanisms of the THPP group sessions |
| 11:15 am – 11:45 am | Tea break |
| 11:45 am – 1:00 pm | Practising delivering the group sessions through conducting role plays |
| 1:00 pm – 1:45 pm | Lunch break |
| 1:45 pm – 2:45 pm | Implementation of the THPP – a case study from Pakistan |
| 2:45 pm – 3:30 pm | Other relevant information |
| 3:30 pm – 4:00 pm | Comments and Feedback |
