# Supplementary material for: Piloting a psychosocial intervention for perinatal depression, the Thinking Healthy Programme–Peer delivered (THPP), in a primary care setting in Lilongwe District, Malawi
Source: PLOS Glob Public Health. 2024 May 1;4(5):e0002128. doi: 10.1371/journal.pgph.0002128 (PMC11062519; doi:10.1371/journal.pgph.0002128)
Supplement: S2 File — (DOCX) [file pgph.0002128.s002.docx]

**THPP VOLUNTEER CLASSROOM TRAINING PROGRAMME – KABUDULA COMMUNITY HOSPITAL 14^TH^ TO 18^TH^ SEPTEMBER, 2020**

PULOGIRAMU YOGANIZIRA ZA MOYO WA THANZI YOPHUNZITSANA PA CHINZAKE

| **Training day 1** | |
| --- | --- |
| Time | Contents of the training day |
| 9:00 am – 9:15 am | Welcome and Introductions |
| 9:15am – 10:00 am | Overview of the Thinking Healthy Programme Peer Delivered (THPP)  Kuwunikira za pulogiramu yoganizira za moyo wa thanzi yophunzitsana pa chinzake / |
| 10:15 am – 10:45 am | An overview of Perinatal depression, its risk factors and impact  Matenda a nkhawa kapena okhumudwa |
| **10:45am – 11:00 am** | **Tea break** |
| 11:00 – 1:00 pm | Introduction to the Cognitive Behavior Therapy (CBT) strategies and its use in THPP  Kuwunikira za momwe tigwilitsile ntchito ndondomeko za mgwirizano wa maganizo, mamvekedwe ndi chikhalidwe/mchitidwe mu pulogiramuyi |
| **1:00 pm – 1:45 pm** | **Lunch break** |
| 1:45 pm – 2:45 pm | Essential Skills 1 for the delivery of THPP  Luso lofunika mu pulogiramuyi |
| 2:45 pm – 4:00pm | Essential Skills 2 & 3 for the delivery of THPP  Luso lofunika mu pulogiramuyi |
| 4.00 pm - 4:30 pm | Comments and Feedback  Ndemanga |
| **Training day 2** | |
| 9:00 am – 9:15 am | Review of training day 1  Kuwunikanso phunziro la tsiku loyamba |
| 9:15 am – 11:15 am | Essential Skills 4 & 5 for the delivery of THPP  Luso lofunika mu pulogiramuyi |
| **11:15 am – 11:45 am** | **Tea break** |
| 11:45 am – 1:00 pm | Continue Role playing Essential Skills |
| **1:00 pm – 1:45 pm** | **Lunch break** |
| 1:45 pm – 4:15 pm | Understand the content and delivery mechanisms of the THPP individual sessions  Kumvetsa za zomwe zili mu pulogiramuyi ndi momwe yimapelekedwera kwa mayi payekha |
| 4.15 pm - 4:30 pm | Comments and Feedback  Ndemanga |
| **Training day 3** | |
| 9:00 am – 9:15 am | Review of training day 2  Kuwunikanso phunziro la tsiku lachiwiri |
| 9:15 am – 11:15 am | Practicing delivering the individual sessions through conducting **role plays**  Kuyesezela za momwe tidzapelekere thandizoli |
| **11:15 am – 11:45 am** | **Tea break** |
| 11:45 am – 1:00 pm | Practicing delivering the individual sessions through conducting **role plays**  Kuyesezela za momwe tidzapelekere thandizoli |
| **1:00 pm – 1:45 pm** | **Lunch break** |
| 1:45 pm – 4:15 pm | Practicing delivering the individual sessions through conducting **role plays**  Kuyesezela za momwe tidzapelekere thandizoli |
| 4:15 pm – 4:30 pm | Comments and Feedback  Ndemanga |
| **Training day 4** | |
| 9:00 am – 9:15 am | Review of training day 3  Kuwunikanso phunziro la tsiku lachitatu |
| 9:15 am – 11:15 am | Understanding the contents and the delivery mechanisms of the THPP group sessions  Kumvetsa za zomwe zili mu pulogiramuyi ndi momwe yimapelekedwera kwa a mayi pagulu |
| **11:15 am – 11:45 am** | **Tea break** |
| 11.45 am – 1:00 pm | Understanding the contents and the delivery mechanisms of the THPP group sessions  Kumvetsa za zomwe zili mu pulogiramuyi ndi momwe yimapelekedwera kwa a mayi pagulu |
| **1:00 pm – 1:45 pm Lunch break** | |
| 1:45 pm - 4:00 pm | Practising delivering the group sessions through conducting **role plays**  Kuyesezela za momwe tidzapelekere thandizoli kwa a mayi pagulu |
| 4:15 pm – 4:30 pm | Comments and Feedback  Ndemanga |
| **Training day 5** | |
| 9:00 am – 9:15 am | Review of training day 4  Kuwunikanso phunziro la tsiku lachinayi |
| 9.15 am – 10.00 am | Reviewing delivering individual and group session  Kobweleza momwe tidzapelekere thandizoli |
| **10:00 am -10:15 am** | **Tea break** |
| 10:30 am – 12 Noon | Implementation of the THPP – a case study from Area 25  Zitsanzo zomwe tapeza poyesela kupeleka thandizoli ku Area 25 |
| **12 Noon – 12:45 pm - Lunch** | |
| 12:45 pm - 2:30 pm | Referal pathway and working with clinic staff  Kukambilane mmene tigwilire ntchito pamodzi ndi a chipatala |
| 2:30 – 3:00 pm | Preparation for field work  Kukambilana ndondomeko ya ntchito yak u filudi |
| 3:00 pm - 4:00 pm | Comments and winding up  Kumaliza |
