# Supplementary material for: Piloting a psychosocial intervention for perinatal depression, the Thinking Healthy Programme–Peer delivered (THPP), in a primary care setting in Lilongwe District, Malawi
Source: PLOS Glob Public Health. 2024 May 1;4(5):e0002128. doi: 10.1371/journal.pgph.0002128 (PMC11062519; doi:10.1371/journal.pgph.0002128)
Supplement: S3 File — (DOC) [file pgph.0002128.s003.doc]

**Enhancing Assessment of Common Therapeutic (ENACT) factors scale**

**Item 1:** **Developing a bond without verbal communication and listening empathically: eye contact, facial expressions, and body language**

**1 = Needs improvement** = no eye contact with the mother or staring constantly, expressing anger, mocking at her, interrupting the mother repeatedly, ignoring her, talking on the phone without asking for permission

**2 = Done partially =** showing consistent lack of interest through body language, rare eye contact, not expressing emotions, presence

**3 = Done well** = proper eye contact with the mother during conversation/discussion, smiling according to situation, proper seating arrangement and showing interest by leaning towards mother, and listening attentively using hmm…, yes, or any other local expression

**Item 2: Communication skills: asking open-ended questions, summarizing, and explaining the discussion**

**1 = Needs improvement** = asking close-ended questions, for example, will you do this? Can you do this?

**2 = Done partially =** asking open-ended questions but not going in detail and not asking for mother’s opinion after summarizing the discussion

**3 = Done well** = asking open-ended questions, summarizing the discussion and asking the details, for example, what happened? Tell me more

**Item 3: Developing a relationship (when the other person feels comfortable talking to you) and introducing yourself**

**1 = Needs improvement** = Peer Volunteer (PV) does not introduce herself or make the mother feel comfortable or Peer Volunteer talks mostly about her personal experiences during the session

**2 = Done partially** = Peer Volunteer introduces herself but does not make the conversation comfortable for the mother by chit chat or the Peer Volunteer talks about herself to the mother but it has no link with mother’s situation

**3 = Done well** = Peer Volunteer introduces herself, tries to make the conversation easy for the mother, and shares her personal experiences relating to the mother’s situation/condition

**Item 4: In-depth understanding of the matter, explaining, and telling her that such emotions can be felt often**

**1 = Needs improvement** = Peer Volunteer does not talk about mothers feelings or makes her own judgment or criticizes mother’s feelings (e.g. you should not think that, you should stop thinking and feeling about it)

**2 = Done partially** = Peer Volunteer asks about mother’s feelings and emotions but does not empathize with her, agree with her feelings, or ask for details

**3 = Done well** = Peer Volunteer explains the mother’s feelings by relating them to the current situations and if appropriate, tells her that such emotions can be felt during these situations

**Item 5: Dealing with empathy, warmth, and sincerity (with being pretentious)**

**1 = Needs improvement** = Peer Volunteer criticizes mother’s concerns and complaints or behaves angrily or rejects her views

**2 = Done partially** = Generally, PV’s attitude is warm and friendly but she does not have the ability to empathize with her/relate with mother’s perspective

**3 = Done well** = Peer Volunteer expresses that she understands that the mother feels exactly how mostly people feel during this situation

**Item 6: Viewing daily activities and effects on life**

**1 = Needs improvement** = Peer Volunteer does not ask the mother about her thoughts, feelings, and psychological issues and their effects on her life

**2 = Done partially** = Peer Volunteer asks about daily activities and tasks but does not link it to mental or psychological problems

**3 = Done well** = Peer Volunteer talks about the connection between psychological problems and daily activities

**Item 7: Knowing about what explanations (simple/common and explanatory model) do the mother and her social support (family members and friends) give about mother’s problems**

**1 = Needs improvement** = Peer Volunteer does not ask the mother about reasons of her problems, or she makes her own judgment, criticizes mother when she gives any explanation (e.g., witchcraft is not the cause of your problems, this is an ignorant way of thinking)

**2 = Done partially** = Peer Volunteer asks the mother about reasons for her problems but does not probe further if her family members also think the same way? (e.g. when in introductory session two sides of a picture are shown to know the perception of family members)

**3 = Done well** = Peer Volunteer asks the mother about the reason and asks if her family/people in her social circle describe it the same way or differently

**Item 8: Method of dealing with problems and using the pre-existing methods of problem solving**

**1 = Needs improvement** = Peer Volunteer does not ask the mother about how she deals with her problems or makes her own opinion about it (for instance, why do you think it is beneficial or not)

**2 = Done partially** = Peer Volunteer talks about dealing with problems and already existing solution but does not tell in a positive manner

**3 = Done well** = Peer Volunteer asks the mother about methods to deal with difficulties and then explains in a positive way

**Item 9: Reviewing the mother’s ongoing life incidents and circumstances and drawing their link with the mother’s psychological and social satisfaction**

**1 = Needs improvement** = Peer Volunteer does not talk about incidents which create problems

**2 = Done partially** = Peer Volunteer talks about life’s incidents and circumstances/issues but does not connect the effects of unhealthy thoughts and moods with mother and child’s satisfaction (for instance, when PV shows pictures in the last step during each session)

**3 = Done well** = Peer Volunteer talks about life incidents and circumstances and connects the effects of unhealthy thoughts and moods with mother and child’s satisfaction (e.g. when she shows pictures in the last step of each session)

**Item10: Reviewing the problems of mother’s own health**

**1 = Needs improvement** = Peer Volunteer does not talk about the thoughts/concerns of the mother about the mother’s personal health

**2 = Done partially** = Peer Volunteer talks about the thoughts/concerns of the mother about her personal health but does not draw the link between the unhealthy thoughts, mood, behaviors, and their effects (with the help of picture and examples)

**3 = Done well** = Peer Volunteer talks about the mother’s thought/concerns regarding her personal health and wherever it is needed she draws a detailed link between the unhealthy thoughts, mood, behaviors, and their effects (with the help of pictures and examples)

**Item 11: Appropriate involvement of family members and other care takers**

**1 = Needs improvement** = when any family member is present: During the session, PV ignores the family or only talks to the family members and ignores the mother. When no family member is present: PV does not talk about the family at all

**2 = Done partially** = when family members are present: PV talks to both, the mother and the child, but does not help the mother and the family during the session to communicate with each other. When the family member is not present: PV talks about the family involvement but does not take the mother’s view if she wants to involve the family or not

**3 = Done well** = when any family member is present: PV encourages and helps in the communication between the mother and the family member. When the family member is not present: PV asks mother about family involvement and guides her

**Item 12: Setting the goals mutually and talking about the mother’s expectations**

**1 = Needs improvement** = Peer Volunteer does not ask the mother about her goals and expectations regarding the treatment, or PV just tells the mother what to do without asking about her expectations

**2 = Done partially** = Peer Volunteer talks to mother about the goal/aim but does not discuss if this goal/aim is achievable

**3 = Done well** = Peer Volunteer talks to the mother about the goal that what is achievable through treatment and what is not, and mother and PV mutually decide the method/procedure of treatment

**Item 13: giving hope for achievable change**

**1 = Needs improvement** = Peer Volunteer either does not give any hope (i.e. you will never get well) or gives unrealistic hopes about the treatment and the betterment through it (i.e. you will get well within few weeks and there will be no issues after that/in future)

**2 = Done partially** = Peer Volunteer ambiguously tells the mother about what will happen during the treatment

**3 = Done well** = Peer Volunteer makes/helps the mother to feel positive about the future and gives her achievable hopes about what can and cannot be achieved through the treatment. Peer Volunteer analyzes the mother’s understanding of achievable change

**Item14: Talking about mental health according to the level of understanding of local people**

**1 = Needs Improvement** = Peer Volunteer uses complicated or embarrassing words while talking about mental health or she does not explain how the treatment would work

**2 = Done partially** = Peer Volunteer rarely uses any complicated words and does not use embarrassing words but she is unable to make the mother or the local people understand about the mother’s mental health

**3 = Done well** = Peer Volunteer uses local proverbs and non-embarrassing language, according to the level of understanding of mother and local people, to talk about mental health and makes sure that the mother understands

**Item 15: Steps for problem solving: 1st step: guiding how to recognize the unhealthy thinking; 2nd step: guiding how to replace the unhealthy thoughts with healthy thoughts; 3rd step: exercising and adopting the healthy thinking**

**1 = Needs improvement** = Peer Volunteer works with the mother to identify her unhealthy thinking

**2 = Done partially** = Peer Volunteer helps the mother to identify the unhealthy thoughts and changing it into healthy thinking

**3 = Done well** = Peer Volunteer helps the mother (1) to identify the unhealthy thoughts, (2) to change the unhealthy thoughts into healthy thoughts, (3) to decide about the work to be done for adopting the healthy thinking according to the health chart

**Item 16: Asking about (mother’s) opinion when a suggestion or advice is given while deciding the task**

**1 = Needs improvement** = Peer Volunteer tells the mother what to do without asking what she (mother) wants or what is easy/doable for her, or does not give any suggestion at all

**2 = Done partially** = Peer Volunteer explains to the mother in a focused manner, for example, tells her to sleep for 7 h at night while talking about rest chart but does not ask the mother if this suggestion is helpful for her

**3 = Done well** = When the mother asks, PV gives some advice and then asks about the mother’s opinion about the advice

**Item 17: Explaining and promoting (ensuring) privacy**

**1 = Needs improvement** = Peer Volunteer does not take care about privacy or does not talk according to the occasion

**2 = Done partially** = Peer Volunteer tells the mother that everything should be kept private but does not mention those things which could harm one’s self or anyone else are exempted. PV talked about private matters even while the session was not done privately

**3 = Done well** = Peer Volunteer explains to the mother that this conversation should be kept private except for the things that could harm her or anyone else. Peer Volunteer takes care of the fact that the session is private or not, and talks accordingly

**Item 18: Causing harm to one’s own self, causing harm to others, analyzing the harm caused by others, and mutually planning to deal with it**

**1 = Needs improvement** = Peer Volunteer does not ask about harming one’s own self or anyone else

**2 = Done partially** = Peer Volunteer explains about causing harm to one’s own self or others but does not help the mother in planning to deal with it

**3 = Done well** = Peer Volunteer talks about causing harm to one’s own self or others and guides the mother for an appropriate strategy
