# Supplementary material for: Piloting a psychosocial intervention for perinatal depression, the Thinking Healthy Programme–Peer delivered (THPP), in a primary care setting in Lilongwe District, Malawi
Source: PLOS Glob Public Health. 2024 May 1;4(5):e0002128. doi: 10.1371/journal.pgph.0002128 (PMC11062519; doi:10.1371/journal.pgph.0002128)
Supplement: S4 File — (DOCX) [file pgph.0002128.s004.docx]

**Interview guide – Peer volunteers, providers of the Thinking Healthy Intervention – Feasibility and Acceptability - English**

**UNIVERISTY OF MALAWI, COLLEGE OF MEDICINE DEPARTMENT OF MENTAL HEALTH**

**Title of Research Study:** Piloting a psychosocial intervention for perinatal depression, the Thinking Healthy Programme – Peer Delivered (THPP) in Malawi

**PI: Mwawi Ng’oma**

**Demographic details**

1. Age
2. Academic qualification
3. Marital status
4. Number of children
5. Residential address
6. Contact details

**Interview guide**

1. Have you ever been involved in any health care services as a volunteer before? Can you tell us more, your previous roles and experience?
2. Can you tell me more about the training you did for this intervention? Was it helpful and sufficient?
3. What has been your experience in delivering this intervention? Probe on complexity of the intervention, time required to deliver each session, support needed versus one offered, experiencing delivering the intervention in participants homes, experience working with family members, materials required?
4. Can you tell me more about participants attitudes towards intervention; adherence to sessions, completion of home work etc.
5. How has been your experiences working in partnership with PHC workers

**Interview guide – Peer volunteers, providers of the Thinking Healthy Intervention – Feasibility and Acceptability - Chichewa**

**UNIVERISTY OF MALAWI, COLLEGE OF MEDICINE DEPARTMENT OF MENTAL HEALTH**

**Title of Research Study:** Piloting a psychosocial intervention for perinatal depression, the Thinking Healthy Programme – Peer Delivered (THPP) in Malawi

**PI: Mwawi Ng’oma**

**Mbali yoyamba: Mbili**

1. Zaka za kubadwa

2. Maphunzilo

3. Okwatiwa/Osakwatiwa

4. Nambala ya ana

5. Mudzi/dela lanu

Keyala

6. Nambala ya phone/ dela lomwe mumakhala

**Mbali yachiwili: Mafunso - Feasibility**

7. Kodi munatengapo mbali mu ntchito za umoyo modzipeleka nokha mmbuyomu? Tandilongosoleleni mwatsatane tsatane za ntchitoyi, mbali yomwe inu mumatenga ndipo mumakumana ndi zotani (zabwino/ndi zovuta zomwe)

8. Mungandifotokozelepo za maphunzilo amene munapatsidwa okuyeneletsani kupeleka thandizo mukupelekali,

maphunzilowa anali okwanila ndipo ofunikila? longosolani

9. Kodi mukukumana ndi zotani mmene mukupeleka thandizoli?

**Fufuzani:** kuvuta kopeleka thandizoli, nthawi yomwe yikufunika kuti apeleke uphungu patsiku, thandizo lomwe iwo opeleka uphungu akulisowa kulingana ndi lomwe akulandila, kupeleka thandizo pa khomo pa/mumudzi mwa mayi, zomwe akukumana nazo popeleka thandizo/kugwira ntchito ndi banja, kaya ndi apongozi kapena amuna awo a amayi, zinthu zowathandizira popeleka thandizo/uphungu

10. Mungandifotokozelepo za kuikapo mtima kwa azimayi mu thandizoli, kodi ndiozipeleka kulandila uphungu/thandizo mundondomeko yake, akutha kukhala nanu nthawi zonse, nanga akutha kumaliza ntchito yomwe mwawapatsa yokhuzana ndi uphunguwu

11. Nanga ndi zinthu zotana zomwe mukukumana nazo pokhuzana ndi kugwira ntchito ndi madotolo a pachipatala.

**Mbali yachitatu: Mafunso - Acceptability**

1. Kodi mukukumana/mumakumana ndizotani mu ntchito yopeleka thandizo la uphungu la “kuganiza mwangwiro” kwa azimayi?

Ndi njila ziti mu thandizoli zomwe mumaziona kuti ndizophweka kuziphunzila ndi kuzipanga?

Nanga ndi zinthu ziti zomwe zikuthandizila/zimathandizila kupeleka thandizoli kapena zolepheletsa?

1. Ndi mbali yiti ya thandizoli yomwe yikuyenda bwino/yinayenda bwino, ndi mbali yiti yomwe siili /siyinali bwino kwenikweni. Ndi mbali yiti yomwe muli womasuka/muli ndikuthekera kupeleka bwinobwino, ndipo ndi mbali yiti yomwe yikuvuta/yimavuta?
2. Ndi mbali yiti ya thandizoli yomwe yikufunika kukonzedwa? Chifukwa chani? Pelekani maganizo anu mmene tingakonzere.
