# Supplementary material for: Piloting a psychosocial intervention for perinatal depression, the Thinking Healthy Programme–Peer delivered (THPP), in a primary care setting in Lilongwe District, Malawi
Source: PLOS Glob Public Health. 2024 May 1;4(5):e0002128. doi: 10.1371/journal.pgph.0002128 (PMC11062519; doi:10.1371/journal.pgph.0002128)
Supplement: S5 File — (DOCX) [file pgph.0002128.s005.docx]

**Interview guide, perinatal women receiving the THPP, Acceptability - English**

**UNIVERISTY OF MALAWI, COLLEGE OF MEDICINE DEPARTMENT OF MENTAL HEALTH**

**Title of Research Study:** Piloting a psychosocial intervention for perinatal depression, the Thinking Healthy Programme – Peer Delivered (THPP) in Malawi

**PI: Mwawi Ng’oma**

(**Demographic details will be recorded during recruitment)**

1. Can you tell me more about the intervention you are receiving?

What aspects of the intervention did you find more helpful/intuitive?

What did you like about this intervention and what you didn’t like?

What do you think were major barriers to receiving this intervention, and what were the facilitators?

1. Who in your family took part in the intervention?

Sessions they were involved in

Their attitude towards the intervention/Did they accept it?

The community’s attitude towards the intervention

1. What did you like about the providers/facilitators?

What are the preferred peer characteristics?

How did you relate to the intervention provider/facilitator?

1. Can you comment on place of delivery of the intervention i.e., in your home?

What were the advantages, disadvantage, and what is your preference?

1. What is your overall experience of this whole process?

How the intervention was delivered

Any changes in your feelings

Any changes in your home situation

1. Would you recommend this intervention to other pregnant women in your community?

Explain

1. Are there areas in this intervention that you think can be changed?

Give reasons for each

**Interview guide, perinatal women receiving the THPP, Acceptability - Chichewa**

**UNIVERISTY OF MALAWI, COLLEGE OF MEDICINE DEPARTMENT OF MENTAL HEALTH**

**Title of Research Study:** Piloting a psychosocial intervention for perinatal depression, the Thinking Healthy Programme – Peer Delivered (THPP) in Malawi

**PI: Mwawi Ng’oma**

**Mbili ya otenga mbali**

1. Zaka za kubadwa
2. Maphunzilo
3. Okwatiwa/Osakwatiwa
4. Mimba
5. Nambala ya ana amoyo/akufa
6. Mudzi/dela lanu
7. Keyala
8. Nambala ya phone/ dela lomwe mumakhala

**Mafunso**

1. Kodi mungandiuzepo zambiri za thandizo la uphungu lomwe mukulandira/munalandira

Kodi ndi gawo liti la uphungu lomwe mukuwona kuti ndilothandiza kwambiri, komanso lokupatsani danga kuti mulingalilepo pa zomwe mwakambilana. chifukwa chani?

Fotokozanipo zimene zakukomelani mu thandizo la uphunguli, ndiponso zomwe simunazikonde

Fotokozanipo za zipsyinjo zomwe zimalepheletse kapena kusokoneza kulandila uphungu. Nanga ndi chani chomwe chimathandizila kuti uphungu uyende bwino?

1. Fotokozanipo za zomwe zinakukomerani mwa opeleka uphungu?

Kodi ndimaKhalidwe /machitidwe a zinthu otani amene anali bwino kwambiri mwa wopeleka thandizo la uphungu?

Mgwilizano wanu unali wotani ndi opeleka thandizo la uphungu.

1. Munganenepo chani za malo amene thandizo la uphungu limapelekedwera. i.e., kunyumba/kumudzi kwanu?

Kodi komwe uphungu umapelekedwela kunathandizila bwanji, munali ndi vuto ndi malo amene uphungu umapelekedwera

Inu mungakonde kuti zikhale bwanji

1. Mungafotokozepo chani za momwe mwaonela thandizo lonse la uphungu ndinso momwe mukumvera.
