# Supplementary material for: Piloting a psychosocial intervention for perinatal depression, the Thinking Healthy Programme–Peer delivered (THPP), in a primary care setting in Lilongwe District, Malawi
Source: PLOS Glob Public Health. 2024 May 1;4(5):e0002128. doi: 10.1371/journal.pgph.0002128 (PMC11062519; doi:10.1371/journal.pgph.0002128)
Supplement: S7 File — (DOCX) [file pgph.0002128.s007.docx]

| 8/9/2021 8:08 AM | | | | | | | | | | | | | | | | | | | | |  |
| --- | --- | --- | --- | --- | --- | --- | --- | --- | --- | --- | --- | --- | --- | --- | --- | --- | --- | --- | --- | --- | --- |
| Coding Summary by File | | | | | | | | | | | | | | | | | | | | |  |
| THPP Acceptability | | | | | | | | | | | | | | | | | | | | |  |
| 8/9/2021 8:08 AM | | | | | | | | | | | | | | | | | | | | |  |
|  | **Classification** |  | **Aggregate** |  | **Coverage** |  | **Number Of Coding References** |  | | **Reference Number** | |  | | **Coded By Initials** | |  | | **Modified On** | |  | |
| **Document** | | | | | | | | | | | | | | | | | | |  | |  |
|  | **Files\\FGD\\FGD with mothers** | | | | | | | | | | | | | | | | | | |  | |
|  | **Code** | | | | | | | | | | | | | | | | | | |  | |
|  | **Codes\\Intervention experience** | | | | | | | | | | | | | | | | | | |  | |
|  |  |  | No |  | 0.1079 |  | 8 |  | | | | | | | | | | | |  |  |
|  |  |  |  |  |  |  |  |  | | | | | | | | | | | | | |
|  | | | | | | | | | | 1 |  | | MTK | |  | | 8/8/2021 6:01 PM | |  | |  |
|  | P8: I was happy when she was coming to provide counseling because we were learning on how we can deal with stress.  I: What was happening during the counseling? Number 4…  P4: There were lessons on replacing unhelpful behavior with helpful behavior. Like in my case, when I was pregnant, I was thinking… “Should I remove the pregnancy?” So I learned that it was not a helpful way of thinking.  I: What helped you to change your thoughts from unhelpful ones whereby you wanted to eliminate the pregnancy to have the helpful thoughts that you should maintain it?  P4: It was because of the counseling.  I: What about counseling?  P4: I should be a free person, I should not hate the relatives of my husband… I should be chatting with people… | | | | | | | | | | | | | | | | | | |  | |
|  |  |  |  |  |  |  |  |  |  |  |  |  |  |  |  |  |  |  |  |  |  |
|  |  | | | | | | | | | | | | | | | | | | |  | |
|  | | | | | | | | | | 2 |  | | MTK | |  | | 8/8/2021 6:10 PM | |  | |  |
|  | I: So how are your thoughts currently?  P: I don’t have any problem and I do everything as usual.  I: Thank you very much. I saw a hand from number 7…  P7: I had similar thoughts that have already been said.  I: Thank you. Are there others with different experiences from what others have said?  P: Relationship with others…  Note taker: You should raise your voice because you are behind…  P: When I learned about the relationship between the mother and the child and when I utilized it, I saw that my child is growing healthy. The thoughts that I had that I am poor and I cannot do anything, I found that they were not true because I am capable of doing things. | | | | | | | | | | | | | | | | | | |  | |
|  |  |  |  |  |  |  |  |  |  |  |  |  |  |  |  |  |  |  |  |  |  |
|  |  | | | | | | | | | | | | | | | | | | |  | |
|  | | | | | | | | | | 3 |  | | MTK | |  | | 8/8/2021 6:25 PM | |  | |  |
|  | I: Behind there, do you have something to say?  P: The counselors helped us to deal with the stress that we had. | | | | | | | | | | | | | | | | | | |  | |
|  |  | | | | | | | | | | | | | | | | | | |  | |
|  | | | | | | | | | | 4 |  | | MTK | |  | | 8/8/2021 6:29 PM | |  | |  |
|  | I: What did others say?  P: I was so depressed when I was pregnant with this child. Everyone in my area knew that I was depressed but now they see that I am living a healthy life and they ask me why? I tell them that it is the counseling that I receive which has helped me. I tell them that depression makes a pregnant woman feel like she is not worth living and she does not live a healthy life. I experienced the same but now I am a changed person and you also need to change. If you are happy, you need to go beyond where you are now. | | | | | | | | | | | | | | | | | | |  | |
|  |  | | | | | | | | | | | | | | | | | | |  | |
|  | | | | | | | | | | 5 |  | | MTK | |  | | 8/8/2021 6:34 PM | |  | |  |
|  | I: Okay. What do others say? How has counseling helped you?  P1: Counseling has helped us and this is the best intervention ever. We are changed people. We moved from where we were to another level.  I: Can you give an example of what changed?  P1: An example is that we were living in isolation without visiting friends but now we are able to visit friends and if you don’t have something, you ask your friend “I don’t have this, can you share me?” We are very thankful. | | | | | | | | | | | | | | | | | | |  | |
|  |  | | | | | | | | | | | | | | | | | | |  | |
| Formatted Reports\\Coding Summary by File Formatted Report | | | | | | | | | Page 1 of 145 | | | | | | | | | | | |  |
| 8/9/2021 8:08 AM | | | | | | | | | | | | | | | | | | | | |  |
|  | **Classification** |  | **Aggregate** |  | **Coverage** |  | **Number Of Coding References** |  | | **Reference Number** | |  | | **Coded By Initials** | |  | | **Modified On** | |  | |
|  |  |  |  |  |  |  |  |  |  |  |  |  |  |  |  |  |  |  |  |  |  |
|  | | | | | | | | | | 6 |  | | MTK | |  | | 8/8/2021 6:36 PM | |  | |  |
|  | I: Number 10?  P10: I stay near her and she knows what I have gone through and if there is someone who has suffered with problems, I am the one. Thoughts of eliminating the pregnancy were in me and thoughts of leaving the pregnancy were in me. I was not in talking terms with my mother in law. We could hardly visit each other nor support each other. When I received this counseling, I said that I needed to visit my mother in law. We currently visit each other and we are in good terms. | | | | | | | | | | | | | | | | | | |  | |
|  |  | | | | | | | | | | | | | | | | | | |  | |
|  | | | | | | | | | | 7 |  | | MTK | |  | | 8/8/2021 6:46 PM | |  | |  |
|  | I: Ladies, this counseling has left marks on us. What is the mark that you can point at? Number 6?  P6: I had nothing when my child was born and I did not have any clothes but after the birth of the child, my friends brought me clothes and assorted items for the child.  I: What is the difference with how things were before?  P6: I am able to differentiate because the other children that I have, no one gave me clothes but this one, I received clothes from my friends. | | | | | | | | | | | | | | | | | | |  | |
|  |  | | | | | | | | | | | | | | | | | | |  | |
|  | | | | | | | | | | 8 |  | | MTK | |  | | 8/8/2021 7:14 PM | |  | |  |
|  | I: How did this affect you considering the counseling that you were receiving?  P: It really had an impact on me because my relationship with my blood sister was very sour but since the counselor intervened, our relationship is very good and strong up to now. For my grandmother, she has bad behavior because the counselor tried her best but she chose not to take part. However, I learnt that it is very important to be in good relationship with my relatives. | | | | | | | | | | | | | | | | | | |  | |
|  |  | | | | | | | | | | | | | | | | | | |  | |
|  |  |  |  |  |  |  |  |  |  |  |  |  |  |  |  |  |  |  |  |  |  |
|  |  |  |  |  |  |  |  |  |  |  |  |  |  |  |  |  |  |  |  |  |  |
|  |  |  |  |  |  |  |  |  |  |  |  |  |  |  |  |  |  |  |  |  |  |
|  |  |  |  |  |  |  |  |  |  |  |  |  |  |  |  |  |  |  |  |  |  |
|  |  |  |  |  |  |  |  |  |  |  |  |  |  |  |  |  |  |  |  |  |  |
|  |  |  |  |  |  |  |  |  |  |  |  |  |  |  |  |  |  |  |  |  |  |
| Formatted Reports\\Coding Summary by File Formatted Report | | | | | | | | | Page 2 of 145 | | | | | | | | | | | |  |
| 8/9/2021 8:08 AM | | | | | | | | | | | | | | | | | | | | |  |
|  | **Classification** |  | **Aggregate** |  | **Coverage** |  | **Number Of Coding References** |  | | **Reference Number** | |  | | **Coded By Initials** | |  | | **Modified On** | |  | |
|  | **Codes\\Intervention experience\Aspects** | | | | | | | | | | | | | | | | | | |  | |
|  |  |  | No |  | 0.2046 |  | 5 |  | | | | | | | | | | | |  |  |
|  |  |  |  |  |  |  |  |  | | | | | | | | | | | | | |
|  | | | | | | | | | | 1 |  | | MTK | |  | | 8/8/2021 6:11 PM | |  | |  |
|  | I: Yes, number 8 you raised your hand…  P8: I was happy when she was coming to provide counseling because we were learning on how we can deal with stress.  I: What was happening during the counseling? Number 4…  P4: There were lessons on replacing unhelpful behavior with helpful behavior. Like in my case, when I was pregnant, I was thinking… “Should I remove the pregnancy?” So I learned that it was not a helpful way of thinking.  I: What helped you to change your thoughts from unhelpful ones whereby you wanted to eliminate the pregnancy to have the helpful thoughts that you should maintain it?  P4: It was because of the counseling.  I: What about counseling?  P4: I should be a free person, I should not hate the relatives of my husband… I should not keep to myself…  I: That is number 4, she has expressed her side. Remember we are not answering for each other. What do others say?  P10: We learned many things for example the issue of nutrition. Sometimes we were lazy to eat saying that “I will just wait for lunch.” When the counselor came, she encouraged us to eat in the morning during breakfast, before lunch, after lunch and supper. She taught us many things.  I: Others replaced unhelpful thoughts with helpful thoughts, others learned about nutrition… what do others say?  P7: I learnt about the health of the child on exclusive breastfeeding. During pregnancy, since I was impregnated by a certain boy who refused responsibility, I thought that I should just throw myself in the river but when we came here, we were told that the counselor would be coming home to provide counseling. When the counselor was coming for counseling sessions, I saw that my thoughts were not helpful and I noted that when the child starts eating other foods, I need to feed her in the morning, during lunch and in the evening just as my friend has said. I learned something.  I: Number 1?  P1: I learned about being in good relationship with my neighbors and letting my family help me to take care of the child. I did not know this before but I learnt a lot from this study. I learned that you cannot take care of the child single handedly but with the support of the family and others, raising of the child can be made easy.  I: Yes, number 9?  P9: I learned that we should relate well with our friends and that we should learn to be in groups so that we can be avoiding stress from the family. We also learnt that we should love our friends the way we love ourselves. This is what I have learned from the study.  I: Number 2?  P2: I am happy to learn about these things because we learnt that when we don’t have something, we should go to our friends and ask from them and if we have stress, we should be able to seek guidance from neighbors whom we trust.  P14: Number 14…  I: Yes number 14…  P14: I have learned that if you have friends, you live a happy life and many people like your child and taking care of the child cannot be a challenge is you have friends. For instance I was worried that “am I going to make it to take care of the child?” With this program, I have seen that I will be able to take care of the child.  I: Yes, number…?  P12: Number 12… This program has helped me to live positively because at first, I was thinking that “am I going to take care of the child?” My thoughts were to eliminate the pregnancy or to hang myself but after being counselled, I saw that what I was thinking was not helpful. I reached the extent that I delivered a live baby and she followed me thereafter.  I: Thank you very much…  P6: Number 6… I had no time to rest but when the counselor came, she told me that “you need to have time to rest after working.” I found it helpful because when I was pregnant, I used to overwork myself. Thank you.  P3: I concur with my friend. I was also not resting because I was staying at somebody’s home. After delivery, I had a problem of continuous menses for three consecutive month and when I came here at the hospital, I was given treatment. I took the medications for one week but there was no change and I came back here. They changed drugs and I saw improvement. When the counselor was coming, I was encouraged because a lot was said at home but I did not care about that because I knew that it is God who cares about me because he is the one who created me .  I: Meaning that you did not care about what people were saying?  P3: The one who was present during counseling was talking a lot about me with people. When the disease was continuing, there were a lot of issues that took place.  I: So how are your thoughts currently?  P: I don’t have any problem and I do everything as usual.  I: Thank you very much. I saw a hand from number 7…  P7: I had similar thoughts that have already been said.  I: Thank you. Are there others with different experiences from what others have said?  P: Relationship with others…  Note taker: You should raise your voice because you are behind…  P: When I learned about the relationship between the mother and the child and when I utilized it, I saw that my child is growing healthy.  The thoughts that I had that I am poor and I cannot do anything, I found that they were not true because I am capable of doing thigs.  I: Others? | | | | | | | | | | | | | | | | | | |  | |
|  |  |  |  |  |  |  |  |  |  |  |  |  |  |  |  |  |  |  |  |  |  |
|  |  |  |  |  |  |  |  |  |  |  |  |  |  |  |  |  |  |  |  |  |  |
|  |  |  |  |  |  |  |  |  |  |  |  |  |  |  |  |  |  |  |  |  |  |
|  |  |  |  |  |  |  |  |  |  |  |  |  |  |  |  |  |  |  |  |  |  |
|  |  |  |  |  |  |  |  |  |  |  |  |  |  |  |  |  |  |  |  |  |  |
|  |  |  |  |  |  |  |  |  |  |  |  |  |  |  |  |  |  |  |  |  |  |
|  |  |  |  |  |  |  |  |  |  |  |  |  |  |  |  |  |  |  |  |  |  |
|  |  |  |  |  |  |  |  |  |  |  |  |  |  |  |  |  |  |  |  |  |  |
| Formatted Reports\\Coding Summary by File Formatted Report | | | | | | | | | Page 3 of 145 | | | | | | | | | | | |  |
| 8/9/2021 8:08 AM | | | | | | | | | | | | | | | | | | | | |  |
|  | **Classification** |  | **Aggregate** |  | **Coverage** |  | **Number Of Coding References** |  | | **Reference Number** | |  | | **Coded By Initials** | |  | | **Modified On** | |  | |
|  | P: In addition to what she has said, when you love your child, s/he knows that you are showing her/him love. We need to talk to the children they hear.  I: Is that what you learned?  All: Yes!  P: Even during pregnancy, you can tell her/him that “my child I love you/” S/he hears you.  All: [Laughter]…  P9: Singing spiritual songs to the child…  I: Number 9?  P9: Singing spiritual songs to the child makes her/him feel happy.  I: Behind there, do you have something to say?  P: The counselors helped us to deal with the stress that we had. | | | | | | | | | | | | | | | | | | |  | |
|  |  |  |  |  |  |  |  |  |  |  |  |  |  |  |  |  |  |  |  |  |  |
|  |  | | | | | | | | | | | | | | | | | | |  | |
|  | | | | | | | | | | 2 |  | | MTK | |  | | 8/8/2021 6:26 PM | |  | |  |
|  | I: If you want to describe this counseling to someone, what can you tell them?  P14: It has helped me because most of the times I was not happy but when the counselor started coming, my stress was addressed and if someone can be depressed, I can tell her that “that will not help you. I had depression but it was addressed. I left everything.” I can tell her the way the counselor was teaching me so that she should also not be depressed but she should live a happy life.. | | | | | | | | | | | | | | | | | | |  | |
|  |  | | | | | | | | | | | | | | | | | | |  | |
|  | | | | | | | | | | 3 |  | | MTK | |  | | 8/8/2021 6:33 PM | |  | |  |
|  | I: Others, what do you say…? You mentioned a lot of things… there was an issue of stress, nutrition… taking care of the child…  P7: In terms of taking care of the child, the child is supposed to eat frequently. There are other mothers who were asking me why the counselor was visiting me only and I was just telling them what I was being taught by the counselors that “she taught me how to feed the child, helpful thinking. If you have unhelpful thoughts, you need to leave them and replace them with helpful thoughts.” | | | | | | | | | | | | | | | | | | |  | |
|  |  | | | | | | | | | | | | | | | | | | |  | |
|  | | | | | | | | | | 4 |  | | MTK | |  | | 8/8/2021 7:16 PM | |  | |  |
|  | I: Are there some additions? Why was it important to include relatives during the counseling sessions?  P14: It was important because we were learning about nutrition for example. So the involvement of the relatives was helpful because there was no way you could stay up to lunch hour without any food. The relatives would take part to give you some food because they would say “what we learned was that you need to eat frequently.” | | | | | | | | | | | | | | | | | | |  | |
|  |  | | | | | | | | | | | | | | | | | | |  | |
|  | | | | | | | | | | 5 |  | | MTK | |  | | 8/8/2021 7:16 PM | |  | |  |
|  | I: Number 16?  P16: [Part of the discussion not audible because of hissing sound of children in the background] The time to eat, time to rest and time to chat was to be adhered to. | | | | | | | | | | | | | | | | | | |  | |
|  |  | | | | | | | | | | | | | | | | | | |  | |
|  | **Codes\\Intervention experience\Facilitators** | | | | | | | | | | | | | | | | | | |  | |
|  |  |  | No |  | 0.0062 |  | 1 |  | | | | | | | | | | | |  |  |
|  |  |  |  |  |  |  |  |  | | | | | | | | | | | | | |
|  | | | | | | | | | | 1 |  | | MTK | |  | | 8/8/2021 7:08 PM | |  | |  |
|  | I: So what happened next?  P: I saw that most of them understood especially the one who was present during the counseling.  I: What happened for them to understand?  P: She was present during the first session with her husband. | | | | | | | | | | | | | | | | | | |  | |
|  |  | | | | | | | | | | | | | | | | | | |  | |
|  | **Codes\\Intervention experience\Influence on others** | | | | | | | | | | | | | | | | | | |  | |
|  |  |  | No |  | 0.0425 |  | 3 |  | | | | | | | | | | | |  |  |
|  |  |  |  |  |  |  |  |  | | | | | | | | | | | | | |
|  | | | | | | | | | | 1 |  | | MTK | |  | | 8/8/2021 6:27 PM | |  | |  |
|  | I: So it has helped you to deal with depression and also to help other people deal with depression. What do others say?  P: My comment is that it is good to deal with depression and we need to help our friends to deal with depression the way we were helped to deal with depression and this counseling strategy should not stop. | | | | | | | | | | | | | | | | | | |  | |
|  |  | | | | | | | | | | | | | | | | | | |  | |
|  |  |  |  |  |  |  |  |  |  |  |  |  |  |  |  |  |  |  |  |  |  |
| Formatted Reports\\Coding Summary by File Formatted Report | | | | | | | | | Page 4 of 145 | | | | | | | | | | | |  |
| 8/9/2021 8:08 AM | | | | | | | | | | | | | | | | | | | | |  |
|  | **Classification** |  | **Aggregate** |  | **Coverage** |  | **Number Of Coding References** |  | | **Reference Number** | |  | | **Coded By Initials** | |  | | **Modified On** | |  | |
|  |  |  |  |  |  |  |  |  |  |  |  |  |  |  |  |  |  |  |  |  |  |
|  | | | | | | | | | | 2 |  | | MTK | |  | | 8/8/2021 6:29 PM | |  | |  |
|  | I: What do others say?  P: I was so depressed when I was pregnant with this child. Everyone in my area knew that I was depressed but now they see that I am living a healthy life and they ask me why? I tell them that it is the counseling that I receive which has helped me. I tell them that depression makes a pregnant woman feel like she is not worth living and she does not live a healthy life. I experienced the same but now I am a changed person and you also need to change. If you are happy, you need to go beyond where you are now. | | | | | | | | | | | | | | | | | | |  | |
|  |  | | | | | | | | | | | | | | | | | | |  | |
|  | | | | | | | | | | 3 |  | | MTK | |  | | 8/8/2021 7:43 PM | |  | |  |
|  | I: So what would you tell other pregnant women about this counseling?  P14: If I can see a pregnant women who is depressed the way I was when I was pregnant, I can counsel her the way [Name of volunteer] counselled me so that she should be free from stress and live a healthy life and that when she delivers, she should take care of the child the way s/he is supposed to be taken care of.  I: that is number 14… Number 12?  P12: I had the same opinion.  P4: I can counsel the pregnant woman because I had the same experience. If she is stressed, the fetus inside also become stressed and I can tell her that “your child is stressed when you are not happy and you need to have good diet.” | | | | | | | | | | | | | | | | | | |  | |
|  |  |  |  |  |  |  |  |  |  |  |  |  |  |  |  |  |  |  |  |  |  |
|  |  | | | | | | | | | | | | | | | | | | |  | |
|  | **Codes\\Intervention experience\Likes** | | | | | | | | | | | | | | | | | | |  | |
|  |  |  | No |  | 0.0119 |  | 1 |  | | | | | | | | | | | |  |  |
|  |  |  |  |  |  |  |  |  | | | | | | | | | | | | | |
|  | | | | | | | | | | 1 |  | | MTK | |  | | 8/8/2021 5:59 PM | |  | |  |
|  | I: Yes, number 8 you raised your hand…  P8: I was happy when she was coming to provide counseling because we were learning on how we can deal with stress.  I: What was happening during the counseling? Number 4…  P4: There were lessons on replacing unhelpful behavior with helpful behavior. Like in my case, when I was pregnant, I was thinking… “Should I abort the pregnancy?” So I learned that it was not a helpful way of thinking. | | | | | | | | | | | | | | | | | | |  | |
|  |  | | | | | | | | | | | | | | | | | | |  | |
|  | **Codes\\Intervention experience\Misconceptions** | | | | | | | | | | | | | | | | | | |  | |
|  |  |  | No |  | 0.0736 |  | 7 |  | | | | | | | | | | | |  |  |
|  |  |  |  |  |  |  |  |  | | | | | | | | | | | | | |
|  | | | | | | | | | | 1 |  | | MTK | |  | | 8/8/2021 7:03 PM | |  | |  |
|  | I: Fine. Number 2?  P2: During the first visit, they came to confirm because people were saying that she was coming to give me ARVs.  All: [Laughter]….  Choral responses: Not audible.  P: They were also saying that to me…  P2: Then they saw that what they were thinking was not what was happening. | | | | | | | | | | | | | | | | | | |  | |
|  |  | | | | | | | | | | | | | | | | | | |  | |
|  | | | | | | | | | | 2 |  | | MTK | |  | | 8/8/2021 7:03 PM | |  | |  |
|  | I: That reminds me; how did the people around you react to the counseling? So she has started “they wanted to see if there were really ARVs [Chuckles]… What do others say?  P14: That was what they were saying… | | | | | | | | | | | | | | | | | | |  | |
|  |  | | | | | | | | | | | | | | | | | | |  | |
|  | | | | | | | | | | 3 |  | | MTK | |  | | 8/8/2021 7:03 PM | |  | |  |
|  | P: I only stayed with them once.  P: They were saying that “they are satanic group of people. Remember she was staying in [Name of location], she must have done something… They want to pick her up.” | | | | | | | | | | | | | | | | | | |  | |
|  |  | | | | | | | | | | | | | | | | | | |  | |
|  | | | | | | | | | | 4 |  | | MTK | |  | | 8/8/2021 7:04 PM | |  | |  |
|  | I: So let us be on the same page here; at first they have said that “she is coming to provide ARVs,” or “they are satanic…” How did their reactions change overtime after the counseling? Number 2?  P2: I did not know that they are saying that I receive ARVs. Then a friend of mine came. She found [Name of volunteer] counseling me. After the counseling, she confessed that “I was wondering they were saying that you come here to provide ARVs but this is good counseling and I have also learned.” Other people started coming during the counseling sessions to sit in. It was shameful because it was the aunt of my husband who was fabricating that. | | | | | | | | | | | | | | | | | | |  | |
|  |  | | | | | | | | | | | | | | | | | | |  | |
| Formatted Reports\\Coding Summary by File Formatted Report | | | | | | | | | Page 5 of 145 | | | | | | | | | | | |  |
| 8/9/2021 8:08 AM | | | | | | | | | | | | | | | | | | | | |  |
|  | **Classification** |  | **Aggregate** |  | **Coverage** |  | **Number Of Coding References** |  | | **Reference Number** | |  | | **Coded By Initials** | |  | | **Modified On** | |  | |
|  |  |  |  |  |  |  |  |  |  |  |  |  |  |  |  |  |  |  |  |  |  |
|  | | | | | | | | | | 5 |  | | MTK | |  | | 8/8/2021 7:05 PM | |  | |  |
|  | I: Okay thank you very much.  P: My mother in law confessed when she realized that it was not what people were saying. “Sorry we thought you were satanic people because that is what people were saying but this is a good intervention.” | | | | | | | | | | | | | | | | | | |  | |
|  |  | | | | | | | | | | | | | | | | | | |  | |
|  | | | | | | | | | | 6 |  | | MTK | |  | | 8/8/2021 7:06 PM | |  | |  |
|  | I: Okay thank you very much. Others?  P3: I heard from my husband. When he came back from where he went he said “Mother of [Name of child], people are saying that the counselor comes to give us ARVs.” I called my neighbor because she is the one who has been present during the counseling and she said “I heard this sometime back that you take ARVs. I wanted you to hear it by yourselves.” Then when the counselor came to tell me that I should come here for group session, when I went back home, that was what they were saying “people are saying that you have an infection. Why are you hiding the infection?” I was trying to explain to them that “she comes for counseling and this is what we discuss…” but there are some people who do not understand much but there are others who understand…. | | | | | | | | | | | | | | | | | | |  | |
|  |  |  |  |  |  |  |  |  |  |  |  |  |  |  |  |  |  |  |  |  |  |
|  |  | | | | | | | | | | | | | | | | | | |  | |
|  | | | | | | | | | | 7 |  | | MTK | |  | | 8/8/2021 7:08 PM | |  | |  |
|  | P4: They were saying “people are saying she provides ARVs but then where are they?” They were surprised when they heard that the discussion is about how to take care of the child and they said “the issue of ARVs is not true. We also need to have access to this counseling so that in future our children should be healthy.” | | | | | | | | | | | | | | | | | | |  | |
|  |  | | | | | | | | | | | | | | | | | | |  | |
|  |  |  |  |  |  |  |  |  |  |  |  |  |  |  |  |  |  |  |  |  |  |
|  |  |  |  |  |  |  |  |  |  |  |  |  |  |  |  |  |  |  |  |  |  |
|  |  |  |  |  |  |  |  |  |  |  |  |  |  |  |  |  |  |  |  |  |  |
|  |  |  |  |  |  |  |  |  |  |  |  |  |  |  |  |  |  |  |  |  |  |
|  |  |  |  |  |  |  |  |  |  |  |  |  |  |  |  |  |  |  |  |  |  |
|  |  |  |  |  |  |  |  |  |  |  |  |  |  |  |  |  |  |  |  |  |  |
| Formatted Reports\\Coding Summary by File Formatted Report | | | | | | | | | Page 6 of 145 | | | | | | | | | | | |  |
| 8/9/2021 8:08 AM | | | | | | | | | | | | | | | | | | | | |  |
|  | **Classification** |  | **Aggregate** |  | **Coverage** |  | **Number Of Coding References** |  | | **Reference Number** | |  | | **Coded By Initials** | |  | | **Modified On** | |  | |
|  | **Codes\\Intervention process\Delivery** | | | | | | | | | | | | | | | | | | |  | |
|  |  |  | No |  | 0.1627 |  | 2 |  | | | | | | | | | | | |  |  |
|  |  |  |  |  |  |  |  |  | | | | | | | | | | | | | |
|  | | | | | | | | | | 1 |  | | MTK | |  | | 8/8/2021 6:12 PM | |  | |  |
|  | P8: I was happy when she was coming to provide counseling because we were learning on how we can deal with stress.  I: What was happening during the counseling? Number 4…  P4: There were lessons on replacing unhelpful behavior with helpful behavior. Like in my case, when I was pregnant, I was thinking… “Should I remove the pregnancy?” So I learned that it was not a helpful way of thinking.  I: What helped you to change your thoughts from unhelpful ones whereby you wanted to eliminate the pregnancy to have the helpful thoughts that you should maintain it?  P4: It was because of the counseling.  I: What about counseling?  P4: I should be a free person, I should not hate the relatives of my husband… I should be chatting with people…  I: That is number 4, she has expressed her side. Remember we are not answering for each other. What do others say?  P10: We learned many things for example the issue of nutrition. Sometimes we were lazy to eat saying that “I will just wait for lunch.” When the counselor came, she encouraged us to eat in the morning during breakfast, before lunch, after lunch and supper. She taught us many things.  I: Others replaced unhelpful thoughts with helpful thoughts, others learned about nutrition… what do others say?  P7: I learnt about the health of the child on exclusive breastfeeding. During pregnancy, since I was impregnated by a certain boy who refused responsibility, I thought that I should just throw myself in the river but when we came here, we were told that the counselor would be coming home to provide counseling. When the counselor was coming for counseling sessions, I saw that my thoughts were not helpful and I noted that when the child starts eating other foods, I need to feed her in the morning, during lunch and in the evening just as my friend has said. I learned something.  I: Number 1?  P1: I learned about being in good relationship with my neighbors and letting my family help me to take care of the child. I did not know this before but I learnt a lot from this study. I learned that you cannot take care of the child single handedly but with the support of the family and others, the raising of the child can be made easy.  I: Yes, number 9?  P9: I learned that we should relate well with our friends and that we should learn to be in groups so that we can be avoiding stress from the family. We also learnt that we should love our friends the way we love ourselves. This is what I have learned from the study.  I: Number 2?  P2: I am happy to learn about these things because we learnt that when we don’t have something, we should go to our friends and ask from them and if we have stress, we should be able to seek guidance from neighbors whom we trust.  P14: Number 14…  I: Yes number 14…  P14: I have learned that if you have friends, you live a happy life and many people like your child and taking care of the child cannot be a challenge is you have friends. For instance I was worried that “am I going to make it to take care of the child?” With this program, I have seen that I will be able to take care of the child.  I: Yes, number…?  P12: Number 12… This program has helped me to live positively because at first, I was thinking that “am I going to take care of the child?” My thoughts were to eliminate the pregnancy or to hang myself but after being counselled, I saw that what I was thinking was not helpful. I reached the extent that I delivered a live baby and she followed me thereafter.  I: Thank you very much…  P6: Number 6… I had no time to rest but when the counselor came, she told me that “you need to have time to rest after working.” I found it helpful because when I was pregnant, I used to overwork myself. Thank you.  P3: I concur with my friend. I was also not resting because I was staying at somebody’s home. After delivery, I had a problem of continuous menses for three consecutive month and when I came here at the hospital, I was given treatment. I took the medications for one week but there was no change and I came back here. They changed drugs and I saw improvement. When the counselor was coming, I was encouraged because a lot was said at home but I sis not care about them because I knew that it is God who cares about me because he is the one who created me .  I: Meaning that you did not care about what people were saying?  P3: The one who was present during counseling was talking a lot about me with people. When the disease was continuing, there were a lot of issues that took place.  I: So how are your thoughts currently?  P: I don’t have any problem and I do everything as usual.  I: Thank you very much. I saw a hand from number 7…  P7: I had similar thoughts that have already been said.  I: Thank you. Are there others with different experiences from what others have said?  P: Relationship with others…  Note taker: You should raise your voice because you are behind…  P: When I learned about the relationship between the mother and the child and when I utilized it, I saw that my child is growing healthy. The thoughts that I had that I am poor and I cannot do anything, I found that they were not true because I am capable of doing things.  I: Others?  P: In addition to what she has said, when you love your child, s/he knows that you are showing her/him love. We need to talk to the | | | | | | | | | | | | | | | | | | |  | |
|  |  |  |  |  |  |  |  |  |  |  |  |  |  |  |  |  |  |  |  |  |  |
|  |  |  |  |  |  |  |  |  |  |  |  |  |  |  |  |  |  |  |  |  |  |
|  |  |  |  |  |  |  |  |  |  |  |  |  |  |  |  |  |  |  |  |  |  |
|  |  |  |  |  |  |  |  |  |  |  |  |  |  |  |  |  |  |  |  |  |  |
|  |  |  |  |  |  |  |  |  |  |  |  |  |  |  |  |  |  |  |  |  |  |
|  |  |  |  |  |  |  |  |  |  |  |  |  |  |  |  |  |  |  |  |  |  |
|  |  |  |  |  |  |  |  |  |  |  |  |  |  |  |  |  |  |  |  |  |  |
|  |  |  |  |  |  |  |  |  |  |  |  |  |  |  |  |  |  |  |  |  |  |
| Formatted Reports\\Coding Summary by File Formatted Report | | | | | | | | | Page 7 of 145 | | | | | | | | | | | |  |
| 8/9/2021 8:08 AM | | | | | | | | | | | | | | | | | | | | |  |
|  | **Classification** |  | **Aggregate** |  | **Coverage** |  | **Number Of Coding References** |  | | **Reference Number** | |  | | **Coded By Initials** | |  | | **Modified On** | |  | |
|  | children they hear.  I: Is that what you learned?  All: Yes! | | | | | | | | | | | | | | | | | | |  | |
|  |  | | | | | | | | | | | | | | | | | | |  | |
|  | | | | | | | | | | 2 |  | | MTK | |  | | 8/8/2021 6:33 PM | |  | |  |
|  | I: Others, what do you say…? You mentioned a lot of things… there was an issue of stress, nutrition… taking care of the child…  P7: In terms of taking care of the child, the child is supposed to eat frequently. There are other mothers who were asking me why the counselor was visiting me only and I was just telling them what I was being taught by the counselor | | | | | | | | | | | | | | | | | | |  | |
|  |  | | | | | | | | | | | | | | | | | | |  | |
|  | **Codes\\Intervention process\Feelings** | | | | | | | | | | | | | | | | | | |  | |
|  |  |  | No |  | 0.0054 |  | 1 |  | | | | | | | | | | | |  |  |
|  |  |  |  |  |  |  |  |  | | | | | | | | | | | | | |
|  | | | | | | | | | | 1 |  | | MTK | |  | | 8/8/2021 7:06 PM | |  | |  |
|  | I: Thank you.  P: I told her that “I heard everything but I did not want to argue because I knew that you were saying that because you did not know and it is good not that you have known by yourself…” | | | | | | | | | | | | | | | | | | |  | |
|  |  | | | | | | | | | | | | | | | | | | |  | |
|  | **Codes\\Intervention process\Home situation** | | | | | | | | | | | | | | | | | | |  | |
|  |  |  | No |  | 0.0211 |  | 3 |  | | | | | | | | | | | |  |  |
|  |  |  |  |  |  |  |  |  | | | | | | | | | | | | | |
|  | | | | | | | | | | 1 |  | | MTK | |  | | 8/8/2021 6:35 PM | |  | |  |
|  | I: Can you give an example of what changed?  P1: An example is that we were living in isolation without visiting friends but now we are able to visit friends and if you don’t have something, you ask your friend “I don’t have this, can you share me? Or I need help” We are very thankful. | | | | | | | | | | | | | | | | | | |  | |
|  |  | | | | | | | | | | | | | | | | | | |  | |
|  | | | | | | | | | | 2 |  | | MTK | |  | | 8/8/2021 6:35 PM | |  | |  |
|  | I: you are able to ask from others?  P1: Very much so. | | | | | | | | | | | | | | | | | | |  | |
|  |  | | | | | | | | | | | | | | | | | | |  | |
|  | | | | | | | | | | 3 |  | | MTK | |  | | 8/8/2021 7:12 PM | |  | |  |
|  | I: Or why was it important to have relatives around when you were receiving the counseling intervention? [Sound of children playing in the background]  P7: It was important because the work that I was doing at home was beyond my capacity as a pregnant woman and the inclusion of my mother in law helped a lot because that helped me to be relieved from hard work. Even my father in law admitted that I don’t need to work hard after the counseling. | | | | | | | | | | | | | | | | | | |  | |
|  |  | | | | | | | | | | | | | | | | | | |  | |
|  |  |  |  |  |  |  |  |  |  |  |  |  |  |  |  |  |  |  |  |  |  |
|  |  |  |  |  |  |  |  |  |  |  |  |  |  |  |  |  |  |  |  |  |  |
|  |  |  |  |  |  |  |  |  |  |  |  |  |  |  |  |  |  |  |  |  |  |
| Formatted Reports\\Coding Summary by File Formatted Report | | | | | | | | | Page 8 of 145 | | | | | | | | | | | |  |
| 8/9/2021 8:08 AM | | | | | | | | | | | | | | | | | | | | |  |
|  | **Classification** |  | **Aggregate** |  | **Coverage** |  | **Number Of Coding References** |  | | **Reference Number** | |  | | **Coded By Initials** | |  | | **Modified On** | |  | |
|  |  | | | | | | | | | | | | | | | | | | |  | |
|  |  | | | | | | | | | | | | | | | | | | |  | |
|  |  |  |  |  |  |  |  |  |  |  |  |  |  |  |  |  |  |  |  |  |  |
|  |  |  |  |  |  |  |  |  |  |  |  |  |  |  |  |  |  |  |  |  |  |
|  |  |  |  |  |  |  |  |  |  |  |  |  |  |  |  |  |  |  |  |  |  |
|  |  | | | | | | | | | | | | | | | | | | |  | |
|  | **Codes\\Other interesting issues** | | | | | | | | | | | | | | | | | | |  | |
|  |  |  | No |  | 0.0641 |  | 5 |  | | | | | | | | | | | |  |  |
|  |  |  |  |  |  |  |  |  | | | | | | | | | | | | | |
|  | | | | | | | | | | 1 |  | | MTK | |  | | 8/8/2021 6:23 PM | |  | |  |
|  | I: Is that what you learned?  All: Yes!  P: Even during pregnancy, you can tell her/him that “my child I love you/” S/he hears you.  All: [Laughter]…  P9: Singing spiritual songs to the child…  I: Number 9?  P9: Singing spiritual songs to the child makes her/him feel happy. | | | | | | | | | | | | | | | | | | |  | |
|  |  |  |  |  |  |  |  |  |  |  |  |  |  |  |  |  |  |  |  |  |  |
|  |  | | | | | | | | | | | | | | | | | | |  | |
|  | | | | | | | | | | 2 |  | | MTK | |  | | 8/8/2021 6:34 PM | |  | |  |
|  | I: Okay. What do others say? How has counseling helped you?  P1: Counseling has helped us and this is the best intervention ever. We are changed people. We moved from where we were to another level.  I: Can you give an example of what changed?  P1: An example is that we were living in isolation without visiting friends but now we are able to visit friends and if you don’t have something, you ask your friend “I don’t have this, can you share me? Or I need help” We are very thankful. | | | | | | | | | | | | | | | | | | |  | |
|  |  | | | | | | | | | | | | | | | | | | |  | |
| Formatted Reports\\Coding Summary by File Formatted Report | | | | | | | | | Page 9 of 145 | | | | | | | | | | | |  |
| 8/9/2021 8:08 AM | | | | | | | | | | | | | | | | | | | | |  |
|  | **Classification** |  | **Aggregate** |  | **Coverage** |  | **Number Of Coding References** |  | | **Reference Number** | |  | | **Coded By Initials** | |  | | **Modified On** | |  | |
|  |  |  |  |  |  |  |  |  |  |  |  |  |  |  |  |  |  |  |  |  |  |
|  | | | | | | | | | | 3 |  | | MTK | |  | | 8/8/2021 6:35 PM | |  | |  |
|  | I: Number 10?  P10: I stay near her and she knows what I have gone through and if there is someone who has suffered with problems, I am the one. Thoughts of eliminating the pregnancy were in me and thoughts of leaving the pregnancy were in me. I was not talking with my mother in law. We could hardly visit each other nor support each other. When I received this counseling, I said that I needed to visit my mother in law. We currently visit each other and we are in good terms. | | | | | | | | | | | | | | | | | | |  | |
|  |  | | | | | | | | | | | | | | | | | | |  | |
|  | | | | | | | | | | 4 |  | | MTK | |  | | 8/8/2021 7:07 PM | |  | |  |
|  | I: Okay thank you very much. Others?  P3: I heard from my husband. When he came back from where he went he said “Mother of [Name of child], people are saying that the counselor comes to give us ARVs.” I called my neighbor because she is the one who has been present during the counseling and she said “I heard this sometime back that you take ARVs. I wanted you to hear it by yourselves.” Then when the counselor came to tell me that I should come here for group session, when I went back home, that was what they were saying “people are saying that you have an infection. Why are you hiding the infection?” I was trying to explain to them that “she comes for counseling and this is what we discuss…” but there are some people who do not understand much as there are others who understand…. | | | | | | | | | | | | | | | | | | |  | |
|  |  |  |  |  |  |  |  |  |  |  |  |  |  |  |  |  |  |  |  |  |  |
|  |  | | | | | | | | | | | | | | | | | | |  | |
|  | | | | | | | | | | 5 |  | | MTK | |  | | 8/8/2021 8:09 PM | |  | |  |
|  | I: Is there anything you would have loved that we should discuss concerning our discussion?  All: [No response]  I: Or anything you would want to share?  P9: It is reducing cases of abuse.  Note taker: What did you say?  P9: Counseling is reducing cases of abuses.  P14: And domestic violence is reduced in the families because of counseling. | | | | | | | | | | | | | | | | | | |  | |
|  |  |  |  |  |  |  |  |  |  |  |  |  |  |  |  |  |  |  |  |  |  |
|  |  | | | | | | | | | | | | | | | | | | |  | |
|  | **Codes\\Participation of others** | | | | | | | | | | | | | | | | | | |  | |
|  |  |  | No |  | 0.1213 |  | 7 |  | | | | | | | | | | | |  |  |
|  |  |  |  |  |  |  |  |  | | | | | | | | | | | | | |
|  | | | | | | | | | | 1 |  | | MTK | |  | | 8/8/2021 6:38 PM | |  | |  |
|  | I: Can you give an example of what changed?  P1: An example is that we were living in isolation without visiting friends but now we are able to visit friends and if you don’t have something, you ask your friend “I don’t have this, can you share me? Or I need help” We are very thankful.  I: you are able to ask from others?  P1: Very much so.  I: Number 10?  P10: I stay near her and she knows what I have gone through and if there is someone who has suffered with problems, I am the one. Thoughts of eliminating the pregnancy were in me and thoughts of leaving the pregnancy were in me. I was not talking with my mother in law. We could hardly visit each other nor support each other. When I received this counseling, I said that I needed to visit my mother in law. We currently visit each other and we are in good terms. | | | | | | | | | | | | | | | | | | |  | |
|  |  |  |  |  |  |  |  |  |  |  |  |  |  |  |  |  |  |  |  |  |  |
|  |  | | | | | | | | | | | | | | | | | | |  | |
|  |  |  |  |  |  |  |  |  |  |  |  |  |  |  |  |  |  |  |  |  |  |
|  |  |  |  |  |  |  |  |  |  |  |  |  |  |  |  |  |  |  |  |  |  |
| Formatted Reports\\Coding Summary by File Formatted Report | | | | | | | | | Page 10 of 145 | | | | | | | | | | | |  |
| 8/9/2021 8:08 AM | | | | | | | | | | | | | | | | | | | | |  |
|  | **Classification** |  | **Aggregate** |  | **Coverage** |  | **Number Of Coding References** |  | | **Reference Number** | |  | | **Coded By Initials** | |  | | **Modified On** | |  | |
|  |  |  |  |  |  |  |  |  |  |  |  |  |  |  |  |  |  |  |  |  |  |
|  | | | | | | | | | | 4 |  | | MTK | |  | | 8/8/2021 7:12 PM | |  | |  |
|  | I: Or why was it important to have relatives around when you were receiving the counseling intervention? [Sound of children playing in the background]  P7: It was important because the work that I was doing at home was beyond my capacity as a pregnant woman and the inclusion of my mother in law helped a lot because that helped me to be relieved from hard work. Even my father in law admitted that I don’t need to work hard after the counseling. | | | | | | | | | | | | | | | | | | |  | |
|  |  | | | | | | | | | | | | | | | | | | |  | |
|  | | | | | | | | | | 5 |  | | MTK | |  | | 8/8/2021 7:14 PM | |  | |  |
|  | I: That was number 7. Others?  P: There was great enmity between me and my sister and my grandmother. She gave me some cloths but later took them back. When the counselor came, we discussed good relationship between the mother and the people around her, and she asked me if I was comfortable to invite my sister and grandmother to be part of the sessions. I accepted. When they came, the counselor explained everything to them and emphasized on the importance of good relationship with relatives. She did not disclose what I told her but she went through the session. Since then, my sister changed and we are in good relationship up to now but my grandmother changed only for two weeks and later started hating me again. I left her home because of her behavior and I stay alone. | | | | | | | | | | | | | | | | | | |  | |
|  |  |  |  |  |  |  |  |  |  |  |  |  |  |  |  |  |  |  |  |  |  |
|  |  | | | | | | | | | | | | | | | | | | |  | |
|  | | | | | | | | | | 6 |  | | MTK | |  | | 8/8/2021 7:15 PM | |  | |  |
|  | I: How did this affect you considering the counseling that you were receiving?  P: It really had an impact on me because my relationship with my blood sister was very sour but since the counselor intervened, our relationship is very good and strong up to now. For my grandmother, she has bad behavior because the counselor tried her best but she chose not to take part. However, I learnt that it is very important to be in good relationship with my relatives. | | | | | | | | | | | | | | | | | | |  | |
|  |  | | | | | | | | | | | | | | | | | | |  | |
|  | | | | | | | | | | 7 |  | | MTK | |  | | 8/8/2021 7:24 PM | |  | |  |
|  | P8: It is better to have the counseling session at home so that your mother in law should hear by herself whatever is happening.  I: Tell us more…  P8: If she does not give you food, she should know the importance of giving you food.  P: I agree to this one. It is important that the counseling sessions should be done at home because you can invite the family and if there are issues, they should be discussed there and the counselor should know what is happening.  P9: When there was something sensitive, the counselor was asking the other people to go out so that they should not hear what was being discussed between you and the counselor. So the counseling should be done at home but when there are sensitive issues, the other people should not be present they should give room for private discussion between you and the counselor because if they are present, you cannot be free. The issue of food is general but there are some confidential issues that don’t require big audience. | | | | | | | | | | | | | | | | | | |  | |
|  |  |  |  |  |  |  |  |  |  |  |  |  |  |  |  |  |  |  |  |  |  |
|  |  | | | | | | | | | | | | | | | | | | |  | |
|  | **Codes\\Participation of others\Attitude** | | | | | | | | | | | | | | | | | | |  | |
|  |  |  | No |  | 0.1023 |  | 12 |  | | | | | | | | | | | |  |  |
|  |  |  |  |  |  |  |  |  | | | | | | | | | | | | | |
|  | | | | | | | | | | 1 |  | | MTK | |  | | 8/8/2021 7:05 PM | |  | |  |
|  | I: So let us be on the same page here; at first they have said that “she is coming to provide ARVs,” or “they are satanic…” How did their reactions change overtime after the counseling? Number 2?  P2: I did not know that they are saying that I receive ARVs. Then a friend of mine came. She found [Name of volunteer] counseling me. After the counseling, she confessed that “I was wondering they were saying that you come here to provide ARVs but this is good counseling and I have also learned.” Other people started coming during the counseling sessions to sit in. It was shameful because it was the aunt of my husband who was fabricating that. | | | | | | | | | | | | | | | | | | |  | |
|  |  | | | | | | | | | | | | | | | | | | |  | |
|  | | | | | | | | | | 2 |  | | MTK | |  | | 8/8/2021 7:05 PM | |  | |  |
|  | I: Okay thank you very much.  P: My mother in law confessed when she realized that it was not what people were saying. “Sorry we thought you were satanic people because that is what people were saying but this is a good intervention.” | | | | | | | | | | | | | | | | | | |  | |
|  |  | | | | | | | | | | | | | | | | | | |  | |
|  |  |  |  |  |  |  |  |  |  |  |  |  |  |  |  |  |  |  |  |  |  |
|  |  | | | | | | | | | | | | | | | | | | |  | |
|  |  |  |  |  |  |  |  |  |  |  |  |  |  |  |  |  |  |  |  |  |  |
|  |  | | | | | | | | | | | | | | | | | | |  | |
| Formatted Reports\\Coding Summary by File Formatted Report | | | | | | | | | Page 11 of 145 | | | | | | | | | | | |  |
| 8/9/2021 8:08 AM | | | | | | | | | | | | | | | | | | | | |  |
|  | **Classification** |  | **Aggregate** |  | **Coverage** |  | **Number Of Coding References** |  | | **Reference Number** | |  | | **Coded By Initials** | |  | | **Modified On** | |  | |
|  |  |  |  |  |  |  |  |  |  |  |  |  |  |  |  |  |  |  |  |  |  |
|  | | | | | | | | | | 4 |  | | MTK | |  | | 8/8/2021 7:08 PM | |  | |  |
|  | P4: They were saying “people are saying she provides ARVs but then where are they?” They were surprised when they heard that the discussion is about how to take care of the child and they said “the issue of ARVs is not true. We also need to have access to this counseling so that in future our children should be healthy.” | | | | | | | | | | | | | | | | | | |  | |
|  |  | | | | | | | | | | | | | | | | | | |  | |
|  | | | | | | | | | | 5 |  | | MTK | |  | | 8/8/2021 7:09 PM | |  | |  |
|  | I: Number 15? How did your relatives react to the counseling?  P15: They accepted it and when the counselor was coming, she was finding that the people were there waiting. | | | | | | | | | | | | | | | | | | |  | |
|  |  | | | | | | | | | | | | | | | | | | |  | |
|  | | | | | | | | | | 6 |  | | MTK | |  | | 8/8/2021 7:09 PM | |  | |  |
|  | I: Were there experiences of opposition from the people surrounding you?  P: The relatives of my husband called for me to ask “where is the person who comes to your house coming from?” I explained everything to them. Then because my neighbor always go there to gossip about me and those people tell me everything that my neighbor tells them. So I just leave her like that because there was nothing else I could do. | | | | | | | | | | | | | | | | | | |  | |
|  |  | | | | | | | | | | | | | | | | | | |  | |
|  | | | | | | | | | | 7 |  | | MTK | |  | | 8/8/2021 7:09 PM | |  | |  |
|  | I: meaning that those people were talking about things which they were told by your neighbor?  P: Yes.  I: But your neighbor went to another person…  P: Yes but I explained to them everything in details. | | | | | | | | | | | | | | | | | | |  | |
|  |  | | | | | | | | | | | | | | | | | | |  | |
|  | | | | | | | | | | 8 |  | | MTK | |  | | 8/8/2021 7:11 PM | |  | |  |
|  | I: And what happened next?  P: Nothing happened because after explaining to them, they understood everything. | | | | | | | | | | | | | | | | | | |  | |
|  |  | | | | | | | | | | | | | | | | | | |  | |
|  | | | | | | | | | | 9 |  | | MTK | |  | | 8/8/2021 7:11 PM | |  | |  |
|  | I: Thank you very much. So since you are many, I would like to hear from someone who faced negative reaction from relatives. Reactions against the counseling… we would like to hear those experiences.  P: On that one, there can be no experiences of negative reactions because in most cases, pregnant women are in contact with health workers and no one can be against that. | | | | | | | | | | | | | | | | | | |  | |
|  |  | | | | | | | | | | | | | | | | | | |  | |
|  | | | | | | | | | | 10 |  | | MTK | |  | | 8/8/2021 7:28 PM | |  | |  |
|  | P: Someone came to get my health profile, she checked where HIV testing results are indicated and when she saw the results she did not have anything to say. | | | | | | | | | | | | | | | | | | |  | |
|  |  | | | | | | | | | | | | | | | | | | |  | |
|  | | | | | | | | | | 11 |  | | MTK | |  | | 8/8/2021 8:12 PM | |  | |  |
|  | Note taker: I asked this question because she asked a question about how the family accepted the intervention and how the community perceived it but we also wanted to hear about how the husbands perceived it…  P1: They accepted it. | | | | | | | | | | | | | | | | | | |  | |
|  |  | | | | | | | | | | | | | | | | | | |  | |
|  | | | | | | | | | | 12 |  | | MTK | |  | | 8/8/2021 8:12 PM | |  | |  |
|  | Note taker: What do others say?  P9, P11, P12, P14, P10, P6, P4, P3: Agreed with what number 1 said | | | | | | | | | | | | | | | | | | |  | |
|  |  | | | | | | | | | | | | | | | | | | |  | |
|  | **Codes\\Participation of others\Community participation** | | | | | | | | | | | | | | | | | | |  | |
|  |  |  | No |  | 0.0521 |  | 4 |  | | | | | | | | | | | |  |  |
|  |  |  |  |  |  |  |  |  | | | | | | | | | | | | | |
|  | | | | | | | | | | 1 |  | | MTK | |  | | 8/8/2021 7:16 PM | |  | |  |
|  | I: Okay fine. Are there other additions? We should now move from the close relatives… how did the community perceive the counseling intervention?  P4: When people understood that I don’t take ARVs, the community liked this intervention and they said that the counseling should continue. | | | | | | | | | | | | | | | | | | |  | |
|  |  | | | | | | | | | | | | | | | | | | |  | |
| Formatted Reports\\Coding Summary by File Formatted Report | | | | | | | | | Page 12 of 145 | | | | | | | | | | | |  |
| 8/9/2021 8:08 AM | | | | | | | | | | | | | | | | | | | | |  |
|  | **Classification** |  | **Aggregate** |  | **Coverage** |  | **Number Of Coding References** |  | | **Reference Number** | |  | | **Coded By Initials** | |  | | **Modified On** | |  | |
|  |  |  |  |  |  |  |  |  |  |  |  |  |  |  |  |  |  |  |  |  |  |
|  | | | | | | | | | | 2 |  | | MTK | |  | | 8/8/2021 7:17 PM | |  | |  |
|  | I: When the community understood the concept of counseling, they accepted it and said it should continue. What about others?  P9: I had a similar experience. | | | | | | | | | | | | | | | | | | |  | |
|  |  | | | | | | | | | | | | | | | | | | |  | |
|  | | | | | | | | | | 3 |  | | MTK | |  | | 8/8/2021 7:17 PM | |  | |  |
|  | I: Others…? Because it is possible to accept the intervention as a family but the community can be against it and if they can see the counselors, they can reach the extent of chasing them… “We don’t want this here…”  P14: In my community, when people saw that [Name of volunteer] has come, they were sending their children who are pregnant and those with young children so that they should come and benefit from the counseling intervention.  I: Others?  P6: My community is more than willing and they would like to benefit like wise because when they hear that I have been called here at the hospital, pregnant women wish if they were part of the intervention so that they can also benefit from the service that I am accessing.  I: [There is noise of women passing by crying/mourning in the background. Audio was paused]… Okay… we were discussing about the community… how they reacted. Are there additions.  P12: The counseling was accepted by the community because when the counselor was coming, even married people who are not pregnant in the community wished if they could have an opportunity to access the counseling so that they can be able to deal with stress and depression | | | | | | | | | | | | | | | | | | |  | |
|  |  |  |  |  |  |  |  |  |  |  |  |  |  |  |  |  |  |  |  |  |  |
|  |  | | | | | | | | | | | | | | | | | | |  | |
|  | | | | | | | | | | 4 |  | | MTK | |  | | 8/8/2021 7:17 PM | |  | |  |
|  | I: Others?  P: My community is very happy with the counseling because they see how I have changed and they admit that “there is great difference from the way we have been seeing you. It seems the women who was coming has really helped you.” The chief is happy with the intervention. | | | | | | | | | | | | | | | | | | |  | |
|  |  | | | | | | | | | | | | | | | | | | |  | |
|  |  |  |  |  |  |  |  |  |  |  |  |  |  |  |  |  |  |  |  |  |  |
|  |  |  |  |  |  |  |  |  |  |  |  |  |  |  |  |  |  |  |  |  |  |
|  |  |  |  |  |  |  |  |  |  |  |  |  |  |  |  |  |  |  |  |  |  |
|  |  |  |  |  |  |  |  |  |  |  |  |  |  |  |  |  |  |  |  |  |  |
|  |  |  |  |  |  |  |  |  |  |  |  |  |  |  |  |  |  |  |  |  |  |
| Formatted Reports\\Coding Summary by File Formatted Report | | | | | | | | | Page 13 of 145 | | | | | | | | | | | |  |
| 8/9/2021 8:08 AM | | | | | | | | | | | | | | | | | | | | |  |
|  | **Classification** |  | **Aggregate** |  | **Coverage** |  | **Number Of Coding References** |  | | **Reference Number** | |  | | **Coded By Initials** | |  | | **Modified On** | |  | |
|  | **Codes\\Participation of others\Others** | | | | | | | | | | | | | | | | | | |  | |
|  |  |  | No |  | 0.0996 |  | 3 |  | | | | | | | | | | | |  |  |
|  |  |  |  |  |  |  |  |  | | | | | | | | | | | | | |
|  | | | | | | | | | | 1 |  | | MTK | |  | | 8/8/2021 6:56 PM | |  | |  |
|  | I: Now going back to the counseling room… The counselor has come… who was with you in the counseling session? Number 1?  P1: I was with my family.  I: number 2?  P: On the first day, I was alone but on the second day I was with my partner.  I: Number 3?  P3: I was with my sister in marriage and her husband and my husband like four people.  I: Oh, four people… Number 4?  P4: I was with my family.  I: Number 5?  P5: I was with my sister.  I: Number…?  P6: I was with my husband.  P: I was with my husband and my mother in law.  I: What is your number?  P7: 7.  I: Number 8?  P8: I was with my husband and my mother in law.  I: Number 9?  P9: I was with my husband and my mother in law.  I: Number 10?  P10: I was with my husband.  I: Number 11?  P11: I was with my mother in law.  I: Number 12?  P12: I was alone the first day but the second day I was with my parents.  I: Number 13?  P13: I was with my sister and my grandmother.  I: Number 14?  P14: The first day I was with my husband but from the second day I was with my family. There was also a group of women waiting for the counselor.  I: Number 15?  P15: [No response]  I: Number 16?  P: I was with my sister.  I: So we have seen that each one of you had someone around during the sessions… So which one of you have a family member during all the visits?  P14: There was my grandmother, my sister, my sister in law.  I: What about those expectant women?  P14: She has delivered and she has a baby boy.  I: She was present during all the visits…  P14: Yes.  I:Okay. Number 2?  P2: I was with my sister in marriage, my partner and the women I chat with.  I: They were all present during al the visits?  P2: Yes.  I: Fine. Others? Number 4  P: My grandmother, for six visits.  I: Others?  P: My mother in law, my husband, his father and his grandmother.  I: On how many visits?  P: All of them  I: Fine. Others?  P10: I was with my sister in marriage.  I: You?  P: I was with my sister…  I: Number 6?  P6: I was only with my husband for the five visits  P12: For all the visits I was with my mother except one when I was alone.  I: Number 11?  P11: I was with my mother in law. | | | | | | | | | | | | | | | | | | |  | |
|  |  |  |  |  |  |  |  |  |  |  |  |  |  |  |  |  |  |  |  |  |  |
|  |  |  |  |  |  |  |  |  |  |  |  |  |  |  |  |  |  |  |  |  |  |
|  |  |  |  |  |  |  |  |  |  |  |  |  |  |  |  |  |  |  |  |  |  |
|  |  |  |  |  |  |  |  |  |  |  |  |  |  |  |  |  |  |  |  |  |  |
|  |  |  |  |  |  |  |  |  |  |  |  |  |  |  |  |  |  |  |  |  |  |
|  |  |  |  |  |  |  |  |  |  |  |  |  |  |  |  |  |  |  |  |  |  |
|  |  |  |  |  |  |  |  |  |  |  |  |  |  |  |  |  |  |  |  |  |  |
|  |  |  |  |  |  |  |  |  |  |  |  |  |  |  |  |  |  |  |  |  |  |
| Formatted Reports\\Coding Summary by File Formatted Report | | | | | | | | | Page 14 of 145 | | | | | | | | | | | |  |
| 8/9/2021 8:08 AM | | | | | | | | | | | | | | | | | | | | |  |
|  | **Classification** |  | **Aggregate** |  | **Coverage** |  | **Number Of Coding References** |  | | **Reference Number** | |  | | **Coded By Initials** | |  | | **Modified On** | |  | |
|  | I: Is there anyone whose relative came but later never came?  P3: During the first visit, there was my husband, my other family members. The second visit, I was with my husband until the last visit.  I: So he was available on all the visits?  P3: he was not present once.  I: He was present…  P3: For the other family members, they were just coming to greet the counselor and they were going to do other things.  I: Fine. Number 2?  P2: During the first visit, they [other family members] came to confirm because people were saying that she was coming to give me ARVs. | | | | | | | | | | | | | | | | | | |  | |
|  |  |  |  |  |  |  |  |  |  |  |  |  |  |  |  |  |  |  |  |  |  |
|  |  | | | | | | | | | | | | | | | | | | |  | |
|  |  | | | | | | | | | | | | | | | | | | |  | |
|  |  | | | | | | | | | | | | | | | | | | |  | |
|  | | | | | | | | | | 3 |  | | MTK | |  | | 8/8/2021 8:11 PM | |  | |  |
|  | I: Others?  P: I was with relatives because I am not with my husband. | | | | | | | | | | | | | | | | | | |  | |
|  |  | | | | | | | | | | | | | | | | | | |  | |
|  | **Codes\\Participation of others\Partners** | | | | | | | | | | | | | | | | | | |  | |
|  |  |  | No |  | 0.0477 |  | 7 |  | | | | | | | | | | | |  |  |
|  |  |  |  |  |  |  |  |  | | | | | | | | | | | | | |
|  | | | | | | | | | | 1 |  | | MTK | |  | | 8/8/2021 6:55 PM | |  | |  |
|  | I: Number 6?  P6: I was only with my husband for the five visits | | | | | | | | | | | | | | | | | | |  | |
|  |  | | | | | | | | | | | | | | | | | | |  | |
|  | | | | | | | | | | 2 |  | | MTK | |  | | 8/8/2021 8:10 PM | |  | |  |
|  | Note taker: I have one question; when you were asked about who was present during the counseling session, you mentioned your partners, mothers in laws… and you did mention that they were present only for one day or two days… I would like to understand why your husband was not available during the other visits? What was happening?  P10: my husband was present only twice but the other days he was busy with work. You know it is difficult to give excuses often. | | | | | | | | | | | | | | | | | | |  | |
|  |  | | | | | | | | | | | | | | | | | | |  | |
|  | | | | | | | | | | 3 |  | | MTK | |  | | 8/8/2021 8:10 PM | |  | |  |
|  | I: For number 10, the husband was busy at work.  P9: My husband was not present once because he was busy applying fertilizer in the garden and she came late.  P14: he was not present once because she found that we went to the garden because of the rains. So someone came to call us and he just sent me to go for counseling because he was busy. | | | | | | | | | | | | | | | | | | |  | |
|  |  | | | | | | | | | | | | | | | | | | |  | |
|  | | | | | | | | | | 4 |  | | MTK | |  | | 8/8/2021 8:10 PM | |  | |  |
|  | I: Others?  P3: as for me, he was just going out for a walk. He knew that she was coming for counseling but when she came she was finding that he was not there. For the three days he was present but he was absent the rest of the sessions.  P1: my husband is mobile. He was present twice. | | | | | | | | | | | | | | | | | | |  | |
|  |  | | | | | | | | | | | | | | | | | | |  | |
|  | | | | | | | | | | 5 |  | | MTK | |  | | 8/8/2021 8:10 PM | |  | |  |
|  | Note taker: When you say “he is mobile” you mean he does mobile work?  P1: He just moves aimlessly because he does business in the morning.  I: So he was gone for business?  P1: No he was just walking around. | | | | | | | | | | | | | | | | | | |  | |
|  |  | | | | | | | | | | | | | | | | | | |  | |
|  | | | | | | | | | | 6 |  | | MTK | |  | | 8/8/2021 8:11 PM | |  | |  |
|  | Note taker: I want to understand because we might think he was mobile while…  P1: When he came back, I was explaining to him whatever we discussed…  Note taker: But during the other days he was going for business?  P1: Yes. | | | | | | | | | | | | | | | | | | |  | |
|  |  | | | | | | | | | | | | | | | | | | |  | |
| Formatted Reports\\Coding Summary by File Formatted Report | | | | | | | | | Page 15 of 145 | | | | | | | | | | | |  |
| 8/9/2021 8:08 AM | | | | | | | | | | | | | | | | | | | | |  |
|  | **Classification** |  | **Aggregate** |  | **Coverage** |  | **Number Of Coding References** |  | | **Reference Number** | |  | | **Coded By Initials** | |  | | **Modified On** | |  | |
|  |  |  |  |  |  |  |  |  |  |  |  |  |  |  |  |  |  |  |  |  |  |
|  | | | | | | | | | | 7 |  | | MTK | |  | | 8/8/2021 8:11 PM | |  | |  |
|  | I: What about those of you with husbands?  P: mine was not present during all the sessions but when he came back, I was able to tell him and he was implementing it. | | | | | | | | | | | | | | | | | | |  | |
|  |  | | | | | | | | | | | | | | | | | | |  | |
|  | **Codes\\Participation of others\Support** | | | | | | | | | | | | | | | | | | |  | |
|  |  |  | No |  | 0.0237 |  | 3 |  | | | | | | | | | | | |  |  |
|  |  |  |  |  |  |  |  |  | | | | | | | | | | | | | |
|  | | | | | | | | | | 1 |  | | MTK | |  | | 8/8/2021 6:36 PM | |  | |  |
|  | I: Ladies, if you say this counseling has left marks on us. What is the mark that you can point at? Number 6?  P6: I had nothing when my child was born and I did not have any clothes but after the birth of the child, my friends brought me clothes and assorted items for the child. | | | | | | | | | | | | | | | | | | |  | |
|  |  | | | | | | | | | | | | | | | | | | |  | |
|  | | | | | | | | | | 2 |  | | MTK | |  | | 8/8/2021 7:12 PM | |  | |  |
|  | I: Or why was it important to have relatives around when you were receiving the counseling intervention? [Sound of children playing in the background]  P7: It was important because the work that I was doing at home was beyond my capacity as a pregnant woman and the inclusion of my mother in law helped a lot because that helped me to be relieved from hard work. Even my father in law admitted that I don’t need to work hard after the counseling. | | | | | | | | | | | | | | | | | | |  | |
|  |  | | | | | | | | | | | | | | | | | | |  | |
|  | | | | | | | | | | 3 |  | | MTK | |  | | 8/8/2021 7:13 PM | |  | |  |
|  | I: What was his intervention?  P7: When he came, he said that “this one should not take part in this work because she is close to delivery period.” | | | | | | | | | | | | | | | | | | |  | |
|  |  | | | | | | | | | | | | | | | | | | |  | |
|  | **Codes\\Recommendations\General** | | | | | | | | | | | | | | | | | | |  | |
|  |  |  | No |  | 0.1140 |  | 11 |  | | | | | | | | | | | |  |  |
|  |  |  |  |  |  |  |  |  | | | | | | | | | | | | | |
|  | | | | | | | | | | 1 |  | | MTK | |  | | 8/8/2021 6:28 PM | |  | |  |
|  | I: So it has helped you to deal with depression and also to help other people deal with depression. What do others say?  P: My comment is that it is good to deal with depression and we need to help our friends to deal with depression the way we were helped to deal with depression and this counseling strategy should not stop. | | | | | | | | | | | | | | | | | | |  | |
|  |  | | | | | | | | | | | | | | | | | | |  | |
|  | | | | | | | | | | 2 |  | | MTK | |  | | 8/8/2021 8:06 PM | |  | |  |
|  | I: If the government may decide to roll out this special counseling intervention to pregnant women, you as pioneers of this program, what would you say? Say anything you want… Are we together?  All: Yes!  P12: it can be good that the government should consider rolling out this counseling intervention so that future pregnant women should live happy lives and be able to give birth to healthy babies. | | | | | | | | | | | | | | | | | | |  | |
|  |  | | | | | | | | | | | | | | | | | | |  | |
|  | | | | | | | | | | 3 |  | | MTK | |  | | 8/8/2021 8:06 PM | |  | |  |
|  | I: What do others say?  Note taker: What are you going to tell the government?  P1: The government should consider planning to start providing this counseling intervention when the woman is four to five months so that she should be visited several times. For example, I came here during my sixth month So it is better to start earlier. | | | | | | | | | | | | | | | | | | |  | |
|  |  | | | | | | | | | | | | | | | | | | |  | |
|  | | | | | | | | | | 4 |  | | MTK | |  | | 8/8/2021 8:07 PM | |  | |  |
|  | I: That is number 1. What do others say?  P: I can tell the government to continue with this counseling intervention so that the future mothers should also benefit. | | | | | | | | | | | | | | | | | | |  | |
|  |  | | | | | | | | | | | | | | | | | | |  | |
| Formatted Reports\\Coding Summary by File Formatted Report | | | | | | | | | Page 16 of 145 | | | | | | | | | | | |  |
| 8/9/2021 8:08 AM | | | | | | | | | | | | | | | | | | | | |  |
|  | **Classification** |  | **Aggregate** |  | **Coverage** |  | **Number Of Coding References** |  | | **Reference Number** | |  | | **Coded By Initials** | |  | | **Modified On** | |  | |
|  |  |  |  |  |  |  |  |  |  |  |  |  |  |  |  |  |  |  |  |  |  |
|  | | | | | | | | | | 5 |  | | MTK | |  | | 8/8/2021 8:07 PM | |  | |  |
|  | I: Others?  The government needs to continue with this program… [Not audible due to noise of children hissing in the background]… We are worried that “am I going to make it to deliver the child? So they need to be supported.  I: Others?  P: This counseling should continue because some pregnant women are not married and beside the counseling sessions, the government should also provide financial support so that they can be able to buy basic needs. | | | | | | | | | | | | | | | | | | |  | |
|  |  | | | | | | | | | | | | | | | | | | |  | |
|  | | | | | | | | | | 6 |  | | MTK | |  | | 8/8/2021 8:08 PM | |  | |  |
|  | I: Counseling and financial support. Others, what do you say?  P3: This counseling should continue and as she has said, the government should be able to give us something so that we should be motivated.  I: What support?  P: To say the truth, the support is financial support that is required because they cannot say that I should be feeding the child when I have no food. Nowadays we need to buy everything and we need to have money to buy. | | | | | | | | | | | | | | | | | | |  | |
|  |  | | | | | | | | | | | | | | | | | | |  | |
|  | | | | | | | | | | 7 |  | | MTK | |  | | 8/8/2021 8:08 PM | |  | |  |
|  | I: Number 9?  P9: The government needs to go ahead with implementation of the counseling intervention but not in terms of food because food is locally available. It is during the rainy season that many people were struggling to find food but nowadays there is plenty of soya beans. Free things are not sustainable but we should just encourage the government to continue with the implementation of the intervention. | | | | | | | | | | | | | | | | | | |  | |
|  |  | | | | | | | | | | | | | | | | | | |  | |
|  | | | | | | | | | | 8 |  | | MTK | |  | | 8/8/2021 8:09 PM | |  | |  |
|  | I: Number 15?  P15: I can say that this counseling intervention because most of the men are abusive because they impregnate the woman but are not responsible enough to take care of the woman. In this case, counseling should continue so that men should be educated on how they can take care of their families.  I: Number 11?  P11: We are encouraging the government to continue with the counseling so that other pregnant women should be helped. | | | | | | | | | | | | | | | | | | |  | |
|  |  | | | | | | | | | | | | | | | | | | |  | |
|  | | | | | | | | | | 9 |  | | MTK | |  | | 8/8/2021 8:09 PM | |  | |  |
|  | P10: The government should continue with the counseling intervention to pregnant women because more women continue to become pregnant and there is need to know the importance of resting for pregnant women. | | | | | | | | | | | | | | | | | | |  | |
|  |  | | | | | | | | | | | | | | | | | | |  | |
|  | | | | | | | | | | 10 |  | | MTK | |  | | 8/8/2021 8:12 PM | |  | |  |
|  | I: In other words, for you to receive the intervention at your home, it was the counselor who was traveling but in this case, you are the one to travel… what do you think?  P14: Transport is a challenge.  P: Unless it is close to home, we can walk because we want to access the counseling service. Also, people have bicycles in the village and if you have good friends, they can borrow you a bicycle and you can go to access the service by bicycle…  Choral: Cross talk [Not audible] | | | | | | | | | | | | | | | | | | |  | |
|  |  | | | | | | | | | | | | | | | | | | |  | |
|  | | | | | | | | | | 11 |  | | MTK | |  | | 8/8/2021 8:13 PM | |  | |  |
|  | I: Why don’t you vote?  All: Yes!  I: For those who say that counseling should be provided at home raise your hands…  All: [Participants raise hands]…  I: 1, 2, 3, 4, 5, 6, 7, 8, 9, 10, 11, 12… And those who say the counseling should be provided at some place?  P14: We are four.  All: [Laughter]… | | | | | | | | | | | | | | | | | | |  | |
|  |  |  |  |  |  |  |  |  |  |  |  |  |  |  |  |  |  |  |  |  |  |
|  |  | | | | | | | | | | | | | | | | | | |  | |
|  |  |  |  |  |  |  |  |  |  |  |  |  |  |  |  |  |  |  |  |  |  |
| Formatted Reports\\Coding Summary by File Formatted Report | | | | | | | | | Page 17 of 145 | | | | | | | | | | | |  |
| 8/9/2021 8:08 AM | | | | | | | | | | | | | | | | | | | | |  |
|  | **Classification** |  | **Aggregate** |  | **Coverage** |  | **Number Of Coding References** |  | | **Reference Number** | |  | | **Coded By Initials** | |  | | **Modified On** | |  | |
|  | **Codes\\Recommendations\Intervention process** | | | | | | | | | | | | | | | | | | |  | |
|  |  |  | No |  | 0.0142 |  | 1 |  | | | | | | | | | | | |  |  |
|  |  |  |  |  |  |  |  |  | | | | | | | | | | | | | |
|  | | | | | | | | | | 1 |  | | MTK | |  | | 8/8/2021 7:25 PM | |  | |  |
|  | P9: When there was something sensitive, the counselor was asking the other people to go out so that they should not hear what was being discussed between you and the counselor. So the counseling should be done at home but when there are sensitive issues, the other people should not be present they should give room for private discussion between you and the counselor because if they are present, you cannot be free. The issue of food is general but there are some confidential issues that don’t require big audience. | | | | | | | | | | | | | | | | | | |  | |
|  |  | | | | | | | | | | | | | | | | | | |  | |
|  | **Codes\\Recommendations\Period** | | | | | | | | | | | | | | | | | | |  | |
|  |  |  | No |  | 0.0144 |  | 1 |  | | | | | | | | | | | |  |  |
|  |  |  |  |  |  |  |  |  | | | | | | | | | | | | | |
|  | | | | | | | | | | 1 |  | | MTK | |  | | 8/8/2021 8:06 PM | |  | |  |
|  | I: What do others say?  Note taker: What are you going to tell the government?  P1: The government should consider planning to start providing this counseling intervention when the woman is four to five months pregnant so that she should be visited several times. | | | | | | | | | | | | | | | | | | |  | |
|  |  | | | | | | | | | | | | | | | | | | |  | |
|  | **Codes\\Recommendations\Place of delivery** | | | | | | | | | | | | | | | | | | |  | |
|  |  |  | No |  | 0.0863 |  | 2 |  | | | | | | | | | | | |  |  |
|  |  |  |  |  |  |  |  |  | | | | | | | | | | | | | |
|  | | | | | | | | | | 1 |  | | MTK | |  | | 8/8/2021 7:20 PM | |  | |  |
|  | I: So we have received this counseling as individuals, then it impacts to the family and then to the entire community… What is your opinion of accessing the counseling in the community as you did and not at the clinic?  P4: It would be better if this counseling would be provided at an independent place rather than a home because there are different disturbances at home. “someone wants you, something is happening..” as a result, the participant comes out often but if we could go somewhere, we could complete the whole session without any disturbance.  I: Thank you very much. So let us respond to this question in our own perspective. We should not respond based on someone’s experiences… Number 6?  P6: This counseling should be done at home. This time people were talking a lot because this is a new thing but once it is established, people will be aware of it.  I: Why do you want it to take place at home?  P6: Because in a group like this one, we may not take something seriously but if we are the two of us, there is more concentration on what we are discussing.  I: So, in this context, you should consider that you are receiving individual counseling at home. Number 10?  P10: it is better to provide the counseling here at the clinic because at home you cannot be free. You may be in conflict with your partner and if you talk about it during the counseling, your mother in law may not be happy. In addition to that, some mothers in law are not happy when you are having good nutrition at your home.  I: Thank you. What do others say? | | | | | | | | | | | | | | | | | | |  | |
|  |  |  |  |  |  |  |  |  |  |  |  |  |  |  |  |  |  |  |  |  |  |
|  |  |  |  |  |  |  |  |  |  |  |  |  |  |  |  |  |  |  |  |  |  |
|  |  |  |  |  |  |  |  |  |  |  |  |  |  |  |  |  |  |  |  |  |  |
|  |  | | | | | | | | | | | | | | | | | | |  | |
|  |  |  |  |  |  |  |  |  |  |  |  |  |  |  |  |  |  |  |  |  |  |
| Formatted Reports\\Coding Summary by File Formatted Report | | | | | | | | | Page 18 of 145 | | | | | | | | | | | |  |
| 8/9/2021 8:08 AM | | | | | | | | | | | | | | | | | | | | |  |
|  | **Classification** |  | **Aggregate** |  | **Coverage** |  | **Number Of Coding References** |  | | **Reference Number** | |  | | **Coded By Initials** | |  | | **Modified On** | |  | |
|  |  |  |  |  |  |  |  |  |  |  |  |  |  |  |  |  |  |  |  |  |  |
|  | | | | | | | | | | 2 |  | | MTK | |  | | 8/8/2021 7:20 PM | |  | |  |
|  | P8: It is better to have the counseling session at home so that your mother in law should hear by herself whatever is happening.  I: Tell us more…  P8: If she does not give you food, she should know the importance of giving you food.  P: I agree to this one. It is important that the counseling sessions should be done at home because you can invite the neighbors and if there are issues, they should be discussed there and the counselor should know what is happening.  P9: When there was something sensitive, the counselor was asking the other people to go out so that they should not hear what was being discussed between you and the counselor. So the counseling should be done at home but when there are sensitive issues, the other people should not be present they should give room for private discussion between you and the counselor because if they are present, you cannot be free. The issue of food is general but there are some confidential issues that don’t require big audience. | | | | | | | | | | | | | | | | | | |  | |
|  |  |  |  |  |  |  |  |  |  |  |  |  |  |  |  |  |  |  |  |  |  |
|  |  | | | | | | | | | | | | | | | | | | |  | |
|  | **Codes\\THPP providers** | | | | | | | | | | | | | | | | | | |  | |
|  |  |  | No |  | 0.0169 |  | 2 |  | | | | | | | | | | | |  |  |
|  |  |  |  |  |  |  |  |  | | | | | | | | | | | | | |
|  | | | | | | | | | | 1 |  | | MTK | |  | | 8/8/2021 6:25 PM | |  | |  |
|  | I: Behind there, do you have something to say?  P: The counselors helped us to deal with the stress that we had. | | | | | | | | | | | | | | | | | | |  | |
|  |  | | | | | | | | | | | | | | | | | | |  | |
|  | | | | | | | | | | 2 |  | | MTK | |  | | 8/8/2021 6:49 PM | |  | |  |
|  | I: Are there additions?  P: I did not have means to start antenatal clinic…  I: You did not have means?  P: Yes, I did not have means  I: Can you clarify?  P: She mean that she did not have a partner the time when she came to start antenatal clinic…  I: [Laughter]… Now I understand… can you tell me what happened?  P: What happened was that the man did not marry me and when the counselor came, she told me that I should come here to start antenatal clinic and that was when I was screened for depression. | | | | | | | | | | | | | | | | | | |  | |
|  |  |  |  |  |  |  |  |  |  |  |  |  |  |  |  |  |  |  |  |  |  |
|  |  | | | | | | | | | | | | | | | | | | |  | |
|  | **Files\\IDIs\\FGD with mothers** | | | | | | | | | | | | | | | | | | |  | |
|  | **Code** | | | | | | | | | | | | | | | | | | |  | |
|  | **Codes\\Intervention experience** | | | | | | | | | | | | | | | | | | |  | |
|  |  |  | No |  | 0.1079 |  | 8 |  | | | | | | | | | | | |  |  |
|  |  |  |  |  |  |  |  |  | | | | | | | | | | | | | |
|  | | | | | | | | | | 1 |  | | MTK | |  | | 8/9/2021 8:00 AM | |  | |  |
|  | P8: I was happy when she was coming to provide counseling because we were learning on how we can deal with stress.  I: What was happening during the counseling? Number 4…  P4: There were lessons on replacing unhelpful behavior with helpful behavior. Like in my case, when I was pregnant, I was thinking… “Should I abort the pregnancy?” So I learned that it was not a helpful way of thinking.  I: What helped you to change your thoughts from unhelpful ones, of wanting to eliminate the pregnancy to have the helpful thoughts that you should keep the baby?  P4: It was because of the counseling.  I: What about counseling?  P4: The sessions she took me through, the relationship with others people around, the relatives, my husband. | | | | | | | | | | | | | | | | | | |  | |
|  |  |  |  |  |  |  |  |  |  |  |  |  |  |  |  |  |  |  |  |  |  |
|  |  | | | | | | | | | | | | | | | | | | |  | |
|  |  |  |  |  |  |  |  |  |  |  |  |  |  |  |  |  |  |  |  |  |  |
| Formatted Reports\\Coding Summary by File Formatted Report | | | | | | | | | Page 19 of 145 | | | | | | | | | | | |  |
| 8/9/2021 8:08 AM | | | | | | | | | | | | | | | | | | | | |  |
|  | **Classification** |  | **Aggregate** |  | **Coverage** |  | **Number Of Coding References** |  | | **Reference Number** | |  | | **Coded By Initials** | |  | | **Modified On** | |  | |
|  |  |  |  |  |  |  |  |  |  |  |  |  |  |  |  |  |  |  |  |  |  |
|  | | | | | | | | | | 2 |  | | MTK | |  | | 8/9/2021 8:00 AM | |  | |  |
|  | I: So how are your thoughts currently?  P: I don’t have any problem and I do everything as before.  I: Thank you very much. I saw a hand from number 7…  P7: I had similar thoughts that have already been said.  I: Thank you. Are there others with different experiences from what others have said?  P: Relationship with others…  Note taker: You should raise your voice because you are behind…  P: When I learned about the relationship between the mother and the child and when I utilized it, I saw that my child is growing healthy. The thoughts that I had that I am poor and I cannot do anything, I found that they were not true because I am capable of doing things. | | | | | | | | | | | | | | | | | | |  | |
|  |  |  |  |  |  |  |  |  |  |  |  |  |  |  |  |  |  |  |  |  |  |
|  |  | | | | | | | | | | | | | | | | | | |  | |
|  | | | | | | | | | | 3 |  | | MTK | |  | | 8/9/2021 8:00 AM | |  | |  |
|  | I: Behind there, do you have something to say?  P: The counselors helped us to deal with the stress that we had. | | | | | | | | | | | | | | | | | | |  | |
|  |  | | | | | | | | | | | | | | | | | | |  | |
|  | | | | | | | | | | 4 |  | | MTK | |  | | 8/9/2021 8:00 AM | |  | |  |
|  | I: What do others say?  P: I was so depressed when I was pregnant on this child. Everyone in my area knew that I was depressed but now they see that I am leaving a healthy life and they ask me why? I tell them that it is the counseling that I receive which has helped me. I tell them that depression makes a pregnant woman feel like she is not worth living and she does not live a healthy life. I experienced the same but now I am a changed person and you also need to change. If you are happy, you need to go beyond where you are now. | | | | | | | | | | | | | | | | | | |  | |
|  |  | | | | | | | | | | | | | | | | | | |  | |
|  | | | | | | | | | | 5 |  | | MTK | |  | | 8/9/2021 8:00 AM | |  | |  |
|  | I: Okay. What do others say? How has counseling helped you?  P1: Counseling has helped us and this is the best intervention ever. We are changed people. We moved from where we were to another level.  I: Can you give an example of what changed?  P1: An example is that we were living in isolation without visiting friends but now we are able to visit friends and if you don’t have something, you ask your friend “I don’t have this, can you share me? Or I need help” We are very thankful. | | | | | | | | | | | | | | | | | | |  | |
|  |  | | | | | | | | | | | | | | | | | | |  | |
|  | | | | | | | | | | 6 |  | | MTK | |  | | 8/9/2021 8:00 AM | |  | |  |
|  | I: Number 10?  P10: I stay near her and she knows what I have gone through and if there is someone who has suffered from problems, I am the one. Thoughts of aborting the pregnancy were in me. I was not talking with my mother in law. We could hardly visit each other nor support each other. When I received this counseling, I said that I needed to visit my mother in law. We currently visit each other and we are in good terms. | | | | | | | | | | | | | | | | | | |  | |
|  |  | | | | | | | | | | | | | | | | | | |  | |
|  | | | | | | | | | | 7 |  | | MTK | |  | | 8/9/2021 8:00 AM | |  | |  |
|  | I: Ladies, if you say this counseling has left marks on us. What is the mark that you can point at? Number 6?  P6: I had nothing when my child was born and I did not have any clothes but after the birth of the child, my friends brought me clothes and assorted items for the child.  I: What is the difference with how things were before?  P6: I am able to differentiate because the other children that I have, no one gave me clothes but this one, I received clothes from my friends. | | | | | | | | | | | | | | | | | | |  | |
|  |  | | | | | | | | | | | | | | | | | | |  | |
|  | | | | | | | | | | 8 |  | | MTK | |  | | 8/9/2021 8:00 AM | |  | |  |
|  | I: How did this affect you considering the counseling that you were receiving?  P: It really had an impact on me because my relationship with my blood sister was very sour but since the counselor intervened, our relationship is very good and strong up to now. For my grandmother, she has bad behavior because the counselor tried her best but she chose not to take part. However, I learnt that it is very important to be in good relationship with my relatives. | | | | | | | | | | | | | | | | | | |  | |
|  |  | | | | | | | | | | | | | | | | | | |  | |
|  |  |  |  |  |  |  |  |  |  |  |  |  |  |  |  |  |  |  |  |  |  |
| Formatted Reports\\Coding Summary by File Formatted Report | | | | | | | | | Page 20 of 145 | | | | | | | | | | | |  |
| 8/9/2021 8:08 AM | | | | | | | | | | | | | | | | | | | | |  |
|  | **Classification** |  | **Aggregate** |  | **Coverage** |  | **Number Of Coding References** |  | | **Reference Number** | |  | | **Coded By Initials** | |  | | **Modified On** | |  | |
|  | **Codes\\Intervention experience\Aspects** | | | | | | | | | | | | | | | | | | |  | |
|  |  |  | No |  | 0.2046 |  | 5 |  | | | | | | | | | | | |  |  |
|  |  |  |  |  |  |  |  |  | | | | | | | | | | | | | |
|  | | | | | | | | | | 1 |  | | MTK | |  | | 8/9/2021 8:00 AM | |  | |  |
|  | I: Yes, number 8 you raised your hand…  P8: I was happy when she was coming to provide counseling because we were learning on how we can deal with stress.  I: What was happening during the counseling? Number 4…  P4: There were lessons on replacing unhelpful behavior with helpful behavior. Like in my case, when I was pregnant, I was thinking… “Should I remove the pregnancy?” So I learned that it was not a helpful way of thinking.  I: What helped you to change your thoughts from unhelpful ones whereby you wanted to eliminate the pregnancy to have the helpful thoughts that you should maintain it?  P4: It was because of the counseling.  I: What about counseling?  P4: The sessions, the relationship with other people, the relatives, my husband.  I: That is number 4, she has expressed her side. Remember we are not answering for each other. What do others say?  P10: We learned many things for example the issue of nutrition. Sometimes we were lazy to eat saying that “I will just wait for lunch.” When the counselor came, she encouraged us to eat in the morning during breakfast, before lunch, after lunch and supper. She taught us many things.  I: Others replaced unhelpful thoughts with helpful thoughts, others learned about nutrition… what do others say?  P7: I learnt about the health of the child on exclusive breastfeeding. During pregnancy, since I was impregnated by a certain boy who refused responsibility, I thought that I should just throw myself in the river but when we came here, we were told that the counselor would be coming home to provide counseling. When the counselor was coming for counseling sessions, I saw that my thoughts were not helpful and I noted that when the child starts eating other foods, I need to feed her in the morning, during lunch and in the evening just as my friend has said. I learned something.  I: Number 1?  P1: I learned about being in good relationship with my neighbors and letting my family help me to take care of the child. I did not know this before but I learnt a lot from this study. I learned that you cannot take care of the child single handedly but with the support of the family and others, the raising of the child can be made easy.  I: Yes, number 9?  P9: I learned that we should relate well with our friends and that we should learn to be in groups so that we can be avoiding stress from the family. We also learnt that we should love our friends the way we love ourselves. This is what I have learned from the study.  I: Number 2?  P2: I am happy to learn about these things because we learnt that when we don’t have something, we should go to our friends and ask from them and if we have stress, we should be able to seek guidance from neighbors whom we trust.  P14: Number 14…  I: Yes number 14…  P14: I have learned that if you have friends, you live a happy life and many people like your child and taking care of the child cannot be a challenge is you have friends. For instance I was worried that “am I going to make it to take care of the child?” With this program, I have seen that I will be able to take care of the child.  I: Yes, number…?  P12: Number 12… This program has helped me to live positively because at first, I was thinking that “am I going to take care of the child?” My thoughts were to eliminate the pregnancy or to hang myself but after being counselled, I saw that what I was thinking was not helpful. I reached the extent that I delivered a live baby and she followed me thereafter.  I: Thank you very much…  P6: Number 6… I had no time to rest but when the counselor came, she told me that “you need to have time to rest after working.” I found it helpful because when I was pregnant, I used to overwork myself. Thank you.  P3: I concur with my friend. I was also not resting because I was staying at somebody’s home. After delivery, I had a problem of continuous menses for three consecutive month and when I came here at the hospital, I was given treatment. I took the medications for one week but there was no change and I came back here. They changed drugs and I saw improvement. When the counselor was coming, I was encouraged because a lot was said at home but I did not care about them because I knew that it is God who cares about me because he is the one who created me .  I: Meaning that you did not care about what people were saying?  P3: The one who was present during counseling was talking a lot about me with people. When the disease was continuing, there were a lot of issues that took place.  I: So how are your thoughts currently?  P: I don’t have any problem and I do everything as before.  I: Thank you very much. I saw a hand from number 7…  P7: I had similar thoughts that have already been said.  I: Thank you. Are there others with different experiences from what others have said?  P: Relationship with others…  Note taker: You should raise your voice because you are behind…  P: When I learned about the relationship between the mother and the child and when I utilized it, I saw that my child is growing healthy. The thoughts that I had that I am poor and I cannot do anything, I found that they were not true because I am capable of doing things.  I: Others? | | | | | | | | | | | | | | | | | | |  | |
|  |  |  |  |  |  |  |  |  |  |  |  |  |  |  |  |  |  |  |  |  |  |
|  |  |  |  |  |  |  |  |  |  |  |  |  |  |  |  |  |  |  |  |  |  |
|  |  |  |  |  |  |  |  |  |  |  |  |  |  |  |  |  |  |  |  |  |  |
|  |  |  |  |  |  |  |  |  |  |  |  |  |  |  |  |  |  |  |  |  |  |
|  |  |  |  |  |  |  |  |  |  |  |  |  |  |  |  |  |  |  |  |  |  |
|  |  |  |  |  |  |  |  |  |  |  |  |  |  |  |  |  |  |  |  |  |  |
|  |  |  |  |  |  |  |  |  |  |  |  |  |  |  |  |  |  |  |  |  |  |
|  |  |  |  |  |  |  |  |  |  |  |  |  |  |  |  |  |  |  |  |  |  |
| Formatted Reports\\Coding Summary by File Formatted Report | | | | | | | | | Page 21 of 145 | | | | | | | | | | | |  |
| 8/9/2021 8:08 AM | | | | | | | | | | | | | | | | | | | | |  |
|  | **Classification** |  | **Aggregate** |  | **Coverage** |  | **Number Of Coding References** |  | | **Reference Number** | |  | | **Coded By Initials** | |  | | **Modified On** | |  | |
|  | P: In addition to what she has said, when you love your child, s/he knows that you are showing her/him love. We need to talk to the children they hear.  I: Is that what you learned?  All: Yes!  P: Even during pregnancy, you can tell her/him that “my child I love you/” S/he hears you.  All: [Laughter]…  P9: Singing spiritual songs to the child…  I: Number 9?  P9: Singing spiritual songs to the child makes her/him feel happy.  I: Behind there, do you have something to say?  P: The counselors helped us to deal with the stress that we had. | | | | | | | | | | | | | | | | | | |  | |
|  |  |  |  |  |  |  |  |  |  |  |  |  |  |  |  |  |  |  |  |  |  |
|  |  | | | | | | | | | | | | | | | | | | |  | |
|  | | | | | | | | | | 2 |  | | MTK | |  | | 8/9/2021 8:00 AM | |  | |  |
|  | I: If you may tell someone that “counseling helped me in this way…” what can you tell the person.. how has counseling helped you?  P14: It has helped me because most of the times I was not happy but when the counselor started coming, my stress was addressed and if someone can be depressed, I can tell her that “that will not help you. I had depression but it was addressed. I feel better.” I can tell her the way the counselor was teaching me. | | | | | | | | | | | | | | | | | | |  | |
|  |  | | | | | | | | | | | | | | | | | | |  | |
|  | | | | | | | | | | 3 |  | | MTK | |  | | 8/9/2021 8:00 AM | |  | |  |
|  | I: Others, what do you say…? You mentioned a lot of things… there was an issue of stress, nutrition… taking care of the child…  P7: In terms of taking care of the child, the child is supposed to eat frequently. There are other mothers who were asking me why the counselor was visiting me only and I was just telling them what I was learning. | | | | | | | | | | | | | | | | | | |  | |
|  |  | | | | | | | | | | | | | | | | | | |  | |
|  | | | | | | | | | | 4 |  | | MTK | |  | | 8/9/2021 8:00 AM | |  | |  |
|  | I: Are there some additions? Why was it important to include relatives during the counseling sessions?  P14: It was important because we were learning about nutrition for example. So the involvement of the relatives was helpful because there was no way you could stay up to lunch hour without any food. The relatives would take part to give you some food because they would say “what we learned was that you need to eat frequently.” | | | | | | | | | | | | | | | | | | |  | |
|  |  | | | | | | | | | | | | | | | | | | |  | |
|  | | | | | | | | | | 5 |  | | MTK | |  | | 8/9/2021 8:00 AM | |  | |  |
|  | I: Number 16?  P16: [Part of the discussion not audible because of hissing sound of children in the background] The time to eat, time to rest and time to chat was to be adhered to. | | | | | | | | | | | | | | | | | | |  | |
|  |  | | | | | | | | | | | | | | | | | | |  | |
|  | **Codes\\Intervention experience\Facilitators** | | | | | | | | | | | | | | | | | | |  | |
|  |  |  | No |  | 0.0062 |  | 1 |  | | | | | | | | | | | |  |  |
|  |  |  |  |  |  |  |  |  | | | | | | | | | | | | | |
|  | | | | | | | | | | 1 |  | | MTK | |  | | 8/9/2021 8:00 AM | |  | |  |
|  | I: So what happened next?  P: I saw that most of them understood especially the one who was present during the counseling.  I: What happened for them to understand?  P: She was present during the first session with her husband. | | | | | | | | | | | | | | | | | | |  | |
|  |  | | | | | | | | | | | | | | | | | | |  | |
|  | **Codes\\Intervention experience\Influence on others** | | | | | | | | | | | | | | | | | | |  | |
|  |  |  | No |  | 0.0425 |  | 3 |  | | | | | | | | | | | |  |  |
|  |  |  |  |  |  |  |  |  | | | | | | | | | | | | | |
|  | | | | | | | | | | 1 |  | | MTK | |  | | 8/9/2021 8:00 AM | |  | |  |
|  | I: So it has helped you to deal with depression and also to help other people deal with depression. What do others say?  P: My comment is that it is good to deal with depression and we need to help our friends to deal with depression the way we were helped to deal with depression and this counseling strategy should not stop. | | | | | | | | | | | | | | | | | | |  | |
|  |  | | | | | | | | | | | | | | | | | | |  | |
|  |  |  |  |  |  |  |  |  |  |  |  |  |  |  |  |  |  |  |  |  |  |
| Formatted Reports\\Coding Summary by File Formatted Report | | | | | | | | | Page 22 of 145 | | | | | | | | | | | |  |
| 8/9/2021 8:08 AM | | | | | | | | | | | | | | | | | | | | |  |
|  | **Classification** |  | **Aggregate** |  | **Coverage** |  | **Number Of Coding References** |  | | **Reference Number** | |  | | **Coded By Initials** | |  | | **Modified On** | |  | |
|  |  |  |  |  |  |  |  |  |  |  |  |  |  |  |  |  |  |  |  |  |  |
|  | | | | | | | | | | 2 |  | | MTK | |  | | 8/9/2021 8:00 AM | |  | |  |
|  | I: What do others say?  P: I was so depressed when I was pregnant with this child. Everyone in my area knew that I was depressed but now they see that I am leaving a healthy life and they ask me why? I tell them that it is the counseling that I receive which has helped me. I tell them that depression makes a pregnant woman feel like she is not worth living and she does not live a healthy life. I experienced the same but now I am a changed person and you also need to change. If you are happy, you need to go beyond where you are now. | | | | | | | | | | | | | | | | | | |  | |
|  |  | | | | | | | | | | | | | | | | | | |  | |
|  | | | | | | | | | | 3 |  | | MTK | |  | | 8/9/2021 8:00 AM | |  | |  |
|  | I: So what would you tell other pregnant women about this counseling?  P14: If I can see a pregnant women who is depressed the way I was when I was pregnant, I can counsel her the way [Name of volunteer] counselled me so that she should be free from stress and live a healthy life and that when she delivers, she should take care of the child the way s/he is supposed to be taken care of.  I: that is number 14… Number 12?  P12: I had the same opinion.  P4: I can counsel the pregnant woman because I had the same experience. | | | | | | | | | | | | | | | | | | |  | |
|  |  |  |  |  |  |  |  |  |  |  |  |  |  |  |  |  |  |  |  |  |  |
|  |  | | | | | | | | | | | | | | | | | | |  | |
|  | **Codes\\Intervention experience\Likes** | | | | | | | | | | | | | | | | | | |  | |
|  |  |  | No |  | 0.0119 |  | 1 |  | | | | | | | | | | | |  |  |
|  |  |  |  |  |  |  |  |  | | | | | | | | | | | | | |
|  | | | | | | | | | | 1 |  | | MTK | |  | | 8/8/2021 5:59 PM | |  | |  |
|  | I: Yes, number 8 you raised your hand…  P8: I was happy when she was coming to provide counseling because we were learning on how we can deal with stress.  I: What was happening during the counseling? Number 4…  P4: There were lessons on replacing unhelpful behavior with helpful behavior. Like in my case, when I was pregnant, I was thinking… “Should I terminate the pregnancy?” So I learned that it was not a helpful way of thinking. | | | | | | | | | | | | | | | | | | |  | |
|  |  | | | | | | | | | | | | | | | | | | |  | |
|  | **Codes\\Intervention experience\Misconceptions** | | | | | | | | | | | | | | | | | | |  | |
|  |  |  | No |  | 0.0736 |  | 7 |  | | | | | | | | | | | |  |  |
|  |  |  |  |  |  |  |  |  | | | | | | | | | | | | | |
|  | | | | | | | | | | 1 |  | | MTK | |  | | 8/9/2021 8:00 AM | |  | |  |
|  | I: Fine. Number 2?  P2: During the first visit, they [other family members and neighbours] came to confirm because people were saying that she was coming to give me ARVs.  All: [Laughter]….  Choral responses: Not audible.  P: They were also saying that to me…  P2: Then they saw that what they were thinking was not what was happening. | | | | | | | | | | | | | | | | | | |  | |
|  |  | | | | | | | | | | | | | | | | | | |  | |
|  | | | | | | | | | | 2 |  | | MTK | |  | | 8/9/2021 8:00 AM | |  | |  |
|  | I: That reminds me; how did the people around you react to the counseling? So she has started “they wanted to see if there were really ARVs [Chuckles]… What do others say?  P14: That was what they were saying… | | | | | | | | | | | | | | | | | | |  | |
|  |  | | | | | | | | | | | | | | | | | | |  | |
|  | | | | | | | | | | 3 |  | | MTK | |  | | 8/9/2021 8:00 AM | |  | |  |
|  | P: I only stayed with them once.  P: They were saying that “they are satanic group of people. Remember she was staying in [Name of location], she must have done something… They want to pick her up.” | | | | | | | | | | | | | | | | | | |  | |
|  |  | | | | | | | | | | | | | | | | | | |  | |
|  | | | | | | | | | | 4 |  | | MTK | |  | | 8/9/2021 8:00 AM | |  | |  |
|  | I: So let us be on the same page here; at first they have said that “she is coming to provide ARVs,” or “they are satanic…” How did their reactions change overtime after the counseling? Number 2?  P2: I did not know that they are saying that I receive ARVs. Then a friend of mine came. She found [Name of volunteer] counseling me. After the counseling, she confessed that “I was wondering they were saying that you come here to provide ARVs but this is good counseling and I have also learned.” Other people started coming during the counseling sessions to sit in. It was shameful because it was the aunt of my husband who was fabricating that. | | | | | | | | | | | | | | | | | | |  | |
|  |  | | | | | | | | | | | | | | | | | | |  | |
| Formatted Reports\\Coding Summary by File Formatted Report | | | | | | | | | Page 23 of 145 | | | | | | | | | | | |  |
| 8/9/2021 8:08 AM | | | | | | | | | | | | | | | | | | | | |  |
|  | **Classification** |  | **Aggregate** |  | **Coverage** |  | **Number Of Coding References** |  | | **Reference Number** | |  | | **Coded By Initials** | |  | | **Modified On** | |  | |
|  |  |  |  |  |  |  |  |  |  |  |  |  |  |  |  |  |  |  |  |  |  |
|  | | | | | | | | | | 5 |  | | MTK | |  | | 8/9/2021 8:00 AM | |  | |  |
|  | I: Okay thank you very much.  P: My mother in law confessed when she realized that it was not what people were saying. “Sorry we thought you were satanic people because that is what people were saying but this is a good intervention.” | | | | | | | | | | | | | | | | | | |  | |
|  |  | | | | | | | | | | | | | | | | | | |  | |
|  | | | | | | | | | | 6 |  | | MTK | |  | | 8/9/2021 8:00 AM | |  | |  |
|  | I: Okay thank you very much. Others?  P3: I heard from my husband. When he came back from where he went he said “Mother of [Name of child], people are saying that the counselor comes to give us ARVs.” I called my neighbor because she is the one who has been present during the counseling and she said “I heard this sometime back that you take ARVs. I wanted you to hear it by yourselves.” I was trying to explain to them that “she comes for counseling and this is what we discuss…” but there are some people who do not understand much as there are others who understand…. | | | | | | | | | | | | | | | | | | |  | |
|  |  |  |  |  |  |  |  |  |  |  |  |  |  |  |  |  |  |  |  |  |  |
|  |  | | | | | | | | | | | | | | | | | | |  | |
|  | | | | | | | | | | 7 |  | | MTK | |  | | 8/9/2021 8:00 AM | |  | |  |
|  | P4: They were saying “people are saying she provides ARVs but then where are they?” They were surprised when they heard that the discussion is about how to take care of the child and they said “the issue of ARVs is not true. “We also need to have access to this counseling so that in future our children should be healthy.” | | | | | | | | | | | | | | | | | | |  | |
|  |  | | | | | | | | | | | | | | | | | | |  | |
|  |  |  |  |  |  |  |  |  |  |  |  |  |  |  |  |  |  |  |  |  |  |
|  |  |  |  |  |  |  |  |  |  |  |  |  |  |  |  |  |  |  |  |  |  |
|  |  |  |  |  |  |  |  |  |  |  |  |  |  |  |  |  |  |  |  |  |  |
|  |  |  |  |  |  |  |  |  |  |  |  |  |  |  |  |  |  |  |  |  |  |
|  |  |  |  |  |  |  |  |  |  |  |  |  |  |  |  |  |  |  |  |  |  |
|  |  |  |  |  |  |  |  |  |  |  |  |  |  |  |  |  |  |  |  |  |  |
| Formatted Reports\\Coding Summary by File Formatted Report | | | | | | | | | Page 24 of 145 | | | | | | | | | | | |  |
| 8/9/2021 8:08 AM | | | | | | | | | | | | | | | | | | | | |  |
|  | **Classification** |  | **Aggregate** |  | **Coverage** |  | **Number Of Coding References** |  | | **Reference Number** | |  | | **Coded By Initials** | |  | | **Modified On** | |  | |
|  | **Codes\\Intervention process\Delivery** | | | | | | | | | | | | | | | | | | |  | |
|  |  |  | No |  | 0.1627 |  | 2 |  | | | | | | | | | | | |  |  |
|  |  |  |  |  |  |  |  |  | | | | | | | | | | | | | |
|  | | | | | | | | | | 1 |  | | MTK | |  | | 8/9/2021 8:00 AM | |  | |  |
|  | P8: I was happy when she was coming to provide counseling because we were learning on how we can deal with stress.  I: What was happening during the counseling? Number 4…  P4: There were lessons on replacing unhelpful behavior with helpful behavior. Like in my case, when I was pregnant, I was thinking… “Should I terminate the pregnancy?”.  I: What helped you to change your thoughts from unhelpful ones whereby you wanted to eliminate the pregnancy to have the helpful thoughts that you should maintain it?  P4: It was because of the counseling.  P1: I learned about being in good relationship with my neighbors and letting my family help me to take care of the child. I did not know this before but I learnt a lot from this study. I learned that you cannot take care of the child single handedly but with the support of the family and others, the  I: Yes, number…?  P12: Number 12… This program has helped me to live positively because at first, I was thinking that “am I going to take care of the child?” My thoughts were to eliminate the pregnancy or to hang myself but after being counselled, I saw that what I was thinking was not helpful. I reached the extent that I delivered a live baby and she followed me thereafter.  I: Thank you very much…  P6: Number 6… I had no time to rest but when the counselor came, she told me that “you need to have time to rest after working.” I found it helpful because when I was pregnant, I used to overwork myself. Thank you.  P3: I concur with my friend. I was also not resting because I was staying at somebody’s home.  I: Thank you very much. I saw a hand from number 7…  P7: I had similar thoughts that have already been said.  I: Thank you. Are there others with different experiences from what others have said?  P: Relationship with others…  Note taker: You should raise your voice because you are behind…  P: When I learned about the relationship between the mother and the child and when I utilized it, I saw that my child is growing healthy. The thoughts that I had that I am poor and I cannot do anything, I found that they were not true because I am capable of doing things.  I: Others?  P: In addition to what she has said, when you love your child, s/he knows that you are showing her/him love. We need to talk to the  children they hear.  I: Is that what you learned?  All: Yes! | | | | | | | | | | | | | | | | | | |  | |
|  |  |  |  |  |  |  |  |  |  |  |  |  |  |  |  |  |  |  |  |  |  |
|  |  |  |  |  |  |  |  |  |  |  |  |  |  |  |  |  |  |  |  |  |  |
|  |  |  |  |  |  |  |  |  |  |  |  |  |  |  |  |  |  |  |  |  |  |
|  |  |  |  |  |  |  |  |  |  |  |  |  |  |  |  |  |  |  |  |  |  |
|  |  |  |  |  |  |  |  |  |  |  |  |  |  |  |  |  |  |  |  |  |  |
|  |  |  |  |  |  |  |  |  |  |  |  |  |  |  |  |  |  |  |  |  |  |
|  |  |  |  |  |  |  |  |  |  |  |  |  |  |  |  |  |  |  |  |  |  |
|  |  |  |  |  |  |  |  |  |  |  |  |  |  |  |  |  |  |  |  |  |  |
| Formatted Reports\\Coding Summary by File Formatted Report | | | | | | | | | Page 25 of 145 | | | | | | | | | | | |  |
| 8/9/2021 8:08 AM | | | | | | | | | | | | | | | | | | | | |  |
|  | **Classification** |  | **Aggregate** |  | **Coverage** |  | **Number Of Coding References** |  | | **Reference Number** | |  | | **Coded By Initials** | |  | | **Modified On** | |  | |
|  |  | | | | | | | | | | | | | | | | | | |  | |
|  |  | | | | | | | | | | | | | | | | | | |  | |
|  |  |  |  |  |  |  |  |  |  |  |  |  |  |  |  |  |  |  |  |  |  |
|  | **Codes\\Intervention process\Feelings** | | | | | | | | | | | | | | | | | | |  | |
|  |  |  | No |  | 0.0054 |  | 1 |  | | | | | | | | | | | |  |  |
|  |  |  |  |  |  |  |  |  | | | | | | | | | | | | | |
|  | | | | | | | | | | 1 |  | | MTK | |  | | 8/9/2021 8:00 AM | |  | |  |
|  | I: Thank you.  P: I told her that “I heard everything but I did not want to argue because I knew that you were saying that because you did not know and it is good not that you have known by yourself…” | | | | | | | | | | | | | | | | | | |  | |
|  |  | | | | | | | | | | | | | | | | | | |  | |
|  | **Codes\\Intervention process\Home situation** | | | | | | | | | | | | | | | | | | |  | |
|  |  |  | No |  | 0.0211 |  | 3 |  | | | | | | | | | | | |  |  |
|  |  |  |  |  |  |  |  |  | | | | | | | | | | | | | |
|  | | | | | | | | | | 1 |  | | MTK | |  | | 8/9/2021 8:00 AM | |  | |  |
|  | I: Can you give an example of what changed?  P1: An example is that we were living in isolation without visiting friends but now we are able to visit friends and if you don’t have something, you ask your friend “I don’t have this, can you share me? Or I need help” We are very thankful. | | | | | | | | | | | | | | | | | | |  | |
|  |  | | | | | | | | | | | | | | | | | | |  | |
|  | | | | | | | | | | 2 |  | | MTK | |  | | 8/9/2021 8:00 AM | |  | |  |
|  | I: you are able to ask from others?  P1: Very much so. | | | | | | | | | | | | | | | | | | |  | |
|  |  | | | | | | | | | | | | | | | | | | |  | |
|  | | | | | | | | | | 3 |  | | MTK | |  | | 8/9/2021 8:00 AM | |  | |  |
|  | I: Or why was it important to have relatives around when you were receiving the counseling intervention? [Sound of children playing in the background]  P7: It was important because the work that I was doing at home was beyond my capacity as a pregnant woman and the inclusion of my mother in law helped a lot because that helped me to be relieved from hard work. Even my father in law admitted that I don’t need to work hard after the counseling. | | | | | | | | | | | | | | | | | | |  | |
|  |  | | | | | | | | | | | | | | | | | | |  | |
|  |  |  |  |  |  |  |  |  |  |  |  |  |  |  |  |  |  |  |  |  |  |
|  |  |  |  |  |  |  |  |  |  |  |  |  |  |  |  |  |  |  |  |  |  |
|  |  |  |  |  |  |  |  |  |  |  |  |  |  |  |  |  |  |  |  |  |  |
| Formatted Reports\\Coding Summary by File Formatted Report | | | | | | | | | Page 26 of 145 | | | | | | | | | | | |  |
| 8/9/2021 8:08 AM | | | | | | | | | | | | | | | | | | | | |  |
|  | **Classification** |  | **Aggregate** |  | **Coverage** |  | **Number Of Coding References** |  | | **Reference Number** | |  | | **Coded By Initials** | |  | | **Modified On** | |  | |
|  | **Codes\\Participation of others\Attitude** | | | | | | | | | | | | | | | | | | |  | |
|  |  |  | No |  | 0.1023 |  | 12 |  | | | | | | | | | | | |  |  |
|  |  |  |  |  |  |  |  |  | | | | | | | | | | | | | |
|  | | | | | | | | | | 1 |  | | MTK | |  | | 8/9/2021 8:00 AM | |  | |  |
|  | I: So let us be on the same page here; at first they have said that “she is coming to provide ARVs,” or “they are satanic…” How did their reactions change overtime after the counseling? Number 2?  P2: I did not know that they are saying that I receive ARVs. Then a friend of mine came. She found [Name of volunteer] counseling me. After the counseling, she confessed that “I was wondering they were saying that you come here to provide ARVs but this is good counseling and I have also learned.” Other people started coming during the counseling sessions to sit in. It was shameful because it was the aunt of my husband who was fabricating that. | | | | | | | | | | | | | | | | | | |  | |
|  |  | | | | | | | | | | | | | | | | | | |  | |
|  | | | | | | | | | | 2 |  | | MTK | |  | | 8/9/2021 8:00 AM | |  | |  |
|  | I: Okay thank you very much.  P: My mother in law confessed when she realized that it was not what people were saying. “Sorry we thought you were satanic people because that is what people were saying but this is a good intervention.” | | | | | | | | | | | | | | | | | | |  | |
|  |  | | | | | | | | | | | | | | | | | | |  | |
|  | | | | | | | | | | 3 |  | | MTK | |  | | 8/9/2021 8:00 AM | |  | |  |
|  | I: Okay thank you very much. Others?  P3: I heard from my husband. When he came back from where he went he said “Mother of [Name of child], people are saying that the counselor comes to give us ARVs.” I called my neighbor because she is the one who has been present during the counseling and she said “I heard this sometime back that you take ARVs. I wanted you to hear it by yourselves.” Then when the counselor came to tell me that I should come here, when I went back home, that was what they were saying “people are saying that you have an infection. Why are you hiding the infection?” I was trying to explain to them that “she comes for counseling and this is what we discuss…” but there are some people who do not understand much as there are others who understand…. | | | | | | | | | | | | | | | | | | |  | |
|  |  |  |  |  |  |  |  |  |  |  |  |  |  |  |  |  |  |  |  |  |  |
|  |  | | | | | | | | | | | | | | | | | | |  | |
| Formatted Reports\\Coding Summary by File Formatted Report | | | | | | | | | Page 29 of 145 | | | | | | | | | | | |  |
| 8/9/2021 8:08 AM | | | | | | | | | | | | | | | | | | | | |  |
|  | **Classification** |  | **Aggregate** |  | **Coverage** |  | **Number Of Coding References** |  | | **Reference Number** | |  | | **Coded By Initials** | |  | | **Modified On** | |  | |
|  |  |  |  |  |  |  |  |  |  |  |  |  |  |  |  |  |  |  |  |  |  |
|  | | | | | | | | | | 4 |  | | MTK | |  | | 8/9/2021 8:00 AM | |  | |  |
|  | P4: They were saying “people are saying she provides ARVs but then where are they?” They were surprised when they heard that the discussion is about how to take care of the child and they said “the issue of ARVs is not true. We also need to have access to this counseling so that in future our children should be healthy.” | | | | | | | | | | | | | | | | | | |  | |
|  |  | | | | | | | | | | | | | | | | | | |  | |
|  | | | | | | | | | | 5 |  | | MTK | |  | | 8/9/2021 8:00 AM | |  | |  |
|  | I: Number 15? How did your relatives react to the counseling?  P15: They accepted it and when the counselor was coming, she was finding that the people were there waiting. | | | | | | | | | | | | | | | | | | |  | |
|  |  | | | | | | | | | | | | | | | | | | |  | |
|  | | | | | | | | | | 6 |  | | MTK | |  | | 8/9/2021 8:00 AM | |  | |  |
|  | I: Were there experiences of opposition from the people surrounding you?  P: The relatives of my husband called for me to ask “where is the person who comes to your house coming from?” I explained everything to them. Then because my neighbor always go there to talk issues about me and those people they tell me everything that my neighbor tells them. So I just leave her like that because there was nothing else I could do. | | | | | | | | | | | | | | | | | | |  | |
|  |  | | | | | | | | | | | | | | | | | | |  | |
|  | | | | | | | | | | 7 |  | | MTK | |  | | 8/9/2021 8:00 AM | |  | |  |
|  | I: meaning that those people were talking about things which they were told by your neighbor?  P: Yes.  I: But your neighbor went to another person…  P: Yes but I explained to them everything in details. | | | | | | | | | | | | | | | | | | |  | |
|  |  | | | | | | | | | | | | | | | | | | |  | |
|  | | | | | | | | | | 8 |  | | MTK | |  | | 8/9/2021 8:00 AM | |  | |  |
|  | I: And what happened next?  P: Nothing happened because after explaining to them, they understood everything. | | | | | | | | | | | | | | | | | | |  | |
|  |  | | | | | | | | | | | | | | | | | | |  | |
|  | | | | | | | | | | 9 |  | | MTK | |  | | 8/9/2021 8:00 AM | |  | |  |
|  | I: Thank you very much. So since you are many, I would like to hear from someone who faced negative reaction from relatives. Reactions against the counseling… we would like to hear those experiences.  P: On that one, there can be no experiences of negative reactions because in most cases, pregnant women are in contact with health workers and no one can be against that. | | | | | | | | | | | | | | | | | | |  | |
|  |  | | | | | | | | | | | | | | | | | | |  | |
|  | | | | | | | | | | 10 |  | | MTK | |  | | 8/9/2021 8:00 AM | |  | |  |
|  | P: Someone came to get my health profile, she checked where HIV testing results are indicated and when she saw the results she did not have anything to say. | | | | | | | | | | | | | | | | | | |  | |
|  |  | | | | | | | | | | | | | | | | | | |  | |
|  | | | | | | | | | | 11 |  | | MTK | |  | | 8/9/2021 8:00 AM | |  | |  |
|  | Note taker: I asked this question because she asked a question about how the family accepted the intervention and how the community perceived it but we also wanted to hear about how the husbands perceived it…  P1: They accepted it. | | | | | | | | | | | | | | | | | | |  | |
|  |  | | | | | | | | | | | | | | | | | | |  | |
|  | | | | | | | | | | 12 |  | | MTK | |  | | 8/9/2021 8:00 AM | |  | |  |
|  | Note taker: What do others say?  P9, P11, P12, P14, P10, P6, P4, P3: Agree with number 1 | | | | | | | | | | | | | | | | | | |  | |
|  |  | | | | | | | | | | | | | | | | | | |  | |
|  | **Codes\\Participation of others\Community participation** | | | | | | | | | | | | | | | | | | |  | |
|  |  |  | No |  | 0.0521 |  | 4 |  | | | | | | | | | | | |  |  |
|  |  |  |  |  |  |  |  |  | | | | | | | | | | | | | |
|  | | | | | | | | | | 1 |  | | MTK | |  | | 8/9/2021 8:00 AM | |  | |  |
|  | I: Okay fine. Are there other additions? We should now move from the close relatives… how did the community perceive the counseling intervention?  P4: When people understood that I don’t take ARVs, the community liked this intervention and they said that the counseling should continue. | | | | | | | | | | | | | | | | | | |  | |
|  |  | | | | | | | | | | | | | | | | | | |  | |
| Formatted Reports\\Coding Summary by File Formatted Report | | | | | | | | | Page 30 of 145 | | | | | | | | | | | |  |
| 8/9/2021 8:08 AM | | | | | | | | | | | | | | | | | | | | |  |
|  | **Classification** |  | **Aggregate** |  | **Coverage** |  | **Number Of Coding References** |  | | **Reference Number** | |  | | **Coded By Initials** | |  | | **Modified On** | |  | |
|  |  |  |  |  |  |  |  |  |  |  |  |  |  |  |  |  |  |  |  |  |  |
|  | | | | | | | | | | 2 |  | | MTK | |  | | 8/9/2021 8:00 AM | |  | |  |
|  | I: When the community understood the concept of counseling, they accepted it and said it should continue. What about others?  P9: I had a similar experience. | | | | | | | | | | | | | | | | | | |  | |
|  |  | | | | | | | | | | | | | | | | | | |  | |
|  | | | | | | | | | | 3 |  | | MTK | |  | | 8/9/2021 8:00 AM | |  | |  |
|  | I: Others…? Because it is possible to accept the intervention as a family but the community can be against it and if they can see the counselors, they can reach the extent of chasing them… “We don’t want this here…”  P14: In my community, when people saw that [Name of volunteer] has come, they were sending their children who are pregnant and those with young children so that they should come and benefit from the counseling intervention.  I: Others?  P6: My community is more than willing and they would like to benefit like wise because when they hear that I have been called here at the hospital, pregnant women wish if they were part of the intervention so that they can also benefit from the service that I am accessing.  I: [There is noise of women crying/mourning in the background. Audio was paused]… Okay… we were discussing about the community… how they reacted. Are there additions.  P12: The counseling was accepted by the community because when the counselor was coming, even married people who are not pregnant in the community wished if they could have an opportunity to access the counseling so that they can be able to deal with stress and depression | | | | | | | | | | | | | | | | | | |  | |
|  |  |  |  |  |  |  |  |  |  |  |  |  |  |  |  |  |  |  |  |  |  |
|  |  | | | | | | | | | | | | | | | | | | |  | |
|  | | | | | | | | | | 4 |  | | MTK | |  | | 8/9/2021 8:00 AM | |  | |  |
|  | I: Others?  P: My community is very happy with the counseling because they see how I have changed and they admit that “there is great difference from the way we have been seeing you. It seems the women who was coming has really helped you.” The chief is happy with the intervention. | | | | | | | | | | | | | | | | | | |  | |
|  |  | | | | | | | | | | | | | | | | | | |  | |
|  |  |  |  |  |  |  |  |  |  |  |  |  |  |  |  |  |  |  |  |  |  |
|  |  |  |  |  |  |  |  |  |  |  |  |  |  |  |  |  |  |  |  |  |  |
|  |  |  |  |  |  |  |  |  |  |  |  |  |  |  |  |  |  |  |  |  |  |
|  |  |  |  |  |  |  |  |  |  |  |  |  |  |  |  |  |  |  |  |  |  |
|  |  |  |  |  |  |  |  |  |  |  |  |  |  |  |  |  |  |  |  |  |  |
| Formatted Reports\\Coding Summary by File Formatted Report | | | | | | | | | Page 31 of 145 | | | | | | | | | | | |  |
| 8/9/2021 8:08 AM | | | | | | | | | | | | | | | | | | | | |  |
|  | **Classification** |  | **Aggregate** |  | **Coverage** |  | **Number Of Coding References** |  | | **Reference Number** | |  | | **Coded By Initials** | |  | | **Modified On** | |  | |
|  |  | | | | | | | | | | | | | | | | | | |  | |
|  | **Codes\\Recommendations\General** | | | | | | | | | | | | | | | | | | |  | |
|  |  |  | No |  | 0.1140 |  | 11 |  | | | | | | | | | | | |  |  |
|  |  |  |  |  |  |  |  |  | | | | | | | | | | | | | |
|  | | | | | | | | | | 1 |  | | MTK | |  | | 8/9/2021 8:00 AM | |  | |  |
|  | I: So it has helped you to deal with depression and also to help other people deal with depression. What do others say?  P: My comment is that it is good to deal with depression and we need to help our friends to deal with depression the way we were helped to deal with depression and this counseling strategy should not stop. | | | | | | | | | | | | | | | | | | |  | |
|  |  | | | | | | | | | | | | | | | | | | |  | |
|  | | | | | | | | | | 2 |  | | MTK | |  | | 8/9/2021 8:00 AM | |  | |  |
|  | I: If the government may decide to roll out this special counseling intervention to pregnant women, you as pioneers of this program, what would you say? Say anything you want… Are we together?  All: Yes!  P12: it can be good that the government should consider rolling out this counseling intervention so that future pregnant women should live happy lives and be able to give birth to healthy babies. | | | | | | | | | | | | | | | | | | |  | |
|  |  | | | | | | | | | | | | | | | | | | |  | |
|  | | | | | | | | | | 3 |  | | MTK | |  | | 8/9/2021 8:00 AM | |  | |  |
|  | I: What do others say?  Note taker: What are you going to tell the government?  P1: The government should consider planning to start providing this counseling intervention when the woman is four to five months so that she should be visited several times. | | | | | | | | | | | | | | | | | | |  | |
|  |  | | | | | | | | | | | | | | | | | | |  | |
|  | | | | | | | | | | 4 |  | | MTK | |  | | 8/9/2021 8:00 AM | |  | |  |
|  | I: That is number 1. What do others say?  P: I can tell the government to continue with this counseling intervention so that the future mothers should also benefit. | | | | | | | | | | | | | | | | | | |  | |
|  |  | | | | | | | | | | | | | | | | | | |  | |
| Formatted Reports\\Coding Summary by File Formatted Report | | | | | | | | | Page 34 of 145 | | | | | | | | | | | |  |
| 8/9/2021 8:08 AM | | | | | | | | | | | | | | | | | | | | |  |
|  | **Classification** |  | **Aggregate** |  | **Coverage** |  | **Number Of Coding References** |  | | **Reference Number** | |  | | **Coded By Initials** | |  | | **Modified On** | |  | |
|  |  |  |  |  |  |  |  |  |  |  |  |  |  |  |  |  |  |  |  |  |  |
|  | | | | | | | | | | 5 |  | | MTK | |  | | 8/9/2021 8:00 AM | |  | |  |
|  | I: Others?  The government needs to continue with this program… [Not audible due to noise of children hissing in the background]… We are worried that “am I going to make it to deliver the child? So they need to be supported.  I: Others?  P: This counseling should continue because some pregnant women are not married and beside the counseling sessions, the government should also provide financial support so that they can be able to buy basic needs. | | | | | | | | | | | | | | | | | | |  | |
|  |  | | | | | | | | | | | | | | | | | | |  | |
|  | | | | | | | | | | 6 |  | | MTK | |  | | 8/9/2021 8:00 AM | |  | |  |
|  | I: Counseling and financial support. Others, what do you say?  P3: This counseling should continue and as she has said, the government should be able to give us something so that we should be motivated.  I: What support?  P: To say the truth, the support is financial support that is required because they cannot say that I should be feeding the child when I have no food. Nowadays we need to buy everything and we need to have money to buy. | | | | | | | | | | | | | | | | | | |  | |
|  |  | | | | | | | | | | | | | | | | | | |  | |
|  | | | | | | | | | | 7 |  | | MTK | |  | | 8/9/2021 8:00 AM | |  | |  |
|  | I: Number 9?  P9: The government needs to go ahead with implementation of the counseling intervention but not in terms of food because food is locally available. It is during the rainy season that many people were struggling to find food but nowadays there is plenty of soya beans. Free things are not sustainable but we should just encourage the government to continue with the implementation of the intervention. | | | | | | | | | | | | | | | | | | |  | |
|  |  | | | | | | | | | | | | | | | | | | |  | |
|  | | | | | | | | | | 8 |  | | MTK | |  | | 8/9/2021 8:00 AM | |  | |  |
|  | I: Number 15?  P15: I can say that this counseling intervention because most of the men are abusive because they impregnate the woman but are not responsible enough to take care of the woman. In this case, counseling should continue so that men should be educated on how they can take care of their families.  I: Number 11?  P11: We are encouraging the government to continue with the counseling so that other pregnant women should be helped. | | | | | | | | | | | | | | | | | | |  | |
|  |  | | | | | | | | | | | | | | | | | | |  | |
|  | | | | | | | | | | 9 |  | | MTK | |  | | 8/9/2021 8:00 AM | |  | |  |
|  | P10: The government should continue with the counseling intervention to pregnant women because more women continue to become pregnant and there is need to know the importance of resting for pregnant women. | | | | | | | | | | | | | | | | | | |  | |
|  |  | | | | | | | | | | | | | | | | | | |  | |
|  | | | | | | | | | | 10 |  | | MTK | |  | | 8/9/2021 8:00 AM | |  | |  |
|  | I: In other words, for you to receive the intervention at your home, it was the counselor who was traveling but in this case, you are the one to travel… what do you think?  P14: Transport is a challenge.  P: Unless it is close to home, we can walk because we want to access the counseling service. Also, people have bicycles in the village and if you have good friends, they can borrow you a bicycle and you can go to access the service by bicycle…  Choral: Cross talk [Not audible] | | | | | | | | | | | | | | | | | | |  | |
|  |  | | | | | | | | | | | | | | | | | | |  | |
|  | | | | | | | | | | 11 |  | | MTK | |  | | 8/9/2021 8:00 AM | |  | |  |
|  | I: Why don’t you vote?  All: Yes!  I: For those who say that counseling should be provided at home raise your hands…  All: [Participants raise hands]…  I: 1, 2, 3, 4, 5, 6, 7, 8, 9, 10, 11, 12… And those who say the counseling should be provided at some place?  P14: We are four.  All: [Laughter]… | | | | | | | | | | | | | | | | | | |  | |
|  |  |  |  |  |  |  |  |  |  |  |  |  |  |  |  |  |  |  |  |  |  |
|  |  | | | | | | | | | | | | | | | | | | |  | |
|  |  |  |  |  |  |  |  |  |  |  |  |  |  |  |  |  |  |  |  |  |  |
| Formatted Reports\\Coding Summary by File Formatted Report | | | | | | | | | Page 35 of 145 | | | | | | | | | | | |  |
| 8/9/2021 8:08 AM | | | | | | | | | | | | | | | | | | | | |  |
|  | **Classification** |  | **Aggregate** |  | **Coverage** |  | **Number Of Coding References** |  | | **Reference Number** | |  | | **Coded By Initials** | |  | | **Modified On** | |  | |
|  | **Codes\\Recommendations\Intervention process** | | | | | | | | | | | | | | | | | | |  | |
|  |  |  | No |  | 0.0142 |  | 1 |  | | | | | | | | | | | |  |  |
|  |  |  |  |  |  |  |  |  | | | | | | | | | | | | | |
|  | | | | | | | | | | 1 |  | | MTK | |  | | 8/9/2021 8:00 AM | |  | |  |
|  | P9: When there was something sensitive, the counselor was asking the other people to go out so that they should not hear what was being discussed between you and the counselor. So the counseling should be done at home but when there are sensitive issues, the other people should not be present they should give room for private discussion between you and the counselor because if they are present, you cannot be free. The issue of food is general but there are some confidential issues that don’t require big audience. | | | | | | | | | | | | | | | | | | |  | |
|  |  | | | | | | | | | | | | | | | | | | |  | |
|  | **Codes\\THPP providers** | | | | | | | | | | | | | | | | | | |  | |
|  |  |  | No |  | 0.0169 |  | 2 |  | | | | | | | | | | | |  |  |
|  |  |  |  |  |  |  |  |  | | | | | | | | | | | | | |
|  | | | | | | | | | | 1 |  | | MTK | |  | | 8/9/2021 8:00 AM | |  | |  |
|  | I: Behind there, do you have something to say?  P: The counselors helped us to deal with the stress that we had. | | | | | | | | | | | | | | | | | | |  | |
|  |  | | | | | | | | | | | | | | | | | | |  | |
|  | | | | | | | | | | 2 |  | | MTK | |  | | 8/9/2021 8:00 AM | |  | |  |
|  | I: Are there additions?  P: I did not have means to start antenatal clinic…  I: You did not have means?  P: Yes, I did not have means  I: Can you clarify?  P: She mean that she did not have a partner the time when she came to start antenatal clinic…  I: [Laughter]… Now I understand… can you tell me what happened?  P: What happened was that the man did not marry me and when the counselor came, she told me that I should come here to start antenatal clinic and that was when I was screened for depression. | | | | | | | | | | | | | | | | | | |  | |
|  |  |  |  |  |  |  |  |  |  |  |  |  |  |  |  |  |  |  |  |  |  |
|  |  | | | | | | | | | | | | | | | | | | |  | |
|  |  |  |  |  |  |  |  |  |  |  |  |  |  |  |  |  |  |  |  |  |  |
|  |  |  |  |  |  |  |  |  |  |  |  |  |  |  |  |  |  |  |  |  |  |
|  |  |  |  |  |  |  |  |  |  |  |  |  |  |  |  |  |  |  |  |  |  |
|  |  |  |  |  |  |  |  |  |  |  |  |  |  |  |  |  |  |  |  |  |  |
| Formatted Reports\\Coding Summary by File Formatted Report | | | | | | | | | Page 37 of 145 | | | | | | | | | | | |  |
| 8/9/2021 8:08 AM | | | | | | | | | | | | | | | | | | | | |  |
|  | **Classification** |  | **Aggregate** |  | **Coverage** |  | **Number Of Coding References** |  | | **Reference Number** | |  | | **Coded By Initials** | |  | | **Modified On** | |  | |
|  | **Files\\IDIs\\IDI #1** | | | | | | | | | | | | | | | | | | |  | |
|  | **Code** | | | | | | | | | | | | | | | | | | |  | |
|  | **Codes\\Intervention experience** | | | | | | | | | | | | | | | | | | |  | |
|  |  |  | No |  | 0.1219 |  | 1 |  | | | | | | | | | | | |  |  |
|  |  |  |  |  |  |  |  |  | | | | | | | | | | | | | |
|  | | | | | | | | | | 1 |  | | MTK | |  | | 8/3/2021 8:54 PM | |  | |  |
|  | I: Do you remember how many times you were visited?  P: eight visits and when you told her to come the ninth visit, she came and it was the one we talked over phone.  I: What was your understanding of the visits that were conducted?  P: So that I could change my life that I was leading.  I: What do you mean when you say changing the life that you had?  P: I mean that I was leading an oppressed life but now I am healthy because previously I was looking down on myself. sometimes I was not talking to anyone and maybe I could isolate myself but with the counseling that I received, I am able to associate with friends and even when she [THE VC] comes, I could invite my friends that “come we should be together here.” Sure.  I: What do you mean when you say looking down on yourself? Maybe I do not understand the language…  P: I was not happy in my life. I could be disappointed with minor issues but with the counseling, things changed.  I: You have said that with the counselling, things changed. Can you tell me more about this program? What was this support all about? What was she doing? How was the counseling provided?  P: The counseling session was between her and myself so that my issues I had could be addressed. So I saw that it was a wonderful support because I could not manage the issues by myself. | | | | | | | | | | | | | | | | | | |  | |
|  |  |  |  |  |  |  |  |  |  |  |  |  |  |  |  |  |  |  |  |  |  |
|  |  |  |  |  |  |  |  |  |  |  |  |  |  |  |  |  |  |  |  |  |  |
|  |  | | | | | | | | | | | | | | | | | | |  | |
|  | **Codes\\Intervention experience\Aspects** | | | | | | | | | | | | | | | | | | |  | |
|  |  |  | No |  | 0.0833 |  | 1 |  | | | | | | | | | | | |  |  |
|  |  |  |  |  |  |  |  |  | | | | | | | | | | | | | |
|  | | | | | | | | | | 1 |  | | MTK | |  | | 8/3/2021 6:48 PM | |  | |  |
|  | I: Mh. Which part of counseling did you see to be the most helpful?  P: The most helpful part of the counseling was concerning maternal health that the child should be exclusively breastfed so that s/he should be healthy. At six months, the child should be given other foods but they should be the six food groups. This part was great.  I: Okay. So you have said that she was counseling you on the health of the child and the six food groups and your health?  P: Yes.  I: Meaning that you should also be healthy?  P: Yes.  I: What else?  P: The other thing was about our association with friends. after completing household chores, we can go and chat with friends.  I: Was there anyone from your family who joined you during the counseling session?  P: No.  I: None…  P: Because I am away from the family members, I am staying at a rented house at a trading centre but I am close to neighbors with whom I interact well. | | | | | | | | | | | | | | | | | | |  | |
|  |  |  |  |  |  |  |  |  |  |  |  |  |  |  |  |  |  |  |  |  |  |
|  |  |  |  |  |  |  |  |  |  |  |  |  |  |  |  |  |  |  |  |  |  |
|  |  | | | | | | | | | | | | | | | | | | |  | |
|  | **Codes\\Intervention experience\Barriers** | | | | | | | | | | | | | | | | | | |  | |
|  |  |  | No |  | 0.0112 |  | 1 |  | | | | | | | | | | | |  |  |
|  |  |  |  |  |  |  |  |  | | | | | | | | | | | | | |
|  | | | | | | | | | | 1 |  | | MTK | |  | | 8/3/2021 7:00 PM | |  | |  |
|  | I: Were there any challenges that you faced for receiving the intervention at home such as stigma?  P: No. there was no problem | | | | | | | | | | | | | | | | | | |  | |
|  |  | | | | | | | | | | | | | | | | | | |  | |
| Formatted Reports\\Coding Summary by File Formatted Report | | | | | | | | | Page 38 of 145 | | | | | | | | | | | |  |
| 8/9/2021 8:08 AM | | | | | | | | | | | | | | | | | | | | |  |
|  | **Classification** |  | **Aggregate** |  | **Coverage** |  | **Number Of Coding References** |  | | **Reference Number** | |  | | **Coded By Initials** | |  | | **Modified On** | |  | |
|  | **Codes\\Intervention experience\Dislikes** | | | | | | | | | | | | | | | | | | |  | |
|  |  |  | No |  | 0.0084 |  | 1 |  | | | | | | | | | | | |  |  |
|  |  |  |  |  |  |  |  |  | | | | | | | | | | | | | |
|  | | | | | | | | | | 1 |  | | MTK | |  | | 8/3/2021 6:50 PM | |  | |  |
|  | I: Was there anything you feel that you did not really like about the counseling?  P: No nothing. | | | | | | | | | | | | | | | | | | |  | |
|  |  | | | | | | | | | | | | | | | | | | |  | |
|  | **Codes\\Intervention experience\Facilitators** | | | | | | | | | | | | | | | | | | |  | |
|  |  |  | No |  | 0.0367 |  | 1 |  | | | | | | | | | | | |  |  |
|  |  |  |  |  |  |  |  |  | | | | | | | | | | | | | |
|  | | | | | | | | | | 1 |  | | MTK | |  | | 8/3/2021 8:46 PM | |  | |  |
|  | I: Was she just coming or you were agreeing on the next visit date?  P: We were agreeing. When she came on Thursday, the next visit was on a Thursday. If she is preoccupied with funeral activities, she would come on Friday and would say “forgive me for coming today. I was preoccupied with funeral activities but thanks for getting you at home” because there was no day that she found that I was away. I was always ready. | | | | | | | | | | | | | | | | | | |  | |
|  |  | | | | | | | | | | | | | | | | | | |  | |
|  | **Codes\\Intervention experience\Likes** | | | | | | | | | | | | | | | | | | |  | |
|  |  |  | No |  | 0.0413 |  | 1 |  | | | | | | | | | | | |  |  |
|  |  |  |  |  |  |  |  |  | | | | | | | | | | | | | |
|  | | | | | | | | | | 1 |  | | MTK | |  | | 8/3/2021 8:48 PM | |  | |  |
|  | I: Was there anything that you feel you liked the most in this intervention?  P: The programme was really good because most of the things were not known to me for all the births that I have given but I have known many things this time. I was just giving birth to children without knowing what happens but when I came here for antenatal care, I met you people and you said “we will find you at your home” and when you came and offered me counseling, it made me feel happy. | | | | | | | | | | | | | | | | | | |  | |
|  |  | | | | | | | | | | | | | | | | | | |  | |
|  | **Codes\\Intervention process** | | | | | | | | | | | | | | | | | | |  | |
|  |  |  | No |  | 0.0369 |  | 1 |  | | | | | | | | | | | |  |  |
|  |  |  |  |  |  |  |  |  | | | | | | | | | | | | | |
|  | | | | | | | | | | 1 |  | | MTK | |  | | 8/3/2021 6:50 PM | |  | |  |
|  | I: Was she just coming or you were agreeing on the next visit date?  P: We were agreeing. When she came on Thursday, the next visit was on a Thursday. If she is preoccupied with funeral activities, she would come on Friday and would say “forgive me for coming today. I was preoccupied with funeral activities but thanks for getting you at home” because there was no day that she found that I was away. I was always ready. | | | | | | | | | | | | | | | | | | |  | |
|  |  | | | | | | | | | | | | | | | | | | |  | |
|  | **Codes\\Intervention process\Delivery** | | | | | | | | | | | | | | | | | | |  | |
|  |  |  | No |  | 0.0339 |  | 1 |  | | | | | | | | | | | |  |  |
|  |  |  |  |  |  |  |  |  | | | | | | | | | | | | | |
|  | | | | | | | | | | 1 |  | | MTK | |  | | 8/3/2021 8:46 PM | |  | |  |
|  | I: Can you tell me more about getting the counseling intervention at your home versus getting it from the clinic? What is your opinion.  P: It is good. You can still receive the intervention at your home but to know more about things, we know it from the clinic just like what happened with us that when we went to the clinic, it was when we were told about the counseling intervention. | | | | | | | | | | | | | | | | | | |  | |
|  |  | | | | | | | | | | | | | | | | | | |  | |
|  |  |  |  |  |  |  |  |  |  |  |  |  |  |  |  |  |  |  |  |  |  |
| Formatted Reports\\Coding Summary by File Formatted Report | | | | | | | | | Page 39 of 145 | | | | | | | | | | | |  |
| 8/9/2021 8:08 AM | | | | | | | | | | | | | | | | | | | | |  |
|  | **Classification** |  | **Aggregate** |  | **Coverage** |  | **Number Of Coding References** |  | | **Reference Number** | |  | | **Coded By Initials** | |  | | **Modified On** | |  | |
|  | **Codes\\Intervention process\Feelings** | | | | | | | | | | | | | | | | | | |  | |
|  |  |  | No |  | 0.0411 |  | 1 |  | | | | | | | | | | | |  |  |
|  |  |  |  |  |  |  |  |  | | | | | | | | | | | | | |
|  | | | | | | | | | | 1 |  | | MTK | |  | | 8/3/2021 8:45 PM | |  | |  |
|  | I: I mean that you were getting the counseling intervention at home instead of getting it from the clinic. What is your perception? How can you differentiate the two; receiving the counseling intervention at home versus at the clinic?  P: It is okay to receive the counseling intervention at home.  I: How good is it?  P: It is good because as I said, at home there are people whom you can invite to be part of the sessions and the other family members can also benefit. | | | | | | | | | | | | | | | | | | |  | |
|  |  | | | | | | | | | | | | | | | | | | |  | |
|  | **Codes\\Participation of others\Others** | | | | | | | | | | | | | | | | | | |  | |
|  |  |  | No |  | 0.0837 |  | 3 |  | | | | | | | | | | | |  |  |
|  |  |  |  |  |  |  |  |  | | | | | | | | | | | | | |
|  | | | | | | | | | | 1 |  | | MTK | |  | | 8/3/2021 8:49 PM | |  | |  |
|  | I: Did he go for a walk or for tobacco work as you said?  P: My neighbor who took part in the counseling is the one who was busy in the tobacco field, not him [the spouse]. He just participated for a short time then left for a walk. The rest of the session it was me and the volunteer | | | | | | | | | | | | | | | | | | |  | |
|  |  | | | | | | | | | | | | | | | | | | |  | |
|  | | | | | | | | | | 2 |  | | MTK | |  | | 8/3/2021 8:44 PM | |  | |  |
|  | I: How did your family members receive it/perceived it that you will be visited by counselors and receive the counseling sessions at your home…? But before that, let us hear about how you perceived it.  P: I welcomed it because when I was told at the clinic, I told my partner when I got home and he said “is that the case? It is okay. Let the people come and welcome them and chat with them as your fellow women”. That is what he said. | | | | | | | | | | | | | | | | | | |  | |
|  |  | | | | | | | | | | | | | | | | | | |  | |
|  | | | | | | | | | | 3 |  | | MTK | |  | | 8/3/2021 6:54 PM | |  | |  |
|  | I: How about people surrounding you, how did they welcome this? Did it look new or how did they receive it?  P: Since this is a new thing, the really looked at it as a new thing.  I: Since it is just beginning, did it bring you any challenges?  P: No. | | | | | | | | | | | | | | | | | | |  | |
|  |  | | | | | | | | | | | | | | | | | | |  | |
|  | **Codes\\Participation of others\Partners** | | | | | | | | | | | | | | | | | | |  | |
|  |  |  | No |  | 0.0560 |  | 1 |  | | | | | | | | | | | |  |  |
|  |  |  |  |  |  |  |  |  | | | | | | | | | | | | | |
|  | | | | | | | | | | 1 |  | | MTK | |  | | 8/3/2021 6:49 PM | |  | |  |
|  | I: Was there time when your partner was there when counseling was provided to you?  P: One day.  I: One day… What happened for him to be present only for one day?  P: It was because when ……….. [VC] came, he was there and we said that since people are busy with tobacco work, he should join the discussion but he was there for few minutes then he went for a walk.  I: Did he go for a walk or for tobacco work as you said?  P: My neighbor who took part in the counseling is the one who was busy in the tobacco field, not him. He just participated for a short time then left for a walk. The rest of the session it was me and the volunteer | | | | | | | | | | | | | | | | | | |  | |
|  |  |  |  |  |  |  |  |  |  |  |  |  |  |  |  |  |  |  |  |  |  |
|  |  | | | | | | | | | | | | | | | | | | |  | |
|  |  | | | | | | | | | | | | | | | | | | |  | |
| Formatted Reports\\Coding Summary by File Formatted Report | | | | | | | | | Page 40 of 145 | | | | | | | | | | | |  |
| 8/9/2021 8:08 AM | | | | | | | | | | | | | | | | | | | | |  |
|  | **Classification** |  | **Aggregate** |  | **Coverage** |  | **Number Of Coding References** |  | | **Reference Number** | |  | | **Coded By Initials** | |  | | **Modified On** | |  | |
|  | **Codes\\Personal history** | | | | | | | | | | | | | | | | | | |  | |
|  |  |  | No |  | 0.0897 |  | 1 |  | | | | | | | | | | | |  |  |
|  |  |  |  |  |  |  |  |  | | | | | | | | | | | | | |
|  | | | | | | | | | | 1 |  | | MTK | |  | | 8/3/2021 6:42 PM | |  | |  |
|  | P: I am [NAME OF PARTICIPANT]  I: [NAME OF PARTICIPANT]?  P: Yes.  I: So because of the masks we are wearing, may you raise your voice?  P: Okay.  I: So that we should hear the voice.  P: I am [NAME OF PARTICIPANT]. I come from……. Village  I: Okay fine. How old are you?  P: I am thirty two years old.  I: Fine. How far did you go with your education?  P: I did not go far. I went up to standard five.  I: You really did it… Are you married?  P: Yes.  I: Okay fine. Is this your first or second marriage?  P: My first marriage.  I: Okay. How many children do you have?  P: Five.  I: Is this newborn the fifth one?  P: Yes.  I: Are all the children alive?  P: Yes.  I: How many times have you been pregnant?  P: Five times.  I: Do you have a phone number so that we can contact you if there is need?  P: My partner’s.  I: Your partner’s… did you memorize it?  P: I wrote in the health passport book. | | | | | | | | | | | | | | | | | | |  | |
|  |  |  |  |  |  |  |  |  |  |  |  |  |  |  |  |  |  |  |  |  |  |
|  |  |  |  |  |  |  |  |  |  |  |  |  |  |  |  |  |  |  |  |  |  |
|  |  |  |  |  |  |  |  |  |  |  |  |  |  |  |  |  |  |  |  |  |  |
|  |  |  |  |  |  |  |  |  |  |  |  |  |  |  |  |  |  |  |  |  |  |
|  |  | | | | | | | | | | | | | | | | | | |  | |
|  | **Codes\\Place of intervention delivery** | | | | | | | | | | | | | | | | | | |  | |
|  |  |  | No |  | 0.0753 |  | 1 |  | | | | | | | | | | | |  |  |
|  |  |  |  |  |  |  |  |  | | | | | | | | | | | | | |
|  | | | | | | | | | | 1 |  | | MTK | |  | | 8/3/2021 6:51 PM | |  | |  |
|  | I: Can you tell me more about getting the counseling intervention at your home versus getting it from the clinic? What is your opinion.  P: It is good. You can still receive the intervention at your home but to know more about things, we know it from the clinic just like what happened with us that when we went to the clinic, it was when we were told about the counseling intervention.  I: I mean that you were getting the counseling intervention at home instead of getting it from the clinic. What is your perception? How can you differentiate the two; receiving the counseling intervention at home versus at the clinic?  P: It is okay to receive the counseling intervention at home.  I: How good is it?  P: It is good because as I said, at home there are people whom you can invite to be part of the sessions and the other family members can also benefit. | | | | | | | | | | | | | | | | | | |  | |
|  |  |  |  |  |  |  |  |  |  |  |  |  |  |  |  |  |  |  |  |  |  |
|  |  | | | | | | | | | | | | | | | | | | |  | |
|  |  |  |  |  |  |  |  |  |  |  |  |  |  |  |  |  |  |  |  |  |  |
| Formatted Reports\\Coding Summary by File Formatted Report | | | | | | | | | Page 41 of 145 | | | | | | | | | | | |  |
| 8/9/2021 8:08 AM | | | | | | | | | | | | | | | | | | | | |  |
|  | **Classification** |  | **Aggregate** |  | **Coverage** |  | **Number Of Coding References** |  | | **Reference Number** | |  | | **Coded By Initials** | |  | | **Modified On** | |  | |
|  | **Codes\\Place of intervention delivery\Advantages** | | | | | | | | | | | | | | | | | | |  | |
|  |  |  | No |  | 0.0150 |  | 1 |  | | | | | | | | | | | |  |  |
|  |  |  |  |  |  |  |  |  | | | | | | | | | | | | | |
|  | | | | | | | | | | 1 |  | | MTK | |  | | 8/3/2021 6:52 PM | |  | |  |
|  | I: How good is it?  P: It is good because as I said, at home there are people whom you can invite to be part of the sessions and the other family members can also benefit. | | | | | | | | | | | | | | | | | | |  | |
|  |  | | | | | | | | | | | | | | | | | | |  | |
|  | **Codes\\Recommendations\Intervention process** | | | | | | | | | | | | | | | | | | |  | |
|  |  |  | No |  | 0.0595 |  | 1 |  | | | | | | | | | | | |  |  |
|  |  |  |  |  |  |  |  |  | | | | | | | | | | | | | |
|  | | | | | | | | | | 1 |  | | MTK | |  | | 8/3/2021 6:56 PM | |  | |  |
|  | I: What can you say about the whole intervention that you received , I know you started explaining a bit but now I would like you to go into details how you have perceived the intervention from the time you started to now? How are things?  P: When we started the sessions, I did not understand, I had a lot of problems but as we went along with the subsequent sessions I saw that things started changing because I was depressed and she was helping me to calm down and deal with the problems, stress triggers. So I saw that this intervention is very helpful.  I: Mh. Can you encourage other people to join this program?  P: Yes I can encourage them. | | | | | | | | | | | | | | | | | | |  | |
|  |  |  |  |  |  |  |  |  |  |  |  |  |  |  |  |  |  |  |  |  |  |
|  |  | | | | | | | | | | | | | | | | | | |  | |
|  |  | | | | | | | | | | | | | | | | | | |  | |
|  |  | | | | | | | | | | | | | | | | | | |  | |
|  | **Codes\\Recommendations\Period** | | | | | | | | | | | | | | | | | | |  | |
|  |  |  | No |  | 0.0581 |  | 1 |  | | | | | | | | | | | |  |  |
|  |  |  |  |  |  |  |  |  | | | | | | | | | | | | | |
|  | | | | | | | | | | 1 |  | | MTK | |  | | 8/3/2021 6:59 PM | |  | |  |
|  | I: Do you think this intervention is essential for every pregnant woman or there should be specific time during pregnancy?  P: On that one I may not be able to specify the gestation period of women to receive the intervention because I came when I was six months pregnant. So you are the ones as health workers to determine.  I: Based on how you have received this intervention, can you say it should be given to women who have just conceived or they can be given at any time during pregnancy?  P: It has to be provided when the woman is six months pregnant because you can conduct several sessions before she delivers and she can be able to follow the program. | | | | | | | | | | | | | | | | | | |  | |
|  |  |  |  |  |  |  |  |  |  |  |  |  |  |  |  |  |  |  |  |  |  |
|  |  | | | | | | | | | | | | | | | | | | |  | |
|  |  |  |  |  |  |  |  |  |  |  |  |  |  |  |  |  |  |  |  |  |  |
| Formatted Reports\\Coding Summary by File Formatted Report | | | | | | | | | Page 42 of 145 | | | | | | | | | | | |  |
| 8/9/2021 8:08 AM | | | | | | | | | | | | | | | | | | | | |  |
|  | **Classification** |  | **Aggregate** |  | **Coverage** |  | **Number Of Coding References** |  | | **Reference Number** | |  | | **Coded By Initials** | |  | | **Modified On** | |  | |
|  | **Codes\\Recommendations\Place of delivery** | | | | | | | | | | | | | | | | | | |  | |
|  |  |  | No |  | 0.0397 |  | 1 |  | | | | | | | | | | | |  |  |
|  |  |  |  |  |  |  |  |  | | | | | | | | | | | | | |
|  | | | | | | | | | | 1 |  | | MTK | |  | | 8/3/2021 6:56 PM | |  | |  |
|  | I: Which one would you choose if you were given an opportunity to receive counseling at home or at the clinic?  P: At home.  I: At home… why?  P: She should be coming. She [volunteer/counselor] should also travel [Laughs]…  I: Meaning that walking to you is a burden?  P: They should come so that when we tell people that “we went to the clinic and this is what we have heard”, they should know that it is true when they see the volunteer coming to visit. | | | | | | | | | | | | | | | | | | |  | |
|  |  |  |  |  |  |  |  |  |  |  |  |  |  |  |  |  |  |  |  |  |  |
|  |  | | | | | | | | | | | | | | | | | | |  | |
|  | **Codes\\THPP providers** | | | | | | | | | | | | | | | | | | |  | |
|  |  |  | No |  | 0.0238 |  | 1 |  | | | | | | | | | | | |  |  |
|  |  |  |  |  |  |  |  |  | | | | | | | | | | | | | |
|  | | | | | | | | | | 1 |  | | MTK | |  | | 8/3/2021 6:44 PM | |  | |  |
|  | I: Okay, it means we will have it at the end of the discussion. So as I said, the aim of our discussion today is to learn from you how the counseling sessions that you had with the volunteers went. | | | | | | | | | | | | | | | | | | |  | |
|  |  | | | | | | | | | | | | | | | | | | |  | |
|  | **Codes\\THPP providers\Characteristics** | | | | | | | | | | | | | | | | | | |  | |
|  |  |  | No |  | 0.0467 |  | 1 |  | | | | | | | | | | | |  |  |
|  |  |  |  |  |  |  |  |  | | | | | | | | | | | | | |
|  | | | | | | | | | | 1 |  | | MTK | |  | | 8/3/2021 6:55 PM | |  | |  |
|  | I: Are there some behaviors of the counselor that you liked or there are some behaviors of the counselor that you did not like?  P: On that one, since this was the first time to have a counselor visiting me at home, I saw that everything was okay. She didn’t disappoint us, we accepted everything that came to us, sometimes she brought soap and that was okay. When I was not able to answer some questions in the sessions, I was able to tell her that I am not able to answer. Where I answered, she was appreciating that I have answered. | | | | | | | | | | | | | | | | | | |  | |
|  |  | | | | | | | | | | | | | | | | | | |  | |
|  | **Codes\\THPP providers\Relationship** | | | | | | | | | | | | | | | | | | |  | |
|  |  |  | No |  | 0.0406 |  | 1 |  | | | | | | | | | | | |  |  |
|  |  |  |  |  |  |  |  |  | | | | | | | | | | | | | |
|  | | | | | | | | | | 1 |  | | MTK | |  | | 8/3/2021 6:54 PM | |  | |  |
|  | I: Can you tell me about your relationship with the counselor who comes here?  P: We had a good relationship. When she comes, we do chat. She tells me what the session is about and we go through it and she goes. Even when we meet in the community, we greet each other because she stays close to me. When we meet at the funeral, we sit together and chat. At the market, Saturday is our market day. When we meet there we greet each other and invite each other home. | | | | | | | | | | | | | | | | | | |  | |
|  |  | | | | | | | | | | | | | | | | | | |  | |
|  |  |  |  |  |  |  |  |  |  |  |  |  |  |  |  |  |  |  |  |  |  |
|  |  |  |  |  |  |  |  |  |  |  |  |  |  |  |  |  |  |  |  |  |  |
| Formatted Reports\\Coding Summary by File Formatted Report | | | | | | | | | Page 43 of 145 | | | | | | | | | | | |  |
| 8/9/2021 8:08 AM | | | | | | | | | | | | | | | | | | | | |  |
|  | **Classification** |  | **Aggregate** |  | **Coverage** |  | **Number Of Coding References** |  | | **Reference Number** | |  | | **Coded By Initials** | |  | | **Modified On** | |  | |
|  | **Files\\IDIs\\IDI #10** | | | | | | | | | | | | | | | | | | |  | |
|  | **Code** | | | | | | | | | | | | | | | | | | |  | |
|  | **Codes\\Intervention experience** | | | | | | | | | | | | | | | | | | |  | |
|  |  |  | No |  | 0.1011 |  | 1 |  | | | | | | | | | | | |  |  |
|  |  |  |  |  |  |  |  |  | | | | | | | | | | | | | |
|  | | | | | | | | | | 1 |  | | MTK | |  | | 8/4/2021 1:01 PM | |  | |  |
|  | I: Thank you. What can you tell me about the counselling services you were receiving?  R: The counselling we were receiving was about helpful behaviors and unhelpful behaviors and also helpful thinking and unhelpful thinking.  I: So what can you tell me about the counselling you were getting?  R: This counselling was very good because it has helped me, at first, I had stress/depression but when I started receiving the counselling all the stress was gone because I realized that continued unhelpful thinking will not help me then I started helpful thinking as seen in the pictures that we were using where it was seen that unhelpful thinking was unhelpful indeed so I wanted to try helpful behavior to be helped and care for the child to grow healthy.  I: What was your main problem?  R: My problem was that I had stress thinking if I will have safe delivery, then after counselling sessions I was encouraged and helped. I was encouraged and delivered very well that when I knew that this counselling is good and promotes good health. | | | | | | | | | | | | | | | | | | |  | |
|  |  |  |  |  |  |  |  |  |  |  |  |  |  |  |  |  |  |  |  |  |  |
|  |  | | | | | | | | | | | | | | | | | | |  | |
|  | **Codes\\Intervention experience\Aspects** | | | | | | | | | | | | | | | | | | |  | |
|  |  |  | No |  | 0.1688 |  | 1 |  | | | | | | | | | | | |  |  |
|  |  |  |  |  |  |  |  |  | | | | | | | | | | | | | |
|  | | | | | | | | | | 1 |  | | MTK | |  | | 8/4/2021 1:02 PM | |  | |  |
|  | I: Thank you. What can you tell me about the counselling services you were receiving?  R: The counselling we were receiving was about helpful behaviors and unhelpful behaviors and also helpful thinking and unhelpful thinking.  I: So what can you tell me about the counselling you were getting?  R: This counselling was very good because it has helped me, at first, I had stress/depression but when I started receiving the counselling all the stress was gone because I realized that continued unhelpful thinking will not help me then I started helpful thinking as seen in the pictures that we were using where it was seen that unhelpful thinking was unhelpful indeed so I wanted to try helpful behavior to be helped and care for the child to grow healthy.  I: What was your main problem?  R: My problem was that I had stress thinking if I will have safe delivery, then after counselling sessions I was encouraged and helped. I was encouraged and delivered very well that when I knew that this counselling is good and promotes good health.  I: So you have told me helpful behavior and unhelpful behavior, can you tell me the helpful behavior which was used as a substitute for unhelpful behavior that was there?  R: The helpful behavior was that I was told that eating nutritious foods helps to produce enough breast milk for the baby while in unhelpful behavior you just stay alone stressed, not eating, not doing anything……I decided to eat nutritious foods so that the child is healthy, have energy for delivery and also charting with friends to avoid stress that is what I learnt in this program, they visited me since pregnancy till I gave birth while in unhelpful thinking you just stay alone without friends to help you in sharing ideas. | | | | | | | | | | | | | | | | | | |  | |
|  |  |  |  |  |  |  |  |  |  |  |  |  |  |  |  |  |  |  |  |  |  |
|  |  |  |  |  |  |  |  |  |  |  |  |  |  |  |  |  |  |  |  |  |  |
|  |  | | | | | | | | | | | | | | | | | | |  | |
|  | **Codes\\Intervention experience\Barriers** | | | | | | | | | | | | | | | | | | |  | |
|  |  |  | No |  | 0.0348 |  | 1 |  | | | | | | | | | | | |  |  |
|  |  |  |  |  |  |  |  |  | | | | | | | | | | | | | |
|  | | | | | | | | | | 1 |  | | MTK | |  | | 8/4/2021 1:04 PM | |  | |  |
|  | I: Ok thank you. Were there any challenges that made you not receive counselling services properly?  R: Services like what?  I: The same counselling service, was there anything that made you not get the required services.  R: There was none, when the counsellor started visiting me I found peace than it was before according to what they said at the beginning. | | | | | | | | | | | | | | | | | | |  | |
|  |  | | | | | | | | | | | | | | | | | | |  | |
|  |  |  |  |  |  |  |  |  |  |  |  |  |  |  |  |  |  |  |  |  |  |
| Formatted Reports\\Coding Summary by File Formatted Report | | | | | | | | | Page 44 of 145 | | | | | | | | | | | |  |
| 8/9/2021 8:08 AM | | | | | | | | | | | | | | | | | | | | |  |
|  | **Classification** |  | **Aggregate** |  | **Coverage** |  | **Number Of Coding References** |  | | **Reference Number** | |  | | **Coded By Initials** | |  | | **Modified On** | |  | |
|  | **Codes\\Intervention experience\Facilitators** | | | | | | | | | | | | | | | | | | |  | |
|  |  |  | No |  | 0.0883 |  | 2 |  | | | | | | | | | | | |  |  |
|  |  |  |  |  |  |  |  |  | | | | | | | | | | | | | |
|  | | | | | | | | | | 1 |  | | MTK | |  | | 8/4/2021 1:05 PM | |  | |  |
|  | I: So if there were no challenges what do you think made the counselling to go well?  R: For the counselling to go on well it was because of the counsellors who came to my house for counselling had it been they did not come my life would have been miserable and my life could not have changed as the way it is now. But because they were visiting me and giving me counseling that’s why my life changed | | | | | | | | | | | | | | | | | | |  | |
|  |  | | | | | | | | | | | | | | | | | | |  | |
|  | | | | | | | | | | 2 |  | | MTK | |  | | 8/4/2021 1:11 PM | |  | |  |
|  | I: So how do you think getting counselling at your home helped the counselling to go well?  R: This counselling helped me in that other family members were able to participate in the program. Other pregnant women in my village wanted to attend, I had a friend with stress I invited her and she complained that she wished she met you at the clinic to be enrolled in the program. Other people in the village were also interested to be part of the program.  I: What problems were there in getting counselling at home? | | | | | | | | | | | | | | | | | | |  | |
|  |  | | | | | | | | | | | | | | | | | | |  | |
|  | **Codes\\Intervention experience\Influence on others** | | | | | | | | | | | | | | | | | | |  | |
|  |  |  | No |  | 0.0459 |  | 2 |  | | | | | | | | | | | |  |  |
|  |  |  |  |  |  |  |  |  | | | | | | | | | | | | | |
|  | | | | | | | | | | 1 |  | | MTK | |  | | 8/4/2021 1:18 PM | |  | |  |
|  | I: Meaning that you can also help others?  R: Yes, I can help others, the way my counsellor (name withheld) was doing I would also do the same in helping my friends by following how she was doing with me. | | | | | | | | | | | | | | | | | | |  | |
|  |  | | | | | | | | | | | | | | | | | | |  | |
|  | | | | | | | | | | 2 |  | | MTK | |  | | 8/4/2021 1:19 PM | |  | |  |
|  | I: How are you going to use the skills gained in the program in your day to day life?  R: I will share some of the skills gained through the counselling with my friends so that they can also gain something. | | | | | | | | | | | | | | | | | | |  | |
|  |  | | | | | | | | | | | | | | | | | | |  | |
|  | **Codes\\Intervention experience\Likes** | | | | | | | | | | | | | | | | | | |  | |
|  |  |  | No |  | 0.1366 |  | 1 |  | | | | | | | | | | | |  |  |
|  |  |  |  |  |  |  |  |  | | | | | | | | | | | | | |
|  | | | | | | | | | | 1 |  | | MTK | |  | | 8/4/2021 1:04 PM | |  | |  |
|  | I: Ok looking at the counselling sessions you got what did you like most?  R: The most interesting part for me was on helpful behavior for good health, I liked that part most because you do not have hatred with friends …… and have a peaceful and happy life.  I: Ok sometimes you had time to think about what you learnt, which part helped you to reflect about what was discussed in counselling?  R: Like what I was taught during counselling on stress prevention, it all started when I was pregnant and I learnt that when I have a lot of worries, I don’t eat properly, that will make me weak and will have problems during delivery. I was encouraged that I should eat nutritious meals, chart with friends and have time for rest after doing household chores, that is why I had safe delivery after participating in the program. They continued visiting me after delivery up to now that I am okay ……..  I: Ok thank you very much. What did you like most about this counselling?  R: What I liked is that they helped in improving my health, I have good health and my child is healthy too. I live happily without getting angry with someone.  I: What part did not benefit you in the counselling?  R: I benefitted in all parts of counselling in this program because my life changed, I was in a bad place and they removed me and put me where I am now that I live happily. I thank this programme, it should continue because it is helpful. | | | | | | | | | | | | | | | | | | |  | |
|  |  |  |  |  |  |  |  |  |  |  |  |  |  |  |  |  |  |  |  |  |  |
|  |  |  |  |  |  |  |  |  |  |  |  |  |  |  |  |  |  |  |  |  |  |
|  |  | | | | | | | | | | | | | | | | | | |  | |
|  |  |  |  |  |  |  |  |  |  |  |  |  |  |  |  |  |  |  |  |  |  |
| Formatted Reports\\Coding Summary by File Formatted Report | | | | | | | | | Page 45 of 145 | | | | | | | | | | | |  |
| 8/9/2021 8:08 AM | | | | | | | | | | | | | | | | | | | | |  |
|  | **Classification** |  | **Aggregate** |  | **Coverage** |  | **Number Of Coding References** |  | | **Reference Number** | |  | | **Coded By Initials** | |  | | **Modified On** | |  | |
|  | **Codes\\Intervention process** | | | | | | | | | | | | | | | | | | |  | |
|  |  |  | No |  | 0.0397 |  | 1 |  | | | | | | | | | | | |  |  |
|  |  |  |  |  |  |  |  |  | | | | | | | | | | | | | |
|  | | | | | | | | | | 1 |  | | MTK | |  | | 8/4/2021 1:08 PM | |  | |  |
|  | I: You say the coming of the counsellor to you was helpful, what pleased you about this counsellor?  R: It was very good to see the counsellor teaching us. The counselor was following through the procedures the way she was trained when providing counseling to us unlike just doing the work haphazard. That helped me to learn a lot of things and improved my life.  I: So she was following the procedures?  R: Yes | | | | | | | | | | | | | | | | | | |  | |
|  |  | | | | | | | | | | | | | | | | | | |  | |
|  | **Codes\\Intervention process\Delivery** | | | | | | | | | | | | | | | | | | |  | |
|  |  |  | No |  | 0.0837 |  | 1 |  | | | | | | | | | | | |  |  |
|  |  |  |  |  |  |  |  |  | | | | | | | | | | | | | |
|  | | | | | | | | | | 1 |  | | MTK | |  | | 8/4/2021 1:08 PM | |  | |  |
|  | I: Can you tell me what made you feel good when visited during counselling?  R: What was more interesting is when they were teaching us with the pictures. The time we saw pictures of caring for children, one was taking care of the child and the other just left the child unattended without care. So I said if the child is not taken care of will s/ he grow healthy and I decide to follow the helpful behaviors, what the other woman was doing in caring for the child.  I: So the pictured helped you to take care of the child?  R: Yes I was so encouraged to try the helpful behavior to have a healthy baby.  I: So what can you describe the counselling through pictures?  R: The counseling through pictures is good because you see and differentiate the helpful and unhelpful behavior and decide what to follow unlike just hearing you hardly see the ways of caring a child. | | | | | | | | | | | | | | | | | | |  | |
|  |  |  |  |  |  |  |  |  |  |  |  |  |  |  |  |  |  |  |  |  |  |
|  |  | | | | | | | | | | | | | | | | | | |  | |
|  | **Codes\\Intervention process\Feelings** | | | | | | | | | | | | | | | | | | |  | |
|  |  |  | No |  | 0.1148 |  | 2 |  | | | | | | | | | | | |  |  |
|  |  |  |  |  |  |  |  |  | | | | | | | | | | | | | |
|  | | | | | | | | | | 1 |  | | MTK | |  | | 8/4/2021 1:07 PM | |  | |  |
|  | I: Can you tell me what made you feel good when visited during counselling?  R: What was more interesting is when they were teaching us with the pictures. The time we saw pictures of caring for children, one was taking care of the child and the other just left the child unattended without care. So I said if the child is not taken care of will s/ he grow healthy and I decide to follow the helpful behaviors, what the other woman was doing in caring for the child.  I: So the pictured helped you to take care of the child?  R: Yes I was so encouraged to try the helpful behavior to have a healthy baby. | | | | | | | | | | | | | | | | | | |  | |
|  |  | | | | | | | | | | | | | | | | | | |  | |
|  | | | | | | | | | | 2 |  | | MTK | |  | | 8/4/2021 1:13 PM | |  | |  |
|  | I: What problems were there in getting counselling at home?  R: There were no problems and nobody discouraged me in participating in the program.  I: Or other people disturbing you when getting counselling?  R: No, there was nothing only that other woman with babies would come asking to get the services so that they can benefit as well. So there were a lot of people who wanted to get the information.  I: How was this helpful to you?  R: This made me feel good. This program was seen to be helpful for our health and health of the baby. And I felt good because other people were involved. | | | | | | | | | | | | | | | | | | |  | |
|  |  |  |  |  |  |  |  |  |  |  |  |  |  |  |  |  |  |  |  |  |  |
|  |  | | | | | | | | | | | | | | | | | | |  | |
|  | **Codes\\Other interesting issues** | | | | | | | | | | | | | | | | | | |  | |
|  |  |  | No |  | 0.0588 |  | 2 |  | | | | | | | | | | | |  |  |
|  |  |  |  |  |  |  |  |  | | | | | | | | | | | | | |
|  | | | | | | | | | | 1 |  | | MTK | |  | | 8/4/2021 1:19 PM | |  | |  |
|  | I: Ok thank you, lastly is there anything you want to share concerning this program?  R: I would like to thank you for coming up with this idea, bringing the counselling program to help pregnant women. A lot of women have depression and some have miscarriages because of stress. But since the program started many have been helped to live happily and I ask you to continue so that others can also see the change. | | | | | | | | | | | | | | | | | | |  | |
|  |  | | | | | | | | | | | | | | | | | | |  | |
| Formatted Reports\\Coding Summary by File Formatted Report | | | | | | | | | Page 46 of 145 | | | | | | | | | | | |  |
| 8/9/2021 8:08 AM | | | | | | | | | | | | | | | | | | | | |  |
|  | **Classification** |  | **Aggregate** |  | **Coverage** |  | **Number Of Coding References** |  | | **Reference Number** | |  | | **Coded By Initials** | |  | | **Modified On** | |  | |
|  |  |  |  |  |  |  |  |  |  |  |  |  |  |  |  |  |  |  |  |  |  |
|  | | | | | | | | | | 2 |  | | MTK | |  | | 8/4/2021 1:19 PM | |  | |  |
|  | I: Thank you, do you have any questions?  R: I have no questions, am only thankful to the program because it has helped me out of the depression. But because of this program the depression is gone. | | | | | | | | | | | | | | | | | | |  | |
|  |  | | | | | | | | | | | | | | | | | | |  | |
|  | **Codes\\Participation of others** | | | | | | | | | | | | | | | | | | |  | |
|  |  |  | No |  | 0.0497 |  | 1 |  | | | | | | | | | | | |  |  |
|  |  |  |  |  |  |  |  |  | | | | | | | | | | | | | |
|  | | | | | | | | | | 1 |  | | MTK | |  | | 8/4/2021 1:12 PM | |  | |  |
|  | I: So how do you think getting counselling at your home helped the counselling to go well?  R: This counselling helped me in that other family members were able to participate in the program. Other pregnant women in my village wanted to attend, I had a friend with stress I invited her and she complained that she wished she met you at the clinic to be enrolled in the program. Other people in the village were also interested to be part of the program.  I: What problems were there in getting counselling at home? | | | | | | | | | | | | | | | | | | |  | |
|  |  | | | | | | | | | | | | | | | | | | |  | |
|  | **Codes\\Participation of others\Attitude** | | | | | | | | | | | | | | | | | | |  | |
|  |  |  | No |  | 0.0567 |  | 1 |  | | | | | | | | | | | |  |  |
|  |  |  |  |  |  |  |  |  | | | | | | | | | | | | | |
|  | | | | | | | | | | 1 |  | | MTK | |  | | 8/4/2021 1:12 PM | |  | |  |
|  | I: What problems were there in getting counselling at home?  R: There were no problems and nobody discouraged me in participating in the program.  I: Or other people disturbing you when getting counselling?  R: No, there was nothing only that other woman with babies would come asking to get the services so that they can benefit as well. So there were a lot of people who wanted to get the information.  I: How was this helpful to you?  R: This made me feel good. This program was seen to be helpful for our health and health of the baby. And I felt good because other people were involved. | | | | | | | | | | | | | | | | | | |  | |
|  |  |  |  |  |  |  |  |  |  |  |  |  |  |  |  |  |  |  |  |  |  |
|  |  | | | | | | | | | | | | | | | | | | |  | |
|  |  |  |  |  |  |  |  |  |  |  |  |  |  |  |  |  |  |  |  |  |  |
|  |  |  |  |  |  |  |  |  |  |  |  |  |  |  |  |  |  |  |  |  |  |
|  |  |  |  |  |  |  |  |  |  |  |  |  |  |  |  |  |  |  |  |  |  |
|  |  |  |  |  |  |  |  |  |  |  |  |  |  |  |  |  |  |  |  |  |  |
| Formatted Reports\\Coding Summary by File Formatted Report | | | | | | | | | Page 47 of 145 | | | | | | | | | | | |  |
| 8/9/2021 8:08 AM | | | | | | | | | | | | | | | | | | | | |  |
|  | **Classification** |  | **Aggregate** |  | **Coverage** |  | **Number Of Coding References** |  | | **Reference Number** | |  | | **Coded By Initials** | |  | | **Modified On** | |  | |
|  | **Codes\\Personal history** | | | | | | | | | | | | | | | | | | |  | |
|  |  |  | No |  | 0.0768 |  | 1 |  | | | | | | | | | | | |  |  |
|  |  |  |  |  |  |  |  |  | | | | | | | | | | | | | |
|  | | | | | | | | | | 1 |  | | MTK | |  | | 8/4/2021 1:00 PM | |  | |  |
|  | I: I am…. (Laughter) a counsellor in this study as I explained. So I would like to know how old are you?  R: I am 18  I: How far did you go with your education?  R: I did form two.  I: How is it in regards to marriage?  R: It is good and am married  I: How many times have you been pregnant?  R: This was my first time.  I: Thank you. Which village do you come from?  R: …….. village | | | | | | | | | | | | | | | | | | |  | |
|  |  |  |  |  |  |  |  |  |  |  |  |  |  |  |  |  |  |  |  |  |  |
|  |  |  |  |  |  |  |  |  |  |  |  |  |  |  |  |  |  |  |  |  |  |
|  |  |  |  |  |  |  |  |  |  |  |  |  |  |  |  |  |  |  |  |  |  |
|  |  | | | | | | | | | | | | | | | | | | |  | |
|  | **Codes\\Place of intervention delivery** | | | | | | | | | | | | | | | | | | |  | |
|  |  |  | No |  | 0.0402 |  | 1 |  | | | | | | | | | | | |  |  |
|  |  |  |  |  |  |  |  |  | | | | | | | | | | | | | |
|  | | | | | | | | | | 1 |  | | MTK | |  | | 8/4/2021 1:11 PM | |  | |  |
|  | I: I know that she visited you to at your home, what can you explain about the place for counselling?  R: The place for counselling was good for example the surrounding people wanted to know if the counselling was helpful or not, and the parents from both sides saw that the program was helpful because they saw that my life had improved. They encouraged me to continue so that I should not have stress in my heart. | | | | | | | | | | | | | | | | | | |  | |
|  |  | | | | | | | | | | | | | | | | | | |  | |
|  | **Codes\\Recommendations\General** | | | | | | | | | | | | | | | | | | |  | |
|  |  |  | No |  | 0.0526 |  | 1 |  | | | | | | | | | | | |  |  |
|  |  |  |  |  |  |  |  |  | | | | | | | | | | | | | |
|  | | | | | | | | | | 1 |  | | MTK | |  | | 8/4/2021 1:14 PM | |  | |  |
|  | I: You said this program is helpful and should continue, how do you think it should be provided in future?  R: If this counselling is to be provided to everyone, I would like to still be in the program to continue getting counselling and live healthy peaceful life, have peace of mind.  I: You said counselling helped you change from unhelpful to helpful behavior, what can you do that other people can benefit from this service as well?  R: If I can have books and other materials I could share with them so that they can be helped just as I did. | | | | | | | | | | | | | | | | | | |  | |
|  |  | | | | | | | | | | | | | | | | | | |  | |
|  |  |  |  |  |  |  |  |  |  |  |  |  |  |  |  |  |  |  |  |  |  |
| Formatted Reports\\Coding Summary by File Formatted Report | | | | | | | | | Page 48 of 145 | | | | | | | | | | | |  |
| 8/9/2021 8:08 AM | | | | | | | | | | | | | | | | | | | | |  |
|  | **Classification** |  | **Aggregate** |  | **Coverage** |  | **Number Of Coding References** |  | | **Reference Number** | |  | | **Coded By Initials** | |  | | **Modified On** | |  | |
|  | **Codes\\THPP providers** | | | | | | | | | | | | | | | | | | |  | |
|  |  |  | No |  | 0.0586 |  | 2 |  | | | | | | | | | | | |  |  |
|  |  |  |  |  |  |  |  |  | | | | | | | | | | | | | |
|  | | | | | | | | | | 1 |  | | MTK | |  | | 8/4/2021 1:09 PM | |  | |  |
|  | I: You say the coming of the counsellor to you was helpful, what pleased you about this counsellor?  R: It was very good to see the counsellor teaching us. The counselor was following through the procedures the way she was trained when providing counseling to us unlike just doing the work haphazard. That helped me to learn a lot of things and improved my life.  I: So she was following the procedures?  R: Yes | | | | | | | | | | | | | | | | | | |  | |
|  |  | | | | | | | | | | | | | | | | | | |  | |
|  | | | | | | | | | | 2 |  | | MTK | |  | | 8/4/2021 1:09 PM | |  | |  |
|  | I: What did you not like about your counsellor?  R: Frankly speaking there is nothing (Laughter)  I: (Laughter) she did nothing wrong?  R: Yes from the start to the end I had no problems with her. | | | | | | | | | | | | | | | | | | |  | |
|  |  | | | | | | | | | | | | | | | | | | |  | |
|  | **Codes\\THPP providers\Characteristics** | | | | | | | | | | | | | | | | | | |  | |
|  |  |  | No |  | 0.0159 |  | 1 |  | | | | | | | | | | | |  |  |
|  |  |  |  |  |  |  |  |  | | | | | | | | | | | | | |
|  | | | | | | | | | | 1 |  | | MTK | |  | | 8/4/2021 1:09 PM | |  | |  |
|  | I: How can you explain their behavior?  R: She is good, cheerful and not getting angry when teaching. When asked she answered frankly because she wanted us to know. | | | | | | | | | | | | | | | | | | |  | |
|  |  | | | | | | | | | | | | | | | | | | |  | |
|  | **Codes\\THPP providers\Relationship** | | | | | | | | | | | | | | | | | | |  | |
|  |  |  | No |  | 0.0473 |  | 1 |  | | | | | | | | | | | |  |  |
|  |  |  |  |  |  |  |  |  | | | | | | | | | | | | | |
|  | | | | | | | | | | 1 |  | | MTK | |  | | 8/4/2021 1:10 PM | |  | |  |
|  | I: How was your cooperation?  R: Very good, she visits me even now, plays with the child, we chart and she goes.  I: She comes for counselling or…….?  R: After the counselling, just out of her good will, she visits me.  I: Ok so you were like friends, how did you feel about this?  R: I felt good because after the last assessment, when we talked on the phone, she continued visiting me. If it was someone else, they would have just said the since we have terminated the visits then that’s it. | | | | | | | | | | | | | | | | | | |  | |
|  |  |  |  |  |  |  |  |  |  |  |  |  |  |  |  |  |  |  |  |  |  |
|  |  | | | | | | | | | | | | | | | | | | |  | |
|  |  |  |  |  |  |  |  |  |  |  |  |  |  |  |  |  |  |  |  |  |  |
|  |  |  |  |  |  |  |  |  |  |  |  |  |  |  |  |  |  |  |  |  |  |
|  |  |  |  |  |  |  |  |  |  |  |  |  |  |  |  |  |  |  |  |  |  |
| Formatted Reports\\Coding Summary by File Formatted Report | | | | | | | | | Page 49 of 145 | | | | | | | | | | | |  |
| 8/9/2021 8:08 AM | | | | | | | | | | | | | | | | | | | | |  |
|  | **Classification** |  | **Aggregate** |  | **Coverage** |  | **Number Of Coding References** |  | | **Reference Number** | |  | | **Coded By Initials** | |  | | **Modified On** | |  | |
|  | **Files\\IDIs\\IDI #11** | | | | | | | | | | | | | | | | | | |  | |
|  | **Code** | | | | | | | | | | | | | | | | | | |  | |
|  | **Codes\\Intervention experience** | | | | | | | | | | | | | | | | | | |  | |
|  |  |  | No |  | 0.0866 |  | 3 |  | | | | | | | | | | | |  |  |
|  |  |  |  |  |  |  |  |  | | | | | | | | | | | | | |
|  | | | | | | | | | | 1 |  | | MTK | |  | | 8/4/2021 2:09 PM | |  | |  |
|  | I: Thank you, so can you tell me about the counselling you have been receiving  P: In the counselling I have learnt a lot of things firstly eating the six food groups which I never knew and breast feeding a child that we should breast feed the baby exclusively and also we should be mixing/charting with relatives and allow them to help take care of the child, this is what I have learnt  I: What are the other things apart from these?  P: I have been assisted to learn relaxation and have helpful thoughts, because if you have unhelpful ideas and thinking too much you may have a high blood pressure, people will just hear that you have run mad/developed mental illness or high blood pressure and have died leaving a small baby…… this counselling has help us reason properly. | | | | | | | | | | | | | | | | | | |  | |
|  |  |  |  |  |  |  |  |  |  |  |  |  |  |  |  |  |  |  |  |  |  |
|  |  | | | | | | | | | | | | | | | | | | |  | |
|  | | | | | | | | | | 2 |  | | MTK | |  | | 8/4/2021 2:53 PM | |  | |  |
|  | I: how did it not help you?  P: there is no part which did not help me, because every bad thoughts that I had has now changed. | | | | | | | | | | | | | | | | | | |  | |
|  |  | | | | | | | | | | | | | | | | | | |  | |
|  | | | | | | | | | | 3 |  | | MTK | |  | | 8/4/2021 2:55 PM | |  | |  |
|  | I: do you have any question or other things you would like to say regarding what we have discussed today?  P: no, I don’t have questions but just a comment that I am thankful because I have known a lot of things and my life has changed because of this programme. | | | | | | | | | | | | | | | | | | |  | |
|  |  | | | | | | | | | | | | | | | | | | |  | |
|  | **Codes\\Intervention experience\Aspects** | | | | | | | | | | | | | | | | | | |  | |
|  |  |  | No |  | 0.1354 |  | 2 |  | | | | | | | | | | | |  |  |
|  |  |  |  |  |  |  |  |  | | | | | | | | | | | | | |
|  | | | | | | | | | | 1 |  | | MTK | |  | | 8/4/2021 2:10 PM | |  | |  |
|  | I: So, which part of counselling has helped you a lot?  P: Helpful thinking and also eating the six food groups for good health of me and the baby.  I: How has this helped you?  P: I have changed the way I was thinking. I was thinking of very bad things that I should just kill myself or terminate the pregnancy because of what this man was doing to me… Now after receiving the counselling everything has changed as I have changed my way of thinking and learnt that in any misunderstandings we have sit down for dialogue.  I: Mmm okay thank you very much, so you see that the way you were helped in thinking has changed that you now think in a helpful way?  P: Yes  I: What is the other part that excited you?  P: Breastfeeding the child exclusively, because when the child’s bones are growing, exclusive breastfeeding helps the child to grow healthy and energetic and looks good which makes me happy as a mother that my child is growing healthy | | | | | | | | | | | | | | | | | | |  | |
|  |  |  |  |  |  |  |  |  |  |  |  |  |  |  |  |  |  |  |  |  |  |
|  |  | | | | | | | | | | | | | | | | | | |  | |
|  | | | | | | | | | | 2 |  | | MTK | |  | | 8/4/2021 2:25 PM | |  | |  |
|  | I: So sometimes when you were receiving counselling you were supposed to take time and reflect on what you do in your life, which areas helped you to do this?  P: Changing the way of thinking helped me a lot. I say to myself that the study team has helped me a lot regarding my life because I was thinking of bad things before [thinking of harming self] without this counseling I would have been dead by now, so the counselling has helped me to think wisely, calmly, and find a real answer in my head.  I: Mmmh, so which part of counselling helped you to be reflecting on your thoughts?  P: Helpful thinking, because when you have a helpful thinking it helps you think on how to solve your problems even when you are angry then you see yourself happy again and you realize that you have been helped and helped yourself. .  I: So you are saying that helpful thinking has helped you also to start thinking in a helpful way?  P: Yes | | | | | | | | | | | | | | | | | | |  | |
|  |  |  |  |  |  |  |  |  |  |  |  |  |  |  |  |  |  |  |  |  |  |
|  |  | | | | | | | | | | | | | | | | | | |  | |
| Formatted Reports\\Coding Summary by File Formatted Report | | | | | | | | | Page 50 of 145 | | | | | | | | | | | |  |
| 8/9/2021 8:08 AM | | | | | | | | | | | | | | | | | | | | |  |
|  | **Classification** |  | **Aggregate** |  | **Coverage** |  | **Number Of Coding References** |  | | **Reference Number** | |  | | **Coded By Initials** | |  | | **Modified On** | |  | |
|  | **Codes\\Intervention experience\Barriers** | | | | | | | | | | | | | | | | | | |  | |
|  |  |  | No |  | 0.0415 |  | 1 |  | | | | | | | | | | | |  |  |
|  |  |  |  |  |  |  |  |  | | | | | | | | | | | | | |
|  | | | | | | | | | | 1 |  | | MTK | |  | | 8/4/2021 2:26 PM | |  | |  |
|  | I: Okay thank you very much, what are the challenges that were there during counselling which made you not to receive the counselling in the way you were supposed to get? For this counselling was given at different days, and on those days if there were any problems?  P: On the days that we were meeting, there was no day that we failed to meet, we had meetings as scheduled, and there was also no problem that made us unable to get counselling, all the days we were meeting.  I: Mmmmh (paraphrasing…..)So you we meeting all the days  P: Yes | | | | | | | | | | | | | | | | | | |  | |
|  |  |  |  |  |  |  |  |  |  |  |  |  |  |  |  |  |  |  |  |  |  |
|  |  | | | | | | | | | | | | | | | | | | |  | |
|  | **Codes\\Intervention experience\Facilitators** | | | | | | | | | | | | | | | | | | |  | |
|  |  |  | No |  | 0.0567 |  | 1 |  | | | | | | | | | | | |  |  |
|  |  |  |  |  |  |  |  |  | | | | | | | | | | | | | |
|  | | | | | | | | | | 1 |  | | MTK | |  | | 8/4/2021 2:27 PM | |  | |  |
|  | I: What helped in making it possible to meet all the days without any problem?  P: Mainly I put myself in God’s hands and pray that on the day I should be strong, not sick and that nothing should happen on that day apart from being in the counseling session and by His grace this was happening without any disturbances.  I: So you see that prayers helped you?  P: Yes  I: Apart from prayers what is the other thing that helped you?  P: The counsellors were also very dedicated who knew that the person we are visiting needs our help and we should not fail to meet her so that she should not say that we are not serious, so the counsellors had a passion for visiting us.  P: So, you are saying that the dedication of the counsellors helped the counselling to be done as required?  P: Yes | | | | | | | | | | | | | | | | | | |  | |
|  |  |  |  |  |  |  |  |  |  |  |  |  |  |  |  |  |  |  |  |  |  |
|  |  | | | | | | | | | | | | | | | | | | |  | |
|  | **Codes\\Intervention experience\Influence on others** | | | | | | | | | | | | | | | | | | |  | |
|  |  |  | No |  | 0.0408 |  | 1 |  | | | | | | | | | | | |  |  |
|  |  |  |  |  |  |  |  |  | | | | | | | | | | | | | |
|  | | | | | | | | | | 1 |  | | MTK | |  | | 8/4/2021 2:54 PM | |  | |  |
|  | I: what can you tell others about this counselling?  P: I can tell them not to ignore health services or projects by other organizations, they should welcome these things with a positive mind and have the passion to learn. They should give correct information about the name and address and not lie by giving false information.  I: if you can be asked, would you like other people to be receiving this counselling?  P: yes  I: why  P: because I have been motivated leaving my old thinking I had to start helpfully thinking and I would like to see others helped too | | | | | | | | | | | | | | | | | | |  | |
|  |  |  |  |  |  |  |  |  |  |  |  |  |  |  |  |  |  |  |  |  |  |
|  |  | | | | | | | | | | | | | | | | | | |  | |
|  | **Codes\\Intervention experience\Likes** | | | | | | | | | | | | | | | | | | |  | |
|  |  |  | No |  | 0.0611 |  | 2 |  | | | | | | | | | | | |  |  |
|  |  |  |  |  |  |  |  |  | | | | | | | | | | | | | |
|  | | | | | | | | | | 1 |  | | MTK | |  | | 8/4/2021 2:24 PM | |  | |  |
|  | I: Okay…. So in every good things there are bad things as well, what are the things that did not please you?  P: Aaah on these discussions there is nothing that I feel did not please me or it was not good for me because at first, I receive this counseling very well. I was not mixing with others, but now I have changed, I chart with relatives, I have started giving them the baby, so they can assist in taking care of the baby and assist in doing some work while I rest. Now everything is good to me. | | | | | | | | | | | | | | | | | | |  | |
|  |  | | | | | | | | | | | | | | | | | | |  | |
|  |  |  |  |  |  |  |  |  |  |  |  |  |  |  |  |  |  |  |  |  |  |
| Formatted Reports\\Coding Summary by File Formatted Report | | | | | | | | | Page 51 of 145 | | | | | | | | | | | |  |
| 8/9/2021 8:08 AM | | | | | | | | | | | | | | | | | | | | |  |
|  | **Classification** |  | **Aggregate** |  | **Coverage** |  | **Number Of Coding References** |  | | **Reference Number** | |  | | **Coded By Initials** | |  | | **Modified On** | |  | |
|  |  |  |  |  |  |  |  |  |  |  |  |  |  |  |  |  |  |  |  |  |  |
|  | | | | | | | | | | 2 |  | | MTK | |  | | 8/4/2021 2:25 PM | |  | |  |
|  | I: So which part of counselling did not please you?  P: Mmmh frankly speaking there is no part of counselling that I disliked, I received everything well because it gave me courage in my life, that I should have a healthy life and a helpful thinking so that my child should be healthy and grow without stress like I used to be, I don’t want that. | | | | | | | | | | | | | | | | | | |  | |
|  |  | | | | | | | | | | | | | | | | | | |  | |
|  | **Codes\\Intervention process\Delivery** | | | | | | | | | | | | | | | | | | |  | |
|  |  |  | No |  | 0.0673 |  | 2 |  | | | | | | | | | | | |  |  |
|  |  |  |  |  |  |  |  |  | | | | | | | | | | | | | |
|  | | | | | | | | | | 1 |  | | MTK | |  | | 8/4/2021 2:31 PM | |  | |  |
|  | I: What were the things that did not please you?  P: There is nothing that I did not like  I: What made you feel there was nothing you disliked?  P: When counsellors came to use, they were following their guide book and the lessons they got from the senior counsellors who taught them what they knew. | | | | | | | | | | | | | | | | | | |  | |
|  |  | | | | | | | | | | | | | | | | | | |  | |
|  | | | | | | | | | | 2 |  | | MTK | |  | | 8/4/2021 2:32 PM | |  | |  |
|  | I: Were there things that somehow did not please you? Remember this is confidential between you and me.  P: What I did not like very much in terms of teaching is that she could read well but no clarification, I had a problem interpreting some terms used, since no clarification was given. So I just tried to figure out what the meaning on my own.  I: Mmmh meaning what?  P: Was just reading from the books without clarifications/interpretation which made me not understand some of the things. And I was taking a long time to realize/understand what she meant | | | | | | | | | | | | | | | | | | |  | |
|  |  |  |  |  |  |  |  |  |  |  |  |  |  |  |  |  |  |  |  |  |  |
|  |  | | | | | | | | | | | | | | | | | | |  | |
|  | **Codes\\Intervention process\Feelings** | | | | | | | | | | | | | | | | | | |  | |
|  |  |  | No |  | 0.0430 |  | 1 |  | | | | | | | | | | | |  |  |
|  |  |  |  |  |  |  |  |  | | | | | | | | | | | | | |
|  | | | | | | | | | | 1 |  | | MTK | |  | | 8/4/2021 2:52 PM | |  | |  |
|  | I: thank you very much, so what can you explain about this type of counselling? And also how are you feeling in your heart?  P: I can say that it has changed my way of thinking than previously like the thoughts of terminating the pregnancy or leaving my husband and going back to my home village because of what my husband was doing to me.  I: yes, yes …  P: but after counselling I have nothing to worry about, if I have a problem, I look for the solution and eventually it is solved  I: mmmh okay so you see how the counselling has helped you?  P: it has helped me to think reasonably and fast | | | | | | | | | | | | | | | | | | |  | |
|  |  |  |  |  |  |  |  |  |  |  |  |  |  |  |  |  |  |  |  |  |  |
|  |  | | | | | | | | | | | | | | | | | | |  | |
|  |  |  |  |  |  |  |  |  |  |  |  |  |  |  |  |  |  |  |  |  |  |
|  |  |  |  |  |  |  |  |  |  |  |  |  |  |  |  |  |  |  |  |  |  |
|  |  |  |  |  |  |  |  |  |  |  |  |  |  |  |  |  |  |  |  |  |  |
| Formatted Reports\\Coding Summary by File Formatted Report | | | | | | | | | Page 52 of 145 | | | | | | | | | | | |  |
| 8/9/2021 8:08 AM | | | | | | | | | | | | | | | | | | | | |  |
|  | **Classification** |  | **Aggregate** |  | **Coverage** |  | **Number Of Coding References** |  | | **Reference Number** | |  | | **Coded By Initials** | |  | | **Modified On** | |  | |
|  | **Codes\\Personal history** | | | | | | | | | | | | | | | | | | |  | |
|  |  |  | No |  | 0.0555 |  | 1 |  | | | | | | | | | | | |  |  |
|  |  |  |  |  |  |  |  |  | | | | | | | | | | | | | |
|  | | | | | | | | | | 1 |  | | MTK | |  | | 8/4/2021 2:09 PM | |  | |  |
|  | I: So, at the end of this study we would like to know about your thoughts of what you went through while receiving that counselling, but firstly I would like to know how old are you?  P: 20  I: How far did you go with your education?  P: Form four  I: Are you married?  P: Yes  I: How many times have you been pregnant?  P: It’s the first one  I: Okay, so you have never given birth before?  P: Yes, it’s my first time  I: Okay, where do you come from?  P: …………. village | | | | | | | | | | | | | | | | | | |  | |
|  |  |  |  |  |  |  |  |  |  |  |  |  |  |  |  |  |  |  |  |  |  |
|  |  |  |  |  |  |  |  |  |  |  |  |  |  |  |  |  |  |  |  |  |  |
|  |  |  |  |  |  |  |  |  |  |  |  |  |  |  |  |  |  |  |  |  |  |
|  |  | | | | | | | | | | | | | | | | | | |  | |
|  | **Codes\\Place of intervention delivery** | | | | | | | | | | | | | | | | | | |  | |
|  |  |  | No |  | 0.1329 |  | 2 |  | | | | | | | | | | | |  |  |
|  |  |  |  |  |  |  |  |  | | | | | | | | | | | | | |
|  | | | | | | | | | | 1 |  | | MTK | |  | | 8/4/2021 2:50 PM | |  | |  |
|  | I: Mmh what about the place where you were getting counselling, how was it?  P: Aaah the place was private, we were only two, me and the counsellor meeting inside the house  I: How did this help the counselling to move well?  P: About the place?  P: We were meeting in the house, a private place, that people could not know what was being discussed, they were just asking what the counsellor does when she comes to my home and why did she choose me alone yet we are so many in the village, and I told them that I was just lucky.it helped to maintain confidentiality so that others could not know about our discussions. | | | | | | | | | | | | | | | | | | |  | |
|  |  |  |  |  |  |  |  |  |  |  |  |  |  |  |  |  |  |  |  |  |  |
|  |  | | | | | | | | | | | | | | | | | | |  | |
|  | | | | | | | | | | 2 |  | | MTK | |  | | 8/4/2021 2:52 PM | |  | |  |
|  | I: what were the main problem concerning place  P: there was no problem with place because when the counsellor comes, we could sit on a mat inside the house.  I: what advice can you give in regards to place?  P: on place, what is needed is that when the counsellor comes should be welcomed and given place inside the house not outside because counselling issues are confidential  I: okay so on your side you see that it was good because you are the one who organized it, and that you have freedom of preparing a place, helped you so much, if we can imagine that in the future the government of Malawi has started giving counselling to people who have depression problems in the country, on place what can be the best place for counselling?  P: If there can be a place where we could meet but not in a large group, the way we are doing here but find a special place and build a structure not in a village.  I: why not in a village?  P: To avoid some discouragements from others if done in the village, but when it is outside the village you can just walk out without others knowing where you are going. Because sometimes people discourage you that you are just wasting your time and they doubt the usefulness of the programme. | | | | | | | | | | | | | | | | | | |  | |
|  |  |  |  |  |  |  |  |  |  |  |  |  |  |  |  |  |  |  |  |  |  |
|  |  |  |  |  |  |  |  |  |  |  |  |  |  |  |  |  |  |  |  |  |  |
|  |  | | | | | | | | | | | | | | | | | | |  | |
|  |  |  |  |  |  |  |  |  |  |  |  |  |  |  |  |  |  |  |  |  |  |
| Formatted Reports\\Coding Summary by File Formatted Report | | | | | | | | | Page 53 of 145 | | | | | | | | | | | |  |
| 8/9/2021 8:08 AM | | | | | | | | | | | | | | | | | | | | |  |
|  | **Classification** |  | **Aggregate** |  | **Coverage** |  | **Number Of Coding References** |  | | **Reference Number** | |  | | **Coded By Initials** | |  | | **Modified On** | |  | |
|  | **Codes\\Recommendations\Intervention providers** | | | | | | | | | | | | | | | | | | |  | |
|  |  |  | No |  | 0.0684 |  | 1 |  | | | | | | | | | | | |  |  |
|  |  |  |  |  |  |  |  |  | | | | | | | | | | | | | |
|  | | | | | | | | | | 1 |  | | MTK | |  | | 8/4/2021 2:43 PM | |  | |  |
|  | I: Okay, so to avoid this happening again in future< what advice can you give?  P: To the counsellor or who?  I: To us who would want that this type of counselling be given again in future? So that when teaching others we can look into that.  P: I think the solution should be recruiting people who are well educated and able to read properly, not somebody who have just reached standard 7 or 4, because it becomes difficult to get the meaning of what they are reading to you instead you just guess, that this is what they are trying to say. So select people who have gone far with their education  I: Mmmh in case someone is not educated, what can happen if they are the ones giving counselling?  P: These counsellors?  I: Yes  P: Then it is going to be a problem if someone does not know how to read, may be if she can find someone to be reading for her, or the counsellors to be reading and interpreting, this is what uneducated person can do. | | | | | | | | | | | | | | | | | | |  | |
|  |  |  |  |  |  |  |  |  |  |  |  |  |  |  |  |  |  |  |  |  |  |
|  |  | | | | | | | | | | | | | | | | | | |  | |
|  | **Codes\\THPP providers** | | | | | | | | | | | | | | | | | | |  | |
|  |  |  | No |  | 0.0919 |  | 2 |  | | | | | | | | | | | |  |  |
|  |  |  |  |  |  |  |  |  | | | | | | | | | | | | | |
|  | | | | | | | | | | 1 |  | | MTK | |  | | 8/4/2021 2:28 PM | |  | |  |
|  | I: Mmh okay thank you very much, so if you look at the counsellors who have been visiting you, how can you describe them?  P: The counsellors were doing helpful things because as I have already said they have changed my life from where I was, I am now a changed person in the way of thinking and the counsellors have done a great job and cannot say anything bad about the counsellors, they have helped me a lot and I am thankful.  I:Our discussions are confidential, whatever we are going to discuss here nobody will know it even your counsellors, so I would like you to tell me what pleased you on what the counsellors were doing  P: In terms of what?  I: Of what the counsellors were doing what was good for you?  P: The way they were teaching us and the other time they were bringing soap for us, these were good for us. | | | | | | | | | | | | | | | | | | |  | |
|  |  |  |  |  |  |  |  |  |  |  |  |  |  |  |  |  |  |  |  |  |  |
|  |  | | | | | | | | | | | | | | | | | | |  | |
|  | | | | | | | | | | 2 |  | | MTK | |  | | 8/4/2021 2:51 PM | |  | |  |
|  | I: Okay, you have said talked about some problems like clarification of some things during counselling, so on the place for counselling, was there any problem?  P: in terms of place, it was alright, the only problem I had with counselling is interpreting of some words which I could not really understand because I could just try to figure out on my own and this did not touch my heart, but for the place was good. | | | | | | | | | | | | | | | | | | |  | |
|  |  | | | | | | | | | | | | | | | | | | |  | |
|  | **Codes\\THPP providers\Characteristics** | | | | | | | | | | | | | | | | | | |  | |
|  |  |  | No |  | 0.0341 |  | 1 |  | | | | | | | | | | | |  |  |
|  |  |  |  |  |  |  |  |  | | | | | | | | | | | | | |
|  | | | | | | | | | | 1 |  | | MTK | |  | | 8/4/2021 2:31 PM | |  | |  |
|  | I: What are the good behavior of your counsellors, are any behaviors that you can say were good according to you?  P: They were always happy when they come to us and we could chart, they also had a passion of teaching this made me feel happy with them. They were dedicated not pompus.  I: Is there another thing?  P: The other thing I can say is that the times they gave me soap as a gift I was very happy and she was happy too, that you our senior counselors sent us gifts | | | | | | | | | | | | | | | | | | |  | |
|  |  | | | | | | | | | | | | | | | | | | |  | |
|  |  |  |  |  |  |  |  |  |  |  |  |  |  |  |  |  |  |  |  |  |  |
| Formatted Reports\\Coding Summary by File Formatted Report | | | | | | | | | Page 54 of 145 | | | | | | | | | | | |  |
| 8/9/2021 8:08 AM | | | | | | | | | | | | | | | | | | | | |  |
|  | **Classification** |  | **Aggregate** |  | **Coverage** |  | **Number Of Coding References** |  | | **Reference Number** | |  | | **Coded By Initials** | |  | | **Modified On** | |  | |
|  | **Codes\\THPP providers\Relationship** | | | | | | | | | | | | | | | | | | |  | |
|  |  |  | No |  | 0.0241 |  | 1 |  | | | | | | | | | | | |  |  |
|  |  |  |  |  |  |  |  |  | | | | | | | | | | | | | |
|  | | | | | | | | | | 1 |  | | MTK | |  | | 8/4/2021 2:43 PM | |  | |  |
|  | I: Mmh but how was your relation with your counsellor, the way you see?  P: There was a great relationship because there was no day, we missed our meeting and we could agree about time with much emphasis, seeing the benefits of the counselling sessions to me. This made me attend the sessions as scheduled, there was good relationship. | | | | | | | | | | | | | | | | | | |  | |
|  |  | | | | | | | | | | | | | | | | | | |  | |
|  | **Files\\IDIs\\IDI #12** | | | | | | | | | | | | | | | | | | |  | |
|  | **Code** | | | | | | | | | | | | | | | | | | |  | |
|  | **Codes\\Intervention experience** | | | | | | | | | | | | | | | | | | |  | |
|  |  |  | No |  | 0.2194 |  | 2 |  | | | | | | | | | | | |  |  |
|  |  |  |  |  |  |  |  |  | | | | | | | | | | | | | |
|  | | | | | | | | | | 1 |  | | MTK | |  | | 8/4/2021 3:04 PM | |  | |  |
|  | I: Fine. Thank you very much. Now I would like us to go to another section of our discussion. In this section, I would like to know about the study in which you were taking part. Firstly, before you joined the study, you were screened for depression and you scored high and that was why you were eligible to take part in this study. So, as part of the treatment, you were supposed to receive counseling intervention in order to help you deal with the depression. So can you tell me more about the counseling intervention sessions?  R: It went on very well because we were answering them freely based on how she had asked us the questions. Sure.  I: What areas of the counseling intervention went well?  R: When she was asking me “how are things going?” I was answering her “everything is going on well.” She was telling me that “it shows that you are no longer thinking too much now”.  I: Okay. The counselor was coming to your home to provide you with the counseling. What have you benefitted from the counseling intervention that you received?  R: From the beginning to the end, I realized that it is good to have people like her to be visiting me because I learned a lot of things. | | | | | | | | | | | | | | | | | | |  | |
|  |  |  |  |  |  |  |  |  |  |  |  |  |  |  |  |  |  |  |  |  |  |
|  |  | | | | | | | | | | | | | | | | | | |  | |
|  | | | | | | | | | | 2 |  | | MTK | |  | | 8/4/2021 3:23 PM | |  | |  |
|  | I: Okay. So we are going towards the end of our discussion but I would like to ask you; how do you perceive this counseling? I will seem like repeating the same question, what differences have you noted in your life?  R: Counseling is helpful because I am no longer thinking too much.  I: Meaning that the study has helped you to be free from stress?  R: Yes because I stopped having unhelpful behavior and I have helpful behavior now.  I: And you think you can sustain it?  R: I will now be focusing on the helpful behavior.  I: Okay. I understand that the counselor no longer visits you at home…  R: Yes she stopped.  I: Problems are always there in life right?  R: Yes.  I: Can you go back to your unhelpful behavior, in case you meet problems?  R: I cannot.  I: Why?  R: Because having learnt helpful behaviors like mixing with others, so when you share your problems with your friends or family members, they are able to help you. | | | | | | | | | | | | | | | | | | |  | |
|  |  |  |  |  |  |  |  |  |  |  |  |  |  |  |  |  |  |  |  |  |  |
|  |  |  |  |  |  |  |  |  |  |  |  |  |  |  |  |  |  |  |  |  |  |
|  |  |  |  |  |  |  |  |  |  |  |  |  |  |  |  |  |  |  |  |  |  |
|  |  | | | | | | | | | | | | | | | | | | |  | |
| Formatted Reports\\Coding Summary by File Formatted Report | | | | | | | | | Page 55 of 145 | | | | | | | | | | | |  |
| 8/9/2021 8:08 AM | | | | | | | | | | | | | | | | | | | | |  |
|  | **Classification** |  | **Aggregate** |  | **Coverage** |  | **Number Of Coding References** |  | | **Reference Number** | |  | | **Coded By Initials** | |  | | **Modified On** | |  | |
|  | **Codes\\Intervention experience\Facilitators** | | | | | | | | | | | | | | | | | | |  | |
|  |  |  | No |  | 0.0873 |  | 2 |  | | | | | | | | | | | |  |  |
|  |  |  |  |  |  |  |  |  | | | | | | | | | | | | | |
|  | | | | | | | | | | 1 |  | | MTK | |  | | 8/4/2021 3:06 PM | |  | |  |
|  | I: Okay. What do you think made the counseling to go on well?  R: Because we accepted that the counselor should be coming and we were having sessions for a long time.  I: So you liked the aspect that the counselor was visiting you at your home?  R: Yes and that was very prestigious.  I: The fact that she was coming, it was prestigious to you?  R: Yes. | | | | | | | | | | | | | | | | | | |  | |
|  |  | | | | | | | | | | | | | | | | | | |  | |
|  | | | | | | | | | | 2 |  | | MTK | |  | | 8/4/2021 3:12 PM | |  | |  |
|  | I: How can you describe your relationship with your counselor?  R: It was a good relationship because we were free and open with each other.  I: Was she new to you or you knew her before?  R: I knew her from [Name of village].  I: Did you note any change in her from the way you knew her before?  R: I did not know that she provides counseling to people in the community.  I: I thank you so much for telling me that you had a good relationship with the counselor and that made you to benefit from the counseling…  R: Yes.  I: Can we say that you benefitted because the counselor was open and cheerful?  R: Yes. | | | | | | | | | | | | | | | | | | |  | |
|  |  |  |  |  |  |  |  |  |  |  |  |  |  |  |  |  |  |  |  |  |  |
|  |  | | | | | | | | | | | | | | | | | | |  | |
|  |  | | | | | | | | | | | | | | | | | | |  | |
|  | **Codes\\Intervention experience\Likes** | | | | | | | | | | | | | | | | | | |  | |
|  |  |  | No |  | 0.0586 |  | 1 |  | | | | | | | | | | | |  |  |
|  |  |  |  |  |  |  |  |  | | | | | | | | | | | | | |
|  | | | | | | | | | | 1 |  | | MTK | |  | | 8/4/2021 3:05 PM | |  | |  |
|  | I: What was it that you liked the most about the counseling?  R: I liked everything that she was doing to me because she was also giving me soap.  I: Apart from the soap that you were given, there were several sessions that you had…  R: Yes.  I: What was it that you liked the most about the counseling intervention?  R: I likes three things that I learned and I realized that if I can integrate what I learned in my life, it can be very helpful.  I: You said you learned how many things?  R: Three things.  I: What were they?  R: Health of the woman… I have forgotten the others.  I: You simply remember that you liked the three aspects?  R: Yes. | | | | | | | | | | | | | | | | | | |  | |
|  |  |  |  |  |  |  |  |  |  |  |  |  |  |  |  |  |  |  |  |  |  |
|  |  | | | | | | | | | | | | | | | | | | |  | |
|  |  |  |  |  |  |  |  |  |  |  |  |  |  |  |  |  |  |  |  |  |  |
| Formatted Reports\\Coding Summary by File Formatted Report | | | | | | | | | Page 56 of 145 | | | | | | | | | | | |  |
| 8/9/2021 8:08 AM | | | | | | | | | | | | | | | | | | | | |  |
|  | **Classification** |  | **Aggregate** |  | **Coverage** |  | **Number Of Coding References** |  | | **Reference Number** | |  | | **Coded By Initials** | |  | | **Modified On** | |  | |
|  | **Codes\\Intervention process\Feelings** | | | | | | | | | | | | | | | | | | |  | |
|  |  |  | No |  | 0.0285 |  | 1 |  | | | | | | | | | | | |  |  |
|  |  |  |  |  |  |  |  |  | | | | | | | | | | | | | |
|  | | | | | | | | | | 1 |  | | MTK | |  | | 8/4/2021 3:20 PM | |  | |  |
|  | I: Can you tell me more?  R: I was depressed when I was pregnant. I had fears, I was worried that “am I going to deliver?” being it was my first pregnancy  I: So how did the counseling that you received help you to deal with the depression?  R: It was addressed. By the end of two sessions I was free from stress because she taught me what to do. | | | | | | | | | | | | | | | | | | |  | |
|  |  | | | | | | | | | | | | | | | | | | |  | |
|  | **Codes\\Participation of others\Attitude** | | | | | | | | | | | | | | | | | | |  | |
|  |  |  | No |  | 0.0451 |  | 2 |  | | | | | | | | | | | |  |  |
|  |  |  |  |  |  |  |  |  | | | | | | | | | | | | | |
|  | | | | | | | | | | 1 |  | | MTK | |  | | 8/4/2021 3:16 PM | |  | |  |
|  | I: So who else was present during the sessions?  R: My husband and I. The other family members were also happy because they knew that there is a visitor in our home. | | | | | | | | | | | | | | | | | | |  | |
|  |  | | | | | | | | | | | | | | | | | | |  | |
|  | | | | | | | | | | 2 |  | | MTK | |  | | 8/4/2021 3:16 PM | |  | |  |
|  | I: So, what was the reaction of your partner when he saw that there is a counselor coming and that he was involved in the sessions.  R: He was very happy because he knew that the counseling intervention was benefiting both of us and this was shown because he was able to engage in the discussion and answer questions when he was asked. | | | | | | | | | | | | | | | | | | |  | |
|  |  | | | | | | | | | | | | | | | | | | |  | |
|  | **Codes\\Participation of others\Partners** | | | | | | | | | | | | | | | | | | |  | |
|  |  |  | No |  | 0.0618 |  | 1 |  | | | | | | | | | | | |  |  |
|  |  |  |  |  |  |  |  |  | | | | | | | | | | | | | |
|  | | | | | | | | | | 1 |  | | MTK | |  | | 8/4/2021 3:15 PM | |  | |  |
|  | I: So who else was present during the sessions?  R: My husband and I. The other family members were also happy because they knew that there is a visitor in our home.  I: So, what was the reaction of your partner when he saw that there is a counselor coming and that he was involved in the sessions.  R: He was very happy because he knew that the counseling intervention was benefiting both of us and this was shown because he was able to engage in the discussion and answer questions when he was asked.  I: Was your partner present during all the sessions?  R: He was present during all the sessions.  I: Meaning that he did not miss a session?  R: No. He accepted the intervention | | | | | | | | | | | | | | | | | | |  | |
|  |  |  |  |  |  |  |  |  |  |  |  |  |  |  |  |  |  |  |  |  |  |
|  |  | | | | | | | | | | | | | | | | | | |  | |
|  |  |  |  |  |  |  |  |  |  |  |  |  |  |  |  |  |  |  |  |  |  |
|  |  |  |  |  |  |  |  |  |  |  |  |  |  |  |  |  |  |  |  |  |  |
|  |  |  |  |  |  |  |  |  |  |  |  |  |  |  |  |  |  |  |  |  |  |
| Formatted Reports\\Coding Summary by File Formatted Report | | | | | | | | | Page 57 of 145 | | | | | | | | | | | |  |
| 8/9/2021 8:08 AM | | | | | | | | | | | | | | | | | | | | |  |
|  | **Classification** |  | **Aggregate** |  | **Coverage** |  | **Number Of Coding References** |  | | **Reference Number** | |  | | **Coded By Initials** | |  | | **Modified On** | |  | |
|  | **Codes\\Personal history** | | | | | | | | | | | | | | | | | | |  | |
|  |  |  | No |  | 0.1500 |  | 2 |  | | | | | | | | | | | |  |  |
|  |  |  |  |  |  |  |  |  | | | | | | | | | | | | | |
|  | | | | | | | | | | 1 |  | | MTK | |  | | 8/4/2021 3:02 PM | |  | |  |
|  | I: Thank you very much. So I am going to ask you questions which are in two sections. Firstly, I would like to know how old you are. It would be helpful if you can remember the date, month and year when you were born…  R: I was born in 2000 on 23rd December.  I: So how old are you?  R: I am in my 21st year.  I: Meaning that you are 20 and in December you will be 21?  R: Yes.  I: How far did you go with your education?  R: Standard eight.  I: Can you tell us why you did not want to further your education?  R: I did not want to repeat and that was when I made a decision to stop school and get married.  I: How did it go in standard eight?  R: It was okay but I did not pass the national examinations.  I: So you decided to get married?  R: Yes.  I: So you are currently married?  R: Yes, I am married.  I: How many wives are in the marriage?  R: Meaning the type of marriage?  I: Are you in a polygamous marriage or you are alone?  R: I am alone.  I: Okay fine. How many children do you have?  R: This is the only child.  I: How old is the child?  R: One month and three weeks. | | | | | | | | | | | | | | | | | | |  | |
|  |  |  |  |  |  |  |  |  |  |  |  |  |  |  |  |  |  |  |  |  |  |
|  |  |  |  |  |  |  |  |  |  |  |  |  |  |  |  |  |  |  |  |  |  |
|  |  |  |  |  |  |  |  |  |  |  |  |  |  |  |  |  |  |  |  |  |  |
|  |  |  |  |  |  |  |  |  |  |  |  |  |  |  |  |  |  |  |  |  |  |
|  |  | | | | | | | | | | | | | | | | | | |  | |
|  | | | | | | | | | | 2 |  | | MTK | |  | | 8/4/2021 3:03 PM | |  | |  |
|  | I: Okay fine. Where do you currently stay?  R: At …….Village.  I: Which TA is …………?  R: Kabudula. | | | | | | | | | | | | | | | | | | |  | |
|  |  |  |  |  |  |  |  |  |  |  |  |  |  |  |  |  |  |  |  |  |  |
|  |  | | | | | | | | | | | | | | | | | | |  | |
|  | **Codes\\Place of intervention delivery** | | | | | | | | | | | | | | | | | | |  | |
|  |  |  | No |  | 0.0491 |  | 3 |  | | | | | | | | | | | |  |  |
|  |  |  |  |  |  |  |  |  | | | | | | | | | | | | | |
|  | | | | | | | | | | 1 |  | | MTK | |  | | 8/4/2021 3:13 PM | |  | |  |
|  | I: So you mentioned earlier on that it was good that the counselor was visiting you at home…  R: Yes.  I: I want you to explain to me clearly, how it was good to be visited by the counselor at home?  R: Yes it was very good. | | | | | | | | | | | | | | | | | | |  | |
|  |  | | | | | | | | | | | | | | | | | | |  | |
| Formatted Reports\\Coding Summary by File Formatted Report | | | | | | | | | Page 58 of 145 | | | | | | | | | | | |  |
| 8/9/2021 8:08 AM | | | | | | | | | | | | | | | | | | | | |  |
|  | **Classification** |  | **Aggregate** |  | **Coverage** |  | **Number Of Coding References** |  | | **Reference Number** | |  | | **Coded By Initials** | |  | | **Modified On** | |  | |
|  |  |  |  |  |  |  |  |  |  |  |  |  |  |  |  |  |  |  |  |  |  |
|  | | | | | | | | | | 2 |  | | MTK | |  | | 8/4/2021 3:21 PM | |  | |  |
|  | I: Okay. What I want to know is about the challenges that you had with the coming of the counselor at your home…  R: I had no challenge. | | | | | | | | | | | | | | | | | | |  | |
|  |  | | | | | | | | | | | | | | | | | | |  | |
|  | | | | | | | | | | 3 |  | | MTK | |  | | 8/4/2021 3:21 PM | |  | |  |
|  | I: Can you advise the research study that bringing counseling to the homes of participants is a good intervention…  R: It is very good because it helps you to deal with your problems. | | | | | | | | | | | | | | | | | | |  | |
|  |  | | | | | | | | | | | | | | | | | | |  | |
|  | **Codes\\Place of intervention delivery\Advantages** | | | | | | | | | | | | | | | | | | |  | |
|  |  |  | No |  | 0.0391 |  | 2 |  | | | | | | | | | | | |  |  |
|  |  |  |  |  |  |  |  |  | | | | | | | | | | | | | |
|  | | | | | | | | | | 1 |  | | MTK | |  | | 8/4/2021 3:13 PM | |  | |  |
|  | I: What do you think is the advantage of being visited by the counselor at your home versus you going somewhere to seek counseling intervention, like the hospital?  R: It was very good because she was leaving whatever she was supposed to do at her home and come to my home to provide the intervention.so that was very good.  I: So it was good?  R: Yes. | | | | | | | | | | | | | | | | | | |  | |
|  |  | | | | | | | | | | | | | | | | | | |  | |
|  | | | | | | | | | | 2 |  | | MTK | |  | | 8/4/2021 3:14 PM | |  | |  |
|  | I: Meaning that coming to your home eased your burden of travelling…  R: Yes. | | | | | | | | | | | | | | | | | | |  | |
|  |  | | | | | | | | | | | | | | | | | | |  | |
|  | **Codes\\Place of intervention delivery\Disadvantages** | | | | | | | | | | | | | | | | | | |  | |
|  |  |  | No |  | 0.0191 |  | 1 |  | | | | | | | | | | | |  |  |
|  |  |  |  |  |  |  |  |  | | | | | | | | | | | | | |
|  | | | | | | | | | | 1 |  | | MTK | |  | | 8/4/2021 3:14 PM | |  | |  |
|  | I: What would have happened if you were the one who was supposed to be going to seek the counseling intervention?  R: I would not have managed because I was pregnant and I would not manage to walk long distances. | | | | | | | | | | | | | | | | | | |  | |
|  |  | | | | | | | | | | | | | | | | | | |  | |
|  | **Codes\\Recommendations\Place of delivery** | | | | | | | | | | | | | | | | | | |  | |
|  |  |  | No |  | 0.0166 |  | 1 |  | | | | | | | | | | | |  |  |
|  |  |  |  |  |  |  |  |  | | | | | | | | | | | | | |
|  | | | | | | | | | | 1 |  | | MTK | |  | | 8/4/2021 3:22 PM | |  | |  |
|  | I: Can you advise the research study that bringing counseling to the homes of participants is a good intervention…  R: It is very good because it helps you to deal with your problems. | | | | | | | | | | | | | | | | | | |  | |
|  |  | | | | | | | | | | | | | | | | | | |  | |
|  |  |  |  |  |  |  |  |  |  |  |  |  |  |  |  |  |  |  |  |  |  |
|  |  |  |  |  |  |  |  |  |  |  |  |  |  |  |  |  |  |  |  |  |  |
| Formatted Reports\\Coding Summary by File Formatted Report | | | | | | | | | Page 59 of 145 | | | | | | | | | | | |  |
| 8/9/2021 8:08 AM | | | | | | | | | | | | | | | | | | | | |  |
|  | **Classification** |  | **Aggregate** |  | **Coverage** |  | **Number Of Coding References** |  | | **Reference Number** | |  | | **Coded By Initials** | |  | | **Modified On** | |  | |
|  | **Codes\\THPP providers** | | | | | | | | | | | | | | | | | | |  | |
|  |  |  | No |  | 0.0232 |  | 1 |  | | | | | | | | | | | |  |  |
|  |  |  |  |  |  |  |  |  | | | | | | | | | | | | | |
|  | | | | | | | | | | 1 |  | | MTK | |  | | 8/4/2021 3:10 PM | |  | |  |
|  | I: So the counseling aspect aside, what was it that you liked about the counselor herself?  R: She was cheerful in her approach.  I: What do you mean?  R: She was very open with me. She was explaining everything thoroughly to me. She was not hiding anything. | | | | | | | | | | | | | | | | | | |  | |
|  |  | | | | | | | | | | | | | | | | | | |  | |
|  | **Codes\\THPP providers\Characteristics** | | | | | | | | | | | | | | | | | | |  | |
|  |  |  | No |  | 0.0055 |  | 1 |  | | | | | | | | | | | |  |  |
|  |  |  |  |  |  |  |  |  | | | | | | | | | | | | | |
|  | | | | | | | | | | 1 |  | | MTK | |  | | 8/4/2021 3:11 PM | |  | |  |
|  | I: How can you describe her behavior?  R: She was cheerful. | | | | | | | | | | | | | | | | | | |  | |
|  |  | | | | | | | | | | | | | | | | | | |  | |
|  | **Codes\\THPP providers\Relationship** | | | | | | | | | | | | | | | | | | |  | |
|  |  |  | No |  | 0.0472 |  | 1 |  | | | | | | | | | | | |  |  |
|  |  |  |  |  |  |  |  |  | | | | | | | | | | | | | |
|  | | | | | | | | | | 1 |  | | MTK | |  | | 8/4/2021 3:11 PM | |  | |  |
|  | I: How can you describe your relationship with your counselor?  R: It was a good relationship because we were free and open with each other.  I: Was she new to you or you knew her before?  R: I knew her from [Name of village].  I: Did you note any change in her from the way you knew her before?  R: I did not know that she provides counseling to people in the community.  I: I thank you so much for telling me that you had a good relationship with the counselor and that made you to benefit from the counseling…  R: Yes. | | | | | | | | | | | | | | | | | | |  | |
|  |  |  |  |  |  |  |  |  |  |  |  |  |  |  |  |  |  |  |  |  |  |
|  |  | | | | | | | | | | | | | | | | | | |  | |
|  | **Files\\IDIs\\IDI #13** | | | | | | | | | | | | | | | | | | |  | |
|  | **Code** | | | | | | | | | | | | | | | | | | |  | |
|  | **Codes\\Intervention experience** | | | | | | | | | | | | | | | | | | |  | |
|  |  |  | No |  | 0.0704 |  | 2 |  | | | | | | | | | | | |  |  |
|  |  |  |  |  |  |  |  |  | | | | | | | | | | | | | |
|  | | | | | | | | | | 1 |  | | MTK | |  | | 8/4/2021 3:31 PM | |  | |  |
|  | I: What have been your experiences of the pregnancy of this child?  R: I can say that I had good experiences because of the counseling that I was receiving. It come at the right time, when I was starting antenatal care | | | | | | | | | | | | | | | | | | |  | |
|  |  | | | | | | | | | | | | | | | | | | |  | |
|  |  |  |  |  |  |  |  |  |  |  |  |  |  |  |  |  |  |  |  |  |  |
| Formatted Reports\\Coding Summary by File Formatted Report | | | | | | | | | Page 60 of 145 | | | | | | | | | | | |  |
| 8/9/2021 8:08 AM | | | | | | | | | | | | | | | | | | | | |  |
|  | **Classification** |  | **Aggregate** |  | **Coverage** |  | **Number Of Coding References** |  | | **Reference Number** | |  | | **Coded By Initials** | |  | | **Modified On** | |  | |
|  |  |  |  |  |  |  |  |  |  |  |  |  |  |  |  |  |  |  |  |  |  |
|  | | | | | | | | | | 2 |  | | MTK | |  | | 8/5/2021 11:00 AM | |  | |  |
|  | R: The counseling was very good because it has protected me in many aspects. I was a depressed person but now I am trying to associate with my friends because previously I was not able to associate with my friends. I am encouraging other women that they should also join the programme  I: Can you tell me what was your main problem?  R: I had worries, and thinking most of the times. I also used to experience persistent headache. I was depressed because of a lot of problems I was going through, I had no support for my child. | | | | | | | | | | | | | | | | | | |  | |
|  |  | | | | | | | | | | | | | | | | | | |  | |
|  | **Codes\\Intervention experience\Aspects** | | | | | | | | | | | | | | | | | | |  | |
|  |  |  | No |  | 0.0470 |  | 1 |  | | | | | | | | | | | |  |  |
|  |  |  |  |  |  |  |  |  | | | | | | | | | | | | | |
|  | | | | | | | | | | 1 |  | | MTK | |  | | 8/5/2021 11:49 AM | |  | |  |
|  | I: Fine. So you have said that the counseling went on well. What aspect of the counseling intervention did you find to be most useful?  R: Taking care of a pregnant woman.  I: I would like you to feel free. The counselor has been visiting you and you have said that the counseling was very good. So what aspect of the counseling intervention did you find to be most useful because the counselor was coming with different lessons…?  R: The aspect of enhancing my relationship with people around me. | | | | | | | | | | | | | | | | | | |  | |
|  |  | | | | | | | | | | | | | | | | | | |  | |
|  | **Codes\\Intervention experience\Dislikes** | | | | | | | | | | | | | | | | | | |  | |
|  |  |  | No |  | 0.0083 |  | 1 |  | | | | | | | | | | | |  |  |
|  |  |  |  |  |  |  |  |  | | | | | | | | | | | | | |
|  | | | | | | | | | | 1 |  | | MTK | |  | | 8/5/2021 11:51 AM | |  | |  |
|  | I: So what aspect of the counseling did you not like?  R: Nothing, I liked everything. | | | | | | | | | | | | | | | | | | |  | |
|  |  | | | | | | | | | | | | | | | | | | |  | |
|  | **Codes\\Intervention experience\Influence on others** | | | | | | | | | | | | | | | | | | |  | |
|  |  |  | No |  | 0.0627 |  | 2 |  | | | | | | | | | | | |  |  |
|  |  |  |  |  |  |  |  |  | | | | | | | | | | | | | |
|  | | | | | | | | | | 1 |  | | MTK | |  | | 8/5/2021 11:58 AM | |  | |  |
|  | I: Can you tell me if there were some challenges when the counselor was coming to your home for counseling sessions?  R: Everything was okay.  I: Were other people not surprised to see the counselor coming to your home?  R: No.  I: What questions were people surrounding you asking when they saw you with the counselor?  R: They were asking but we were encouraging them.  I: What were you telling them?  R: We were telling them that a pregnant woman should not be stressed as well as a lactating woman. | | | | | | | | | | | | | | | | | | |  | |
|  |  |  |  |  |  |  |  |  |  |  |  |  |  |  |  |  |  |  |  |  |  |
|  |  | | | | | | | | | | | | | | | | | | |  | |
|  | | | | | | | | | | 2 |  | | MTK | |  | | 8/5/2021 11:59 AM | |  | |  |
|  | I: Can we say that you can be counseling other women to deal with their problems?  R: Yes.  I: And you think this counseling is essential for women…?  R: Yes. | | | | | | | | | | | | | | | | | | |  | |
|  |  | | | | | | | | | | | | | | | | | | |  | |
|  |  |  |  |  |  |  |  |  |  |  |  |  |  |  |  |  |  |  |  |  |  |
| Formatted Reports\\Coding Summary by File Formatted Report | | | | | | | | | Page 61 of 145 | | | | | | | | | | | |  |
| 8/9/2021 8:08 AM | | | | | | | | | | | | | | | | | | | | |  |
|  | **Classification** |  | **Aggregate** |  | **Coverage** |  | **Number Of Coding References** |  | | **Reference Number** | |  | | **Coded By Initials** | |  | | **Modified On** | |  | |
|  | **Codes\\Intervention experience\Likes** | | | | | | | | | | | | | | | | | | |  | |
|  |  |  | No |  | 0.0208 |  | 1 |  | | | | | | | | | | | |  |  |
|  |  |  |  |  |  |  |  |  | | | | | | | | | | | | | |
|  | | | | | | | | | | 1 |  | | MTK | |  | | 8/5/2021 11:51 AM | |  | |  |
|  | I: So, in general, what did you like about the counseling?  R: I liked the aspect of taking care of the pregnant woman because if there cannot be good care of the pregnant woman, a pregnant woman can have a miscarriage. | | | | | | | | | | | | | | | | | | |  | |
|  |  | | | | | | | | | | | | | | | | | | |  | |
|  | **Codes\\Intervention process** | | | | | | | | | | | | | | | | | | |  | |
|  |  |  | No |  | 0.0101 |  | 1 |  | | | | | | | | | | | |  |  |
|  |  |  |  |  |  |  |  |  | | | | | | | | | | | | | |
|  | | | | | | | | | | 1 |  | | MTK | |  | | 8/5/2021 11:53 AM | |  | |  |
|  | I: What was happening?  R: When she came, she was telling us to meet at one place and we were discussing. | | | | | | | | | | | | | | | | | | |  | |
|  |  | | | | | | | | | | | | | | | | | | |  | |
|  | **Codes\\Intervention process\Delivery** | | | | | | | | | | | | | | | | | | |  | |
|  |  |  | No |  | 0.0174 |  | 1 |  | | | | | | | | | | | |  |  |
|  |  |  |  |  |  |  |  |  | | | | | | | | | | | | | |
|  | | | | | | | | | | 1 |  | | MTK | |  | | 8/5/2021 11:55 AM | |  | |  |
|  | I: What else apart from encouraging you?  R: She introduced us to this program so that we should be breastfeeding well and relieve our stress, that we shouldn’t be thinking too much. | | | | | | | | | | | | | | | | | | |  | |
|  |  | | | | | | | | | | | | | | | | | | |  | |
|  | **Codes\\Intervention process\Feelings** | | | | | | | | | | | | | | | | | | |  | |
|  |  |  | No |  | 0.0251 |  | 1 |  | | | | | | | | | | | |  |  |
|  |  |  |  |  |  |  |  |  | | | | | | | | | | | | | |
|  | | | | | | | | | | 1 |  | | MTK | |  | | 8/5/2021 12:00 PM | |  | |  |
|  | I: One of the things that was happening in this program was that a person was supposed to assess her unhelpful behavior and helpful behavior…  R: Yes I am encouraging pregnant women not to have stress.  I: Meaning that you now have helpful thoughts right?  R: Yes. | | | | | | | | | | | | | | | | | | |  | |
|  |  | | | | | | | | | | | | | | | | | | |  | |
|  | **Codes\\Intervention process\Number of visits** | | | | | | | | | | | | | | | | | | |  | |
|  |  |  | No |  | 0.0468 |  | 1 |  | | | | | | | | | | | |  |  |
|  |  |  |  |  |  |  |  |  | | | | | | | | | | | | | |
|  | | | | | | | | | | 1 |  | | MTK | |  | | 8/4/2021 3:45 PM | |  | |  |
|  | I: So moving forward with our discussion, as I said, you were enrolled in this study because you scored high during the screening of depression meaning that you were depressed. This means that you were supposed to go through counseling that was provided to you at your home… You were visited by the counselor… So in general, how can you describe the counseling? | | | | | | | | | | | | | | | | | | |  | |
|  |  | | | | | | | | | | | | | | | | | | |  | |
|  |  |  |  |  |  |  |  |  |  |  |  |  |  |  |  |  |  |  |  |  |  |
| Formatted Reports\\Coding Summary by File Formatted Report | | | | | | | | | Page 62 of 145 | | | | | | | | | | | |  |
| 8/9/2021 8:08 AM | | | | | | | | | | | | | | | | | | | | |  |
|  | **Classification** |  | **Aggregate** |  | **Coverage** |  | **Number Of Coding References** |  | | **Reference Number** | |  | | **Coded By Initials** | |  | | **Modified On** | |  | |
|  | **Codes\\Participation of others** | | | | | | | | | | | | | | | | | | |  | |
|  |  |  | No |  | 0.0263 |  | 1 |  | | | | | | | | | | | |  |  |
|  |  |  |  |  |  |  |  |  | | | | | | | | | | | | | |
|  | | | | | | | | | | 1 |  | | MTK | |  | | 8/5/2021 11:52 AM | |  | |  |
|  | I: Now I would like to talk about the counselor herself… what was it that you liked about her as a person when she was coming to your home for counseling?  R: She was encouraging me and she was visiting me at home, meeting me with others then we will go through the discussing. | | | | | | | | | | | | | | | | | | |  | |
|  |  | | | | | | | | | | | | | | | | | | |  | |
|  | **Codes\\Participation of others\Attitude** | | | | | | | | | | | | | | | | | | |  | |
|  |  |  | No |  | 0.0475 |  | 1 |  | | | | | | | | | | | |  |  |
|  |  |  |  |  |  |  |  |  | | | | | | | | | | | | | |
|  | | | | | | | | | | 1 |  | | MTK | |  | | 8/5/2021 11:59 AM | |  | |  |
|  | I: Can you tell me if there were some challenges when the counselor was coming to your home for counseling sessions?  R: Everything was okay.  I: Were other people not surprised to see the counselor coming to your home?  R: No.  I: What questions were people surrounding you asking when they saw you with the counselor?  R: They were asking but we were encouraging them.  I: What were you telling them?  R: We were telling them that a pregnant woman should not be stressed as well as a lactating woman. | | | | | | | | | | | | | | | | | | |  | |
|  |  |  |  |  |  |  |  |  |  |  |  |  |  |  |  |  |  |  |  |  |  |
|  |  | | | | | | | | | | | | | | | | | | |  | |
|  | **Codes\\Participation of others\Others** | | | | | | | | | | | | | | | | | | |  | |
|  |  |  | No |  | 0.0218 |  | 2 |  | | | | | | | | | | | |  |  |
|  |  |  |  |  |  |  |  |  | | | | | | | | | | | | | |
|  | | | | | | | | | | 1 |  | | MTK | |  | | 8/5/2021 11:53 AM | |  | |  |
|  | I: Who were these people?  R: My friend [Name of friend] and my mother. | | | | | | | | | | | | | | | | | | |  | |
|  |  | | | | | | | | | | | | | | | | | | |  | |
|  | | | | | | | | | | 2 |  | | MTK | |  | | 8/5/2021 11:54 AM | |  | |  |
|  | I: Was there another person present during the counseling sessions apart from these ones?  R: My sister in – law.  I: The sister to your husband?  R: Yes. | | | | | | | | | | | | | | | | | | |  | |
|  |  | | | | | | | | | | | | | | | | | | |  | |
|  | **Codes\\Participation of others\Partners** | | | | | | | | | | | | | | | | | | |  | |
|  |  |  | No |  | 0.0059 |  | 1 |  | | | | | | | | | | | |  |  |
|  |  |  |  |  |  |  |  |  | | | | | | | | | | | | | |
|  | | | | | | | | | | 1 |  | | MTK | |  | | 8/5/2021 11:53 AM | |  | |  |
|  | I: Was your husband present during the discussions?  R: Yes. | | | | | | | | | | | | | | | | | | |  | |
|  |  | | | | | | | | | | | | | | | | | | |  | |
|  |  |  |  |  |  |  |  |  |  |  |  |  |  |  |  |  |  |  |  |  |  |
| Formatted Reports\\Coding Summary by File Formatted Report | | | | | | | | | Page 63 of 145 | | | | | | | | | | | |  |
| 8/9/2021 8:08 AM | | | | | | | | | | | | | | | | | | | | |  |
|  | **Classification** |  | **Aggregate** |  | **Coverage** |  | **Number Of Coding References** |  | | **Reference Number** | |  | | **Coded By Initials** | |  | | **Modified On** | |  | |
|  | **Codes\\Personal history** | | | | | | | | | | | | | | | | | | |  | |
|  |  |  | No |  | 0.1478 |  | 2 |  | | | | | | | | | | | |  |  |
|  |  |  |  |  |  |  |  |  | | | | | | | | | | | | | |
|  | | | | | | | | | | 1 |  | | MTK | |  | | 8/4/2021 3:28 PM | |  | |  |
|  | I: Thank you very much. So firstly when were you born?  R: I was born in 1994?  I: Can you remember the year and date?  R: I was born on 11th March.  I: How old are you?  R: I am 24 years old?  I: 24?  R: 25.  I: Yes, you are 25 years old.  I: How far did you go with your education?  R: I went up to form 2.  I: Did you write examinations?  R: No, I did not have school fees.  I: Which term did you stop going to school?  R: First term.  I: Are you married?  R: Yes I am married.  I: How many wives are you in the marriage?  R: Only one wife.  I: You are his first wife…?  R: Yes.  I: And he is your first husband?  R: Yes.  I: I would like to know how many pregnancies have you had?  R: Two.  I: Two?  R: Yes.  I: When was the child born?  R: S/he was born on 11th December 2020  R: Yes. | | | | | | | | | | | | | | | | | | |  | |
|  |  |  |  |  |  |  |  |  |  |  |  |  |  |  |  |  |  |  |  |  |  |
|  |  |  |  |  |  |  |  |  |  |  |  |  |  |  |  |  |  |  |  |  |  |
|  |  |  |  |  |  |  |  |  |  |  |  |  |  |  |  |  |  |  |  |  |  |
|  |  |  |  |  |  |  |  |  |  |  |  |  |  |  |  |  |  |  |  |  |  |
|  |  | | | | | | | | | | | | | | | | | | |  | |
|  | | | | | | | | | | 2 |  | | MTK | |  | | 8/4/2021 3:32 PM | |  | |  |
|  | I: Okay. So you have told me that you have had two pregnancies meaning that this one is your second born?  R: Yes.  I: Is the other child alive?  R: Yes.  I: Did you experience any abortions?  R:No.  I: Meaning that you have had two pregnancies and that both children are alive…  R: Yes.  I: Where do you come from?  R: …….. village.  I: Which TA?  R: Kabudula. | | | | | | | | | | | | | | | | | | |  | |
|  |  |  |  |  |  |  |  |  |  |  |  |  |  |  |  |  |  |  |  |  |  |
|  |  |  |  |  |  |  |  |  |  |  |  |  |  |  |  |  |  |  |  |  |  |
|  |  |  |  |  |  |  |  |  |  |  |  |  |  |  |  |  |  |  |  |  |  |
|  |  | | | | | | | | | | | | | | | | | | |  | |
| Formatted Reports\\Coding Summary by File Formatted Report | | | | | | | | | Page 64 of 145 | | | | | | | | | | | |  |
| 8/9/2021 8:08 AM | | | | | | | | | | | | | | | | | | | | |  |
|  | **Classification** |  | **Aggregate** |  | **Coverage** |  | **Number Of Coding References** |  | | **Reference Number** | |  | | **Coded By Initials** | |  | | **Modified On** | |  | |
|  | **Codes\\Place of intervention delivery** | | | | | | | | | | | | | | | | | | |  | |
|  |  |  | No |  | 0.0473 |  | 1 |  | | | | | | | | | | | |  |  |
|  |  |  |  |  |  |  |  |  | | | | | | | | | | | | | |
|  | | | | | | | | | | 1 |  | | MTK | |  | | 8/5/2021 11:57 AM | |  | |  |
|  | I: Okay. As we move towards the end of our discussion, the counselor was the one who was coming to your home to provide the counseling session…  R: Yes.  I: In terms of the place where you were meeting, can you say that it was good for the counselor to be coming to your home to provide the counseling?  R: It was good because had it been that it was done away from home, we have no means of transport and we could not manage.  I: Meaning that it was good that you were meeting at your home?  R: Yes. | | | | | | | | | | | | | | | | | | |  | |
|  |  |  |  |  |  |  |  |  |  |  |  |  |  |  |  |  |  |  |  |  |  |
|  |  | | | | | | | | | | | | | | | | | | |  | |
|  | **Codes\\Place of intervention delivery\Disadvantages** | | | | | | | | | | | | | | | | | | |  | |
|  |  |  | No |  | 0.0323 |  | 1 |  | | | | | | | | | | | |  |  |
|  |  |  |  |  |  |  |  |  | | | | | | | | | | | | | |
|  | | | | | | | | | | 1 |  | | MTK | |  | | 8/5/2021 11:58 AM | |  | |  |
|  | I: What would have happened had it been that you were told to travel somewhere to seek the counseling intervention session?  R: It would be a challenge.  I: Meaning that you would be missing some of the sessions?  R: Yes.  I: You have said that it would be difficult for you to travel to access the counseling intervention sessions…  R: Yes. | | | | | | | | | | | | | | | | | | |  | |
|  |  | | | | | | | | | | | | | | | | | | |  | |
|  | **Codes\\THPP providers** | | | | | | | | | | | | | | | | | | |  | |
|  |  |  | No |  | 0.0263 |  | 1 |  | | | | | | | | | | | |  |  |
|  |  |  |  |  |  |  |  |  | | | | | | | | | | | | | |
|  | | | | | | | | | | 1 |  | | MTK | |  | | 8/5/2021 11:52 AM | |  | |  |
|  | I: Now I would like to talk about the counselor herself… what was it that you liked about her as a person when she was coming to your home for counseling?  R: She was encouraging me and she was visiting me at home, meeting me with others then we will go through the discussing. | | | | | | | | | | | | | | | | | | |  | |
|  |  | | | | | | | | | | | | | | | | | | |  | |
|  | **Codes\\THPP providers\Characteristics** | | | | | | | | | | | | | | | | | | |  | |
|  |  |  | No |  | 0.0128 |  | 1 |  | | | | | | | | | | | |  |  |
|  |  |  |  |  |  |  |  |  | | | | | | | | | | | | | |
|  | | | | | | | | | | 1 |  | | MTK | |  | | 8/5/2021 11:54 AM | |  | |  |
|  | I: So how can you describe the behavior of the counselor?  R: She is a well behaved woman because she encourages us and giving us hope. | | | | | | | | | | | | | | | | | | |  | |
|  |  | | | | | | | | | | | | | | | | | | |  | |
|  |  |  |  |  |  |  |  |  |  |  |  |  |  |  |  |  |  |  |  |  |  |
|  |  |  |  |  |  |  |  |  |  |  |  |  |  |  |  |  |  |  |  |  |  |
| Formatted Reports\\Coding Summary by File Formatted Report | | | | | | | | | Page 65 of 145 | | | | | | | | | | | |  |
| 8/9/2021 8:08 AM | | | | | | | | | | | | | | | | | | | | |  |
|  | **Classification** |  | **Aggregate** |  | **Coverage** |  | **Number Of Coding References** |  | | **Reference Number** | |  | | **Coded By Initials** | |  | | **Modified On** | |  | |
|  | **Codes\\THPP providers\Relationship** | | | | | | | | | | | | | | | | | | |  | |
|  |  |  | No |  | 0.0988 |  | 1 |  | | | | | | | | | | | |  |  |
|  |  |  |  |  |  |  |  |  | | | | | | | | | | | | | |
|  | | | | | | | | | | 1 |  | | MTK | |  | | 8/5/2021 11:56 AM | |  | |  |
|  | I: So how was your relationship with the counselor?  R: It was very important because of the sessions where we were discussing about health of pregnant and lactating mothers.  I: I would like you describe your relationship with the counselor when she was coming to you during the counseling sessions?  R: She was able to encourage us to come here to the clinic…  I: I don’t understand your description of your relationship with the counselor… Even in the village you are able to relate to people right?  R: Yes.  I: Maybe I should ask it in this way; during sessions were you agreeing on the next visit date yah?  R: Yes.  I: I would like to know if this was done according to your agreement…  R: Yes.  I: Was she able to come at the agreed time?  R: Yes.  I: Was she able to honor her promise that she was coming to your home as promised and agreed?  R: Yes.  I: Was she absent without telling you any reason for her absenteeism?  R: Never.  I: It never happened… Can we conclude that you had a good relationship with your counselor?  R: Yes. | | | | | | | | | | | | | | | | | | |  | |
|  |  |  |  |  |  |  |  |  |  |  |  |  |  |  |  |  |  |  |  |  |  |
|  |  |  |  |  |  |  |  |  |  |  |  |  |  |  |  |  |  |  |  |  |  |
|  |  | | | | | | | | | | | | | | | | | | |  | |
|  | **Files\\IDIs\\IDI #14** | | | | | | | | | | | | | | | | | | |  | |
|  | **Code** | | | | | | | | | | | | | | | | | | |  | |
|  | **Codes\\Intervention experience** | | | | | | | | | | | | | | | | | | |  | |
|  |  |  | No |  | 0.0866 |  | 2 |  | | | | | | | | | | | |  |  |
|  |  |  |  |  |  |  |  |  | | | | | | | | | | | | | |
|  | | | | | | | | | | 1 |  | | MTK | |  | | 8/5/2021 12:04 PM | |  | |  |
|  | I: Fine. So during that time when you were pregnant, during the screening for depression, it was found that you had high score. What happened?  R: I had a lot of stress because I did not know if I was going to manage to deliver a baby, | | | | | | | | | | | | | | | | | | |  | |
|  |  | | | | | | | | | | | | | | | | | | |  | |
|  | | | | | | | | | | 2 |  | | MTK | |  | | 8/5/2021 12:20 PM | |  | |  |
|  | I: Okay. Thank you very much. So our second part, I am going to ask you about the study in which you were taking part. What can you tell me about the counseling that you were receiving?  R: This counseling has helped me to associate with other people and have helpful behavior, now I do not think too much as I used to.  I: What were you previously thinking about?  R: I was thinking a lot.  I: Can you share me some of the thoughts that you had?  R: They were a lot.  I: Do you remember some of the thoughts?  R: Because it was long time ago, I forgot some of them. However, I stopped having unhelpful thoughts. | | | | | | | | | | | | | | | | | | |  | |
|  |  |  |  |  |  |  |  |  |  |  |  |  |  |  |  |  |  |  |  |  |  |
|  |  | | | | | | | | | | | | | | | | | | |  | |
|  |  |  |  |  |  |  |  |  |  |  |  |  |  |  |  |  |  |  |  |  |  |
| Formatted Reports\\Coding Summary by File Formatted Report | | | | | | | | | Page 66 of 145 | | | | | | | | | | | |  |
| 8/9/2021 8:08 AM | | | | | | | | | | | | | | | | | | | | |  |
|  | **Classification** |  | **Aggregate** |  | **Coverage** |  | **Number Of Coding References** |  | | **Reference Number** | |  | | **Coded By Initials** | |  | | **Modified On** | |  | |
|  | **Codes\\Intervention experience\Aspects** | | | | | | | | | | | | | | | | | | |  | |
|  |  |  | No |  | 0.0352 |  | 1 |  | | | | | | | | | | | |  |  |
|  |  |  |  |  |  |  |  |  | | | | | | | | | | | | | |
|  | | | | | | | | | | 1 |  | | MTK | |  | | 8/5/2021 12:20 PM | |  | |  |
|  | I: So, talking about the counseling that you were receiving, I hope it was the counseling that helped you to leave unhelpful thoughts…?  R: Yes.  I: What did you find to be most helpful in the counseling?  R: It was helpful because it helped me to stop having unhelpful thoughts. It helped me a lot and I would like the intervention to continue. | | | | | | | | | | | | | | | | | | |  | |
|  |  | | | | | | | | | | | | | | | | | | |  | |
|  | **Codes\\Intervention experience\Barriers** | | | | | | | | | | | | | | | | | | |  | |
|  |  |  | No |  | 0.0148 |  | 1 |  | | | | | | | | | | | |  |  |
|  |  |  |  |  |  |  |  |  | | | | | | | | | | | | | |
|  | | | | | | | | | | 1 |  | | MTK | |  | | 8/5/2021 12:22 PM | |  | |  |
|  | I: Fine. Were there any barriers to the counseling that you received?  R: No.  I: And you can confirm that there were no any barriers?  R: No. | | | | | | | | | | | | | | | | | | |  | |
|  |  | | | | | | | | | | | | | | | | | | |  | |
|  | **Codes\\Intervention experience\Dislikes** | | | | | | | | | | | | | | | | | | |  | |
|  |  |  | No |  | 0.0294 |  | 2 |  | | | | | | | | | | | |  |  |
|  |  |  |  |  |  |  |  |  | | | | | | | | | | | | | |
|  | | | | | | | | | | 1 |  | | MTK | |  | | 8/5/2021 12:21 PM | |  | |  |
|  | I: Okay… Now I would like to know; was there any aspect of the counseling that you did not like?  R: No I liked every aspect of the counseling because everything was helpful in my life. | | | | | | | | | | | | | | | | | | |  | |
|  |  | | | | | | | | | | | | | | | | | | |  | |
|  | | | | | | | | | | 2 |  | | MTK | |  | | 8/5/2021 12:21 PM | |  | |  |
|  | I: So there was nothing you felt like “this is just wasting my time”?  R: Nothing. Everything was okay. | | | | | | | | | | | | | | | | | | |  | |
|  |  | | | | | | | | | | | | | | | | | | |  | |
|  | **Codes\\Intervention experience\Likes** | | | | | | | | | | | | | | | | | | |  | |
|  |  |  | No |  | 0.0410 |  | 1 |  | | | | | | | | | | | |  |  |
|  |  |  |  |  |  |  |  |  | | | | | | | | | | | | | |
|  | | | | | | | | | | 1 |  | | MTK | |  | | 8/5/2021 12:21 PM | |  | |  |
|  | I: So there were several lessons that were covered during the counseling sessions; healthy of the mother and child, your relationship with the child and your relationship with people around you…  R: Yes…  I: What lesson did you like the most?  R: My relationship with people around me and my relationship with the child.  I: So you liked this very much?  R: I liked this one because it is very helpful. | | | | | | | | | | | | | | | | | | |  | |
|  |  |  |  |  |  |  |  |  |  |  |  |  |  |  |  |  |  |  |  |  |  |
|  |  | | | | | | | | | | | | | | | | | | |  | |
|  | **Codes\\Intervention process\Feelings** | | | | | | | | | | | | | | | | | | |  | |
|  |  |  | No |  | 0.1011 |  | 2 |  | | | | | | | | | | | |  |  |
|  |  |  |  |  |  |  |  |  | | | | | | | | | | | | | |
|  | | | | | | | | | | 1 |  | | MTK | |  | | 8/5/2021 12:05 PM | |  | |  |
|  | I: Okay… and you are happy now?  R: Yes I am happy.  I: Because this is what was stressful to you…  R: I am no longer stressed because I have the gift that I was waiting for [a Baby]. | | | | | | | | | | | | | | | | | | |  | |
|  |  | | | | | | | | | | | | | | | | | | |  | |
| Formatted Reports\\Coding Summary by File Formatted Report | | | | | | | | | Page 67 of 145 | | | | | | | | | | | |  |
| 8/9/2021 8:08 AM | | | | | | | | | | | | | | | | | | | | |  |
|  | **Classification** |  | **Aggregate** |  | **Coverage** |  | **Number Of Coding References** |  | | **Reference Number** | |  | | **Coded By Initials** | |  | | **Modified On** | |  | |
|  |  |  |  |  |  |  |  |  |  |  |  |  |  |  |  |  |  |  |  |  |  |
|  | | | | | | | | | | 2 |  | | MTK | |  | | 8/5/2021 12:41 PM | |  | |  |
|  | I: Okay. Now as we are going towards the end of our discussion, I would like you to tell me how you compare your life from the time when you were screened for depression to this time?  R: I am able to differentiate the past and now because I now have peace of mind but now I have peace of mind. I had no peace of mind before  I: Did your husband know that you had no peace of mind?  R: He knew.  I: And is he able to see that you now have peace of mind?  R: He knows.  I: Can we say that he is just failing to say that he has noted some change in you since you started receiving counseling?  R: Yes he cannot say it because he knows that he was thinking I was not saying the truth. He has noted change in me compared to how I was in the past.  I: And he sees that you are now free.  R: I am free than in the past. | | | | | | | | | | | | | | | | | | |  | |
|  |  |  |  |  |  |  |  |  |  |  |  |  |  |  |  |  |  |  |  |  |  |
|  |  | | | | | | | | | | | | | | | | | | |  | |
|  | **Codes\\Intervention process\Home situation** | | | | | | | | | | | | | | | | | | |  | |
|  |  |  | No |  | 0.0500 |  | 1 |  | | | | | | | | | | | |  |  |
|  |  |  |  |  |  |  |  |  | | | | | | | | | | | | | |
|  | | | | | | | | | | 1 |  | | MTK | |  | | 8/5/2021 12:41 PM | |  | |  |
|  | I: Did your husband know that you had no peace of mind?  R: He knew.  I: And is he able to see that you now have peace of mind?  R: He knows.  I: Can we say that he is just failing to say that he has noted some change in you since you started receiving counseling?  R: Yes he cannot say it because he knows that he was thinking I was not saying the truth. He has noted change in me compared to how I was in the past.  I: And he sees that you are now free.  R: I am free than in the past. | | | | | | | | | | | | | | | | | | |  | |
|  |  |  |  |  |  |  |  |  |  |  |  |  |  |  |  |  |  |  |  |  |  |
|  |  | | | | | | | | | | | | | | | | | | |  | |
|  | **Codes\\Other interesting issues** | | | | | | | | | | | | | | | | | | |  | |
|  |  |  | No |  | 0.0633 |  | 1 |  | | | | | | | | | | | |  |  |
|  |  |  |  |  |  |  |  |  | | | | | | | | | | | | | |
|  | | | | | | | | | | 1 |  | | MTK | |  | | 8/5/2021 12:44 PM | |  | |  |
|  | I: Thank you very much. My final words to you are that you need to continue to be a counselor of your own so that you can continue having helpful behavior. I understand problems will always be there in life but if you have healthy ways of dealing with your problems, you find that you cannot be stressed. At the same time, you are able to counsel other women. I thank you for taking the time in this interview and may God bless you. Before we close, maybe you have final words?  R: My final words are that they should provide us with support such as soap because we are living in the village where these things are scarce. | | | | | | | | | | | | | | | | | | |  | |
|  |  | | | | | | | | | | | | | | | | | | |  | |
|  | **Codes\\Participation of others\Others** | | | | | | | | | | | | | | | | | | |  | |
|  |  |  | No |  | 0.0099 |  | 1 |  | | | | | | | | | | | |  |  |
|  |  |  |  |  |  |  |  |  | | | | | | | | | | | | | |
|  | | | | | | | | | | 1 |  | | MTK | |  | | 8/5/2021 12:38 PM | |  | |  |
|  | I: Who else was present during the counseling sessions?  R: My mother, myself and the counselor. | | | | | | | | | | | | | | | | | | |  | |
|  |  | | | | | | | | | | | | | | | | | | |  | |
|  |  |  |  |  |  |  |  |  |  |  |  |  |  |  |  |  |  |  |  |  |  |
| Formatted Reports\\Coding Summary by File Formatted Report | | | | | | | | | Page 68 of 145 | | | | | | | | | | | |  |
| 8/9/2021 8:08 AM | | | | | | | | | | | | | | | | | | | | |  |
|  | **Classification** |  | **Aggregate** |  | **Coverage** |  | **Number Of Coding References** |  | | **Reference Number** | |  | | **Coded By Initials** | |  | | **Modified On** | |  | |
|  | **Codes\\Participation of others\Partners** | | | | | | | | | | | | | | | | | | |  | |
|  |  |  | No |  | 0.1087 |  | 2 |  | | | | | | | | | | | |  |  |
|  |  |  |  |  |  |  |  |  | | | | | | | | | | | | | |
|  | | | | | | | | | | 1 |  | | MTK | |  | | 8/5/2021 12:39 PM | |  | |  |
|  | I: Okay. What about the father of the child, was there a time when he was present during the sessions?  R: No he was not present.  I: Why do you think he was not present?  R: He was saying that they are useless things and that was why he did not attend any session.  I: Meaning that he did not attend even a single session?  R: No.  I: Was he aware that you were going through stress?  R: When I explained my problems, what I was going through, He was saying that I was not telling him the truth.  I: How does he perceive you now?  R: I don’t know how he perceives me now. | | | | | | | | | | | | | | | | | | |  | |
|  |  |  |  |  |  |  |  |  |  |  |  |  |  |  |  |  |  |  |  |  |  |
|  |  | | | | | | | | | | | | | | | | | | |  | |
|  | | | | | | | | | | 2 |  | | MTK | |  | | 8/5/2021 12:40 PM | |  | |  |
|  | I: Did your husband know that you had no peace of mind?  R: He knew.  I: And is he able to see that you now have peace of mind?  R: He knows.  I: Can we say that he is just failing to say that he has noted some change in you since you started receiving counseling?  R: Yes he cannot say it because he knows that he was thinking I was not saying the truth. He has noted change in me compared to how I was in the past.  I: And he sees that you are now free.  R: I am free than in the past. | | | | | | | | | | | | | | | | | | |  | |
|  |  |  |  |  |  |  |  |  |  |  |  |  |  |  |  |  |  |  |  |  |  |
|  |  | | | | | | | | | | | | | | | | | | |  | |
|  | **Codes\\Personal history** | | | | | | | | | | | | | | | | | | |  | |
|  |  |  | No |  | 0.2253 |  | 3 |  | | | | | | | | | | | |  |  |
|  |  |  |  |  |  |  |  |  | | | | | | | | | | | | | |
|  | | | | | | | | | | 1 |  | | MTK | |  | | 8/5/2021 12:03 PM | |  | |  |
|  | I: I would like to know your experiences in this program. Where you were receiving counseling intervention. So I would like to ask you to feel free as we discuss. This is a confidential discussion and that is why we are discussion in a private place so that you should feel free. Whatever you are going to tell us here will be used only for research purposes. The results of this study will help us to come up with plans on how we are supposed to provide care to pregnant women. So our discussion is in two parts; the first part is where I will ask about your demographic information and the last part, I will ask you about the study in general… Firstly, which year were you born?  R: I was born in 2001  I: Do you remember the date and month?  R: On 28th of August.  I: 28th of August… How old are you?  R: 19 years…  I: It is true. In August this year you will be 20 right?  R: Yes.  I: How far did you go with your education?  R: Standard seven.  I: Did anything happen for you to stop at standard seven?  R: I had no financial support to carter for examinations.  I: Okay. Are you married?  R: Yes I am married.  I: How many wives does your husband has?  R: I am his only wife.  I: Are you his first wife?  R: Yes, I am his first wife.  I: And he is your first husband?  R: Yes, my first husband. | | | | | | | | | | | | | | | | | | |  | |
|  |  |  |  |  |  |  |  |  |  |  |  |  |  |  |  |  |  |  |  |  |  |
|  |  |  |  |  |  |  |  |  |  |  |  |  |  |  |  |  |  |  |  |  |  |
|  |  |  |  |  |  |  |  |  |  |  |  |  |  |  |  |  |  |  |  |  |  |
|  |  | | | | | | | | | | | | | | | | | | |  | |
| Formatted Reports\\Coding Summary by File Formatted Report | | | | | | | | | Page 69 of 145 | | | | | | | | | | | |  |
| 8/9/2021 8:08 AM | | | | | | | | | | | | | | | | | | | | |  |
|  | **Classification** |  | **Aggregate** |  | **Coverage** |  | **Number Of Coding References** |  | | **Reference Number** | |  | | **Coded By Initials** | |  | | **Modified On** | |  | |
|  |  |  |  |  |  |  |  |  |  |  |  |  |  |  |  |  |  |  |  |  |  |
|  | | | | | | | | | | 2 |  | | MTK | |  | | 8/5/2021 12:04 PM | |  | |  |
|  | I: How many times have you been pregnant?  R: It was my first pregnancy.  I: Mh… I now see that you are carrying a healthy baby…  R: Yes.  I: When was this baby born?  R: S/he was born at the end of the month of February. | | | | | | | | | | | | | | | | | | |  | |
|  |  | | | | | | | | | | | | | | | | | | |  | |
|  | | | | | | | | | | 3 |  | | MTK | |  | | 8/5/2021 12:06 PM | |  | |  |
|  |  | | | | | | | | | | | | | | | | | | |  | |
|  |  |  |  |  |  |  |  |  |  |  |  |  |  |  |  |  |  |  |  |  |  |
|  |  |  |  |  |  |  |  |  |  |  |  |  |  |  |  |  |  |  |  |  |  |
|  |  | | | | | | | | | | | | | | | | | | |  | |
|  | **Codes\\Place of intervention delivery** | | | | | | | | | | | | | | | | | | |  | |
|  |  |  | No |  | 0.0242 |  | 1 |  | | | | | | | | | | | |  |  |
|  |  |  |  |  |  |  |  |  | | | | | | | | | | | | | |
|  | | | | | | | | | | 1 |  | | MTK | |  | | 8/5/2021 12:35 PM | |  | |  |
|  | I: Now I would like to discuss about the place where the counseling was provided… How did you perceive the venue of the counseling that the counselor was meeting you at your home?  R: It was good because the place was convenient for me. | | | | | | | | | | | | | | | | | | |  | |
|  |  | | | | | | | | | | | | | | | | | | |  | |
|  | **Codes\\Place of intervention delivery\Advantages** | | | | | | | | | | | | | | | | | | |  | |
|  |  |  | No |  | 0.0700 |  | 1 |  | | | | | | | | | | | |  |  |
|  |  |  |  |  |  |  |  |  | | | | | | | | | | | | | |
|  | | | | | | | | | | 1 |  | | MTK | |  | | 8/5/2021 12:37 PM | |  | |  |
|  | I: Now I would like to discuss about the place where the counseling was provided… How did you perceive the venue of the counseling that the counselor was meeting you at your home?  R: It was good because the place was convenient for me.  I: Did you like to receive the counseling session at your home or you would have loved to go somewhere to receive the counseling?  R: It was good that she was coming home because I would not afford to be leaving home to another place or to the health facility here because of transport problems.  I: So, to you the challenge is the transport?  R: Yes.  I: What if transport was provided?  R: Maybe if there can be provision of transport, I can go. | | | | | | | | | | | | | | | | | | |  | |
|  |  |  |  |  |  |  |  |  |  |  |  |  |  |  |  |  |  |  |  |  |  |
|  |  | | | | | | | | | | | | | | | | | | |  | |
|  |  |  |  |  |  |  |  |  |  |  |  |  |  |  |  |  |  |  |  |  |  |
| Formatted Reports\\Coding Summary by File Formatted Report | | | | | | | | | Page 70 of 145 | | | | | | | | | | | |  |
| 8/9/2021 8:08 AM | | | | | | | | | | | | | | | | | | | | |  |
|  | **Classification** |  | **Aggregate** |  | **Coverage** |  | **Number Of Coding References** |  | | **Reference Number** | |  | | **Coded By Initials** | |  | | **Modified On** | |  | |
|  | **Codes\\Place of intervention delivery\Disadvantages** | | | | | | | | | | | | | | | | | | |  | |
|  |  |  | No |  | 0.0122 |  | 1 |  | | | | | | | | | | | |  |  |
|  |  |  |  |  |  |  |  |  | | | | | | | | | | | | | |
|  | | | | | | | | | | 1 |  | | MTK | |  | | 8/5/2021 12:38 PM | |  | |  |
|  | I: Was there any effect for the counseling sessions to be done at your home or community?  R: There was no any effect. | | | | | | | | | | | | | | | | | | |  | |
|  |  | | | | | | | | | | | | | | | | | | |  | |
|  | **Codes\\Recommendations** | | | | | | | | | | | | | | | | | | |  | |
|  |  |  | No |  | 0.0310 |  | 1 |  | | | | | | | | | | | |  |  |
|  |  |  |  |  |  |  |  |  | | | | | | | | | | | | | |
|  | | | | | | | | | | 1 |  | | MTK | |  | | 8/5/2021 12:43 PM | |  | |  |
|  | I: So now the counselor stopped coming to your home because this was a study but you still think that this counseling intervention should continue…  R: Yes it should continue…  I: Every pregnant woman and those with young babies should receive the counseling?  R: Yes they should receive the counseling. | | | | | | | | | | | | | | | | | | |  | |
|  |  | | | | | | | | | | | | | | | | | | |  | |
|  | **Codes\\Recommendations\Areas to be changed** | | | | | | | | | | | | | | | | | | |  | |
|  |  |  | No |  | 0.0558 |  | 1 |  | | | | | | | | | | | |  |  |
|  |  |  |  |  |  |  |  |  | | | | | | | | | | | | | |
|  | | | | | | | | | | 1 |  | | MTK | |  | | 8/5/2021 12:43 PM | |  | |  |
|  | I: So what comments do you have for the counseling that you received?  R: I would like every pregnant woman to be counselled because this counseling is very helpful.  I: So you think there should be no screening for depression but every pregnant woman should be counselled?  R: Yes, every pregnant woman should be counselled because there are some who seem to have no problems but in true sense if they start receiving the counseling, you will note that they have multiple problems. because this counseling is very helpful and it should continue. | | | | | | | | | | | | | | | | | | |  | |
|  |  | | | | | | | | | | | | | | | | | | |  | |
|  | **Codes\\Recommendations\General** | | | | | | | | | | | | | | | | | | |  | |
|  |  |  | No |  | 0.0558 |  | 1 |  | | | | | | | | | | | |  |  |
|  |  |  |  |  |  |  |  |  | | | | | | | | | | | | | |
|  | | | | | | | | | | 1 |  | | MTK | |  | | 8/5/2021 12:42 PM | |  | |  |
|  | I: So what comments do you have for the counseling that you received?  R: I would like every pregnant woman to be counselled because this counseling is very helpful.  I: So you think there should be no screening for depression but every pregnant woman should be counselled?  R: Yes, every pregnant woman should be counselled because there are some who seem to have no problems but in true sense if they start receiving the counseling, you will note that they have multiple problems. because this counseling is very helpful and it should continue. | | | | | | | | | | | | | | | | | | |  | |
|  |  | | | | | | | | | | | | | | | | | | |  | |
|  | **Codes\\THPP providers** | | | | | | | | | | | | | | | | | | |  | |
|  |  |  | No |  | 0.0167 |  | 1 |  | | | | | | | | | | | |  |  |
|  |  |  |  |  |  |  |  |  | | | | | | | | | | | | | |
|  | | | | | | | | | | 1 |  | | MTK | |  | | 8/5/2021 12:34 PM | |  | |  |
|  | I: So what was it that you liked about the counselor who was coming to you to provide the counseling intervention?  R: She was helping me to have helpful thoughts. | | | | | | | | | | | | | | | | | | |  | |
|  |  | | | | | | | | | | | | | | | | | | |  | |
| Formatted Reports\\Coding Summary by File Formatted Report | | | | | | | | | Page 71 of 145 | | | | | | | | | | | |  |
| 8/9/2021 8:08 AM | | | | | | | | | | | | | | | | | | | | |  |
|  | **Classification** |  | **Aggregate** |  | **Coverage** |  | **Number Of Coding References** |  | | **Reference Number** | |  | | **Coded By Initials** | |  | | **Modified On** | |  | |
|  | **Codes\\THPP providers\Characteristics** | | | | | | | | | | | | | | | | | | |  | |
|  |  |  | No |  | 0.0378 |  | 1 |  | | | | | | | | | | | |  |  |
|  |  |  |  |  |  |  |  |  | | | | | | | | | | | | | |
|  | | | | | | | | | | 1 |  | | MTK | |  | | 8/5/2021 12:34 PM | |  | |  |
|  | I: How was her behavior as a counselor… how can you describe her?  R: She was free, open and had good behavior?  I: Can you define good behavior?  R: She was calm and she was not judgmental when delivering sessions, we were discussing in a friendly manner as if we are chatting.  I: Meaning that she was like a friend?  R: She was like a relative and even like my mother. | | | | | | | | | | | | | | | | | | |  | |
|  |  | | | | | | | | | | | | | | | | | | |  | |
|  | **Codes\\THPP providers\Relationship** | | | | | | | | | | | | | | | | | | |  | |
|  |  |  | No |  | 0.0088 |  | 1 |  | | | | | | | | | | | |  |  |
|  |  |  |  |  |  |  |  |  | | | | | | | | | | | | | |
|  | | | | | | | | | | 1 |  | | MTK | |  | | 8/5/2021 12:34 PM | |  | |  |
|  | I: So how can you describe your relationship with her?  R: Our relationship as good. | | | | | | | | | | | | | | | | | | |  | |
|  |  | | | | | | | | | | | | | | | | | | |  | |
|  | **Files\\IDIs\\IDI #15** | | | | | | | | | | | | | | | | | | |  | |
|  | **Code** | | | | | | | | | | | | | | | | | | |  | |
|  | **Codes\\Intervention experience** | | | | | | | | | | | | | | | | | | |  | |
|  |  |  | No |  | 0.1278 |  | 2 |  | | | | | | | | | | | |  |  |
|  |  |  |  |  |  |  |  |  | | | | | | | | | | | | | |
|  | | | | | | | | | | 1 |  | | MTK | |  | | 8/5/2021 12:46 PM | |  | |  |
|  | I: What were you through when you were pregnant?  R: I had ups and downs because there were times when we could disappoint each other… I reached the extent of wishing to kill myself. However, when the counselor started, things changed and I would like this program to continue so that other pregnant mothers can be assisted.  I: You reached the extent of wishing to kill yourself?  R: Yes because of how the situation was and I thought things were not in order.  I: What was the major problem if you may wish to share?  R: The major reason was that we were misunderstanding each other.  I: Why?  R: Over issues of women, promiscuity  I: What about women?  R: He used to have multiple sexual partners and this led to misunderstandings but when the counselor [Name of counselor] started coming, I saw that things started to change.  I: I appreciate that you stopped thinking about killing yourself…  R: And I cannot even think about it. | | | | | | | | | | | | | | | | | | |  | |
|  |  |  |  |  |  |  |  |  |  |  |  |  |  |  |  |  |  |  |  |  |  |
|  |  |  |  |  |  |  |  |  |  |  |  |  |  |  |  |  |  |  |  |  |  |
|  |  | | | | | | | | | | | | | | | | | | |  | |
|  | | | | | | | | | | 2 |  | | MTK | |  | | 8/5/2021 12:49 PM | |  | |  |
|  | I: Okay. Now I would like to ask you about the study whereby the counselor was visiting you at your home. How can you describe the counseling intervention that you had with the counselor?  R: The counseling intervention went on well because I am able to differentiate from the time she first visited me. She can also agree with me that had it been that I was not involved in this counseling intervention, it would either be me losing my life or losing the child. I really feel that things are better for me. Please do not stop providing this counseling intervention to people. It is very helpful and it is unfortunate that you are only targeting pregnant women but there are also other married people who are not pregnant but the can benefit from this intervention. | | | | | | | | | | | | | | | | | | |  | |
|  |  |  |  |  |  |  |  |  |  |  |  |  |  |  |  |  |  |  |  |  |  |
|  |  | | | | | | | | | | | | | | | | | | |  | |
| Formatted Reports\\Coding Summary by File Formatted Report | | | | | | | | | Page 72 of 145 | | | | | | | | | | | |  |
| 8/9/2021 8:08 AM | | | | | | | | | | | | | | | | | | | | |  |
|  | **Classification** |  | **Aggregate** |  | **Coverage** |  | **Number Of Coding References** |  | | **Reference Number** | |  | | **Coded By Initials** | |  | | **Modified On** | |  | |
|  | **Codes\\Intervention experience\Aspects** | | | | | | | | | | | | | | | | | | |  | |
|  |  |  | No |  | 0.0331 |  | 1 |  | | | | | | | | | | | |  |  |
|  |  |  |  |  |  |  |  |  | | | | | | | | | | | | | |
|  | | | | | | | | | | 1 |  | | MTK | |  | | 8/5/2021 12:49 PM | |  | |  |
|  | I: Thank you very much. I understand that this counseling was in different lessons. Which counseling lesson did you find to be most useful?  R: The most helpful lesson was the part of dealing with stress. Of course, we know that there are many stressors but sometimes we fail and reach an extent of thinking about terminating life because of unhelpful thinking. Despite having stressors but we learned how to deal with stressors positively. | | | | | | | | | | | | | | | | | | |  | |
|  |  | | | | | | | | | | | | | | | | | | |  | |
|  | **Codes\\Intervention experience\Barriers** | | | | | | | | | | | | | | | | | | |  | |
|  |  |  | No |  | 0.0111 |  | 1 |  | | | | | | | | | | | |  |  |
|  |  |  |  |  |  |  |  |  | | | | | | | | | | | | | |
|  | | | | | | | | | | 1 |  | | MTK | |  | | 8/5/2021 12:50 PM | |  | |  |
|  | I: Were there any barriers to the counseling that you received?  R: No there wasn’t any because every time she came, we were discussing as required. | | | | | | | | | | | | | | | | | | |  | |
|  |  | | | | | | | | | | | | | | | | | | |  | |
|  | **Codes\\Intervention experience\Dislikes** | | | | | | | | | | | | | | | | | | |  | |
|  |  |  | No |  | 0.0168 |  | 1 |  | | | | | | | | | | | |  |  |
|  |  |  |  |  |  |  |  |  | | | | | | | | | | | | | |
|  | | | | | | | | | | 1 |  | | MTK | |  | | 8/5/2021 12:50 PM | |  | |  |
|  | I: Was there a part that you did not like about the counseling that was provided to you by the counselor?  R: To say the truth, there was no part that was not helpful because everything that we discussed seemed to be helpful. | | | | | | | | | | | | | | | | | | |  | |
|  |  | | | | | | | | | | | | | | | | | | |  | |
|  | **Codes\\Intervention experience\Facilitators** | | | | | | | | | | | | | | | | | | |  | |
|  |  |  | No |  | 0.0243 |  | 1 |  | | | | | | | | | | | |  |  |
|  |  |  |  |  |  |  |  |  | | | | | | | | | | | | | |
|  | | | | | | | | | | 1 |  | | MTK | |  | | 8/5/2021 12:50 PM | |  | |  |
|  | I: So what was the facilitator of the good counseling?  R: What was facilitating the counseling is the way the sessions were conducted, we were chatting like friends, from one woman to the other [mayi ndi mayi mzake] and it was an open and free discussion without any problems besides the challenges that I was going through. | | | | | | | | | | | | | | | | | | |  | |
|  |  | | | | | | | | | | | | | | | | | | |  | |
|  | **Codes\\Intervention experience\Influence on others** | | | | | | | | | | | | | | | | | | |  | |
|  |  |  | No |  | 0.0594 |  | 2 |  | | | | | | | | | | | |  |  |
|  |  |  |  |  |  |  |  |  | | | | | | | | | | | | | |
|  | | | | | | | | | | 1 |  | | MTK | |  | | 8/5/2021 12:51 PM | |  | |  |
|  | I: Can you confirm that everything was going on well during the counseling or there were some challenges?  R: The counseling was going on well because we were able to invite others to join the sessions. | | | | | | | | | | | | | | | | | | |  | |
|  |  | | | | | | | | | | | | | | | | | | |  | |
|  |  |  |  |  |  |  |  |  |  |  |  |  |  |  |  |  |  |  |  |  |  |
| Formatted Reports\\Coding Summary by File Formatted Report | | | | | | | | | Page 73 of 145 | | | | | | | | | | | |  |
| 8/9/2021 8:08 AM | | | | | | | | | | | | | | | | | | | | |  |
|  | **Classification** |  | **Aggregate** |  | **Coverage** |  | **Number Of Coding References** |  | | **Reference Number** | |  | | **Coded By Initials** | |  | | **Modified On** | |  | |
|  |  |  |  |  |  |  |  |  |  |  |  |  |  |  |  |  |  |  |  |  |  |
|  | | | | | | | | | | 2 |  | | MTK | |  | | 8/5/2021 1:05 PM | |  | |  |
|  | I: In summary, what can you say about the counseling intervention that you have received?  R: I strongly accept this intervention and I recommend this type of counseling because I am able to see change in myself because it helped me to replace unhelpful thoughts with helpful thoughts. If possible, if this counseling continues, I should also be able to help other people.  I: You have reached the extent of wishing to assist other people?  R: Yes, so that they should also benefit as I have done.  I: Meaning that you are also a counselor.  R: Yes and I need to help other people as well. | | | | | | | | | | | | | | | | | | |  | |
|  |  |  |  |  |  |  |  |  |  |  |  |  |  |  |  |  |  |  |  |  |  |
|  |  | | | | | | | | | | | | | | | | | | |  | |
|  | **Codes\\Intervention experience\Likes** | | | | | | | | | | | | | | | | | | |  | |
|  |  |  | No |  | 0.0119 |  | 1 |  | | | | | | | | | | | |  |  |
|  |  |  |  |  |  |  |  |  | | | | | | | | | | | | | |
|  | | | | | | | | | | 1 |  | | MTK | |  | | 8/5/2021 12:50 PM | |  | |  |
|  | I: Maybe we should start with the part that you liked the most…  R: The part I liked the most was dealing stress and the health of the mother and child. | | | | | | | | | | | | | | | | | | |  | |
|  |  | | | | | | | | | | | | | | | | | | |  | |
|  | **Codes\\Intervention process** | | | | | | | | | | | | | | | | | | |  | |
|  |  |  | No |  | 0.0045 |  | 1 |  | | | | | | | | | | | |  |  |
|  |  |  |  |  |  |  |  |  | | | | | | | | | | | | | |
|  | | | | | | | | | | 1 |  | | MTK | |  | | 8/5/2021 12:57 PM | |  | |  |
|  | I: Were you able to agree on the next visit date?  R: Yes. | | | | | | | | | | | | | | | | | | |  | |
|  |  | | | | | | | | | | | | | | | | | | |  | |
|  | **Codes\\Intervention process\Feelings** | | | | | | | | | | | | | | | | | | |  | |
|  |  |  | No |  | 0.0657 |  | 2 |  | | | | | | | | | | | |  |  |
|  |  |  |  |  |  |  |  |  | | | | | | | | | | | | | |
|  | | | | | | | | | | 1 |  | | MTK | |  | | 8/5/2021 12:49 PM | |  | |  |
|  | I: Okay. Now I would like to ask you about the study whereby the counselor was visiting you at your home. How can you describe the counseling intervention that you had with the counselor?  R: The counseling intervention went on well because I am able to differentiate from the time she first visited me. She can also agree with me that had it been that I was not involved in this counseling intervention, it would either be me losing my life or losing the child. I really feel that things are better for me. Please do not stop providing this counseling intervention to people. It is very helpful and it is unfortunate that you are only targeting pregnant women but there are also other married people who are not pregnant but the can benefit from this intervention. | | | | | | | | | | | | | | | | | | |  | |
|  |  |  |  |  |  |  |  |  |  |  |  |  |  |  |  |  |  |  |  |  |  |
|  |  | | | | | | | | | | | | | | | | | | |  | |
|  | | | | | | | | | | 2 |  | | MTK | |  | | 8/5/2021 1:05 PM | |  | |  |
|  | I: You look very free, an indicator that all those problems that you had are gone…  R: Those problems are gone. | | | | | | | | | | | | | | | | | | |  | |
|  |  | | | | | | | | | | | | | | | | | | |  | |
|  | **Codes\\Other interesting issues** | | | | | | | | | | | | | | | | | | |  | |
|  |  |  | No |  | 0.0218 |  | 1 |  | | | | | | | | | | | |  |  |
|  |  |  |  |  |  |  |  |  | | | | | | | | | | | | | |
|  | | | | | | | | | | 1 |  | | MTK | |  | | 8/5/2021 12:47 PM | |  | |  |
|  | I: Can you briefly tell me how the delivery went?  R: It was an easy labour and delivery and I delivered normally. I was told to deliver here but the way things were, I delivered at a nearby health facility because had it been that we wanted to come here, I would have delivered on the way. | | | | | | | | | | | | | | | | | | |  | |
|  |  | | | | | | | | | | | | | | | | | | |  | |
| Formatted Reports\\Coding Summary by File Formatted Report | | | | | | | | | Page 74 of 145 | | | | | | | | | | | |  |
| 8/9/2021 8:08 AM | | | | | | | | | | | | | | | | | | | | |  |
|  | **Classification** |  | **Aggregate** |  | **Coverage** |  | **Number Of Coding References** |  | | **Reference Number** | |  | | **Coded By Initials** | |  | | **Modified On** | |  | |
|  | **Codes\\Participation of others** | | | | | | | | | | | | | | | | | | |  | |
|  |  |  | No |  | 0.0199 |  | 1 |  | | | | | | | | | | | |  |  |
|  |  |  |  |  |  |  |  |  | | | | | | | | | | | | | |
|  | | | | | | | | | | 1 |  | | MTK | |  | | 8/5/2021 12:54 PM | |  | |  |
|  | I: So family from your side and from your husband’s side were all present?  R: Yes.  I: It should have been a big group…  R: [Laughter]… It was really interesting because everyone was taking part and that is why I am emphasizing that this program should continue. | | | | | | | | | | | | | | | | | | |  | |
|  |  | | | | | | | | | | | | | | | | | | |  | |
|  | **Codes\\Participation of others\Attitude** | | | | | | | | | | | | | | | | | | |  | |
|  |  |  | No |  | 0.0363 |  | 1 |  | | | | | | | | | | | |  |  |
|  |  |  |  |  |  |  |  |  | | | | | | | | | | | | | |
|  | | | | | | | | | | 1 |  | | MTK | |  | | 8/5/2021 12:55 PM | |  | |  |
|  | I: Were you comfortable expressing your views in the presence of your parents and in-laws?  R: Yes because parents from both sides were supposed to know about what happens and it was interesting that they were accepting that there were some mistakes made.  I: So your parents, relatives and friends were able to accept that there were some problems that led you to think about eliminating your life for example?  R: Yes, and they were able to comment that it was not a good decision. | | | | | | | | | | | | | | | | | | |  | |
|  |  | | | | | | | | | | | | | | | | | | |  | |
|  | **Codes\\Participation of others\Others** | | | | | | | | | | | | | | | | | | |  | |
|  |  |  | No |  | 0.0111 |  | 1 |  | | | | | | | | | | | |  |  |
|  |  |  |  |  |  |  |  |  | | | | | | | | | | | | | |
|  | | | | | | | | | | 1 |  | | MTK | |  | | 8/5/2021 12:53 PM | |  | |  |
|  | I: Who else was present during the counseling sessions apart from your friend?  R: People around me.  I: Like who?  R: My parents… and the in-laws. | | | | | | | | | | | | | | | | | | |  | |
|  |  | | | | | | | | | | | | | | | | | | |  | |
|  | **Codes\\Participation of others\Partners** | | | | | | | | | | | | | | | | | | |  | |
|  |  |  | No |  | 0.0119 |  | 1 |  | | | | | | | | | | | |  |  |
|  |  |  |  |  |  |  |  |  | | | | | | | | | | | | | |
|  | | | | | | | | | | 1 |  | | MTK | |  | | 8/5/2021 12:57 PM | |  | |  |
|  | I: Was your husband present during the sessions?  R: Most of the times he was presents but there were few occasions when he was busy when she did not find him. | | | | | | | | | | | | | | | | | | |  | |
|  |  | | | | | | | | | | | | | | | | | | |  | |
|  |  |  |  |  |  |  |  |  |  |  |  |  |  |  |  |  |  |  |  |  |  |
|  |  |  |  |  |  |  |  |  |  |  |  |  |  |  |  |  |  |  |  |  |  |
| Formatted Reports\\Coding Summary by File Formatted Report | | | | | | | | | Page 75 of 145 | | | | | | | | | | | |  |
| 8/9/2021 8:08 AM | | | | | | | | | | | | | | | | | | | | |  |
|  | **Classification** |  | **Aggregate** |  | **Coverage** |  | **Number Of Coding References** |  | | **Reference Number** | |  | | **Coded By Initials** | |  | | **Modified On** | |  | |
|  | **Codes\\Personal history** | | | | | | | | | | | | | | | | | | |  | |
|  |  |  | No |  | 0.1306 |  | 4 |  | | | | | | | | | | | |  |  |
|  |  |  |  |  |  |  |  |  | | | | | | | | | | | | | |
|  | | | | | | | | | | 1 |  | | MTK | |  | | 8/5/2021 12:45 PM | |  | |  |
|  | I: How old are you? It would be helpful if you remember date month and year when you were born…  R: I was born in 2003 on 3rd June.  I: How old are you now?  R: I am 18 years old.  I: How far did you go with your education?  R: Standard eight.  I: Why did you not proceed with your education?  R: I did not have school fees and I stopped school and got married. So I ask those who are still in school to work hard so that they should not do what I did, they should finish their studies so that they can be working as you are.  I: Meaning that it pains you that you did not go further with your education?  R: It pains me because I wanted to be like you, working helping other people like us but I failed.  I: But life should continue right?  R: Yes.  I: So you said you got married. Is the marriage still on?  R: Yes it is still on.  I: Are you in a single or polygamous marriage?  R: I am alone.  I: You are alone…  R: Yes.  I: Is this your first marriage?  R: Yes.  I: Meaning that you are his first wife and he is your first husband?  R: Yes.  I: Okay. How is the marriage life?  R: It is currently going on well. Maybe it will change in future. | | | | | | | | | | | | | | | | | | |  | |
|  |  |  |  |  |  |  |  |  |  |  |  |  |  |  |  |  |  |  |  |  |  |
|  |  |  |  |  |  |  |  |  |  |  |  |  |  |  |  |  |  |  |  |  |  |
|  |  |  |  |  |  |  |  |  |  |  |  |  |  |  |  |  |  |  |  |  |  |
|  |  | | | | | | | | | | | | | | | | | | |  | |
|  | | | | | | | | | | 2 |  | | MTK | |  | | 8/5/2021 12:46 PM | |  | |  |
|  | I: So I see that you have a baby?  R: Yes.  I: What number is this baby?  R: This is my first born. | | | | | | | | | | | | | | | | | | |  | |
|  |  | | | | | | | | | | | | | | | | | | |  | |
|  | | | | | | | | | | 3 |  | | MTK | |  | | 8/5/2021 12:48 PM | |  | |  |
|  | I: Where do you come from?  R: I come from ……… village.  I: Which TA?  R: TA Kabudula.  . | | | | | | | | | | | | | | | | | | |  | |
|  |  |  |  |  |  |  |  |  |  |  |  |  |  |  |  |  |  |  |  |  |  |
|  |  | | | | | | | | | | | | | | | | | | |  | |
|  | | | | | | | | | | 4 |  | | MTK | |  | | 8/5/2021 12:54 PM | |  | |  |
|  | I: Where are you currently staying?  R: I am staying at my home village.  I: And the husband?  R: He is from the same village.  I: What happened, are you cousins?  R: No we are just from the same village. | | | | | | | | | | | | | | | | | | |  | |
|  |  | | | | | | | | | | | | | | | | | | |  | |
|  |  |  |  |  |  |  |  |  |  |  |  |  |  |  |  |  |  |  |  |  |  |
| Formatted Reports\\Coding Summary by File Formatted Report | | | | | | | | | Page 76 of 145 | | | | | | | | | | | |  |
| 8/9/2021 8:08 AM | | | | | | | | | | | | | | | | | | | | |  |
|  | **Classification** |  | **Aggregate** |  | **Coverage** |  | **Number Of Coding References** |  | | **Reference Number** | |  | | **Coded By Initials** | |  | | **Modified On** | |  | |
|  | **Codes\\Place of intervention delivery\Advantages** | | | | | | | | | | | | | | | | | | |  | |
|  |  |  | No |  | 0.0301 |  | 1 |  | | | | | | | | | | | |  |  |
|  |  |  |  |  |  |  |  |  | | | | | | | | | | | | | |
|  | | | | | | | | | | 1 |  | | MTK | |  | | 8/5/2021 12:53 PM | |  | |  |
|  | R: Yes, it was really happening at my home.  I: What can you say about this counseling intervention being provided at your home?  R: It was very helpful and the intervention also benefitted some of the women who were pregnant because for example I have a friend and I was making sure that when she comes I should invite my friend to be part of the counseling session although she was not in this program. | | | | | | | | | | | | | | | | | | |  | |
|  |  | | | | | | | | | | | | | | | | | | |  | |
|  | **Codes\\Place of intervention delivery\Disadvantages** | | | | | | | | | | | | | | | | | | |  | |
|  |  |  | No |  | 0.0564 |  | 1 |  | | | | | | | | | | | |  |  |
|  |  |  |  |  |  |  |  |  | | | | | | | | | | | | | |
|  | | | | | | | | | | 1 |  | | MTK | |  | | 8/5/2021 12:56 PM | |  | |  |
|  | I: Would you not have loved to go somewhere else for the session; maybe at the home of the counselor or at the clinic?  R: Because the programme was just starting, so, at first, we didn’t know how it was going to work, but we discovered that really counselors are coming to visit us, and I felt bad that she was visiting us in our homes because of distances. For example where I stay is very far from where the counselor stays and we were able to see that she was experiencing challenges in mobility.  I: So on part of the counselor?  R: It was hard for the counselor because she had to use transport for example three hundred kwacha when coming and three hundred kwacha when going.  I: So you were worried about the mobility of the counselor…  R: Yes. | | | | | | | | | | | | | | | | | | |  | |
|  |  |  |  |  |  |  |  |  |  |  |  |  |  |  |  |  |  |  |  |  |  |
|  |  | | | | | | | | | | | | | | | | | | |  | |
|  | **Codes\\Recommendations\General** | | | | | | | | | | | | | | | | | | |  | |
|  |  |  | No |  | 0.0443 |  | 1 |  | | | | | | | | | | | |  |  |
|  |  |  |  |  |  |  |  |  | | | | | | | | | | | | | |
|  | | | | | | | | | | 1 |  | | MTK | |  | | 8/5/2021 1:05 PM | |  | |  |
|  | I: In summary, what can you say about the counseling intervention that you have received?  R: I strongly accept this intervention and I recommend this type of counseling because I am able to see change in myself because it helped me to replace unhelpful thoughts with helpful thoughts. If possible, if this counseling continues, I should also be able to help other people.  I: You have reached the extent of wishing to assist other people?  R: Yes, so that they should also benefit as I have done.  I: Meaning that you are also a counselor.  R: Yes and I need to help other people as well. | | | | | | | | | | | | | | | | | | |  | |
|  |  |  |  |  |  |  |  |  |  |  |  |  |  |  |  |  |  |  |  |  |  |
|  |  | | | | | | | | | | | | | | | | | | |  | |
|  | **Codes\\Recommendations\Intervention providers** | | | | | | | | | | | | | | | | | | |  | |
|  |  |  | No |  | 0.0200 |  | 1 |  | | | | | | | | | | | |  |  |
|  |  |  |  |  |  |  |  |  | | | | | | | | | | | | | |
|  | | | | | | | | | | 1 |  | | MTK | |  | | 8/5/2021 1:04 PM | |  | |  |
|  | I: In this case, can you confirm that providing counseling at home is a welcome development if counselors are provided with transport means?  R: Yes, they should be given means of transport but if not, then the counseling should be provided in the health facilities. | | | | | | | | | | | | | | | | | | |  | |
|  |  | | | | | | | | | | | | | | | | | | |  | |
|  |  |  |  |  |  |  |  |  |  |  |  |  |  |  |  |  |  |  |  |  |  |
| Formatted Reports\\Coding Summary by File Formatted Report | | | | | | | | | Page 77 of 145 | | | | | | | | | | | |  |
| 8/9/2021 8:08 AM | | | | | | | | | | | | | | | | | | | | |  |
|  | **Classification** |  | **Aggregate** |  | **Coverage** |  | **Number Of Coding References** |  | | **Reference Number** | |  | | **Coded By Initials** | |  | | **Modified On** | |  | |
|  | **Codes\\Recommendations\Place of delivery** | | | | | | | | | | | | | | | | | | |  | |
|  |  |  | No |  | 0.0200 |  | 1 |  | | | | | | | | | | | |  |  |
|  |  |  |  |  |  |  |  |  | | | | | | | | | | | | | |
|  | | | | | | | | | | 1 |  | | MTK | |  | | 8/5/2021 1:04 PM | |  | |  |
|  | I: In this case, can you confirm that providing counseling at home is a welcome development if counselors are provided with transport means?  R: Yes, they should be given means of transport but if not, then the counseling should be provided in the health facilities. | | | | | | | | | | | | | | | | | | |  | |
|  |  | | | | | | | | | | | | | | | | | | |  | |
|  | **Codes\\THPP providers\Characteristics** | | | | | | | | | | | | | | | | | | |  | |
|  |  |  | No |  | 0.0762 |  | 1 |  | | | | | | | | | | | |  |  |
|  |  |  |  |  |  |  |  |  | | | | | | | | | | | | | |
|  | | | | | | | | | | 1 |  | | MTK | |  | | 8/5/2021 12:52 PM | |  | |  |
|  | I: Thank you very much. We are also going to look into that. Now, being a woman who was screened for depression and the counselor [Name of counselor] had to come to your home to be helping you. What did you like about the counselor as a person?  R: The way she was presenting herself to me because we seemed as if we knew each other before. It was not like we just met for the counseling intervention and many people in the village thought she was my relative but when I told them that “we just met in a counselor – client relationship” they said “she is a very good and open person.”  I: Mh… you have said that she was a good person. Can you tell me more about that?  R: I saw that she was a good person because when she found us eating, she never refused the food and whenever she was coming home, she was making sure she brings me something and if I had something, I was able to give her. In this case, I did not want anyone else to talk bad about her because it means that person does not appreciate good things. | | | | | | | | | | | | | | | | | | |  | |
|  |  |  |  |  |  |  |  |  |  |  |  |  |  |  |  |  |  |  |  |  |  |
|  |  | | | | | | | | | | | | | | | | | | |  | |
|  | **Codes\\THPP providers\Relationship** | | | | | | | | | | | | | | | | | | |  | |
|  |  |  | No |  | 0.0419 |  | 2 |  | | | | | | | | | | | |  |  |
|  |  |  |  |  |  |  |  |  | | | | | | | | | | | | | |
|  | | | | | | | | | | 1 |  | | MTK | |  | | 8/5/2021 12:52 PM | |  | |  |
|  | I: Based on your explanation, it seems you loved this person…  R: I really loved this person and I told her that after terminating the counseling sessions, I will be the one visiting her in her home. | | | | | | | | | | | | | | | | | | |  | |
|  |  | | | | | | | | | | | | | | | | | | |  | |
|  | | | | | | | | | | 2 |  | | MTK | |  | | 8/5/2021 12:52 PM | |  | |  |
|  | I: Meaning that you built a good relationship?  R: A very good relationship.  I: Can you confirm that you had good relationship with the counselor when she was coming to your home for counseling sessions?  R: Yes it was very good. Even when I was not there at home, she was making sure that she waits for me and when I came back we were able to do the session. | | | | | | | | | | | | | | | | | | |  | |
|  |  | | | | | | | | | | | | | | | | | | |  | |
|  |  |  |  |  |  |  |  |  |  |  |  |  |  |  |  |  |  |  |  |  |  |
|  |  |  |  |  |  |  |  |  |  |  |  |  |  |  |  |  |  |  |  |  |  |
|  |  |  |  |  |  |  |  |  |  |  |  |  |  |  |  |  |  |  |  |  |  |
| Formatted Reports\\Coding Summary by File Formatted Report | | | | | | | | | Page 78 of 145 | | | | | | | | | | | |  |
| 8/9/2021 8:08 AM | | | | | | | | | | | | | | | | | | | | |  |
|  | **Classification** |  | **Aggregate** |  | **Coverage** |  | **Number Of Coding References** |  | | **Reference Number** | |  | | **Coded By Initials** | |  | | **Modified On** | |  | |
|  | **Files\\IDIs\\IDI #17** | | | | | | | | | | | | | | | | | | |  | |
|  | **Code** | | | | | | | | | | | | | | | | | | |  | |
|  | **Codes\\Intervention experience** | | | | | | | | | | | | | | | | | | |  | |
|  |  |  | No |  | 0.0723 |  | 1 |  | | | | | | | | | | | |  |  |
|  |  |  |  |  |  |  |  |  | | | | | | | | | | | | | |
|  | | | | | | | | | | 1 |  | | MTK | |  | | 8/5/2021 1:10 PM | |  | |  |
|  | I: Thank you very much. The number is complete. Now we are going to the second part of our discussion. Can you tell me more about the counseling intervention that you were receiving at your home whereby that counselor was coming to your home to provide you with the counseling intervention?  R: I saw that the intervention was very powerful because I was afraid and stressed thinking that “how am I going to deliver?” Before the counselor started coming to my home, I was very worried and I had stress but after receiving the counseling intervention I am very fine. And am very thankfull, I wish this programme continues and help others  I: Can you tell me what your problem was that led to the depression?  R: My main worry was related to my delivery because most of the times I was sick. I used to be on and off sicknesses so I was worried if I will make it during delivery. | | | | | | | | | | | | | | | | | | |  | |
|  |  |  |  |  |  |  |  |  |  |  |  |  |  |  |  |  |  |  |  |  |  |
|  |  | | | | | | | | | | | | | | | | | | |  | |
|  | **Codes\\Intervention experience\Barriers** | | | | | | | | | | | | | | | | | | |  | |
|  |  |  | No |  | 0.0166 |  | 2 |  | | | | | | | | | | | |  |  |
|  |  |  |  |  |  |  |  |  | | | | | | | | | | | | | |
|  | | | | | | | | | | 1 |  | | MTK | |  | | 8/5/2021 1:12 PM | |  | |  |
|  | I: Were there any barriers to the counseling?  R: No. | | | | | | | | | | | | | | | | | | |  | |
|  |  | | | | | | | | | | | | | | | | | | |  | |
|  | | | | | | | | | | 2 |  | | MTK | |  | | 8/5/2021 1:23 PM | |  | |  |
|  | I: Can we say that you had no challenges or were there any challenges having the counseling intervention at your home?  R: There were no challenges. | | | | | | | | | | | | | | | | | | |  | |
|  |  | | | | | | | | | | | | | | | | | | |  | |
|  | **Codes\\Intervention experience\Dislikes** | | | | | | | | | | | | | | | | | | |  | |
|  |  |  | No |  | 0.0060 |  | 1 |  | | | | | | | | | | | |  |  |
|  |  |  |  |  |  |  |  |  | | | | | | | | | | | | | |
|  | | | | | | | | | | 1 |  | | MTK | |  | | 8/5/2021 1:12 PM | |  | |  |
|  | I: Was there anything that you did not like about the counseling?  R: No. | | | | | | | | | | | | | | | | | | |  | |
|  |  | | | | | | | | | | | | | | | | | | |  | |
|  | **Codes\\Intervention experience\Facilitators** | | | | | | | | | | | | | | | | | | |  | |
|  |  |  | No |  | 0.0248 |  | 1 |  | | | | | | | | | | | |  |  |
|  |  |  |  |  |  |  |  |  | | | | | | | | | | | | | |
|  | | | | | | | | | | 1 |  | | MTK | |  | | 8/5/2021 1:12 PM | |  | |  |
|  | I: Okay. So because you have said that there were no challenges, can you mention some of the things that made the counseling to go on well?  R: What helped the most that the counseling intervention should go on well was that she was coming in the afternoon when I had the opportunity to meet with her. | | | | | | | | | | | | | | | | | | |  | |
|  |  | | | | | | | | | | | | | | | | | | |  | |
|  |  |  |  |  |  |  |  |  |  |  |  |  |  |  |  |  |  |  |  |  |  |
| Formatted Reports\\Coding Summary by File Formatted Report | | | | | | | | | Page 79 of 145 | | | | | | | | | | | |  |
| 8/9/2021 8:08 AM | | | | | | | | | | | | | | | | | | | | |  |
|  | **Classification** |  | **Aggregate** |  | **Coverage** |  | **Number Of Coding References** |  | | **Reference Number** | |  | | **Coded By Initials** | |  | | **Modified On** | |  | |
|  |  | | | | | | | | | | | | | | | | | | |  | |
|  |  | | | | | | | | | | | | | | | | | | |  | |
|  | **Codes\\Intervention experience\Likes** | | | | | | | | | | | | | | | | | | |  | |
|  |  |  | No |  | 0.0488 |  | 2 |  | | | | | | | | | | | |  |  |
|  |  |  |  |  |  |  |  |  | | | | | | | | | | | | | |
|  | | | | | | | | | | 1 |  | | MTK | |  | | 8/5/2021 1:10 PM | |  | |  |
|  | I: I understand that the counselor visited you several times…So which part of the counseling intervention did you like the most?  R: The part of the counseling that I liked the most was about resting because I was overworking without resting but when she came with the counseling intervention and when I was able to rest, I saw that things were going on well with me. | | | | | | | | | | | | | | | | | | |  | |
|  |  | | | | | | | | | | | | | | | | | | |  | |
|  | | | | | | | | | | 2 |  | | MTK | |  | | 8/5/2021 1:12 PM | |  | |  |
|  | I: So apart from resting… what were the other things that you liked?  R: The other thing was that she taught me about eating six food groups and my husband was able to bring me the foods after learning from these lessons. | | | | | | | | | | | | | | | | | | |  | |
|  |  | | | | | | | | | | | | | | | | | | |  | |
|  | **Codes\\Intervention process\Feelings** | | | | | | | | | | | | | | | | | | |  | |
|  |  |  | No |  | 0.0301 |  | 1 |  | | | | | | | | | | | |  |  |
|  |  |  |  |  |  |  |  |  | | | | | | | | | | | | | |
|  | | | | | | | | | | 1 |  | | MTK | |  | | 8/5/2021 1:41 PM | |  | |  |
|  | I: Having received this counseling intervention, how do you feel right now?  R:I feel very good and this counseling intervention is very helpful and it needs to continue because I am able to differentiate how I was before I started receiving the counseling.  I: Meaning that things seem to have changed for you since you started receiving the counseling?  R: Yes. | | | | | | | | | | | | | | | | | | |  | |
|  |  | | | | | | | | | | | | | | | | | | |  | |
|  | **Codes\\Other interesting issues** | | | | | | | | | | | | | | | | | | |  | |
|  |  |  | No |  | 0.0470 |  | 1 |  | | | | | | | | | | | |  |  |
|  |  |  |  |  |  |  |  |  | | | | | | | | | | | | | |
|  | | | | | | | | | | 1 |  | | MTK | |  | | 8/5/2021 1:08 PM | |  | |  |
|  | I: How did the delivery go?  R: The delivery went on very well and I did not know that I could be able to live a normal life again but I am thankful to this program.  I: I meant that were there some complications or was it a normal delivery?  R: It was a normal delivery.  I: So can you tell me your experiences with the pregnancy of this child?  R: I had a lot of stress and a lot of problems but I had nowhere to go to address my problems but when I heard about this program I said that “this is my right opportunity to deal with my problems that I was going through. | | | | | | | | | | | | | | | | | | |  | |
|  |  |  |  |  |  |  |  |  |  |  |  |  |  |  |  |  |  |  |  |  |  |
|  |  | | | | | | | | | | | | | | | | | | |  | |
|  |  |  |  |  |  |  |  |  |  |  |  |  |  |  |  |  |  |  |  |  |  |
| Formatted Reports\\Coding Summary by File Formatted Report | | | | | | | | | Page 80 of 145 | | | | | | | | | | | |  |
| 8/9/2021 8:08 AM | | | | | | | | | | | | | | | | | | | | |  |
|  | **Classification** |  | **Aggregate** |  | **Coverage** |  | **Number Of Coding References** |  | | **Reference Number** | |  | | **Coded By Initials** | |  | | **Modified On** | |  | |
|  | **Codes\\Participation of others\Others** | | | | | | | | | | | | | | | | | | |  | |
|  |  |  | No |  | 0.0313 |  | 2 |  | | | | | | | | | | | |  |  |
|  |  |  |  |  |  |  |  |  | | | | | | | | | | | | | |
|  | | | | | | | | | | 1 |  | | MTK | |  | | 8/5/2021 1:11 PM | |  | |  |
|  | I: Was there anyone who was supporting you, to relieve you from house chores?  R: Yes there was one.  I: Who was this one?  R: A girl was helping me.  I: What about your husband, was he not assisting you?  R: He was assisting me. | | | | | | | | | | | | | | | | | | |  | |
|  |  | | | | | | | | | | | | | | | | | | |  | |
|  | | | | | | | | | | 2 |  | | MTK | |  | | 8/5/2021 1:21 PM | |  | |  |
|  | I: Okay. So how many people were present during the counseling session?  R: When she came, I was with my husband, my mother in – law and my sisters. | | | | | | | | | | | | | | | | | | |  | |
|  |  | | | | | | | | | | | | | | | | | | |  | |
|  | **Codes\\Participation of others\Partners** | | | | | | | | | | | | | | | | | | |  | |
|  |  |  | No |  | 0.0191 |  | 1 |  | | | | | | | | | | | |  |  |
|  |  |  |  |  |  |  |  |  | | | | | | | | | | | | | |
|  | | | | | | | | | | 1 |  | | MTK | |  | | 8/5/2021 1:11 PM | |  | |  |
|  | I: Was there anyone who was supporting you, to relieve you from house chores?  R: Yes there was one.  I: Who was this one?  R: A girl was helping me.  I: What about your husband, was he not assisting you?  R: He was assisting me. | | | | | | | | | | | | | | | | | | |  | |
|  |  | | | | | | | | | | | | | | | | | | |  | |
|  | **Codes\\Participation of others\Support** | | | | | | | | | | | | | | | | | | |  | |
|  |  |  | No |  | 0.0613 |  | 2 |  | | | | | | | | | | | |  |  |
|  |  |  |  |  |  |  |  |  | | | | | | | | | | | | | |
|  | | | | | | | | | | 1 |  | | MTK | |  | | 8/5/2021 1:22 PM | |  | |  |
|  | I: So can you briefly describe how the discussion was with your husband, mother in – law and sisters present during the session?  R: It was good because when I failed to answer some of the questions, my mother in – law was able to respond. | | | | | | | | | | | | | | | | | | |  | |
|  |  | | | | | | | | | | | | | | | | | | |  | |
|  | | | | | | | | | | 2 |  | | MTK | |  | | 8/5/2021 1:23 PM | |  | |  |
|  | I: Was it good for the other people to be around when you were expressing your experiences with stress?  R: It was good so that they should know that I had stress.  I: You said that your sister help you with some chores?  R: Yes.  I: Meaning that if they had known what was stressing you they would also help?  R: Yes it is true.  I: Do you think they were able to assist you because they were present during the counseling sessions?  R: Yes they were assisting me with drawing water, cooking and all that. | | | | | | | | | | | | | | | | | | |  | |
|  |  |  |  |  |  |  |  |  |  |  |  |  |  |  |  |  |  |  |  |  |  |
|  |  | | | | | | | | | | | | | | | | | | |  | |
|  |  |  |  |  |  |  |  |  |  |  |  |  |  |  |  |  |  |  |  |  |  |
| Formatted Reports\\Coding Summary by File Formatted Report | | | | | | | | | Page 81 of 145 | | | | | | | | | | | |  |
| 8/9/2021 8:08 AM | | | | | | | | | | | | | | | | | | | | |  |
|  | **Classification** |  | **Aggregate** |  | **Coverage** |  | **Number Of Coding References** |  | | **Reference Number** | |  | | **Coded By Initials** | |  | | **Modified On** | |  | |
|  | **Codes\\Personal history** | | | | | | | | | | | | | | | | | | |  | |
|  |  |  | No |  | 0.1419 |  | 3 |  | | | | | | | | | | | |  |  |
|  |  |  |  |  |  |  |  |  | | | | | | | | | | | | | |
|  | | | | | | | | | | 1 |  | | MTK | |  | | 8/5/2021 1:07 PM | |  | |  |
|  | R: Okay, I was born in 1996. I am currently 26 years.  I: What date were you born?  R: I was born on 2nd February.  I: How far did you go with your education?  R: I went up to standard eight.  I: Can you tell me why you did not go further with your education?  R: I was not able to find money to pay of examinations.  I: Meaning that if you had money to pay for the examinations you would have continued with your education?  R: Yes I would have continued. | | | | | | | | | | | | | | | | | | |  | |
|  |  |  |  |  |  |  |  |  |  |  |  |  |  |  |  |  |  |  |  |  |  |
|  |  | | | | | | | | | | | | | | | | | | |  | |
|  | | | | | | | | | | 2 |  | | MTK | |  | | 8/5/2021 1:08 PM | |  | |  |
|  | I: We may want to find things in life but based on the situations we are going through we tend to fail to do some of the things… Fine. So I would like to know; are you currently married?  R: Yes, I am married.  I: Is this your first husband?  R: Yes.  I: And are you his first wife?  R: Yes.  I: How many wives are you for this husband?  R: I am alone.  I: Should we say that the husband did not have another wife before?  R: No.  I: And you did not get married before?  R: No.  I: Thank you very much. As I said earlier on, we knew you when you came for antenatal clinic visit. What number is this pregnancy?  R: This was my second pregnancy. | | | | | | | | | | | | | | | | | | |  | |
|  |  |  |  |  |  |  |  |  |  |  |  |  |  |  |  |  |  |  |  |  |  |
|  |  |  |  |  |  |  |  |  |  |  |  |  |  |  |  |  |  |  |  |  |  |
|  |  | | | | | | | | | | | | | | | | | | |  | |
|  | | | | | | | | | | 3 |  | | MTK | |  | | 8/5/2021 1:09 PM | |  | |  |
|  | I: Thank you very much. We are going to look into that in detail. So you said that this is your second child…?  R: Yes.  I: Was this your second pregnancy or there were some losses?  R: No this was my second pregnancy.  I: Thank you. Which village are you currently staying?  R: I stay in ……… village.  I: Which TA?  R: TA Kabudula. | | | | | | | | | | | | | | | | | | |  | |
|  |  |  |  |  |  |  |  |  |  |  |  |  |  |  |  |  |  |  |  |  |  |
|  |  |  |  |  |  |  |  |  |  |  |  |  |  |  |  |  |  |  |  |  |  |
|  |  | | | | | | | | | | | | | | | | | | |  | |
|  |  |  |  |  |  |  |  |  |  |  |  |  |  |  |  |  |  |  |  |  |  |
|  |  |  |  |  |  |  |  |  |  |  |  |  |  |  |  |  |  |  |  |  |  |
| Formatted Reports\\Coding Summary by File Formatted Report | | | | | | | | | Page 82 of 145 | | | | | | | | | | | |  |
| 8/9/2021 8:08 AM | | | | | | | | | | | | | | | | | | | | |  |
|  | **Classification** |  | **Aggregate** |  | **Coverage** |  | **Number Of Coding References** |  | | **Reference Number** | |  | | **Coded By Initials** | |  | | **Modified On** | |  | |
|  | **Codes\\Place of intervention delivery** | | | | | | | | | | | | | | | | | | |  | |
|  |  |  | No |  | 0.0499 |  | 1 |  | | | | | | | | | | | |  |  |
|  |  |  |  |  |  |  |  |  | | | | | | | | | | | | | |
|  | | | | | | | | | | 1 |  | | MTK | |  | | 8/5/2021 1:20 PM | |  | |  |
|  | I: Thank you. Now I would like us to discuss about the place where you were meeting for the counseling intervention… I would like you to tell me how you perceived the place where you were receiving the counseling? Can you say that it was good for the counselor to be coming to your home to provide the counseling intervention?  R: It was good.  I: What was good about providing the counseling at your home?  R: Can you repeat the question?  I: You have said that it was good for the counselor to be coming to your home for counseling. Why do you think it was good?  R: It was good because she assisted me. | | | | | | | | | | | | | | | | | | |  | |
|  |  |  |  |  |  |  |  |  |  |  |  |  |  |  |  |  |  |  |  |  |  |
|  |  | | | | | | | | | | | | | | | | | | |  | |
|  | **Codes\\Place of intervention delivery\Disadvantages** | | | | | | | | | | | | | | | | | | |  | |
|  |  |  | No |  | 0.0160 |  | 1 |  | | | | | | | | | | | |  |  |
|  |  |  |  |  |  |  |  |  | | | | | | | | | | | | | |
|  | | | | | | | | | | 1 |  | | MTK | |  | | 8/5/2021 1:20 PM | |  | |  |
|  | I: Let me clarify it in this way; had it been that they said “this counseling should be provided at the clinic here…” would you manage to be coming here for counseling?  R: No I would not manage. | | | | | | | | | | | | | | | | | | |  | |
|  |  | | | | | | | | | | | | | | | | | | |  | |
|  | **Codes\\Recommendations\Areas to be changed** | | | | | | | | | | | | | | | | | | |  | |
|  |  |  | No |  | 0.0459 |  | 2 |  | | | | | | | | | | | |  |  |
|  |  |  |  |  |  |  |  |  | | | | | | | | | | | | | |
|  | | | | | | | | | | 1 |  | | MTK | |  | | 8/5/2021 1:23 PM | |  | |  |
|  | I: Okay… maybe before we finish… is there anything you would have wished to be changed in this counseling intervention?  R: [No response]  I: I understand you have said good things about this study…  R: Yes.  I: Do you think there are other things that need to be changed in this intervention?  R: The most important thing is that you should make sure that we have basic needs in our lives so that we should be able to implement the program. | | | | | | | | | | | | | | | | | | |  | |
|  |  |  |  |  |  |  |  |  |  |  |  |  |  |  |  |  |  |  |  |  |  |
|  |  | | | | | | | | | | | | | | | | | | |  | |
|  | | | | | | | | | | 2 |  | | MTK | |  | | 8/5/2021 1:41 PM | |  | |  |
|  | I: That is on your part… Is there anything you would like to be changed about the counseling intervention?  R: No. | | | | | | | | | | | | | | | | | | |  | |
|  |  | | | | | | | | | | | | | | | | | | |  | |
|  | **Codes\\Recommendations\Place of delivery** | | | | | | | | | | | | | | | | | | |  | |
|  |  |  | No |  | 0.0134 |  | 1 |  | | | | | | | | | | | |  |  |
|  |  |  |  |  |  |  |  |  | | | | | | | | | | | | | |
|  | | | | | | | | | | 1 |  | | MTK | |  | | 8/5/2021 1:21 PM | |  | |  |
|  | I: Can you confirm that you saw that it was very important for the counseling to be conducted at home?  R: Yes it was important that it should be conducted at home. | | | | | | | | | | | | | | | | | | |  | |
|  |  | | | | | | | | | | | | | | | | | | |  | |
|  |  |  |  |  |  |  |  |  |  |  |  |  |  |  |  |  |  |  |  |  |  |
| Formatted Reports\\Coding Summary by File Formatted Report | | | | | | | | | Page 83 of 145 | | | | | | | | | | | |  |
| 8/9/2021 8:08 AM | | | | | | | | | | | | | | | | | | | | |  |
|  | **Classification** |  | **Aggregate** |  | **Coverage** |  | **Number Of Coding References** |  | | **Reference Number** | |  | | **Coded By Initials** | |  | | **Modified On** | |  | |
|  | **Codes\\THPP providers\Characteristics** | | | | | | | | | | | | | | | | | | |  | |
|  |  |  | No |  | 0.0442 |  | 1 |  | | | | | | | | | | | |  |  |
|  |  |  |  |  |  |  |  |  | | | | | | | | | | | | | |
|  | | | | | | | | | | 1 |  | | MTK | |  | | 8/5/2021 1:13 PM | |  | |  |
|  | I: So what did you like about your counselor as a person?  R: [Chuckles]…  I: You are laughing…  R: [Laughter]… I liked her because she was teaching me good things  I: Apart from the good things that she was teaching you, what did you like about her as a person? Some people like a person because of the things that she does… for example, some people like teachers because of the way they teach… what was it that you liked about the counselor?  R: I liked the counselor because she was ever cheerful.  I: So she was cheerful…  R: Yes. | | | | | | | | | | | | | | | | | | |  | |
|  |  |  |  |  |  |  |  |  |  |  |  |  |  |  |  |  |  |  |  |  |  |
|  |  | | | | | | | | | | | | | | | | | | |  | |
|  | **Codes\\THPP providers\Relationship** | | | | | | | | | | | | | | | | | | |  | |
|  |  |  | No |  | 0.0604 |  | 1 |  | | | | | | | | | | | |  |  |
|  |  |  |  |  |  |  |  |  | | | | | | | | | | | | | |
|  | | | | | | | | | | 1 |  | | MTK | |  | | 8/5/2021 1:20 PM | |  | |  |
|  | I: What relationship can you describe between you and the counselor?  R: We built a good relationship because we did not know each other before but during the counseling intervention we became friends.  I: So I understand that the counseling relationship was terminated but I would like to know if the relationship is still there…  R: The relationship is still there. As I said that we knew each other because of the counseling intervention that she was providing me. We continue to be friends and if I can meet her I can hag her as a good friend.  I: You cannot change your route…?  R: Not at all. She gave me good counseling.  I: We will continue to hag each other in the near future after the COVID 19 right?  R: Okay… [Chuckles]… | | | | | | | | | | | | | | | | | | |  | |
|  |  |  |  |  |  |  |  |  |  |  |  |  |  |  |  |  |  |  |  |  |  |
|  |  | | | | | | | | | | | | | | | | | | |  | |
|  | **Files\\IDIs\\IDI #18** | | | | | | | | | | | | | | | | | | |  | |
|  | **Code** | | | | | | | | | | | | | | | | | | |  | |
|  | **Codes\\Intervention experience** | | | | | | | | | | | | | | | | | | |  | |
|  |  |  | No |  | 0.1760 |  | 5 |  | | | | | | | | | | | |  |  |
|  |  |  |  |  |  |  |  |  | | | | | | | | | | | | | |
|  | | | | | | | | | | 1 |  | | MTK | |  | | 8/5/2021 1:55 PM | |  | |  |
|  | I: You have a very beautiful child… can you explain what you were going through/ was happening to you the time you were pregnant?  R: For this child to be like this, it is because of the counselor whom you sent us. She helped me to deal with my stresses and that is why I have a child like this one.  I: Mh…  R: For me to stop having the thoughts that I had, it was because of that woman. I had unhelpful thoughts. I was not able to eat, I was not able to associate with my friends and I was not able to share my problems with my friends. | | | | | | | | | | | | | | | | | | |  | |
|  |  | | | | | | | | | | | | | | | | | | |  | |
|  | | | | | | | | | | 2 |  | | MTK | |  | | 8/5/2021 1:55 PM | |  | |  |
|  | I: Meaning that when you were pregnant, you were depressed and that the counseling intervention that you received helped you to deal with the depression?  R: Yes. | | | | | | | | | | | | | | | | | | |  | |
|  |  | | | | | | | | | | | | | | | | | | |  | |
| Formatted Reports\\Coding Summary by File Formatted Report | | | | | | | | | Page 84 of 145 | | | | | | | | | | | |  |
| 8/9/2021 8:08 AM | | | | | | | | | | | | | | | | | | | | |  |
|  | **Classification** |  | **Aggregate** |  | **Coverage** |  | **Number Of Coding References** |  | | **Reference Number** | |  | | **Coded By Initials** | |  | | **Modified On** | |  | |
|  |  |  |  |  |  |  |  |  |  |  |  |  |  |  |  |  |  |  |  |  |  |
|  | | | | | | | | | | 3 |  | | MTK | |  | | 8/5/2021 1:56 PM | |  | |  |
|  | I: Thank you very much. Now I would like us to discuss about the counseling intervention that you received in this study in which you took part. I would like you to tell me more about your experiences with this Thinking Healthy Program?  R: I have benefitted a lot in this. I am able to do things that I was not able to do before. I am able to eat, chat and feel free with relatives. I am able to eat different food groups.  I: Meaning that you were able to do all these after receiving the counseling intervention?  R: Yes.  I: What was happening before the counseling intervention was received?  R: I was not thinking healthy, my heart was heavy and I would have killed myself.  I: Okay. So because of the counseling you feel that you changed?  R: Yes. | | | | | | | | | | | | | | | | | | |  | |
|  |  |  |  |  |  |  |  |  |  |  |  |  |  |  |  |  |  |  |  |  |  |
|  |  | | | | | | | | | | | | | | | | | | |  | |
|  | | | | | | | | | | 4 |  | | MTK | |  | | 8/5/2021 2:23 PM | |  | |  |
|  | I: Thank you very much. I understand that the counselor was coming to your home to provide counseling to you?  R: Yes.  I: Were there any challenges receiving the counseling at your home?  R: There were no any challenges because we were discussing in the house the two of us.  I: Meaning that you confirm that it was good receiving the counseling at home?  R: Yes. | | | | | | | | | | | | | | | | | | |  | |
|  |  | | | | | | | | | | | | | | | | | | |  | |
|  | | | | | | | | | | 5 |  | | MTK | |  | | 8/5/2021 2:27 PM | |  | |  |
|  | I: So you have told me that you visit each other with the counselor after the termination of the counseling relationship. Can we confirm that counseling helped you?  R: Yes. | | | | | | | | | | | | | | | | | | |  | |
|  |  | | | | | | | | | | | | | | | | | | |  | |
|  | **Codes\\Intervention experience\Aspects** | | | | | | | | | | | | | | | | | | |  | |
|  |  |  | No |  | 0.0509 |  | 1 |  | | | | | | | | | | | |  |  |
|  |  |  |  |  |  |  |  |  | | | | | | | | | | | | | |
|  | | | | | | | | | | 1 |  | | MTK | |  | | 8/5/2021 2:02 PM | |  | |  |
|  | I: So the counseling had different sections; which part of the counseling intervention do you think helped you the most and it helped you think about what you were discussing?  R: The way the sessions were conducted because it helped me to feel better.  I: There were different sections; about healthy eating…  R: Unhelpful thoughts…  I: I would like you to tell me one section that helped you the most…  R: The section that helped me the most was about food and relationship with relatives.  I: So what else did you like about this counseling?  R: I changed how I was feeling. | | | | | | | | | | | | | | | | | | |  | |
|  |  |  |  |  |  |  |  |  |  |  |  |  |  |  |  |  |  |  |  |  |  |
|  |  | | | | | | | | | | | | | | | | | | |  | |
|  | **Codes\\Intervention experience\Dislikes** | | | | | | | | | | | | | | | | | | |  | |
|  |  |  | No |  | 0.0214 |  | 2 |  | | | | | | | | | | | |  |  |
|  |  |  |  |  |  |  |  |  | | | | | | | | | | | | | |
|  | | | | | | | | | | 1 |  | | MTK | |  | | 8/5/2021 2:02 PM | |  | |  |
|  | I: Okay. Can you tell me what areas you did not like about the counseling that you were provided at your home…?  R: Nothing. | | | | | | | | | | | | | | | | | | |  | |
|  |  | | | | | | | | | | | | | | | | | | |  | |
|  | | | | | | | | | | 2 |  | | MTK | |  | | 8/5/2021 2:03 PM | |  | |  |
|  | I: So that is about you. Wat about the counseling… what was it that you did not like about the counseling?  R: Nothing. | | | | | | | | | | | | | | | | | | |  | |
|  |  | | | | | | | | | | | | | | | | | | |  | |
|  |  |  |  |  |  |  |  |  |  |  |  |  |  |  |  |  |  |  |  |  |  |
| Formatted Reports\\Coding Summary by File Formatted Report | | | | | | | | | Page 85 of 145 | | | | | | | | | | | |  |
| 8/9/2021 8:08 AM | | | | | | | | | | | | | | | | | | | | |  |
|  | **Classification** |  | **Aggregate** |  | **Coverage** |  | **Number Of Coding References** |  | | **Reference Number** | |  | | **Coded By Initials** | |  | | **Modified On** | |  | |
|  | **Codes\\Intervention experience\Influence on others** | | | | | | | | | | | | | | | | | | |  | |
|  |  |  | No |  | 0.0357 |  | 1 |  | | | | | | | | | | | |  |  |
|  |  |  |  |  |  |  |  |  | | | | | | | | | | | | | |
|  | | | | | | | | | | 1 |  | | MTK | |  | | 8/5/2021 2:27 PM | |  | |  |
|  | I: Can you help another person in the community to have helpful thoughts?  R: I can manage.  I: Does it mean that the way you received the counseling, helped you to be able to help other people…?  R: Yes.  I: I can encourage you that you should continue having helpful thoughts because problems shall always be there in life but to think about terminating your life is unhelpful thinking right?  R: Yes. | | | | | | | | | | | | | | | | | | |  | |
|  |  |  |  |  |  |  |  |  |  |  |  |  |  |  |  |  |  |  |  |  |  |
|  |  | | | | | | | | | | | | | | | | | | |  | |
|  | **Codes\\Intervention process\Feelings** | | | | | | | | | | | | | | | | | | |  | |
|  |  |  | No |  | 0.1118 |  | 2 |  | | | | | | | | | | | |  |  |
|  |  |  |  |  |  |  |  |  | | | | | | | | | | | | | |
|  | | | | | | | | | | 1 |  | | MTK | |  | | 8/5/2021 1:56 PM | |  | |  |
|  | I: Thank you very much. Now I would like us to discuss about the counseling intervention that you received in this study in which you took part. I would like you to tell me more about your experiences with this Thinking Healthy Program?  R: I have benefitted a lot in this. I am able to do things that I was not able to do before. I am able to eat, chat and feel free with relatives. I am able to eat different food groups.  I: Meaning that you were able to do all these after receiving the counseling intervention?  R: Yes.  I: What was happening before the counseling intervention was received?  R: I was not thinking healthy, my heart was heavy and I would have killed myself.  I: Okay. So because of the counseling you feel that you changed?  R: Yes. | | | | | | | | | | | | | | | | | | |  | |
|  |  |  |  |  |  |  |  |  |  |  |  |  |  |  |  |  |  |  |  |  |  |
|  |  | | | | | | | | | | | | | | | | | | |  | |
|  | | | | | | | | | | 2 |  | | MTK | |  | | 8/5/2021 2:26 PM | |  | |  |
|  | I: Okay. Thank you. We are now moving towards the end of our discussion. How have you perceived the counseling intervention that you were receiving and how do you feel right now?  R: I am feeling good and different from the past. I feel good and I am happy. I am able to eat properly.  I: You earlier told me that you were depressed and that you reached the extent of wishing to terminate your life…  R: I cannot think about that now. I am happy.  I: You really look happy and the child looks healthy…  R: Yes. | | | | | | | | | | | | | | | | | | |  | |
|  |  |  |  |  |  |  |  |  |  |  |  |  |  |  |  |  |  |  |  |  |  |
|  |  | | | | | | | | | | | | | | | | | | |  | |
|  | **Codes\\Participation of others** | | | | | | | | | | | | | | | | | | |  | |
|  |  |  | No |  | 0.0868 |  | 2 |  | | | | | | | | | | | |  |  |
|  |  |  |  |  |  |  |  |  | | | | | | | | | | | | | |
|  | | | | | | | | | | 1 |  | | MTK | |  | | 8/5/2021 2:23 PM | |  | |  |
|  | I: Did you not invite your relatives to take part in the counseling sessions?  R: They were invited once.  I: Was there any relative who was not happy that you were receiving the counseling intervention?  R: No I was not telling them and they were not against the counseling intervention. | | | | | | | | | | | | | | | | | | |  | |
|  |  | | | | | | | | | | | | | | | | | | |  | |
|  | | | | | | | | | | 2 |  | | MTK | |  | | 8/5/2021 2:25 PM | |  | |  |
|  | I: Would you have loved to have family members around during the counseling sessions?  R: I was telling them but they were busy…  I: Part of the intervention was that you should have good relationship with people around you for support and that required them to be present during the counseling sessions…  R: Yes.  I: Don’t you think that by not being present during the sessions, you were denied the opportunity of their support?  R: Although they did not participate in all sessions but they were supporting me.  I: How could they support you if they were not present during the counseling sessions?  R: They knew that they were supposed to provide me with the support and they were providing me. | | | | | | | | | | | | | | | | | | |  | |
|  |  |  |  |  |  |  |  |  |  |  |  |  |  |  |  |  |  |  |  |  |  |
|  |  | | | | | | | | | | | | | | | | | | |  | |
| Formatted Reports\\Coding Summary by File Formatted Report | | | | | | | | | Page 86 of 145 | | | | | | | | | | | |  |
| 8/9/2021 8:08 AM | | | | | | | | | | | | | | | | | | | | |  |
|  | **Classification** |  | **Aggregate** |  | **Coverage** |  | **Number Of Coding References** |  | | **Reference Number** | |  | | **Coded By Initials** | |  | | **Modified On** | |  | |
|  | **Codes\\Participation of others\Partners** | | | | | | | | | | | | | | | | | | |  | |
|  |  |  | No |  | 0.0334 |  | 1 |  | | | | | | | | | | | |  |  |
|  |  |  |  |  |  |  |  |  | | | | | | | | | | | | | |
|  | | | | | | | | | | 1 |  | | MTK | |  | | 8/5/2021 2:25 PM | |  | |  |
|  | I: Okay… You have told me that your family members were not present but you told me that you were married and that the marriage ended. Did your husband not take part in one of the counseling sessions when he came to visit you?  R: He comes to see the children.  I: Did you tell him about the counseling intervention that you were receiving?  R: I told him but he never attended one. | | | | | | | | | | | | | | | | | | |  | |
|  |  | | | | | | | | | | | | | | | | | | |  | |
|  | **Codes\\Participation of others\Support** | | | | | | | | | | | | | | | | | | |  | |
|  |  |  | No |  | 0.0614 |  | 1 |  | | | | | | | | | | | |  |  |
|  |  |  |  |  |  |  |  |  | | | | | | | | | | | | | |
|  | | | | | | | | | | 1 |  | | MTK | |  | | 8/5/2021 2:25 PM | |  | |  |
|  | I: Would you have loved to have your family members around during the counseling sessions?  R: I was telling them but they were busy…  I: Part of the intervention was that you should have good relationship with people around you for support and that required them to be present during the counseling sessions…  R: Yes.  I: Don’t you think that by not being present during the sessions, you were denied the opportunity of their support?  R: Although they did not participate in all sessions but they were supporting me.  I: How could they support you if they were not present during the counseling sessions?  R: They knew that they were supposed to provide me with the support and they were providing me. | | | | | | | | | | | | | | | | | | |  | |
|  |  |  |  |  |  |  |  |  |  |  |  |  |  |  |  |  |  |  |  |  |  |
|  |  | | | | | | | | | | | | | | | | | | |  | |
|  | **Codes\\Personal history** | | | | | | | | | | | | | | | | | | |  | |
|  |  |  | No |  | 0.1866 |  | 2 |  | | | | | | | | | | | |  |  |
|  |  |  |  |  |  |  |  |  | | | | | | | | | | | | | |
|  | | | | | | | | | | 1 |  | | MTK | |  | | 8/5/2021 1:54 PM | |  | |  |
|  | I: Okay… So firstly, I would like you to tell me the month, year and date when you were born if you can remember…  R: I cannot remember because I was left while very young.  I: Do you know how old you are?  R: 20.  I: How far did you go with your education?  R: 4.  I: Standard 4 or form 4?  R: Standard 4.  I: Why did you not continue with your education to standard 8 or secondary level?  R: I was not intelligent and I had no time to go to school because I was busy cooking for my father because my mother died when I was young.  I: Meaning that you were failing to go to school because you had to cook for your father?  R: Yes. Did your father allow that you should not be going to school so that you should be cooking for him?  I: No he was allowing us to go to school but I just made a decision that I should be farming.  R: Are you currently married?  I: No, I am single.  R: Have you ever been married?  I: Yes.  R: I was married in a polygamous marriage and my husband just told me to leave. Just like they do these days  I: Did you know that you husband had another wife?  R: I did not know. When he was marrying me, he told me that he does not have a wife.  I: For how long did you stay there?  R: I stayed there for four years.  I: So as I said earlier, we knew you when you came for antenatal clinic visit… how many children do you have?  R: Two.  I: Are both of these the children your husband with whom your marriage is terminated?  R: Yes. | | | | | | | | | | | | | | | | | | |  | |
|  |  |  |  |  |  |  |  |  |  |  |  |  |  |  |  |  |  |  |  |  |  |
|  |  |  |  |  |  |  |  |  |  |  |  |  |  |  |  |  |  |  |  |  |  |
|  |  |  |  |  |  |  |  |  |  |  |  |  |  |  |  |  |  |  |  |  |  |
|  |  | | | | | | | | | | | | | | | | | | |  | |
| Formatted Reports\\Coding Summary by File Formatted Report | | | | | | | | | Page 87 of 145 | | | | | | | | | | | |  |
| 8/9/2021 8:08 AM | | | | | | | | | | | | | | | | | | | | |  |
|  | **Classification** |  | **Aggregate** |  | **Coverage** |  | **Number Of Coding References** |  | | **Reference Number** | |  | | **Coded By Initials** | |  | | **Modified On** | |  | |
|  |  |  |  |  |  |  |  |  |  |  |  |  |  |  |  |  |  |  |  |  |  |
|  | | | | | | | | | | 2 |  | | MTK | |  | | 8/5/2021 1:55 PM | |  | |  |
|  | I: You have said that you have two children.. are these the only pregnancies you have had or you had some abortions?  R: They are the only two.  I: Meaning that you only had these two pregnancies?  R: Yes.  I: Which village do you live and which TA?  R: I stay in …….village in TA Chitukula.  I: Chitukula is a TA but not for this side… Are you not from TA Kabudula?  R: Yes Ta Kabudula… [Laughs]…  I: Thank you very much. Do you have a phone?  R: No I don’t have.  I: Is there any relative who has a phone?  R: Yes.  I: Do you know the number?  R: No. | | | | | | | | | | | | | | | | | | |  | |
|  |  |  |  |  |  |  |  |  |  |  |  |  |  |  |  |  |  |  |  |  |  |
|  |  |  |  |  |  |  |  |  |  |  |  |  |  |  |  |  |  |  |  |  |  |
|  |  | | | | | | | | | | | | | | | | | | |  | |
|  | **Codes\\Recommendations\Intervention providers** | | | | | | | | | | | | | | | | | | |  | |
|  |  |  | No |  | 0.0141 |  | 1 |  | | | | | | | | | | | |  |  |
|  |  |  |  |  |  |  |  |  | | | | | | | | | | | | | |
|  | | | | | | | | | | 1 |  | | MTK | |  | | 8/5/2021 2:21 PM | |  | |  |
|  | I: What else did you like about her?  R: She helped me and I was very thankful.  I: Okay.  R: She has to do the same to others so that they should feel better. | | | | | | | | | | | | | | | | | | |  | |
|  |  | | | | | | | | | | | | | | | | | | |  | |
|  | **Codes\\THPP providers** | | | | | | | | | | | | | | | | | | |  | |
|  |  |  | No |  | 0.0324 |  | 1 |  | | | | | | | | | | | |  |  |
|  |  |  |  |  |  |  |  |  | | | | | | | | | | | | | |
|  | | | | | | | | | | 1 |  | | MTK | |  | | 8/5/2021 2:03 PM | |  | |  |
|  | I: Fine. Moving forward… let us talk about the counselor who was coming to your home to provide the counseling… What did you like about her as a person?  R: I liked the fact that she helped me.  I: So, the help aside, what did you like about her as a person?  R: She was giving me soap.  I: What else did you like about her?  R: She helped me and I was very thankful. | | | | | | | | | | | | | | | | | | |  | |
|  |  |  |  |  |  |  |  |  |  |  |  |  |  |  |  |  |  |  |  |  |  |
|  |  | | | | | | | | | | | | | | | | | | |  | |
|  | **Codes\\THPP providers\Characteristics** | | | | | | | | | | | | | | | | | | |  | |
|  |  |  | No |  | 0.0185 |  | 1 |  | | | | | | | | | | | |  |  |
|  |  |  |  |  |  |  |  |  | | | | | | | | | | | | | |
|  | | | | | | | | | | 1 |  | | MTK | |  | | 8/5/2021 2:21 PM | |  | |  |
|  | I: Okay. Can you tell me about the behavior of the counselor apart from that she helped you?  R: She has a good behavior.  I: What made you think she has good behavior?  R: Because of what she was teaching me. | | | | | | | | | | | | | | | | | | |  | |
|  |  | | | | | | | | | | | | | | | | | | |  | |
|  |  |  |  |  |  |  |  |  |  |  |  |  |  |  |  |  |  |  |  |  |  |
| Formatted Reports\\Coding Summary by File Formatted Report | | | | | | | | | Page 88 of 145 | | | | | | | | | | | |  |
| 8/9/2021 8:08 AM | | | | | | | | | | | | | | | | | | | | |  |
|  | **Classification** |  | **Aggregate** |  | **Coverage** |  | **Number Of Coding References** |  | | **Reference Number** | |  | | **Coded By Initials** | |  | | **Modified On** | |  | |
|  | **Codes\\THPP providers\Relationship** | | | | | | | | | | | | | | | | | | |  | |
|  |  |  | No |  | 0.0786 |  | 1 |  | | | | | | | | | | | |  |  |
|  |  |  |  |  |  |  |  |  | | | | | | | | | | | | | |
|  | | | | | | | | | | 1 |  | | MTK | |  | | 8/5/2021 2:22 PM | |  | |  |
|  | I: What was your relationship with the counselor?  R: We were able to understand each other when we were discussing in the house?  I: What do you mean by understanding each other?  R: We were able to understand what she was saying.  I: How can you describe your relationship with the counselor?  R: I can say that our relationship is good and we are still visiting each other as we were doing before…  I: Meaning that you still visit each other?  R: Yes, I go to her home and she comes to my home.  I: I understand that the counseling sessions were terminated?  R: Yes but sometimes when I have a problem I still go to her.  I: Meaning that she is like your help in times of difficulties?  R: Yes.  I: Can we say that you built a relationship with the counselor?  R: Yes.  I: Can you describe this relationship?  R: The type of relationship is that she helped me to start thinking healthy. | | | | | | | | | | | | | | | | | | |  | |
|  |  |  |  |  |  |  |  |  |  |  |  |  |  |  |  |  |  |  |  |  |  |
|  |  |  |  |  |  |  |  |  |  |  |  |  |  |  |  |  |  |  |  |  |  |
|  |  | | | | | | | | | | | | | | | | | | |  | |
|  | **Files\\IDIs\\IDI #2** | | | | | | | | | | | | | | | | | | |  | |
|  | **Code** | | | | | | | | | | | | | | | | | | |  | |
|  | **Codes\\Intervention experience** | | | | | | | | | | | | | | | | | | |  | |
|  |  |  | No |  | 0.0861 |  | 1 |  | | | | | | | | | | | |  |  |
|  |  |  |  |  |  |  |  |  | | | | | | | | | | | | | |
|  | | | | | | | | | | 1 |  | | MTK | |  | | 8/3/2021 9:26 PM | |  | |  |
|  | I: As someone who was experiencing problems, did you see this way of counseling; showing you pictures to be helpful?  P: I cannot say that it was helpful because I was in pain and everything was bad to me.  I: Did you get any lesson from the pictures?  P: I was able to learn a lesson because the way the pictures were described I was able to say “this is a better way to live, I should follow this way and not this one.”  I: Were some of the things that were described in the pictures related to what you were going through?  P: Yes there were some that were related to what I was going through.  I: Can you remember some of the things?  P: Because each day has its problems because it happens that sometimes you are not bothered with thoughts/sometimes you are not thinking too much but sometimes you think too much and that influence you to have unhelpful behavior because a day cannot end without problems. That’s how life is.  I: Now I want to hear from you; you have said that she was showing you pictures where you were learning about helpful and unhelpful behavior. What other things were covered in the sessions, or the counseling you received?  P: Some of the counseling that I received was about taking care of myself, taking care of the child and taking care of the home because to live a healthy life, we need to be in a healthy home.  I: So, taking care of yourself, the child and the home… what else did you discuss?  P: I cannot remember because we have taken long since we discussed because during the previous visit, we did not use pictures. She was just asking questions [EPDS screening] and I was answering. It is difficult because the other person is away. It is different when you are together as we are here… | | | | | | | | | | | | | | | | | | |  | |
|  |  |  |  |  |  |  |  |  |  |  |  |  |  |  |  |  |  |  |  |  |  |
|  |  |  |  |  |  |  |  |  |  |  |  |  |  |  |  |  |  |  |  |  |  |
|  |  | | | | | | | | | | | | | | | | | | |  | |
|  |  |  |  |  |  |  |  |  |  |  |  |  |  |  |  |  |  |  |  |  |  |
| Formatted Reports\\Coding Summary by File Formatted Report | | | | | | | | | Page 89 of 145 | | | | | | | | | | | |  |
| 8/9/2021 8:08 AM | | | | | | | | | | | | | | | | | | | | |  |
|  | **Classification** |  | **Aggregate** |  | **Coverage** |  | **Number Of Coding References** |  | | **Reference Number** | |  | | **Coded By Initials** | |  | | **Modified On** | |  | |
|  | **Codes\\Intervention experience\Aspects** | | | | | | | | | | | | | | | | | | |  | |
|  |  |  | No |  | 0.0567 |  | 1 |  | | | | | | | | | | | |  |  |
|  |  |  |  |  |  |  |  |  | | | | | | | | | | | | | |
|  | | | | | | | | | | 1 |  | | MTK | |  | | 8/3/2021 9:27 PM | |  | |  |
|  | I: Did you get any lesson from the pictures?  P: I was able to learn a lesson because the way the pictures were described I was able to say “this is a better way to live, I should follow this way and not this one.”  I: Were some of the things that were described in the pictures related to what you were going through?  P: Yes there were some that were related to what I was going through.  I: Can you remember some of the things?  P: Because each day has its problems because it happens that sometimes you are not bothered with thoughts/sometimes you are not thinking too much but sometimes you think too much and that influence you to have unhelpful behavior because a day cannot end without problems. That’s how life is.  I: Now I want to hear from you; you have said that she was showing you pictures where you were learning about helpful and unhelpful behavior. What other things were covered in the sessions, or the counseling you received?  P: Some of the counseling that I received was about taking care of myself, taking care of the child and taking care of the home because to live a healthy life, we need to be in a healthy home. | | | | | | | | | | | | | | | | | | |  | |
|  |  |  |  |  |  |  |  |  |  |  |  |  |  |  |  |  |  |  |  |  |  |
|  |  | | | | | | | | | | | | | | | | | | |  | |
|  | **Codes\\Intervention experience\Influence on others** | | | | | | | | | | | | | | | | | | |  | |
|  |  |  | No |  | 0.0765 |  | 1 |  | | | | | | | | | | | |  |  |
|  |  |  |  |  |  |  |  |  | | | | | | | | | | | | | |
|  | | | | | | | | | | 1 |  | | MTK | |  | | 8/4/2021 5:03 AM | |  | |  |
|  | I: If this counseling was to continue, would such misconceptions affect you and what can you do?  P: On my part, maybe they can still be talking but I myself know the truth because I have never even seen the ARVs. However, if they continue to talk, I can stop for fear of tarnishing my image and if I decide to continue maybe my partner may say that I should stop to curb the misconception. Like when I was coming here he asked “are you going for the counseling session?” I said “yes” and she said “make sure you do it fast because we have to go to the garden”.  I: Your partner?  P: Yes and I told him that “I am not going to delay and if I don’t meet them I will come back.”  I: Can you encourage other pregnant women to join this program?  P: [NO RESPONSE].  I: Can you encourage a pregnant woman to join the program?  P: For me, I am able to talk as we are talking here but I cannot be able to read.  I: I mean another pregnant woman… Suppose the program is continuing for pregnant women. Can you advise a pregnant woman to join the program?  P: No because all the pregnant women in my community have delivered.  I: Not that they are available, but we are just imagining if they can be available, can you encourage her to join the program?  P: No.  I: You cannot encourage them?  P: No.  P: Because if something happens to them, they can be pointing fingers at me as if I forced them to join but they should meet with you by themselves at the clinic like I did so that you can explain to them and make their informed choices. | | | | | | | | | | | | | | | | | | |  | |
|  |  |  |  |  |  |  |  |  |  |  |  |  |  |  |  |  |  |  |  |  |  |
|  |  |  |  |  |  |  |  |  |  |  |  |  |  |  |  |  |  |  |  |  |  |
|  |  |  |  |  |  |  |  |  |  |  |  |  |  |  |  |  |  |  |  |  |  |
|  |  | | | | | | | | | | | | | | | | | | |  | |
|  |  |  |  |  |  |  |  |  |  |  |  |  |  |  |  |  |  |  |  |  |  |
|  |  |  |  |  |  |  |  |  |  |  |  |  |  |  |  |  |  |  |  |  |  |
| Formatted Reports\\Coding Summary by File Formatted Report | | | | | | | | | Page 90 of 145 | | | | | | | | | | | |  |
| 8/9/2021 8:08 AM | | | | | | | | | | | | | | | | | | | | |  |
|  | **Classification** |  | **Aggregate** |  | **Coverage** |  | **Number Of Coding References** |  | | **Reference Number** | |  | | **Coded By Initials** | |  | | **Modified On** | |  | |
|  | **Codes\\Intervention experience\Likes** | | | | | | | | | | | | | | | | | | |  | |
|  |  |  | No |  | 0.0567 |  | 1 |  | | | | | | | | | | | |  |  |
|  |  |  |  |  |  |  |  |  | | | | | | | | | | | | | |
|  | | | | | | | | | | 1 |  | | MTK | |  | | 8/3/2021 9:27 PM | |  | |  |
|  | I: Did you get any lesson from the pictures?  P: I was able to learn a lesson because the way the pictures were described I was able to say “this is a better way to live, I should follow this way and not this one.”  I: Were some of the things that were described in the pictures related to what you were going through?  P: Yes there were some that were related to what I was going through.  I: Can you remember some of the things?  P: Because each day has its problems because it happens that sometimes you are not bothered with thoughts/sometimes you are not thinking too much but sometimes you think too much and that influence you to have unhelpful behavior because a day cannot end without problems. That’s how life is.  I: Now I want to hear from you; you have said that she was showing you pictures where you were learning about helpful and unhelpful behavior. What other things were covered in the sessions, or the counseling you received?  P: Some of the counseling that I received was about taking care of myself, taking care of the child and taking care of the home because to live a healthy life, we need to be in a healthy home. | | | | | | | | | | | | | | | | | | |  | |
|  |  |  |  |  |  |  |  |  |  |  |  |  |  |  |  |  |  |  |  |  |  |
|  |  | | | | | | | | | | | | | | | | | | |  | |
|  | **Codes\\Intervention experience\Misconceptions** | | | | | | | | | | | | | | | | | | |  | |
|  |  |  | No |  | 0.1031 |  | 1 |  | | | | | | | | | | | |  |  |
|  |  |  |  |  |  |  |  |  | | | | | | | | | | | | | |
|  | | | | | | | | | | 1 |  | | MTK | |  | | 8/4/2021 5:03 AM | |  | |  |
|  | I: Okay fine. How did the other people from the community react when they knew that you are receiving the intervention, being visited by a counselor?  P: The relatives are the ones I have said that they took part because like the daughter of the landlord, she accepted it when she participated during the session and her partner also accepted. My partner also accepted but then there was a certain misconception in the community last month whereby people were saying that “we hear that you are given ARVs by the one who visits you”.  I: Oooh?  P: Yes, the same person who was participating said “I have heard from the other side of the village that you are given ARVs by the person who comes here and I told them that that is not the case because I even participated during some of the sessions and no ARVs are given. If they are given the pills maybe at the clinic when they first met but when I was with them, no pills were given.” So since that rumor, she has never taken part during the discussion but the other two visits she was present.  I: How did the rumor that you are receiving ARVs affect you?  P: About the ARVs?  I: The misconception that you are receiving ARVs?.  P: When I told my partner, he said “just leave it like that. There is something those people heard from someone who took part probably herself when she goes there to chat because there is no way someone who doesn’t know what happens spread such a rumor.” Because the place where we are staying is isolated from the entire village.  I: If this counseling was to continue, would such misconceptions affect you and what can you do?  P: On my part, maybe they can still be talking but I myself know the truth because I have never even seen the ARVs. However, if they continue to talk, I can stop for fear of tarnishing my image and if I decide to continue maybe my partner may say that I should stop to curb the misconception. Like when I was coming here he asked “are you going for the counseling session?” I said “yes” and she said “make sure you do it fast because we have to go to the garden”. | | | | | | | | | | | | | | | | | | |  | |
|  |  |  |  |  |  |  |  |  |  |  |  |  |  |  |  |  |  |  |  |  |  |
|  |  |  |  |  |  |  |  |  |  |  |  |  |  |  |  |  |  |  |  |  |  |
|  |  |  |  |  |  |  |  |  |  |  |  |  |  |  |  |  |  |  |  |  |  |
|  |  | | | | | | | | | | | | | | | | | | |  | |
|  |  |  |  |  |  |  |  |  |  |  |  |  |  |  |  |  |  |  |  |  |  |
|  |  |  |  |  |  |  |  |  |  |  |  |  |  |  |  |  |  |  |  |  |  |
| Formatted Reports\\Coding Summary by File Formatted Report | | | | | | | | | Page 91 of 145 | | | | | | | | | | | |  |
| 8/9/2021 8:08 AM | | | | | | | | | | | | | | | | | | | | |  |
|  | **Classification** |  | **Aggregate** |  | **Coverage** |  | **Number Of Coding References** |  | | **Reference Number** | |  | | **Coded By Initials** | |  | | **Modified On** | |  | |
|  | **Codes\\Intervention experience\Social harms** | | | | | | | | | | | | | | | | | | |  | |
|  |  |  | No |  | 0.1220 |  | 1 |  | | | | | | | | | | | |  |  |
|  |  |  |  |  |  |  |  |  | | | | | | | | | | | | | |
|  | | | | | | | | | | 1 |  | | MTK | |  | | 8/4/2021 5:08 AM | |  | |  |
|  | I: These are the questions I had but I would like to give you an opportunity to tell me whatever is in your mind about this program.  P: The only concern is the one I have talked about. In terms of the conflict at home, it also happened because of the same issue.  I: When you say the same issue, which one are you referring to?  P: The issue that I am being given ARVs by the counselor.  I: So you had a conflict because of this issue?  P: yes, and also the issue of continuous menses after delivery. So it happened that my partner was consistently discussing my issue with our landlord. She was telling him that he should quiz me I might have a sexually transmitted infection. So whenever she met me, she was asking me about it to the extent of saying that “we need to go somewhere [TRADITIONAL HEALERS] so that they can treat the problem. It can stop.” We went there but when I took the medicine, the menses increased and I stopped after taking for only two days. “I don’t see any change and if you are taking treatment and you do not experience any change you stop because you know that it cannot help you. If it can be helpful, I can continue.” So the two issues were related.  I: Did you tell the counselor about the conflict that you were having with your partner on the two issues that you have outlined?  P: Yes I told her everything.  I: How did she help you?  P: I did not see any help.  I: You have said that your partner participated in two sessions…?  P: Yes he participated in two session. During the first session, he participated and the second session, he participate but during the third session, he found that my partner was not there. I also had just come from the garden and I was cooking when she arrived. I had to stop cooking to attend to her first. On that day, she did not take time because there was a sign that it would rain and she was rushing home because when she was coming here she had just come from her garden.  I: During the visits when your partner was not participating, was he elsewhere or he was right there?  P: He was not around.  I: Okay. You said that she found you cooking on this visit… Were you not agreeing on the time to meet?  P: We were really agreeing on the time and he had the conscious that the counselor was coming but you know men they want to do things their way and you find that when we are expecting the counselor, he goes elsewhere. So it was difficult to meet her. | | | | | | | | | | | | | | | | | | |  | |
|  |  |  |  |  |  |  |  |  |  |  |  |  |  |  |  |  |  |  |  |  |  |
|  |  |  |  |  |  |  |  |  |  |  |  |  |  |  |  |  |  |  |  |  |  |
|  |  |  |  |  |  |  |  |  |  |  |  |  |  |  |  |  |  |  |  |  |  |
|  |  | | | | | | | | | | | | | | | | | | |  | |
|  |  |  |  |  |  |  |  |  |  |  |  |  |  |  |  |  |  |  |  |  |  |
|  |  |  |  |  |  |  |  |  |  |  |  |  |  |  |  |  |  |  |  |  |  |
|  |  |  |  |  |  |  |  |  |  |  |  |  |  |  |  |  |  |  |  |  |  |
|  |  |  |  |  |  |  |  |  |  |  |  |  |  |  |  |  |  |  |  |  |  |
|  |  |  |  |  |  |  |  |  |  |  |  |  |  |  |  |  |  |  |  |  |  |
| Formatted Reports\\Coding Summary by File Formatted Report | | | | | | | | | Page 92 of 145 | | | | | | | | | | | |  |
| 8/9/2021 8:08 AM | | | | | | | | | | | | | | | | | | | | |  |
|  | **Classification** |  | **Aggregate** |  | **Coverage** |  | **Number Of Coding References** |  | | **Reference Number** | |  | | **Coded By Initials** | |  | | **Modified On** | |  | |
|  | **Codes\\Intervention process** | | | | | | | | | | | | | | | | | | |  | |
|  |  |  | No |  | 0.1048 |  | 1 |  | | | | | | | | | | | |  |  |
|  |  |  |  |  |  |  |  |  | | | | | | | | | | | | | |
|  | | | | | | | | | | 1 |  | | MTK | |  | | 8/3/2021 9:26 PM | |  | |  |
|  | I: Do you remember how many visits she conducted?  P: Yes.  I: How many were they?  P: The one she came the day before yesterday is the tenth one.  I: When she came to tell you that you should come here?  P: Yes it was the tenth one.  I: You have said that when she was showing you pictures, she was saying that…  P: “This is not helpful behavior, this is helpful behavior”.  I: As someone who was experiencing problems, did you see this way of counseling; showing you pictures to be helpful?  P: I cannot say that it was helpful because I was in pain and everything was bad to me.  I: Did you get any lesson from the pictures?  P: I was able to learn a lesson because the way the pictures were described I was able to say “this is a better way to live, I should follow this way and not this one.”  I: Were some of the things that were described in the pictures related to what you were going through?  P: Yes there were some that were related to what I was going through.  I: Can you remember some of the things?  P: Because each day has its problems because it happens that sometimes you are not bothered with thoughts/sometimes you are not thinking too much but sometimes you think too much and that influence you to have unhelpful behavior because a day cannot end without problems. That’s how life is.  I: Now I want to hear from you; you have said that she was showing you pictures where you were learning about helpful and unhelpful behavior. What other things were covered in the sessions, or the counseling you received?  P: Some of the counseling that I received was about taking care of myself, taking care of the child and taking care of the home because to live a healthy life, we need to be in a healthy home.  I: So, taking care of yourself, the child and the home… what else did you discuss?  P: I cannot remember because we have taken long since we discussed because during the previous visit, we did not use pictures. She was just asking questions [EPDS screening] and I was answering. It is difficult because the other person is away. It is different when you are together as we are here… | | | | | | | | | | | | | | | | | | |  | |
|  |  |  |  |  |  |  |  |  |  |  |  |  |  |  |  |  |  |  |  |  |  |
|  |  |  |  |  |  |  |  |  |  |  |  |  |  |  |  |  |  |  |  |  |  |
|  |  |  |  |  |  |  |  |  |  |  |  |  |  |  |  |  |  |  |  |  |  |
|  |  | | | | | | | | | | | | | | | | | | |  | |
|  | **Codes\\Intervention process\Delivery** | | | | | | | | | | | | | | | | | | |  | |
|  |  |  | No |  | 0.1511 |  | 2 |  | | | | | | | | | | | |  |  |
|  |  |  |  |  |  |  |  |  | | | | | | | | | | | | | |
|  | | | | | | | | | | 1 |  | | MTK | |  | | 8/3/2021 9:21 PM | |  | |  |
|  | I: Did you not receive any counseling from the counselor regarding the health issues you were going through when they were coming to your home?  P: She was giving me counseling through the pictures like she would show me pictures and say “this is not helpful behavior and this is helpful behavior. So I will come again on this date on Thursday.” When she did not come on Thursday, she could come probably on Sunday. Sometimes she could not come for two weeks and you could see her coming the next week. She really worked very hard. | | | | | | | | | | | | | | | | | | |  | |
|  |  | | | | | | | | | | | | | | | | | | |  | |
|  |  |  |  |  |  |  |  |  |  |  |  |  |  |  |  |  |  |  |  |  |  |
|  |  |  |  |  |  |  |  |  |  |  |  |  |  |  |  |  |  |  |  |  |  |
|  |  |  |  |  |  |  |  |  |  |  |  |  |  |  |  |  |  |  |  |  |  |
| Formatted Reports\\Coding Summary by File Formatted Report | | | | | | | | | Page 93 of 145 | | | | | | | | | | | |  |
| 8/9/2021 8:08 AM | | | | | | | | | | | | | | | | | | | | |  |
|  | **Classification** |  | **Aggregate** |  | **Coverage** |  | **Number Of Coding References** |  | | **Reference Number** | |  | | **Coded By Initials** | |  | | **Modified On** | |  | |
|  |  |  |  |  |  |  |  |  |  |  |  |  |  |  |  |  |  |  |  |  |  |
|  | | | | | | | | | | 2 |  | | MTK | |  | | 8/3/2021 9:32 PM | |  | |  |
|  | I: So, taking care of yourself, the child and the home… what else did you discuss?  P: I cannot remember because we have taken long since we discussed because during the previous visit, we did not use pictures. She was just asking questions [EPDS screening] and I was answering. It is difficult because the other person is away. It is different when you are together as we are here…  I: So what part of counseling did you perceive to be more helpful and the one that you liked?  P: So because I did not take it seriously when you told us that you will be visiting us because it has never happened before.  I: So when you saw her coming…?  P: Oh, that’s when I believed that these people are committed, the volunteer was really coming. So I did not take it seriously when you told us but when I saw her coming, that was when I took it to be serious.  I: Mh. So if a fellow pregnant woman can ask you; “what can I benefit from the home based counseling that you were receiving?” What can you tell her?  P: A pregnant woman?  I: Yes, if she can ask you? Imagine I am a pregnant woman in your community and I want you to tell me what benefits I can get from the counseling that you were receiving?  P: I don’t know.  I: Okay. Perhaps what is it that you did not like about this counseling?  P: So, because the way it is, the counselor is there using the books , showing us pictures and asking us questions. Our only role was to answer the one asking us. That is why it is difficult for me because she does that during subsequent visits so I just say “maybe this is the way to go.”  I: So I am giving you the authority to tell us how we can change the intervention in order to improve it. What are your opinions in terms of how we can provide quality service to the woman?  P: It depends on you because that is how you deliver the service.  I: It is also good to hear views of participants, the person receiving the service to comment so that we can improve the service provision besides what we are doing now by showing you pictures, asking questions… as you said… Is there anything you think could have been done so that the woman can be satisfied by the service that we provide her?  P: Because when she was visiting she said that “we will tell you when we will terminate the sessions” but when she came the day before yesterday, she said “this marks the end of our sessions and during the interview.. So I am going to get all the answers from you. | | | | | | | | | | | | | | | | | | |  | |
|  |  |  |  |  |  |  |  |  |  |  |  |  |  |  |  |  |  |  |  |  |  |
|  |  |  |  |  |  |  |  |  |  |  |  |  |  |  |  |  |  |  |  |  |  |
|  |  |  |  |  |  |  |  |  |  |  |  |  |  |  |  |  |  |  |  |  |  |
|  |  |  |  |  |  |  |  |  |  |  |  |  |  |  |  |  |  |  |  |  |  |
|  |  | | | | | | | | | | | | | | | | | | |  | |
|  |  |  |  |  |  |  |  |  |  |  |  |  |  |  |  |  |  |  |  |  |  |
|  |  |  |  |  |  |  |  |  |  |  |  |  |  |  |  |  |  |  |  |  |  |
|  |  |  |  |  |  |  |  |  |  |  |  |  |  |  |  |  |  |  |  |  |  |
|  |  |  |  |  |  |  |  |  |  |  |  |  |  |  |  |  |  |  |  |  |  |
|  |  |  |  |  |  |  |  |  |  |  |  |  |  |  |  |  |  |  |  |  |  |
| Formatted Reports\\Coding Summary by File Formatted Report | | | | | | | | | Page 94 of 145 | | | | | | | | | | | |  |
| 8/9/2021 8:08 AM | | | | | | | | | | | | | | | | | | | | |  |
|  | **Classification** |  | **Aggregate** |  | **Coverage** |  | **Number Of Coding References** |  | | **Reference Number** | |  | | **Coded By Initials** | |  | | **Modified On** | |  | |
|  | **Codes\\Intervention process\Home situation** | | | | | | | | | | | | | | | | | | |  | |
|  |  |  | No |  | 0.1727 |  | 1 |  | | | | | | | | | | | |  |  |
|  |  |  |  |  |  |  |  |  | | | | | | | | | | | | | |
|  | | | | | | | | | | 1 |  | | MTK | |  | | 8/4/2021 5:10 AM | |  | |  |
|  | I: These are the questions I had but I would like to give you an opportunity to tell me whatever is in your mind about this program.  P: The only concern is the one I have talked about. In terms of the conflict at home, it also happened because of the same issue.  I: When you say the same issue, which one are you referring to?  P: The issue that I am being given ARVs by the counselor.  I: So you had a conflict because of this issue?  P: yes, and also the issue of continuous menses after delivery. So it happened that my partner was consistently discussing my issue with the daughter in law of our landlord. She was telling him that he should quiz me I might have a sexually transmitted infection. So whenever she met me, she was asking me about it to the extent of saying that “we need to go somewhere [TRADITIONAL HEALERS] so that they can treat the problem. It can stop.” We went there but when I took the medicine, the menses increased and I stopped after taking for only two days. “I don’t see any change and if you are taking treatment and you do not experience any change you stop because you know that it cannot help you. If it can be helpful, I can continue.” So the two issues were related.  I: Did you tell the counselor about the conflict that you were having with your partner on the two issues that you have outlined?  P: Yes I told her everything.  I: How did she help you?  P: I did not see any help.  I: You have said that your partner participated in two sessions…?  P: Yes he participated in two session. During the first session, he participated and the second session, he participate but during the third session, he found that my partner was not there. I also had just come from the garden and I was cooking when she arrived. I had to stop cooking to attend to her first. On that day, she did not take time because there was a sign that it would rain and she was rushing home because when she was coming here she had just come from her garden.  I: During the visits when your partner was not participating, was he elsewhere or he was right there?  P: He was not around.  I: Okay. You said that she found you cooking on this visit… Were you not agreeing on the time to meet?  P: We were really agreeing on the time and he had the conscious that the counselor was coming but you know men they want to do things their way and you find that when we are expecting the counselor, he goes elsewhere. So it was difficult to meet her.  I: So lastly, I would like to hear from you; when we met you the first time, you seemed depressed. so now after the counseling sessions, how are you feeling, is there any change, what has changed?  P: I feel it is the same as I was before. The only difference is that I had never heard that there is such type of counseling so the fact that I have been exposed to this counseling, that is the change I can say I have experienced.  I: Has it helped you in any way?  P: If it is the help, it is about my feelings as she was telling me.  I: How are you feeling now?  P: It is the same.  I: What do you mean when you say it is the same?  P: It is the same because problems cannot end at once and not all days are Sundays.  I: Okay, thanks you so much Mrs. Wilson, this marks the end of our discussion. If we set date for the group discussion, we will communicate to you. Do you have a phone?  P: No I don’t have.  I: It means the counselor will communicate to you.  P: Okay.  I: Thank you very much.  P: You are welcome. | | | | | | | | | | | | | | | | | | |  | |
|  |  |  |  |  |  |  |  |  |  |  |  |  |  |  |  |  |  |  |  |  |  |
|  |  |  |  |  |  |  |  |  |  |  |  |  |  |  |  |  |  |  |  |  |  |
|  |  |  |  |  |  |  |  |  |  |  |  |  |  |  |  |  |  |  |  |  |  |
|  |  |  |  |  |  |  |  |  |  |  |  |  |  |  |  |  |  |  |  |  |  |
|  |  |  |  |  |  |  |  |  |  |  |  |  |  |  |  |  |  |  |  |  |  |
|  |  |  |  |  |  |  |  |  |  |  |  |  |  |  |  |  |  |  |  |  |  |
|  |  | | | | | | | | | | | | | | | | | | |  | |
|  |  |  |  |  |  |  |  |  |  |  |  |  |  |  |  |  |  |  |  |  |  |
|  |  |  |  |  |  |  |  |  |  |  |  |  |  |  |  |  |  |  |  |  |  |
| Formatted Reports\\Coding Summary by File Formatted Report | | | | | | | | | Page 95 of 145 | | | | | | | | | | | |  |
| 8/9/2021 8:08 AM | | | | | | | | | | | | | | | | | | | | |  |
|  | **Classification** |  | **Aggregate** |  | **Coverage** |  | **Number Of Coding References** |  | | **Reference Number** | |  | | **Coded By Initials** | |  | | **Modified On** | |  | |
|  | **Codes\\Other interesting issues** | | | | | | | | | | | | | | | | | | |  | |
|  |  |  | No |  | 0.1219 |  | 1 |  | | | | | | | | | | | |  |  |
|  |  |  |  |  |  |  |  |  | | | | | | | | | | | | | |
|  | | | | | | | | | | 1 |  | | MTK | |  | | 8/4/2021 9:02 AM | |  | |  |
|  | I: These are the questions I had but I would like to give you an opportunity to tell me whatever is in your mind about this program.  P: The only concern is the one I have talked about. In terms of the conflict at home, it also happened because of the same issue.  I: When you say the same issue, which one are you referring to?  P: The issue that I am being given ARVs by the counselor.  I: So you had a conflict because of this issue?  P: yes, and also the issue of continuous menses after delivery. So it happened that my partner was consistently discussing my issue with the daughter in law of our landlord. She was telling him that he should quiz me I might have a sexually transmitted infection. So whenever she met me, she was asking me about it to the extent of saying that “we need to go somewhere [TRADITIONAL HEALERS] so that they can treat the problem. It can stop.” We went there but when I took the medicine, the menses increased and I stopped after taking for only two days. “I don’t see any change and if you are taking treatment and you do not experience any change you stop because you know that it cannot help you. If it can be helpful, I can continue.” So the two issues were related.  I: Did you tell the counselor about the conflict that you were having with your partner on the two issues that you have outlined?  P: Yes I told her everything.  I: How did she help you?  P: I did not see any help.  I: You have said that your partner participated in two sessions…?  P: Yes he participated in two session. During the first session, he participated and the second session, he participate but during the third session, he found that my partner was not there. I also had just come from the garden and I was cooking when she arrived. I had to stop cooking to attend to her first. On that day, she did not take time because there was a sign that it would rain and she was rushing home because when she was coming here she had just come from her garden.  I: During the visits when your partner was not participating, was he elsewhere or he was right there?  P: He was not around.  I: Okay. You said that she found you cooking on this visit… Were you not agreeing on the time to meet?  P: We were really agreeing on the time and he had the conscious that the counselor was coming but you know men they want to do things their way and you find that when we are expecting the counselor, he goes elsewhere. So it was difficult to meet her. | | | | | | | | | | | | | | | | | | |  | |
|  |  |  |  |  |  |  |  |  |  |  |  |  |  |  |  |  |  |  |  |  |  |
|  |  |  |  |  |  |  |  |  |  |  |  |  |  |  |  |  |  |  |  |  |  |
|  |  |  |  |  |  |  |  |  |  |  |  |  |  |  |  |  |  |  |  |  |  |
|  |  | | | | | | | | | | | | | | | | | | |  | |
|  | **Codes\\Participation of others** | | | | | | | | | | | | | | | | | | |  | |
|  |  |  | No |  | 0.0154 |  | 1 |  | | | | | | | | | | | |  |  |
|  |  |  |  |  |  |  |  |  | | | | | | | | | | | | | |
|  | | | | | | | | | | 1 |  | | MTK | |  | | 8/3/2021 9:36 PM | |  | |  |
|  | I: Was there any family member who was involved in the programme?  P: During the visits?  I: Who was present during the counseling sessions…?  P: There was one. She sat in for two days.  I: Who is this one?  P: We found her right there where we work. She is the daughter of the owner of the house [EMPLOYER]. | | | | | | | | | | | | | | | | | | |  | |
|  |  | | | | | | | | | | | | | | | | | | |  | |
|  | **Codes\\Participation of others\Attitude** | | | | | | | | | | | | | | | | | | |  | |
|  |  |  | No |  | 0.0969 |  | 2 |  | | | | | | | | | | | |  |  |
|  |  |  |  |  |  |  |  |  | | | | | | | | | | | | | |
|  | | | | | | | | | | 1 |  | | MTK | |  | | 8/3/2021 9:39 PM | |  | |  |
|  | I: Okay fine. How did the other people from the community react when they knew that you are receiving the intervention, being visited by a counselor?  P: The relatives are the ones I have said that they took part because like the daughter of the landlord, she accepted it when she participated during the session and her partner also accepted. My partner also accepted but then there was a certain misconception in the community last month whereby people were saying that “we hear that you are given ARVs by the one who visits you”.  I: Oooh?  P: Yes, the same person who was participating said “I have heard from the other side of the village that you are given ARVs by the person who comes here and I told them that that is not the case because I even participated during some of the sessions and no ARVs are given. If they are given the pills maybe at the clinic when they first met but when I was with them, no pills were given.” So since that rumor, she has never taken part during the discussion but the other two visits she was present. | | | | | | | | | | | | | | | | | | |  | |
|  |  |  |  |  |  |  |  |  |  |  |  |  |  |  |  |  |  |  |  |  |  |
|  |  | | | | | | | | | | | | | | | | | | |  | |
| Formatted Reports\\Coding Summary by File Formatted Report | | | | | | | | | Page 96 of 145 | | | | | | | | | | | |  |
| 8/9/2021 8:08 AM | | | | | | | | | | | | | | | | | | | | |  |
|  | **Classification** |  | **Aggregate** |  | **Coverage** |  | **Number Of Coding References** |  | | **Reference Number** | |  | | **Coded By Initials** | |  | | **Modified On** | |  | |
|  |  |  |  |  |  |  |  |  |  |  |  |  |  |  |  |  |  |  |  |  |  |
|  | | | | | | | | | | 2 |  | | MTK | |  | | 8/4/2021 4:55 AM | |  | |  |
|  | I: If this counseling was to continue, would such misconceptions affect you and what can you do?  P: On my part, maybe they can still be talking but I myself know the truth because I have never even seen the ARVs. However, if they continue to talk, I can stop for fear of tarnishing my image and if I decide to continue maybe my partner may say that I should stop to curb the misconception. Like when I was coming here he asked “are you going for the counseling session?” I said “yes” and she said “make sure you do it fast because we have to go to the garden”.  I: Your partner?  P: Yes and I told him that “I am not going to delay and if I don’t meet them I will come back.”  I: Can you encourage other pregnant women to join this program?  P: [NO RESPONSE].  I: Can you encourage a pregnant woman to join the program?  P: For me, I am able to talk as we are talking here but I cannot be able to read. | | | | | | | | | | | | | | | | | | |  | |
|  |  |  |  |  |  |  |  |  |  |  |  |  |  |  |  |  |  |  |  |  |  |
|  |  | | | | | | | | | | | | | | | | | | |  | |
|  | **Codes\\Participation of others\Community participation** | | | | | | | | | | | | | | | | | | |  | |
|  |  |  | No |  | 0.1051 |  | 2 |  | | | | | | | | | | | |  |  |
|  |  |  |  |  |  |  |  |  | | | | | | | | | | | | | |
|  | | | | | | | | | | 1 |  | | MTK | |  | | 8/3/2021 9:38 PM | |  | |  |
|  | I: You have said that you are at an estate, at your work place?  P: Yes.  I: Meaning that your mother or relatives are not there except for your partner?  P: Yes.  I: Okay fine. How did the other people from the community react when they knew that you are receiving the intervention, being visited by a counselor?  P: The relatives are the ones I have said that they took part because like the daughter of the landlord, she accepted it when she participated during the session and her partner also accepted. My partner also accepted but then there was a certain misconception in the community last month whereby people were saying that “we hear that you are given ARVs by the one who visits you”.  I: Oooh? | | | | | | | | | | | | | | | | | | |  | |
|  |  |  |  |  |  |  |  |  |  |  |  |  |  |  |  |  |  |  |  |  |  |
|  |  |  |  |  |  |  |  |  |  |  |  |  |  |  |  |  |  |  |  |  |  |
|  |  | | | | | | | | | | | | | | | | | | |  | |
|  | | | | | | | | | | 2 |  | | MTK | |  | | 8/4/2021 4:57 AM | |  | |  |
|  | I: If this counseling was to continue, would such misconceptions affect you and what can you do?  P: On my part, maybe they can still be talking but I myself know the truth because I have never even seen the ARVs. However, if they continue to talk, I can stop for fear of tarnishing my image and if I decide to continue maybe my partner may say that I should stop to curb the misconception. Like when I was coming here he asked “are you going for the counseling session?” I said “yes” and she said “make sure you do it fast because we have to go to the garden”.  I: Your partner?  P: Yes and I told him that “I am not going to delay and if I don’t meet them I will come back.”  I: Can you encourage other pregnant women to join this program?  P: [NO RESPONSE].  I: Can you encourage a pregnant woman to join the program?  P: For me, I am able to talk as we are talking here but I cannot be able to read. | | | | | | | | | | | | | | | | | | |  | |
|  |  |  |  |  |  |  |  |  |  |  |  |  |  |  |  |  |  |  |  |  |  |
|  |  | | | | | | | | | | | | | | | | | | |  | |
|  | **Codes\\Participation of others\Partners** | | | | | | | | | | | | | | | | | | |  | |
|  |  |  | No |  | 0.0070 |  | 1 |  | | | | | | | | | | | |  |  |
|  |  |  |  |  |  |  |  |  | | | | | | | | | | | | | |
|  | | | | | | | | | | 1 |  | | MTK | |  | | 8/3/2021 9:36 PM | |  | |  |
|  | I: Was your partner taking part?  P: Yes he participated for two days but on the third day, the volunteer found that he had gone else where. | | | | | | | | | | | | | | | | | | |  | |
|  |  | | | | | | | | | | | | | | | | | | |  | |
| Formatted Reports\\Coding Summary by File Formatted Report | | | | | | | | | Page 97 of 145 | | | | | | | | | | | |  |
| 8/9/2021 8:08 AM | | | | | | | | | | | | | | | | | | | | |  |
|  | **Classification** |  | **Aggregate** |  | **Coverage** |  | **Number Of Coding References** |  | | **Reference Number** | |  | | **Coded By Initials** | |  | | **Modified On** | |  | |
|  | **Codes\\Participation of others\Support** | | | | | | | | | | | | | | | | | | |  | |
|  |  |  | No |  | 0.0230 |  | 1 |  | | | | | | | | | | | |  |  |
|  |  |  |  |  |  |  |  |  | | | | | | | | | | | | | |
|  | | | | | | | | | | 1 |  | | MTK | |  | | 8/3/2021 9:39 PM | |  | |  |
|  | I: How did the rumor that you are receiving ARVs affect you?  P: About the ARVs?  I: The misconception that you are receiving ARVs?.  P: When I told my partner, he said “just leave it like that. There is something those people heard from someone who took part probably herself when she goes there to chat because there is no way someone who doesn’t know what happens spread such a rumor.” Because the place where we are staying is isolated from the entire village. | | | | | | | | | | | | | | | | | | |  | |
|  |  | | | | | | | | | | | | | | | | | | |  | |
|  | **Codes\\Personal history** | | | | | | | | | | | | | | | | | | |  | |
|  |  |  | No |  | 0.2415 |  | 3 |  | | | | | | | | | | | |  |  |
|  |  |  |  |  |  |  |  |  | | | | | | | | | | | | | |
|  | | | | | | | | | | 1 |  | | MTK | |  | | 8/3/2021 8:58 PM | |  | |  |
|  | I: So I would like to ask you to feel free. As we said at the beginning, when you join a study, much as you give us personal information, we keep it confidential. It is only used for study purposes. So feel free to tell me whatever you feel like telling me. I have all the time for you. Sure. How old is the child now?  P: Two months old.  I: How old are you?  P: I am 23 years old. | | | | | | | | | | | | | | | | | | |  | |
|  |  | | | | | | | | | | | | | | | | | | |  | |
|  | | | | | | | | | | 2 |  | | MTK | |  | | 8/3/2021 9:10 PM | |  | |  |
|  | I: How far did you go with your education?  P: On the part of my education and in terms of the visits, we really had several visits and since we met up, started having the session to the time of delivery, I did not experience any stress/anxiety. I was living a healthy life and my eating was normal. However, after delivery, I had been sick. I had been having continuous menses. Each time I experience back pains, I would know that very soon I am going to have menses. Sometimes I would stay for two days without menses, then once I have the back pain, the menses would come again, and as heavy as during delivery. I started developing edema but now people can see that I am improving.  I: For how long has this been happening?  P: It has happened for more than one month. So it happened that we started having conflict with my partner and we went to a certain hospital and they referred us back to where I delivered so that I could explain to them. So I came and explained and I was treated. However, after the treatment, the menses did not stop until the time when my child was due for vaccine. That was when I decided to have depo contraception. After the contraception injection, I started having abdominal pains. Then it happened that I had heavy menses once after the contraception injection and since then, I have never had menses to date.  I: You were explaining to me about the problem that you had when they gave you treatment here because I wanted to talk to the people who came. So can you continue?  P: They gave me daily contraception pills, bufen and iron tablets. So I started feeling better but then the problem was with heart palpitation that I could not walk a long distance. When I finished that dose, it was when I had a depot injection. After the injection, in the morning, I noted that I had heavy menses and it just happened that time. It never happened in the afternoon and evening, Up to now as you see me, that problem stopped.  I: So did they check your blood levels when you came to the hospital considering that you had heavy menses for a long time?  P: During that time, they did not test but they asked “have you come a lone?” I said “yes.” Then they said your problem is anemia and it needs transfusion and it is unfortunate that you have come alone. You have lost a lot of blood and after taking the pills that we have given you, if you do not experience any change you should come back.”  I: Did you finish taking the pills?  P: I finished taking the pills but I did not experience great change. The only change was that I stopped having heavy menses. The menses were minimized but I was still having the abdominal pains. So that was when I switched to the injection and that was when I had heavy menses once but still have the abdominal pains. Sometimes when I do hard work, I have heart palpitation. It’s a challenge because I am not staying at my home, am at an estate so I still have to work although I had just delivered, and I didn’t have enough energy. The other thing is that we have been having communal meals which is also a challenge. The other thing is that the people talk a lot about me, the owners of the house at the estate but I do not care about what they say so long I can get better.  I: So before we go further, do you still experience heart palpitation for example they way you have walked to the hospital today?  P: To say the truth, I have walked without challenges and I don’t feel heart palpitation often these day. Sometimes I have palpitations sometimes heartache as if I have a fight/conflict with someone, but today am fine.  I: Are you still having menses?  P: No. I have not had it since last of last week.  I: Meaning you have not had it for two weeks?  P: Yes. That is when I have lived as a normal woman but since 23 December I cannot say that things were okay or things were better no. | | | | | | | | | | | | | | | | | | |  | |
|  |  |  |  |  |  |  |  |  |  |  |  |  |  |  |  |  |  |  |  |  |  |
|  |  |  |  |  |  |  |  |  |  |  |  |  |  |  |  |  |  |  |  |  |  |
|  |  |  |  |  |  |  |  |  |  |  |  |  |  |  |  |  |  |  |  |  |  |
|  |  |  |  |  |  |  |  |  |  |  |  |  |  |  |  |  |  |  |  |  |  |
|  |  |  |  |  |  |  |  |  |  |  |  |  |  |  |  |  |  |  |  |  |  |
|  |  | | | | | | | | | | | | | | | | | | |  | |
| Formatted Reports\\Coding Summary by File Formatted Report | | | | | | | | | Page 98 of 145 | | | | | | | | | | | |  |
| 8/9/2021 8:08 AM | | | | | | | | | | | | | | | | | | | | |  |
|  | **Classification** |  | **Aggregate** |  | **Coverage** |  | **Number Of Coding References** |  | | **Reference Number** | |  | | **Coded By Initials** | |  | | **Modified On** | |  | |
|  |  |  |  |  |  |  |  |  |  |  |  |  |  |  |  |  |  |  |  |  |  |
|  | | | | | | | | | | 3 |  | | MTK | |  | | 8/3/2021 9:10 PM | |  | |  |
|  | I: How many children do you have? …this one…?  P: S/he is the second born.  I: Second born… What about number of pregnancies?  P: I had the other one which I had the same experience. When I slept, I just noted that I had abdominal pains and started menses. It was three months pregnancy and it ended with sickness like that and I stayed for one year without working. It was when I became pregnant again for this one.  I: Is the child alive?  P: Yes.  I: How old is the child?  P: About five years now. | | | | | | | | | | | | | | | | | | |  | |
|  |  |  |  |  |  |  |  |  |  |  |  |  |  |  |  |  |  |  |  |  |  |
|  |  |  |  |  |  |  |  |  |  |  |  |  |  |  |  |  |  |  |  |  |  |
|  |  | | | | | | | | | | | | | | | | | | |  | |
|  | **Codes\\Recommendations\Place of delivery** | | | | | | | | | | | | | | | | | | |  | |
|  |  |  | No |  | 0.0356 |  | 1 |  | | | | | | | | | | | |  |  |
|  |  |  |  |  |  |  |  |  | | | | | | | | | | | | | |
|  | | | | | | | | | | 1 |  | | MTK | |  | | 8/3/2021 9:35 PM | |  | |  |
|  | I: What can you say about the place where the intervention was being provided? Because this is the first time we have provided the intervention at home. So do you think it should be provided at home or at the clinic?  P: The best place is at home because we are far from the clinic…  I: You are far…  P: Especially us who are employed, it means if we go to the clinic, we have not worked while our friends have gone to cut tobacco but if they can be coming at our home, it means they would find that I have already done my work because she was coming in the afternoon.  I: So you have said that the best reason why the counseling visits should be home based is that you do not travel they find you at home…  P: Yes. | | | | | | | | | | | | | | | | | | |  | |
|  |  |  |  |  |  |  |  |  |  |  |  |  |  |  |  |  |  |  |  |  |  |
|  |  | | | | | | | | | | | | | | | | | | |  | |
|  | **Codes\\THPP providers\Characteristics** | | | | | | | | | | | | | | | | | | |  | |
|  |  |  | No |  | 0.0397 |  | 2 |  | | | | | | | | | | | |  |  |
|  |  |  |  |  |  |  |  |  | | | | | | | | | | | | | |
|  | | | | | | | | | | 1 |  | | MTK | |  | | 8/3/2021 9:34 PM | |  | |  |
|  | I: Sure and at the beginning, I said that you should feel free. It’s not like you are reporting the counselor but we want to improve how we provide the service to people. The other question that I would like to ask is about the counselor’s behavior. What behaviors or conduct did you like in your counselor?  P: I observed the good behavior because she was really teaching me that “this is helpful behavior and this is unhelpful behavior”.  I: Do you remember some of the behaviors that were said to be helpful or unhelpful?  P: No. | | | | | | | | | | | | | | | | | | |  | |
|  |  | | | | | | | | | | | | | | | | | | |  | |
|  | | | | | | | | | | 2 |  | | MTK | |  | | 8/3/2021 9:34 PM | |  | |  |
|  | I: Was there any behavior of the counselor that you did not like?  P: Like those from home?  I: The one who was visiting you at your home?  P: She was following her program and I did not see anything bad because that is how she is supposed to work. I don’t know this. | | | | | | | | | | | | | | | | | | |  | |
|  |  | | | | | | | | | | | | | | | | | | |  | |
|  |  |  |  |  |  |  |  |  |  |  |  |  |  |  |  |  |  |  |  |  |  |
|  |  |  |  |  |  |  |  |  |  |  |  |  |  |  |  |  |  |  |  |  |  |
| Formatted Reports\\Coding Summary by File Formatted Report | | | | | | | | | Page 99 of 145 | | | | | | | | | | | |  |
| 8/9/2021 8:08 AM | | | | | | | | | | | | | | | | | | | | |  |
|  | **Classification** |  | **Aggregate** |  | **Coverage** |  | **Number Of Coding References** |  | | **Reference Number** | |  | | **Coded By Initials** | |  | | **Modified On** | |  | |
|  | **Files\\IDIs\\IDI #3** | | | | | | | | | | | | | | | | | | |  | |
|  | **Code** | | | | | | | | | | | | | | | | | | |  | |
|  | **Codes\\Intervention experience\Aspects** | | | | | | | | | | | | | | | | | | |  | |
|  |  |  | No |  | 0.1018 |  | 2 |  | | | | | | | | | | | |  |  |
|  |  |  |  |  |  |  |  |  | | | | | | | | | | | | | |
|  | | | | | | | | | | 1 |  | | MTK | |  | | 8/4/2021 5:33 AM | |  | |  |
|  | I: Okay. What part of counseling did you find to be very helpful?  P: It was very helpful because I can see a difference from how I was before these sessions. In the past I was thinking too much, many bad thoughts but when the counselor started visiting me, she helped me deal with those thoughts. I am thanking her for what she did to me. | | | | | | | | | | | | | | | | | | |  | |
|  |  | | | | | | | | | | | | | | | | | | |  | |
|  | | | | | | | | | | 2 |  | | MTK | |  | | 8/4/2021 5:39 AM | |  | |  |
|  | I: How do you perceive it having your partner and your mother during your counseling intervention session, how helpful was it?  P: [NO RESPONSE]  I: Or was it helpful?  P: It was helpful because it influenced people to change how they communicate to you and how much work I was supposed to be doing at home.  I: Can you elaborate what you are say, change in communication and the amount of work?  P: The way our home is, I was working a lot as if I am not pregnant and their participation in counseling helped them to change their attitude towards me and the way they relate to me. They started supporting me  I: When you say you were working as if you were not pregnant, what do you mean?  P: I was working very hard and that was why it was important that they heard it from the host [counselor]mouth because had it been that I told them, they would think that I was lying. | | | | | | | | | | | | | | | | | | |  | |
|  |  |  |  |  |  |  |  |  |  |  |  |  |  |  |  |  |  |  |  |  |  |
|  |  | | | | | | | | | | | | | | | | | | |  | |
|  | **Codes\\Intervention experience\Barriers** | | | | | | | | | | | | | | | | | | |  | |
|  |  |  | No |  | 0.0135 |  | 1 |  | | | | | | | | | | | |  |  |
|  |  |  |  |  |  |  |  |  | | | | | | | | | | | | | |
|  | | | | | | | | | | 1 |  | | MTK | |  | | 8/4/2021 5:33 AM | |  | |  |
|  | I: Maybe I should ask you another question… Can you tell me if there were any barriers that prevented you from receiving the counseling?  P: No nothing. | | | | | | | | | | | | | | | | | | |  | |
|  |  | | | | | | | | | | | | | | | | | | |  | |
|  | **Codes\\Intervention experience\Facilitators** | | | | | | | | | | | | | | | | | | |  | |
|  |  |  | No |  | 0.0123 |  | 1 |  | | | | | | | | | | | |  |  |
|  |  |  |  |  |  |  |  |  | | | | | | | | | | | | | |
|  | | | | | | | | | | 1 |  | | MTK | |  | | 8/4/2021 5:34 AM | |  | |  |
|  | I: Nothing… Were you able to meet on the scheduled days?  P: Yes. If I was busy, I was able to leave whatever I was doing to meet with her. | | | | | | | | | | | | | | | | | | |  | |
|  |  | | | | | | | | | | | | | | | | | | |  | |
|  | **Codes\\Intervention experience\Influence on others** | | | | | | | | | | | | | | | | | | |  | |
|  |  |  | No |  | 0.0353 |  | 1 |  | | | | | | | | | | | |  |  |
|  |  |  |  |  |  |  |  |  | | | | | | | | | | | | | |
|  | | | | | | | | | | 1 |  | | MTK | |  | | 8/4/2021 5:49 AM | |  | |  |
|  | I: Assuming the programme was continuing, Can you encourage other pregnant women to join the program?  P: Yes I can tell her.  I: What can influence you to tell another pregnant woman to join the program?  P: To influence her?  I: Yes so that she can join the study?  P: Mainly it is about how I was feeling before and how things changed for me and how I am feeling now, she can be influenced to join. | | | | | | | | | | | | | | | | | | |  | |
|  |  | | | | | | | | | | | | | | | | | | |  | |
| Formatted Reports\\Coding Summary by File Formatted Report | | | | | | | | | Page 100 of 145 | | | | | | | | | | | |  |
| 8/9/2021 8:08 AM | | | | | | | | | | | | | | | | | | | | |  |
|  | **Classification** |  | **Aggregate** |  | **Coverage** |  | **Number Of Coding References** |  | | **Reference Number** | |  | | **Coded By Initials** | |  | | **Modified On** | |  | |
|  | **Codes\\Intervention experience\Likes** | | | | | | | | | | | | | | | | | | |  | |
|  |  |  | No |  | 0.0358 |  | 1 |  | | | | | | | | | | | |  |  |
|  |  |  |  |  |  |  |  |  | | | | | | | | | | | | | |
|  | | | | | | | | | | 1 |  | | MTK | |  | | 8/4/2021 5:51 AM | |  | |  |
|  | I: How did you accept this intervention?  P: Yes I accepted it. I accepted the intervention because it has changed and healed me.  I: Do you think it is a kind of service that can be acceptable to pregnant women in their homes?  P: Yes because a pregnant woman has a lot of problems and have many thoughts and if she can receive this intervention she can be helped, she can stop thinking about the things. | | | | | | | | | | | | | | | | | | |  | |
|  |  | | | | | | | | | | | | | | | | | | |  | |
|  | **Codes\\Intervention experience\Misconceptions** | | | | | | | | | | | | | | | | | | |  | |
|  |  |  | No |  | 0.0119 |  | 1 |  | | | | | | | | | | | |  |  |
|  |  |  |  |  |  |  |  |  | | | | | | | | | | | | | |
|  | | | | | | | | | | 1 |  | | MTK | |  | | 8/4/2021 5:45 AM | |  | |  |
|  | I: Was there anything people were talking because they saw you receiving the intervention?  P: There was nothing that was talked about. | | | | | | | | | | | | | | | | | | |  | |
|  |  | | | | | | | | | | | | | | | | | | |  | |
|  | **Codes\\Intervention process\Delivery** | | | | | | | | | | | | | | | | | | |  | |
|  |  |  | No |  | 0.0650 |  | 1 |  | | | | | | | | | | | |  |  |
|  |  |  |  |  |  |  |  |  | | | | | | | | | | | | | |
|  | | | | | | | | | | 1 |  | | MTK | |  | | 8/4/2021 5:32 AM | |  | |  |
|  | I: What were you discussing when she was visited you?  P: She was teaching me about my health, how I was feeling, how I can deal with stress and problem by having helpful behavior and not to have stress.  I: What else were you discussing apart from teaching you to feel better, having helpful behavior and not to have stress. What else were you discussing?  P: She was teaching me about eating six food groups and when I was pregnant, that would help the child to be healthy and currently she was saying the diet will help the child to suckle fresh breast milk.  I: So you were taught to eat the six food groups so that the child should be health when you were pregnant and that now the child should be sucking fresh breast milk…  P: Yes. | | | | | | | | | | | | | | | | | | |  | |
|  |  |  |  |  |  |  |  |  |  |  |  |  |  |  |  |  |  |  |  |  |  |
|  |  | | | | | | | | | | | | | | | | | | |  | |
|  | **Codes\\Intervention process\Feelings** | | | | | | | | | | | | | | | | | | |  | |
|  |  |  | No |  | 0.0665 |  | 1 |  | | | | | | | | | | | |  |  |
|  |  |  |  |  |  |  |  |  | | | | | | | | | | | | | |
|  | | | | | | | | | | 1 |  | | MTK | |  | | 8/4/2021 5:49 AM | |  | |  |
|  | I: Mh. We are going towards the end of our discussion. You told me that you have experienced change. I don’t remember where… I would like to learn from you what change you have noted from the time you started, how were you feeling before you started receiving the intervention and how you are feeling now…  P: In the past, I was not health but when the counselor started visiting me, I saw that things started to changing little by little, up to a point that now I know am very well, as I am speaking now, I am mentally health.  I: What specifically do you think has changed?  P: My thoughts and feelings.  I: How are you feeling now? Let us talk about today or the past week, how have you been feeling?  P: I am feeling much better now that the past days. | | | | | | | | | | | | | | | | | | |  | |
|  |  |  |  |  |  |  |  |  |  |  |  |  |  |  |  |  |  |  |  |  |  |
|  |  | | | | | | | | | | | | | | | | | | |  | |
|  |  |  |  |  |  |  |  |  |  |  |  |  |  |  |  |  |  |  |  |  |  |
| Formatted Reports\\Coding Summary by File Formatted Report | | | | | | | | | Page 101 of 145 | | | | | | | | | | | |  |
| 8/9/2021 8:08 AM | | | | | | | | | | | | | | | | | | | | |  |
|  | **Classification** |  | **Aggregate** |  | **Coverage** |  | **Number Of Coding References** |  | | **Reference Number** | |  | | **Coded By Initials** | |  | | **Modified On** | |  | |
|  | **Codes\\Intervention process\Home situation** | | | | | | | | | | | | | | | | | | |  | |
|  |  |  | No |  | 0.0574 |  | 1 |  | | | | | | | | | | | |  |  |
|  |  |  |  |  |  |  |  |  | | | | | | | | | | | | | |
|  | | | | | | | | | | 1 |  | | MTK | |  | | 8/4/2021 5:42 AM | |  | |  |
|  | I: What change did you note after sessions?  P: I noted that when we had to work in the garden in the afternoon, they were telling me not to go but rest. That is when I saw that things had changed.  I: When you did not go, were you able to rest or you were doing other chores?  P: This was the time I was able to rest unlike in the past, I was not resting at all.  I: What about communication. You have only said about work…?  P: My partner is polygamous and my sister wife used to talk a lot, we did not have a good relationship but when the counseling intervention started, my mother inlaw intervened, she was told to leave me alone, and things changed. | | | | | | | | | | | | | | | | | | |  | |
|  |  |  |  |  |  |  |  |  |  |  |  |  |  |  |  |  |  |  |  |  |  |
|  |  | | | | | | | | | | | | | | | | | | |  | |
|  | **Codes\\Intervention process\Number of visits** | | | | | | | | | | | | | | | | | | |  | |
|  |  |  | No |  | 0.0103 |  | 1 |  | | | | | | | | | | | |  |  |
|  |  |  |  |  |  |  |  |  | | | | | | | | | | | | | |
|  | | | | | | | | | | 1 |  | | MTK | |  | | 8/4/2021 5:37 AM | |  | |  |
|  | I: How many visits were conducted?  P: Eight visits.  I: How many visits were conducted before you delivered?  P: four. | | | | | | | | | | | | | | | | | | |  | |
|  |  | | | | | | | | | | | | | | | | | | |  | |
|  | **Codes\\Participation of others\Attitude** | | | | | | | | | | | | | | | | | | |  | |
|  |  |  | No |  | 0.0388 |  | 1 |  | | | | | | | | | | | |  |  |
|  |  |  |  |  |  |  |  |  | | | | | | | | | | | | | |
|  | | | | | | | | | | 1 |  | | MTK | |  | | 8/4/2021 5:43 AM | |  | |  |
|  | I: Okay. Is the woman I found you with your mother in law?  P: We just met.  I: I saw that she was carrying your baby and thought you are together…  P: No we just met.  I: How did your mother in law, your partner and the others accept it when they saw you were receiving counseling intervention at home?  P: They did accept it. They were happy, considering the situation I was in. their acceptance helped me to change the way I was thinking. | | | | | | | | | | | | | | | | | | |  | |
|  |  | | | | | | | | | | | | | | | | | | |  | |
|  | **Codes\\Participation of others\Community participation** | | | | | | | | | | | | | | | | | | |  | |
|  |  |  | No |  | 0.0854 |  | 2 |  | | | | | | | | | | | |  |  |
|  |  |  |  |  |  |  |  |  | | | | | | | | | | | | | |
|  | | | | | | | | | | 1 |  | | MTK | |  | | 8/4/2021 5:41 AM | |  | |  |
|  | I: How do you perceive it having your partner and your mother during your counseling intervention session, how helpful was it?  P: [NO RESPONSE]  I: Or was it helpful?  P: It was helpful because it influenced people to change how they communicate to you and how much work I was supposed to be doing at home.  I: Can you elaborate what you are say, change in communication and the amount of work?  P: The way our home is, I was working a lot as if I am not pregnant and their participation in counseling helped them to change their attitude towards me, how they relate to me. They started supporting me.  I: When you say you were working as if you were not pregnant, what do you mean?  P: I was working very hard and that was why it was important that they heard it from the host [counselor]mouth because had it been that I told them, they would think that I was lying. | | | | | | | | | | | | | | | | | | |  | |
|  |  |  |  |  |  |  |  |  |  |  |  |  |  |  |  |  |  |  |  |  |  |
|  |  | | | | | | | | | | | | | | | | | | |  | |
|  |  |  |  |  |  |  |  |  |  |  |  |  |  |  |  |  |  |  |  |  |  |
| Formatted Reports\\Coding Summary by File Formatted Report | | | | | | | | | Page 102 of 145 | | | | | | | | | | | |  |
| 8/9/2021 8:08 AM | | | | | | | | | | | | | | | | | | | | |  |
|  | **Classification** |  | **Aggregate** |  | **Coverage** |  | **Number Of Coding References** |  | | **Reference Number** | |  | | **Coded By Initials** | |  | | **Modified On** | |  | |
|  |  |  |  |  |  |  |  |  |  |  |  |  |  |  |  |  |  |  |  |  |  |
|  | | | | | | | | | | 2 |  | | MTK | |  | | 8/4/2021 5:45 AM | |  | |  |
|  | I: How do you think other people from the community accepted the intervention that women should be visited in their homes?  P: They received it very well. | | | | | | | | | | | | | | | | | | |  | |
|  |  | | | | | | | | | | | | | | | | | | |  | |
|  | **Codes\\Participation of others\Others** | | | | | | | | | | | | | | | | | | |  | |
|  |  |  | No |  | 0.0127 |  | 1 |  | | | | | | | | | | | |  |  |
|  |  |  |  |  |  |  |  |  | | | | | | | | | | | | | |
|  | | | | | | | | | | 1 |  | | MTK | |  | | 8/4/2021 5:38 AM | |  | |  |
|  | I: Was there anyone at your home who participated during the counselling sessions?  P: Yes.  I: Who participated?  P: My partner and my mother. | | | | | | | | | | | | | | | | | | |  | |
|  |  | | | | | | | | | | | | | | | | | | |  | |
|  | **Codes\\Participation of others\Partners** | | | | | | | | | | | | | | | | | | |  | |
|  |  |  | No |  | 0.0127 |  | 1 |  | | | | | | | | | | | |  |  |
|  |  |  |  |  |  |  |  |  | | | | | | | | | | | | | |
|  | | | | | | | | | | 1 |  | | MTK | |  | | 8/4/2021 5:38 AM | |  | |  |
|  | I: Was there anyone at your home who participated during the counselling sessions?  P: Yes.  I: Who participated?  P: My partner and my mother. | | | | | | | | | | | | | | | | | | |  | |
|  |  | | | | | | | | | | | | | | | | | | |  | |
|  | **Codes\\Participation of others\Support** | | | | | | | | | | | | | | | | | | |  | |
|  |  |  | No |  | 0.0658 |  | 2 |  | | | | | | | | | | | |  |  |
|  |  |  |  |  |  |  |  |  | | | | | | | | | | | | | |
|  | | | | | | | | | | 1 |  | | MTK | |  | | 8/4/2021 5:40 AM | |  | |  |
|  | I: How do you perceive it having your partner and your mother during your counseling intervention session, how helpful was it?  P: [NO RESPONSE]  I: Or was it helpful?  P: It was helpful because it influenced people to change how they communicate to you and how much work I was supposed to be doing at home. | | | | | | | | | | | | | | | | | | |  | |
|  |  | | | | | | | | | | | | | | | | | | |  | |
|  | | | | | | | | | | 2 |  | | MTK | |  | | 8/4/2021 5:43 AM | |  | |  |
|  | I: Okay. Is the woman I found you with your mother in law?  P: We just met.  I: I saw that she was carrying your baby and thought you are together…  P: No we just met.  I: How did your mother in law, your partner and the others accept it when they saw you were receiving counseling intervention at home?  P: They did accept it. They were happy, considering the situation I was in. their acceptance helped me to change the way I was thinking. | | | | | | | | | | | | | | | | | | |  | |
|  |  | | | | | | | | | | | | | | | | | | |  | |
|  |  |  |  |  |  |  |  |  |  |  |  |  |  |  |  |  |  |  |  |  |  |
|  |  |  |  |  |  |  |  |  |  |  |  |  |  |  |  |  |  |  |  |  |  |
| Formatted Reports\\Coding Summary by File Formatted Report | | | | | | | | | Page 103 of 145 | | | | | | | | | | | |  |
| 8/9/2021 8:08 AM | | | | | | | | | | | | | | | | | | | | |  |
|  | **Classification** |  | **Aggregate** |  | **Coverage** |  | **Number Of Coding References** |  | | **Reference Number** | |  | | **Coded By Initials** | |  | | **Modified On** | |  | |
|  | **Codes\\Personal history** | | | | | | | | | | | | | | | | | | |  | |
|  |  |  | No |  | 0.1359 |  | 2 |  | | | | | | | | | | | |  |  |
|  |  |  |  |  |  |  |  |  | | | | | | | | | | | | | |
|  | | | | | | | | | | 1 |  | | MTK | |  | | 8/4/2021 5:12 AM | |  | |  |
|  | I: As I said, I am the Principal Investigator of a study that is taking place here in Kabudula together with my colleagues. In this study, we are looking at the community based counseling intervention that is provided by volunteers from different parts of Kabudula. This study started sometime back and our meeting today is to hear from you as one of the participants in this study regarding the intervention, how did it go. We also want to know if this has helped you and how it has helped you. In addition, how was this programme accepted in your community but before we start asking questions about the intervention, I would like to know you. You are?  I: How far did you go with your education?  P: I went up to standard seven.  I: Are you married?  P: Yes.  I: Is this your first marriage?  P: Yes.  I: Okay. How many children do you have?  P: One.  I: This one?  P: Yes.  I: How many pregnancies have you had?  P: One.  I: Which village do you come from?  P: ………...  I: Which TA [Traditional Authority]?  P: Khongoni.  I: Do you have a phone number?  P: No.  I: So if someone wants to find you communication can be made through the counselors?  P: Yes. | | | | | | | | | | | | | | | | | | |  | |
|  |  |  |  |  |  |  |  |  |  |  |  |  |  |  |  |  |  |  |  |  |  |
|  |  |  |  |  |  |  |  |  |  |  |  |  |  |  |  |  |  |  |  |  |  |
|  |  |  |  |  |  |  |  |  |  |  |  |  |  |  |  |  |  |  |  |  |  |
|  |  | | | | | | | | | | | | | | | | | | |  | |
|  | | | | | | | | | | 2 |  | | MTK | |  | | 8/4/2021 5:42 AM | |  | |  |
|  | I: Are you the only wife in your marriage?  P: No, we are two.  P: I am the second.  I: How long have you stayed together?  P: We were married the same year. | | | | | | | | | | | | | | | | | | |  | |
|  |  |  |  |  |  |  |  |  |  |  |  |  |  |  |  |  |  |  |  |  |  |
|  |  | | | | | | | | | | | | | | | | | | |  | |
|  | **Codes\\Place of intervention delivery\Advantages** | | | | | | | | | | | | | | | | | | |  | |
|  |  |  | No |  | 0.0775 |  | 1 |  | | | | | | | | | | | |  |  |
|  |  |  |  |  |  |  |  |  | | | | | | | | | | | | | |
|  | | | | | | | | | | 1 |  | | MTK | |  | | 8/4/2021 5:44 AM | |  | |  |
|  | I: How did you perceive home based counseling intervention? Did you see that it was giving you tough time or is it something you accepted? If you may differentiate between receiving the intervention at home versus receiving it at the clinic?  P: It was very good that you decided to be giving us the counseling intervention at home unlike if we were to be going to the clinic. We were happy to receive the intervention at home  I: But how do you differentiate between receiving the intervention at home and at the clinic? Why do you think is the other reason why home based counseling was good?  P: The difference was that there was no transport used for us to travel from home to this hospital because we use transport when coming here. On the contrary, they, the volunteers were coming home. After the counseling, they were leaving us there without any transport cost on our part. | | | | | | | | | | | | | | | | | | |  | |
|  |  |  |  |  |  |  |  |  |  |  |  |  |  |  |  |  |  |  |  |  |  |
|  |  | | | | | | | | | | | | | | | | | | |  | |
| Formatted Reports\\Coding Summary by File Formatted Report | | | | | | | | | Page 104 of 145 | | | | | | | | | | | |  |
| 8/9/2021 8:08 AM | | | | | | | | | | | | | | | | | | | | |  |
|  | **Classification** |  | **Aggregate** |  | **Coverage** |  | **Number Of Coding References** |  | | **Reference Number** | |  | | **Coded By Initials** | |  | | **Modified On** | |  | |
|  | **Codes\\Recommendations\Areas to be changed** | | | | | | | | | | | | | | | | | | |  | |
|  |  |  | No |  | 0.0115 |  | 1 |  | | | | | | | | | | | |  |  |
|  |  |  |  |  |  |  |  |  | | | | | | | | | | | | | |
|  | | | | | | | | | | 1 |  | | MTK | |  | | 8/4/2021 5:46 AM | |  | |  |
|  | I: Is there anything you did not like about the counseling itself which you think if there was change it could improve?  P: Nothing. | | | | | | | | | | | | | | | | | | |  | |
|  |  | | | | | | | | | | | | | | | | | | |  | |
|  | **Codes\\THPP providers** | | | | | | | | | | | | | | | | | | |  | |
|  |  |  | No |  | 0.0938 |  | 2 |  | | | | | | | | | | | |  |  |
|  |  |  |  |  |  |  |  |  | | | | | | | | | | | | | |
|  | | | | | | | | | | 1 |  | | MTK | |  | | 8/4/2021 5:26 AM | |  | |  |
|  | I: And I also wanted to assure you that we are not going to use your name anywhere. We asked about your name so that we connect you with the person who was getting the intervention in this study. So feel free. Where you feel like you want to breastfeed the child, you may tell me to stop. Our discussion is not going to take long. So firstly, I would like to hear more from you about the counseling intervention that you were receiving. How was the intervention?  P: The intervention that I was receiving…  I: Raise your voice a bit…  P: Is this audio recording?  I: Yes. So you should be speaking as I am speaking so that it should be heard.  P: Okay. I am very thankful for the intervention that I was receiving. The counselor that you gave us was very committed and she was consistently coming on the day that you scheduled her on Mondays. She tried to come on all days agreed | | | | | | | | | | | | | | | | | | |  | |
|  |  |  |  |  |  |  |  |  |  |  |  |  |  |  |  |  |  |  |  |  |  |
|  |  | | | | | | | | | | | | | | | | | | |  | |
|  | | | | | | | | | | 2 |  | | MTK | |  | | 8/4/2021 5:51 AM | |  | |  |
|  | I: Would you like the intervention to be provided by a health worker versus the volunteer counselors who were visiting you?  P: The counselors are okay.  I: The same counselors…  P: Yes. | | | | | | | | | | | | | | | | | | |  | |
|  |  | | | | | | | | | | | | | | | | | | |  | |
|  | **Codes\\THPP providers\Characteristics** | | | | | | | | | | | | | | | | | | |  | |
|  |  |  | No |  | 0.0406 |  | 1 |  | | | | | | | | | | | |  |  |
|  |  |  |  |  |  |  |  |  | | | | | | | | | | | | | |
|  | | | | | | | | | | 1 |  | | MTK | |  | | 8/4/2021 5:46 AM | |  | |  |
|  | I: Now I want to talk about the counselor who was visiting you; what behaviors did you like from the counselor?  P: [NO RESPONSE]  I: What type of person was she?  P: My counselor was a good person and when we completed the programme, she told me that “although we have completed the visits, if you have any issue, you are free to come to my home so that I can help you to continue changing”.  I: Was there anything you did not like about the counselor?  P: No. | | | | | | | | | | | | | | | | | | |  | |
|  |  |  |  |  |  |  |  |  |  |  |  |  |  |  |  |  |  |  |  |  |  |
|  |  | | | | | | | | | | | | | | | | | | |  | |
|  |  |  |  |  |  |  |  |  |  |  |  |  |  |  |  |  |  |  |  |  |  |
|  |  |  |  |  |  |  |  |  |  |  |  |  |  |  |  |  |  |  |  |  |  |
| Formatted Reports\\Coding Summary by File Formatted Report | | | | | | | | | Page 105 of 145 | | | | | | | | | | | |  |
| 8/9/2021 8:08 AM | | | | | | | | | | | | | | | | | | | | |  |
|  | **Classification** |  | **Aggregate** |  | **Coverage** |  | **Number Of Coding References** |  | | **Reference Number** | |  | | **Coded By Initials** | |  | | **Modified On** | |  | |
|  | **Files\\IDIs\\IDI #4** | | | | | | | | | | | | | | | | | | |  | |
|  | **Code** | | | | | | | | | | | | | | | | | | |  | |
|  | **Codes\\Intervention experience\Aspects** | | | | | | | | | | | | | | | | | | |  | |
|  |  |  | No |  | 0.0788 |  | 2 |  | | | | | | | | | | | |  |  |
|  |  |  |  |  |  |  |  |  | | | | | | | | | | | | | |
|  | | | | | | | | | | 1 |  | | MTK | |  | | 8/4/2021 8:42 AM | |  | |  |
|  | I: What part of counseling do you find to be most helpful?  P: The part?  I: Or what counseling was provided to you?  P: Exclusive breastfeeding, feeding the child, eating six food groups. That was what she was teaching me.  I: What else?  P: Associating with people surrounding me.  I: How helpful do you think this was?  P: I will give an example of my child. There is my elder sister from my other mother. She was not talking to me since the birth of my child and she was not even touching my child and I even thought that she was capable of harming my child. When the counselor came, she said that my sister should join the discussion and when I called her she came. After the sessions, she changed her attitude and we interact very well and she is able to assist me with my child. | | | | | | | | | | | | | | | | | | |  | |
|  |  |  |  |  |  |  |  |  |  |  |  |  |  |  |  |  |  |  |  |  |  |
|  |  | | | | | | | | | | | | | | | | | | |  | |
|  | | | | | | | | | | 2 |  | | MTK | |  | | 8/4/2021 8:45 AM | |  | |  |
|  | I: if you may think about all the six sessions that the counselor had conducted, which one do you find to be the most helpful?  P: In terms of the stress that I was talking about, I was really helped. because as I said, this stress would have ruined my life. | | | | | | | | | | | | | | | | | | |  | |
|  |  | | | | | | | | | | | | | | | | | | |  | |
|  | **Codes\\Intervention experience\Barriers** | | | | | | | | | | | | | | | | | | |  | |
|  |  |  | No |  | 0.0051 |  | 1 |  | | | | | | | | | | | |  |  |
|  |  |  |  |  |  |  |  |  | | | | | | | | | | | | | |
|  | | | | | | | | | | 1 |  | | MTK | |  | | 8/4/2021 8:46 AM | |  | |  |
|  | I: Were there any barriers to the counseling intervention?  P: No. | | | | | | | | | | | | | | | | | | |  | |
|  |  | | | | | | | | | | | | | | | | | | |  | |
|  | **Codes\\Intervention experience\Facilitators** | | | | | | | | | | | | | | | | | | |  | |
|  |  |  | No |  | 0.0198 |  | 1 |  | | | | | | | | | | | |  |  |
|  |  |  |  |  |  |  |  |  | | | | | | | | | | | | | |
|  | | | | | | | | | | 1 |  | | MTK | |  | | 8/4/2021 8:46 AM | |  | |  |
|  | I: Were you able to do it every day when she came?  P: Yes we were doing it every day.  I: Okay fine. Was there anything at home that influenced the intervention at home to go on well?  P: Yes mainly on care of the child, I was able to breastfeeding the child. | | | | | | | | | | | | | | | | | | |  | |
|  |  | | | | | | | | | | | | | | | | | | |  | |
|  | **Codes\\Intervention experience\Influence on others** | | | | | | | | | | | | | | | | | | |  | |
|  |  |  | No |  | 0.0387 |  | 1 |  | | | | | | | | | | | |  |  |
|  |  |  |  |  |  |  |  |  | | | | | | | | | | | | | |
|  | | | | | | | | | | 1 |  | | MTK | |  | | 8/4/2021 8:48 AM | |  | |  |
|  | I: Can you encourage a fellow woman to receive the intervention if we were to continue?  P: Yes I can.  I: How can you influence the woman who doesn’t know anything about the program, what can you tell her?  P: You can tell that a person is depressed, so I would tell them that; “my friend, your behavior shows that you are depressed and this is what I also went through. So if you can do this and that, you can live a healthy life.” If she can take part in such a programme she can be assisted, she can change. | | | | | | | | | | | | | | | | | | |  | |
|  |  | | | | | | | | | | | | | | | | | | |  | |
| Formatted Reports\\Coding Summary by File Formatted Report | | | | | | | | | Page 106 of 145 | | | | | | | | | | | |  |
| 8/9/2021 8:08 AM | | | | | | | | | | | | | | | | | | | | |  |
|  | **Classification** |  | **Aggregate** |  | **Coverage** |  | **Number Of Coding References** |  | | **Reference Number** | |  | | **Coded By Initials** | |  | | **Modified On** | |  | |
|  | **Codes\\Intervention experience\Likes** | | | | | | | | | | | | | | | | | | |  | |
|  |  |  | No |  | 0.0417 |  | 1 |  | | | | | | | | | | | |  |  |
|  |  |  |  |  |  |  |  |  | | | | | | | | | | | | | |
|  | | | | | | | | | | 1 |  | | MTK | |  | | 8/4/2021 8:48 AM | |  | |  |
|  | I: Okay fine. If you may put in summary, what is your comment on the intervention and how has it helped you?  P: How I feel now?  I: Yes how you feel now comparing with how you were feeling before…  P: My worries are minimized now after receiving the intervention. Because I was very worried because my partner called me telling me that all my kitchen utensils were stolen and in addition, the people I stay with here were saying I should leave their home. In addition to that, my relatives were not talking to me. That was what was worrying me the most. | | | | | | | | | | | | | | | | | | |  | |
|  |  | | | | | | | | | | | | | | | | | | |  | |
|  | **Codes\\Intervention process\Feelings** | | | | | | | | | | | | | | | | | | |  | |
|  |  |  | No |  | 0.0588 |  | 1 |  | | | | | | | | | | | |  |  |
|  |  |  |  |  |  |  |  |  | | | | | | | | | | | | | |
|  | | | | | | | | | | 1 |  | | MTK | |  | | 8/4/2021 8:41 AM | |  | |  |
|  | I: Okay fine. What can you tell me about the intervention that you were getting?  P: This intervention was helpful because the way I was depressed in the past, I had many thoughts but she helped me to change the way I was thinking and I now I experience change in my thoughts.  I: So you have said that in the past you were thinking too much. Can you share me the thoughts that you had or the burden that you had and how the situation has changed because of this intervention?  P: In the past, I was thinking too much. My thoughts were to hung myself up or to drown in a river and when I explained this to my counselor, she helped me to have helpful thoughts to replace the unhelpful thoughts that I had. To her it was an easy thing to address this and this was really addressed. | | | | | | | | | | | | | | | | | | |  | |
|  |  |  |  |  |  |  |  |  |  |  |  |  |  |  |  |  |  |  |  |  |  |
|  |  | | | | | | | | | | | | | | | | | | |  | |
|  | **Codes\\Intervention process\Home situation** | | | | | | | | | | | | | | | | | | |  | |
|  |  |  | No |  | 0.0433 |  | 1 |  | | | | | | | | | | | |  |  |
|  |  |  |  |  |  |  |  |  | | | | | | | | | | | | | |
|  | | | | | | | | | | 1 |  | | MTK | |  | | 8/4/2021 8:44 AM | |  | |  |
|  | I: Who else was involved apart from your sister?  P: My grandmother.  I: So she was invited as well?  P: Yes.  I: Do you think your relationship with these people was affected by the counseling?  P: To say the truth, my grandmother is not able to change even after the counseling. She participates during the discussion but she is not able to change afterwards. But for my sister, she has changed.  I: She changed… does she support you in caring for the child?  P: Yes she supports me.  I: So which part of the counseling did you find to be more helpful?  P: [NO RESPONSE] | | | | | | | | | | | | | | | | | | |  | |
|  |  |  |  |  |  |  |  |  |  |  |  |  |  |  |  |  |  |  |  |  |  |
|  |  | | | | | | | | | | | | | | | | | | |  | |
|  |  | | | | | | | | | | | | | | | | | | |  | |
|  |  | | | | | | | | | | | | | | | | | | |  | |
| Formatted Reports\\Coding Summary by File Formatted Report | | | | | | | | | Page 107 of 145 | | | | | | | | | | | |  |
| 8/9/2021 8:08 AM | | | | | | | | | | | | | | | | | | | | |  |
|  | **Classification** |  | **Aggregate** |  | **Coverage** |  | **Number Of Coding References** |  | | **Reference Number** | |  | | **Coded By Initials** | |  | | **Modified On** | |  | |
|  | **Codes\\Other interesting issues** | | | | | | | | | | | | | | | | | | |  | |
|  |  |  | No |  | 0.1024 |  | 1 |  | | | | | | | | | | | |  |  |
|  |  |  |  |  |  |  |  |  | | | | | | | | | | | | | |
|  | | | | | | | | | | 1 |  | | MTK | |  | | 8/4/2021 8:56 AM | |  | |  |
|  | I: These are the questions I had for you. I don’t know if you have anything to add?  R: Mine is a complaint as I told you that people where I am staying, they are saying that I should leave and the father of the child does not provide the support. So that is my complaint.  I: You have said that the father of the child is in ……..?  R: Yes in …….. and he stole my belongings.  I: You have said that he has never seen the child since you came here?  R: No.  I: Okay. Was this a formal marriage?  R: No.  I: So there is no one who can follow up this issue in terms of reuniting you or following up on your belongings?  R: None because the uncle I have is a distant one. I have relations but they are distant ones and they are not the type of people who can be able to assist because even the grandmother I am staying with, when I tell her she says that she has her own problems and I have to be able to find a solution to my problem.  I: How about chiefs?  R: Chiefs can assist.  I: Maybe if you can seek assistance from them they can help.  R: Okay.  I: As I said earlier, we are conducting this study so that we can see areas that we can improve in this intervention in future. So we are asking the participants in this program as well as the counselors themselves regarding the challenges they were experiencing. So, I thank you very much for your time. However on your issue, I will talk to the volunteer to introduce you to the Chief, so you can present your problem | | | | | | | | | | | | | | | | | | |  | |
|  |  |  |  |  |  |  |  |  |  |  |  |  |  |  |  |  |  |  |  |  |  |
|  |  |  |  |  |  |  |  |  |  |  |  |  |  |  |  |  |  |  |  |  |  |
|  |  | | | | | | | | | | | | | | | | | | |  | |
|  | **Codes\\Participation of others\Attitude** | | | | | | | | | | | | | | | | | | |  | |
|  |  |  | No |  | 0.1457 |  | 2 |  | | | | | | | | | | | |  |  |
|  |  |  |  |  |  |  |  |  | | | | | | | | | | | | | |
|  | | | | | | | | | | 1 |  | | MTK | |  | | 8/4/2021 8:43 AM | |  | |  |
|  | I: What part of counseling do you find to be most helpful?  P: The part?  I: Or what counseling was provided to you?  P: Exclusive breastfeeding, feeding the child, eating six food groups. That was what she was teaching me.  I: What else?  P: Associating with people surrounding me.  I: How helpful do you think this was?  P: I will give an example of my child. There is my elder sister from my other mother. She was not talking to me since the birth of my child and she was not even touching my child and I even thought that she was capable of harming my child. When the counselor came, she said that my sister should join the discussion and when I called her she came. After the sessions, she changed her attitude and we interact very well and she is able to assist me with my child. | | | | | | | | | | | | | | | | | | |  | |
|  |  |  |  |  |  |  |  |  |  |  |  |  |  |  |  |  |  |  |  |  |  |
|  |  | | | | | | | | | | | | | | | | | | |  | |
|  | | | | | | | | | | 2 |  | | MTK | |  | | 8/4/2021 8:50 AM | |  | |  |
|  | I: How did the other people who were taking part perceiving it that the counselor was coming to provide the intervention?  P: For some as I said, when the counselor was there all was well, but when she left my grandmother would change, she was angry.  I: Why do you think she was angry?  P: She thought that I am being given money but I don’t want to support the home.  I: Did you report to the counselor?  P: Yes.  I: Did the counselor tell her that the intervention was not providing any financial support?  R: No she only told me but she did not tell her.  I: How about your sister?  R: To say the truth, she was very eager to be part of the intervention. She was always ready for the session and she has also benefitted because of what she is also going through in her life.  I: In what way do you think she has benefitted?  R: Her partner was having multiple sexual partners and she was always fighting the other women. One day, one of the concubines came and she wanted to fight but when she thought about what we have been learning at my home, she thoughts it was a useless thing and she told me that “I cannot benefit anything from this”. | | | | | | | | | | | | | | | | | | |  | |
|  |  |  |  |  |  |  |  |  |  |  |  |  |  |  |  |  |  |  |  |  |  |
|  |  |  |  |  |  |  |  |  |  |  |  |  |  |  |  |  |  |  |  |  |  |
|  |  | | | | | | | | | | | | | | | | | | |  | |
| Formatted Reports\\Coding Summary by File Formatted Report | | | | | | | | | Page 108 of 145 | | | | | | | | | | | |  |
| 8/9/2021 8:08 AM | | | | | | | | | | | | | | | | | | | | |  |
|  | **Classification** |  | **Aggregate** |  | **Coverage** |  | **Number Of Coding References** |  | | **Reference Number** | |  | | **Coded By Initials** | |  | | **Modified On** | |  | |
|  | **Codes\\Participation of others\Community participation** | | | | | | | | | | | | | | | | | | |  | |
|  |  |  | No |  | 0.0780 |  | 1 |  | | | | | | | | | | | |  |  |
|  |  |  |  |  |  |  |  |  | | | | | | | | | | | | | |
|  | | | | | | | | | | 1 |  | | MTK | |  | | 8/4/2021 8:50 AM | |  | |  |
|  | I: How did the other people from the community perceive the counseling intervention being provided at home?  R: They were talking a lot that “these people simply want to take you for granted but they are not helpful at all.” So I was telling them that “let people learn from me because I am the one who has started this thing from this community.”  I: Apart from saying that they are taking you for granted, what else were they saying?  R: They were saying that “they cannot give you money these people. This is satanic” there were many things people were talking about.  I: Were they made aware of what the intervention was all about?  R: They were asking me “what does she do when she comes?” I was telling them that “we are discussing about depression in women”. “How does depression in women look like?” It was like they are challenging me. I was telling them that “take it or not but I was depressed and my issues are being addressed”. “What issues did you have”” I have been explaining to them but they choose not to understand. | | | | | | | | | | | | | | | | | | |  | |
|  |  |  |  |  |  |  |  |  |  |  |  |  |  |  |  |  |  |  |  |  |  |
|  |  | | | | | | | | | | | | | | | | | | |  | |
|  | **Codes\\Participation of others\Others** | | | | | | | | | | | | | | | | | | |  | |
|  |  |  | No |  | 0.0433 |  | 1 |  | | | | | | | | | | | |  |  |
|  |  |  |  |  |  |  |  |  | | | | | | | | | | | | | |
|  | | | | | | | | | | 1 |  | | MTK | |  | | 8/4/2021 8:44 AM | |  | |  |
|  | I: Who else was involved apart from your sister?  P: My grandmother.  I: So she was invited as well?  P: Yes.  I: Do you think your relationship with these people was affected by the counseling?  P: To say the truth, my grandmother is not able to change even after the counseling. She participates during the discussion but she is not able to change afterwards. But for my sister, she has changed.  I: She changed… does she support you in caring for the child?  P: Yes she supports me.  I: So which part of the counseling did you find to be more helpful?  P: [NO RESPONSE] | | | | | | | | | | | | | | | | | | |  | |
|  |  |  |  |  |  |  |  |  |  |  |  |  |  |  |  |  |  |  |  |  |  |
|  |  | | | | | | | | | | | | | | | | | | |  | |
|  |  |  |  |  |  |  |  |  |  |  |  |  |  |  |  |  |  |  |  |  |  |
|  |  |  |  |  |  |  |  |  |  |  |  |  |  |  |  |  |  |  |  |  |  |
|  |  |  |  |  |  |  |  |  |  |  |  |  |  |  |  |  |  |  |  |  |  |
|  |  |  |  |  |  |  |  |  |  |  |  |  |  |  |  |  |  |  |  |  |  |
| Formatted Reports\\Coding Summary by File Formatted Report | | | | | | | | | Page 109 of 145 | | | | | | | | | | | |  |
| 8/9/2021 8:08 AM | | | | | | | | | | | | | | | | | | | | |  |
|  | **Classification** |  | **Aggregate** |  | **Coverage** |  | **Number Of Coding References** |  | | **Reference Number** | |  | | **Coded By Initials** | |  | | **Modified On** | |  | |
|  | **Codes\\Personal history** | | | | | | | | | | | | | | | | | | |  | |
|  |  |  | No |  | 0.3440 |  | 1 |  | | | | | | | | | | | |  |  |
|  |  |  |  |  |  |  |  |  | | | | | | | | | | | | | |
|  | | | | | | | | | | 1 |  | | MTK | |  | | 8/4/2021 8:39 AM | |  | |  |
|  | I: Yes November when you were joining the program. Myself with my two other collegues, the one I was with outside, as you know, we are conducting a study here in Kabudula. This study is about the counseling intervention that was provided by the volunteers in the communities. So as one of the participants in this study on thinking about the health of the mother and the child, we have invited you today in order to learn how this intervention has been conducted. That is the main aim of our discussion. What is your name?  I: … You said you come from…?  P: I come from ……… village but because my marriage is not working, I am currently at my home village in …………………..  I: Where were you staying when you were joining the study?  P: When I was joining the study I was at ………...  P: Yes since my delivery in December I went to my home village after discharge.  I: So before we start, I just want to confirm that as I said at the beginning, the study processes are that all the information that we collect from you is kept confidential and our discussion today will be kept confidential. We are going to use it only for study purposes. The aim of the discussion is to help us improve or change where necessary depending on what you are going to tell us. So feel free to tell me everything that you can tell me concerning the intervention that you were receiving. Firstly, I would like to know how old you are…  P: I am 19 years old.  I: How far did you go with your education?  P: Standard seven.  I: So you told me that you moved from your matrimonial home and went to stay in your home village. What happened?  P: What happened?  I: Yes…  P: For me to go my home village?  I: Yes…  P: Before getting married, I was working in town and my sister was staying at ……. So I went to visit my sister and that was when I met the man. [THERE IS NOISE OF CAR SOUND IN THE BACKGROUND AND THE AUDIO WAS PAUSED]  I: So you were explaining to me about what happened to you…  P: Yes.  I: Yes so you can start again…  P: I was staying in ……… where I was working. My mother died in 2016, I am the last born in our family, she died when I was young. So as I was working there I went to visit my sister who was staying at …………., I met this man who proposed to me but he was already married. He said “ I want to marry you because my spouse steals money from me and I want to end the marriage so that she goes to her home village. You look like a better marriage partner and I want to take care of you as you have said that you are an orphan.” I said that the way he is talking, he can assist me. We got married and stayed at …………. I became pregnant until December when I came here for delivery. Unfortunately, I delivered through Caesarian section and after discharge I called him to pick us. To my surprise, he came on a bicycle. That’s when he told me that “the child is does not belong to him and you need to take her/him to your home village. You are going to take care of the wound there.” I did not argue but I just said “that is your plan so let it be.” We walked on foot while I was sick. We walked slowly until we reached home. Two days later, the suture on the operation wound lossened because I walked a long distance with a fresh wound. And I came back here at the hospital for re-suturing. Since then, he does not come even if I call him, he just promises to come but he doesn’t. He does not even provide support for the child. So I need to do piece work to find soap and I am staying with my distant grandmother who scolds me saying “you should leave my home because you do not provide any support”. “How do I provide support as the piece works are scarce nowadays and the only money I find from piece work is K200 which I buy soap for the child. How can I buy food for the home if I am not able to find piece work? Since it is your wish, I will do what you tell me. ” “You should leave this home.” This is always her statement. That was the reason why I left the marriage.  I: Okay. So we can say that you left your husband?  P: Yes.  I: You are with your distant granny as you just described?  P: Yes.  I: Do you have any close relative nearby?  P: My real grand mother passed, my elder sister passed. I initially went to town because she [grandmother] chased me out of her house. I walked on foot to go to town ……..  I: How many children do you have?  P: This is my first born.  I: First born… Do you have a phone number?  P: I use the volunteer’s phone number. | | | | | | | | | | | | | | | | | | |  | |
|  |  |  |  |  |  |  |  |  |  |  |  |  |  |  |  |  |  |  |  |  |  |
|  |  |  |  |  |  |  |  |  |  |  |  |  |  |  |  |  |  |  |  |  |  |
|  |  |  |  |  |  |  |  |  |  |  |  |  |  |  |  |  |  |  |  |  |  |
|  |  |  |  |  |  |  |  |  |  |  |  |  |  |  |  |  |  |  |  |  |  |
|  |  |  |  |  |  |  |  |  |  |  |  |  |  |  |  |  |  |  |  |  |  |
|  |  |  |  |  |  |  |  |  |  |  |  |  |  |  |  |  |  |  |  |  |  |
|  |  |  |  |  |  |  |  |  |  |  |  |  |  |  |  |  |  |  |  |  |  |
|  |  | | | | | | | | | | | | | | | | | | |  | |
|  |  |  |  |  |  |  |  |  |  |  |  |  |  |  |  |  |  |  |  |  |  |
| Formatted Reports\\Coding Summary by File Formatted Report | | | | | | | | | Page 110 of 145 | | | | | | | | | | | |  |
| 8/9/2021 8:08 AM | | | | | | | | | | | | | | | | | | | | |  |
|  | **Classification** |  | **Aggregate** |  | **Coverage** |  | **Number Of Coding References** |  | | **Reference Number** | |  | | **Coded By Initials** | |  | | **Modified On** | |  | |
|  | **Codes\\Recommendations\Place of delivery** | | | | | | | | | | | | | | | | | | |  | |
|  |  |  | No |  | 0.0262 |  | 1 |  | | | | | | | | | | | |  |  |
|  |  |  |  |  |  |  |  |  | | | | | | | | | | | | | |
|  | | | | | | | | | | 1 |  | | MTK | |  | | 8/4/2021 8:46 AM | |  | |  |
|  | I: So how did you perceive the intervention being provided at home and not at the clinic? How feasible was it for you to receive the intervention at home?  P: Feasibility?  I: Or I should say did you like receiving the intervention at home?  P: I would have liked either way because at home, it was good because I wanted other people to take part. | | | | | | | | | | | | | | | | | | |  | |
|  |  | | | | | | | | | | | | | | | | | | |  | |
|  |  | | | | | | | | | | | | | | | | | | |  | |
|  |  | | | | | | | | | | | | | | | | | | |  | |
|  | **Codes\\THPP providers\Characteristics** | | | | | | | | | | | | | | | | | | |  | |
|  |  |  | No |  | 0.0159 |  | 1 |  | | | | | | | | | | | |  |  |
|  |  |  |  |  |  |  |  |  | | | | | | | | | | | | | |
|  | | | | | | | | | | 1 |  | | MTK | |  | | 8/4/2021 8:47 AM | |  | |  |
|  | I: Can you describe the behavior of the counselor?  P: The counselor was a loving person and she was doing exactly what she was trained to do on us.  I: How is her behavior?  P: She is well behaved and exemplary. | | | | | | | | | | | | | | | | | | |  | |
|  |  | | | | | | | | | | | | | | | | | | |  | |
|  |  |  |  |  |  |  |  |  |  |  |  |  |  |  |  |  |  |  |  |  |  |
|  |  |  |  |  |  |  |  |  |  |  |  |  |  |  |  |  |  |  |  |  |  |
|  |  |  |  |  |  |  |  |  |  |  |  |  |  |  |  |  |  |  |  |  |  |
| Formatted Reports\\Coding Summary by File Formatted Report | | | | | | | | | Page 111 of 145 | | | | | | | | | | | |  |
| 8/9/2021 8:08 AM | | | | | | | | | | | | | | | | | | | | |  |
|  | **Classification** |  | **Aggregate** |  | **Coverage** |  | **Number Of Coding References** |  | | **Reference Number** | |  | | **Coded By Initials** | |  | | **Modified On** | |  | |
|  | **Files\\IDIs\\IDI #5** | | | | | | | | | | | | | | | | | | |  | |
|  | **Code** | | | | | | | | | | | | | | | | | | |  | |
|  | **Codes\\Intervention experience** | | | | | | | | | | | | | | | | | | |  | |
|  |  |  | No |  | 0.0620 |  | 1 |  | | | | | | | | | | | |  |  |
|  |  |  |  |  |  |  |  |  | | | | | | | | | | | | | |
|  | | | | | | | | | | 1 |  | | MTK | |  | | 8/4/2021 8:59 AM | |  | |  |
|  | I: Okay? The first question; can you tell me more about the counseling intervention that you received?  R: It was a good intervention.  I: It was a good intervention? …  R: Yes because they taught us many things that we were not able to know.  I: Mh. You have learnt many things that you did not know… On that part, I want you to emphasize on the section of the counseling that was very helpful. On sections of the intervention, you remember we were talking about the health of the mother, health of the baby and relationship with other people, right?  R: Yes.  I: Then people that surrounded her…  R: Yes. | | | | | | | | | | | | | | | | | | |  | |
|  |  |  |  |  |  |  |  |  |  |  |  |  |  |  |  |  |  |  |  |  |  |
[truncated: 118,160 more chars]
