# Supplementary material for: Piloting a psychosocial intervention for perinatal depression, the Thinking Healthy Programme–Peer delivered (THPP), in a primary care setting in Lilongwe District, Malawi
Source: PLOS Glob Public Health. 2024 May 1;4(5):e0002128. doi: 10.1371/journal.pgph.0002128 (PMC11062519; doi:10.1371/journal.pgph.0002128)
Supplement: S8 File — (DOCX) [file pgph.0002128.s008.docx]

| 7/5/2021 9:43 AM | | | | | | | | | | | | | | | | | |
| --- | --- | --- | --- | --- | --- | --- | --- | --- | --- | --- | --- | --- | --- | --- | --- | --- | --- |
| Coding Summary by File | | | | | | | | | | | | | | | | | |
| THPP Feasibility | | | | | | | | | | | | | | | | | |
| 7/5/2021 9:43 AM | | | | | | | | | | | | | | | | | |
|  | | | **Classification** |  | **Aggregate** |  | **Coverage** |  | **Number Of Coding References** |  | | **Reference Number** |  | **Coded By Initials** |  | **Modified On** |  |
| **Document** | | | | | | | | | | | | | | | | |  |
|  | **Files\\THPP PV_#01** | | | | | | | | | | | | | | | |  |
|  | | **Code** | | | | | | | | | | | | | | |  |
|  | | | **Codes\\Health workers** | | | | | | | | | | | | | |  |
|  |  |  |  |  | No |  | 0.0232 |  | 1 |  | | | | | | |  |
|  | | |  |  |  |  |  |  |  |  | | | | | | | |
|  | | | | | | | | | | | | 1 |  | MTK |  | 6/30/2021 9:36 PM |  |
|  | | | I: Thank you, we are towards the end of our discussion this afternoon and my next question is as part of the study you were working together with the women and doctors/hospital staff not so?  P: Yes  I: Can you explain how you worked with the doctors/hospital staff?  P: I worked with the women and all the results were given to the doctor/hospital staff, I was giving a detailed report on who I visited and how it went so we worked together with a doctors/hospital staff | | | | | | | | | | | | | |  |
|  | | |  | | | | | | | | | | | | | |  |
|  | | | **Codes\\Health workers\Interaction** | | | | | | | | | | | | | |  |
|  |  |  |  |  | No |  | 0.0184 |  | 1 |  | | | | | | |  |
|  | | |  |  |  |  |  |  |  |  | | | | | | | |
|  | | | | | | | | | | | | 1 |  | MTK |  | 6/30/2021 9:37 PM |  |
|  | | | I: Can you explain how you worked with the doctors/hospital staff?  P: I worked with the women and all the results were given to the doctor/hospital staff, I was giving a detailed report on who I visited and how it went so we worked together with a doctors/hospital staff  I: Were there any problems working with the doctor?  P: I did not have any problems from the doctor? | | | | | | | | | | | | | |  |
|  | | |  | | | | | | | | | | | | | |  |
|  | | | **Codes\\Involvement of family members** | | | | | | | | | | | | | |  |
|  |  |  |  |  | No |  | 0.0616 |  | 2 |  | | | | | | |  |
|  | | |  |  |  |  |  |  |  |  | | | | | | | |
|  | | | | | | | | | | | | 1 |  | MTK |  | 6/30/2021 9:32 PM |  |
|  | | | I: Thank you very much, during the time you were visiting the women and the family what part did the partner or the mother/mother in-law of the woman take part in the counselling sessions or what really happened? Tell us more about the people surrounding this woman like the husband, mother-in-law or other relatives  P: They were involved in sessions, we were using books that had pictures during the lessons and I could ask the mother –in-law together with the woman what is in picture and in return the mother could help talk to the women, talk about her behavior in relation to what was in the pictures , they would ask the woman to relax and talk about what is bothering her and reassured the woman that they could help if she has problems, so I can say that the mother –in-law and the relatives were involved the time we were having sessions with the woman. | | | | | | | | | | | | | |  |
|  | | |  |  |  |  |  |  |  |  |  |  |  |  |  |  |  |
|  | | |  | | | | | | | | | | | | | |  |
|  | | | | | | | | | | | | | | | | | |
| Formatted Reports\\Coding Summary by File Formatted Report | | | | | | | | | | | Page 1 of 53 | | | | | | |
| 7/5/2021 9:43 AM | | | | | | | | | | | | | | | | | |
|  | | | **Classification** |  | **Aggregate** |  | **Coverage** |  | **Number Of Coding References** |  | | **Reference Number** |  | **Coded By Initials** |  | **Modified On** |  |
|  | | | | | | | | | | | | | | | | | |
|  | | | | | | | | | | | | 2 |  | MTK |  | 6/30/2021 9:32 PM |  |
|  | | | I: Can you say that the mother-in-law and the relatives saw that these counselling sessions were helpful?  P: Yes  I: Did you have any other problems like people who did not want to chart with you like some family members or those who were not happy with the help brought by you. Has this ever happened to you?  P: No there is nothing that happened like that | | | | | | | | | | | | | |  |
|  | | |  | | | | | | | | | | | | | |  |
|  | | | **Codes\\Personal history** | | | | | | | | | | | | | |  |
|  |  |  |  |  | No |  | 0.0096 |  | 1 |  | | | | | | |  |
|  | | |  |  |  |  |  |  |  |  | | | | | | | |
|  | | | | | | | | | | | | 1 |  | MTK |  | 6/30/2021 8:19 PM |  |
|  | | | I: now that you have settled at this place, how do you find you daily needs like soap and relish what do you do?  P: We do some piece jobs and we purchased a land where we do farming to get money. | | | | | | | | | | | | | |  |
|  | | |  | | | | | | | | | | | | | |  |
|  | | | **Codes\\Personal history\Age** | | | | | | | | | | | | | |  |
|  |  |  |  |  | No |  | 0.0081 |  | 1 |  | | | | | | |  |
|  | | |  |  |  |  |  |  |  |  | | | | | | | |
|  | | | | | | | | | | | | 1 |  | MTK |  | 6/30/2021 8:12 PM |  |
|  | | | P: I was born in 1987  I: If we do mathematics, have many years do you have?  P: 33 or 34?  I: okay, may be 34 which month?  P: 18 August  I: 18 August 1987?  P: Yes | | | | | | | | | | | | | |  |
|  | | |  |  |  |  |  |  |  |  |  |  |  |  |  |  |  |
|  | | |  | | | | | | | | | | | | | |  |
|  | | | **Codes\\Personal history\Contact information** | | | | | | | | | | | | | |  |
|  |  |  |  |  | No |  | 0.0148 |  | 1 |  | | | | | | |  |
|  | | |  |  |  |  |  |  |  |  | | | | | | | |
|  | | | | | | | | | | | | 1 |  | MTK |  | 6/30/2021 9:09 PM |  |
|  | | | I: Thank you very much, do you have an address which you use for communication where you stay?  P: No, I don’t have an address  I: Do you have a cellphone for communication?  P: Yes I have a phone  I: Do you know the number and can you tell me?  P: Yes I know it  I: Tell me the number  P: | | | | | | | | | | | | | |  |
|  | | |  |  |  |  |  |  |  |  |  |  |  |  |  |  |  |
|  | | |  | | | | | | | | | | | | | |  |
|  | | | | | | | | | | | | | | | | | |
|  | | | | | | | | | | | | | | | | | |
| Formatted Reports\\Coding Summary by File Formatted Report | | | | | | | | | | | Page 2 of 53 | | | | | | |
| 7/5/2021 9:43 AM | | | | | | | | | | | | | | | | | |
|  | | | **Classification** |  | **Aggregate** |  | **Coverage** |  | **Number Of Coding References** |  | | **Reference Number** |  | **Coded By Initials** |  | **Modified On** |  |
|  | | | **Codes\\Personal history\Level of education** | | | | | | | | | | | | | |  |
|  |  |  |  |  | No |  | 0.0228 |  | 1 |  | | | | | | |  |
|  | | |  |  |  |  |  |  |  |  | | | | | | | |
|  | | | | | | | | | | | | 1 |  | MTK |  | 6/30/2021 8:13 PM |  |
|  | | | On the side of education, how far did you go  P: I reached up to form two.  I: Indeed some people stop in form two, but for you what made you to stop in form two?  P: Because of school fees problems  I: What happened to school fees problems?  P: The parents could not manage to pay school fees for me.  I: Meaning that you wanted to continue with education but failed due to school fees?  P: Yes I wanted to continue but because I had no school fees, I could not. | | | | | | | | | | | | | |  |
|  | | |  |  |  |  |  |  |  |  |  |  |  |  |  |  |  |
|  | | |  | | | | | | | | | | | | | |  |
|  | | | **Codes\\Personal history\Location** | | | | | | | | | | | | | |  |
|  |  |  |  |  | No |  | 0.0446 |  | 3 |  | | | | | | |  |
|  | | |  |  |  |  |  |  |  |  | | | | | | | |
|  | | | | | | | | | | | | 1 |  | MTK |  | 6/30/2021 8:17 PM |  |
|  | | | I: Where do you stay?  P: I stay at ……… trading centre  I: Is this your home village or a business place?  P: We bought the land and settled there, we are not doing business  I: Who is the village head at ………?  P : ………….  I: Traditional Authority?  P: Kabudula | | | | | | | | | | | | | |  |
|  | | |  |  |  |  |  |  |  |  |  |  |  |  |  |  |  |
|  | | |  | | | | | | | | | | | | | |  |
|  | | | | | | | | | | | | 2 |  | MTK |  | 6/30/2021 8:17 PM |  |
|  | | | I: Where is your home village?  P: My husband is from …..  I: Which T/A?  P: ……….  I: And your home?  P: ……  I: T/A?  P: ……….. | | | | | | | | | | | | | |  |
|  | | |  |  |  |  |  |  |  |  |  |  |  |  |  |  |  |
|  | | |  | | | | | | | | | | | | | |  |
|  | | | | | | | | | | | | 3 |  | MTK |  | 6/30/2021 8:18 PM |  |
|  | | | I: You said you purchased land and settled at ……… as your own village, what made you move from ………….. to stay at ………..?  P: For us to buy land at ………….is because there were a lot of talking (quarrels), so we decided to find our own place so that we can invest for our children.  I: okay thank you very much, before coming to …………… were you living at your home village or your husband village?  P: We were living at my husband’s village | | | | | | | | | | | | | |  |
|  | | |  | | | | | | | | | | | | | |  |
|  | | | **Codes\\Personal history\Marital status** | | | | | | | | | | | | | |  |
|  |  |  |  |  | No |  | 0.0168 |  | 2 |  | | | | | | |  |
|  | | |  |  |  |  |  |  |  |  | | | | | | | |
|  | | | | | | | | | | | | 1 |  | MTK |  | 6/30/2021 8:13 PM |  |
|  | | | I: Okay, thanks, are you married?  P: Yes I am married | | | | | | | | | | | | | |  |
|  | | |  | | | | | | | | | | | | | |  |
| Formatted Reports\\Coding Summary by File Formatted Report | | | | | | | | | | | Page 3 of 53 | | | | | | |
| 7/5/2021 9:43 AM | | | | | | | | | | | | | | | | | |
|  | | | **Classification** |  | **Aggregate** |  | **Coverage** |  | **Number Of Coding References** |  | | **Reference Number** |  | **Coded By Initials** |  | **Modified On** |  |
|  | | | | | | | | | | | | | | | | | |
|  | | | | | | | | | | | | 2 |  | MTK |  | 6/30/2021 8:14 PM |  |
|  | | | I: Can you tell me more about your marriage, is this your first marriage? are you the only wife? Is this your first husband? Are you his first wife?  P: This is my first marriage with my husband.  I: You are also the first wife to your husband?  P: Yes he has never been married before. | | | | | | | | | | | | | |  |
|  | | |  | | | | | | | | | | | | | |  |
|  | | | **Codes\\Personal history\Number of children** | | | | | | | | | | | | | |  |
|  |  |  |  |  | No |  | 0.0354 |  | 2 |  | | | | | | |  |
|  | | |  |  |  |  |  |  |  |  | | | | | | | |
|  | | | | | | | | | | | | 1 |  | MTK |  | 6/30/2021 8:15 PM |  |
|  | | | I: Do you have children in this family?  P: In this family we have been blessed with three children, 2 boys and 1 girl.  I: Do have plans of having some in future?  P: We will see how it will go but as of now they should be three  I :Did you discuss that they should be three ?  P: As of now they should be three we will decide in the future whether to have some since we have not done permanent method of contraceptives/ having children. | | | | | | | | | | | | | |  |
|  | | |  |  |  |  |  |  |  |  |  |  |  |  |  |  |  |
|  | | |  | | | | | | | | | | | | | |  |
|  | | | | | | | | | | | | 2 |  | MTK |  | 6/30/2021 8:16 PM |  |
|  | | | I: so it looks you have not discussed with your husband on the number of children to have, whether three or more because had it been that they should be three you would have done tubaligation.  P: Yes we have not discussed properly. | | | | | | | | | | | | | |  |
|  | | |  | | | | | | | | | | | | | |  |
|  | | | **Codes\\Previous work experience** | | | | | | | | | | | | | |  |
|  |  |  |  |  | No |  | 0.0269 |  | 1 |  | | | | | | |  |
|  | | |  |  |  |  |  |  |  |  | | | | | | | |
|  | | | | | | | | | | | | 1 |  | MTK |  | 6/30/2021 9:10 PM |  |
|  | | | : Thank you, I have known your history and now we are going into the second part where I will ask you questions about the study, I urge you to feel free and participate as you have done in the first part. My first question is that I want to know if you have you ever taken part in helping people as a health volunteer before taking part in this study.  P: I have never taken part in any voluntary work; this is my first time to participate in such a study or being a volunteer  I: So, this is the first time to be in a study as a volunteer  P: Yes | | | | | | | | | | | | | |  |
|  | | |  | | | | | | | | | | | | | |  |
|  | | | **Codes\\Previous work experience\Volunteer experience** | | | | | | | | | | | | | |  |
|  |  |  |  |  | No |  | 0.0269 |  | 1 |  | | | | | | |  |
|  | | |  |  |  |  |  |  |  |  | | | | | | | |
|  | | | | | | | | | | | | 1 |  | MTK |  | 6/30/2021 9:10 PM |  |
|  | | | : Thank you, I have known your history and now we are going into the second part where I will ask you questions about the study, I urge you to feel free and participate as you have done in the first part. My first question is that I want to know if you have you ever taken part in helping people as a health volunteer before taking part in this study.  P: I have never taken part in any voluntary work; this is my first time to participate in such a study or being a volunteer  I: So, this is the first time to be in a study as a volunteer  P: Yes | | | | | | | | | | | | | |  |
|  | | |  | | | | | | | | | | | | | |  |
|  | | | | | | | | | | | | | | | | | |
| Formatted Reports\\Coding Summary by File Formatted Report | | | | | | | | | | | Page 4 of 53 | | | | | | |
| 7/5/2021 9:43 AM | | | | | | | | | | | | | | | | | |
|  | | | **Classification** |  | **Aggregate** |  | **Coverage** |  | **Number Of Coding References** |  | | **Reference Number** |  | **Coded By Initials** |  | **Modified On** |  |
|  | | | **Codes\\Recommendations** | | | | | | | | | | | | | |  |
|  |  |  |  |  | No |  | 0.0499 |  | 2 |  | | | | | | |  |
|  | | |  |  |  |  |  |  |  |  | | | | | | | |
|  | | | | | | | | | | | | 1 |  | MTK |  | 6/30/2021 9:52 PM |  |
|  | | | I: You have told me about your part, what about the study procedures, did you find any part that you feel could have been looked into or changed or improved?  P: This study should be done again so that some things can be changed if we can take the example of our friend who failed, as I have already said the training period was short I was one of people in the group who had problems and could have failed because I was shy but after exposure to the women I am used. So the training needs to be done again. | | | | | | | | | | | | | |  |
|  | | |  | | | | | | | | | | | | | |  |
|  | | | | | | | | | | | | 2 |  | MTK |  | 6/30/2021 9:53 PM |  |
|  | | | I: Apart from the training, is there anything you see that needs to be changed in this research in order to benefit other people in giving the services? What are the things that you want the researchers look into in giving this service  P: Yes  I: Like what?  P: The time we were visiting the women, they should look into the availability of books, for this is research but when the programme get started they should have enough books of health pictures to be left to women so that they can read properly. | | | | | | | | | | | | | |  |
|  | | |  | | | | | | | | | | | | | |  |
|  | | | **Codes\\THPP delivery experience** | | | | | | | | | | | | | |  |
|  |  |  |  |  | No |  | 0.2448 |  | 8 |  | | | | | | |  |
|  | | |  |  |  |  |  |  |  |  | | | | | | | |
|  | | | | | | | | | | | | 1 |  | MTK |  | 6/30/2021 9:19 PM |  |
|  | | | I: I thank you very much, we are continuing with the questions, now regarding the women you were visiting giving them the services at their homes, how can you describe the process, what were your experiences in reaching the women in their homes.  P: There were no problems for me only that I wish there was some assistance given to the women like the other time they started giving us soap to give the women, one time when I went to visit the woman and gave her soap the next visit after the soap the women welcomed us cheerfully. Maybe they had hope of getting assistance again from the study through sessions as well as other material support like soap, otherwise there were no problems for me but I wish there was some material support to the women. | | | | | | | | | | | | | |  |
|  | | |  | | | | | | | | | | | | | |  |
|  | | | | | | | | | | | | 2 |  | MTK |  | 6/30/2021 9:29 PM |  |
|  | | | I: How many women did you visit?  P: I visited two women  I: Did you manage to involve their husbands for both women, was it possible or not?  P: Yes I managed to involve their husbands but it was not all the days.  I: Why not all the days?  P: The first woman her husband goes for piece jobs so other days he would ask permission from the boss to be excused and meet me for the sessions, sometimes he was unable to be excused. The second woman it was also the same I met the man in some days not every visit. Other men thought this was for women only and they would say, chart with my wife she will tell me. | | | | | | | | | | | | | |  |
|  | | |  |  |  |  |  |  |  |  |  |  |  |  |  |  |  |
|  | | |  | | | | | | | | | | | | | |  |
|  | | | | | | | | | | | | 3 |  | MTK |  | 6/30/2021 9:31 PM |  |
|  | | | I: So, the men were thinking that these sessions were for women only, or did the men really understood the reason of your coming in their homes?  P: The men were well briefed firstly by the woman who met the doctors at the hospital and was asked to tell the husband before the programme started/home visits started. During the introductory session, we explained that we are village counsellors who will be visiting the women and the men accepted being briefed by the women and during the visits they were involved in the programme, this was done during the time we introduced the programme. | | | | | | | | | | | | | |  |
|  | | |  | | | | | | | | | | | | | |  |
|  | | | | | | | | | | | | 4 |  | MTK |  | 6/30/2021 9:33 PM |  |
|  | | | I: Going further with the questions , my next question is tell me about this woman you followed at her home ,what interest did she had apart from you getting to her house to deliver the counselling session  P: These women were showing interest because there were given tasks like pictures about nutrition, rest and they were able to mark correctly once they complete the task and they showed interest in the lessons | | | | | | | | | | | | | |  |
|  | | |  | | | | | | | | | | | | | |  |
|  | | | | | | | | | | | | | | | | | |
| Formatted Reports\\Coding Summary by File Formatted Report | | | | | | | | | | | Page 5 of 53 | | | | | | |
| 7/5/2021 9:43 AM | | | | | | | | | | | | | | | | | |
|  | | | **Classification** |  | **Aggregate** |  | **Coverage** |  | **Number Of Coding References** |  | | **Reference Number** |  | **Coded By Initials** |  | **Modified On** |  |
|  | | | | | | | | | | | | | | | | | |
|  | | | | | | | | | | | | 5 |  | MTK |  | 6/30/2021 9:34 PM |  |
|  | | | I: So when you make arrangements to meet, were the women were found at the agreed place and time. Were you able to meet as planned and do what you planned?  P: Yes, When we agree on the day and time it was my responsibility as a counsellor to keep time, because the woman was already at her house and I was the one visiting her then I found the woman ready waiting for me and we could start our programme.  I: So you kept time, what about the woman did she keep time or maybe she forgot about the meeting or was busy with other things, were they ready for meetings?  P: All the women I visited kept time as agreed, I always found them ready for me. | | | | | | | | | | | | | |  |
|  | | |  |  |  |  |  |  |  |  |  |  |  |  |  |  |  |
|  | | |  | | | | | | | | | | | | | |  |
|  | | | | | | | | | | | | 6 |  | MTK |  | 6/30/2021 9:48 PM |  |
|  | | | I: I have three questions to ask you in our last part, the first question is what are your experience in giving mental health counselling? What were your experience when delivering this service in summary?  P: At the time we were providing service to these women may be I can say that there were a problem or not like we were being asked “where are you going” and the response is that I am going to visit some pregnant woman who sometimes is stressed and they would further ask what do we give them when visited and the response is that we do not give anything because these women were already told that we will not give them any material thing, no medication nor money but counselling sessions to solve their problems. | | | | | | | | | | | | | |  |
|  | | |  | | | | | | | | | | | | | |  |
|  | | | | | | | | | | | | 7 |  | MTK |  | 6/30/2021 9:50 PM |  |
|  | | | I: I am saying that when you were providing the service, which areas do you see that went well or did not go well when you were delivering the intervention?  P: I was visiting two women and, one woman had difficulties, I tried delivering the service, she was being assisted but because she was staying at an estate she had some some problems that peristed relating to the boss,we tried to discuss she improved but not fully but the other woman had no difficulties and the time she was tested everything was normal.  I: What was the problem?  P: The problems with her husband were lessened but with the bosses they were some issues which made her think of going back home to ease the stress that came with the maltreatment from the estate bosses. | | | | | | | | | | | | | |  |
|  | | |  |  |  |  |  |  |  |  |  |  |  |  |  |  |  |
|  | | |  | | | | | | | | | | | | | |  |
|  | | | | | | | | | | | | 8 |  | MTK |  | 6/30/2021 9:52 PM |  |
|  | | | I: I want you to evaluate yourself as a person who gave the services to the women because you were going alone, can you explain which part gave you problems when providing counselling if there were any or you see that you did not have much ability on other issues, is there any weakness if you can evaluate yourself?  P: That part is not there  I: So we can say that you tried on your side?  P: As for me I really tried on my part | | | | | | | | | | | | | |  |
|  | | |  | | | | | | | | | | | | | |  |
|  | | | **Codes\\THPP delivery experience\Elements** | | | | | | | | | | | | | |  |
|  |  |  |  |  | No |  | 0.0142 |  | 1 |  | | | | | | |  |
|  | | |  |  |  |  |  |  |  |  | | | | | | | |
|  | | | | | | | | | | | | 1 |  | MTK |  | 6/30/2021 9:15 PM |  |
|  | | | I: You are telling me a very important point that during the time you were visiting women to be helped you also benefited, not so?  P: Yes I also benefited, had it been the study was there the time I was pregnant I could have benefited the same way but the programme found me not pregnant. | | | | | | | | | | | | | |  |
|  | | |  | | | | | | | | | | | | | |  |
|  | | | **Codes\\THPP delivery experience\Elements\Barriers** | | | | | | | | | | | | | |  |
|  |  |  |  |  | No |  | 0.0670 |  | 3 |  | | | | | | |  |
|  | | |  |  |  |  |  |  |  |  | | | | | | | |
|  | | | | | | | | | | | | 1 |  | MTK |  | 6/30/2021 9:25 PM |  |
|  | | | I: Thank you, before I proceed further, I would like to know if you as the one giving the intervention had anything that you lacked or everything was, okay?  P: The problem we had was transport because we were using a hired bicycle, the owner did not allow to give the service on credit and sometimes I could sell my things to have money for transport for others refused to offer transport services on credit. We were given transport money after each visit.  I: Apart from transport was there any problems?  P: There was no any problem | | | | | | | | | | | | | |  |
|  | | |  | | | | | | | | | | | | | |  |
| Formatted Reports\\Coding Summary by File Formatted Report | | | | | | | | | | | Page 6 of 53 | | | | | | |
| 7/5/2021 9:43 AM | | | | | | | | | | | | | | | | | |
|  | | | **Classification** |  | **Aggregate** |  | **Coverage** |  | **Number Of Coding References** |  | | **Reference Number** |  | **Coded By Initials** |  | **Modified On** |  |
|  | | | | | | | | | | | | | | | | | |
|  | | | | | | | | | | | | 2 |  | MTK |  | 6/30/2021 9:30 PM |  |
|  | | | I: So it seems the other reason men were not found at home is because were in search for resources for the homes?  P: Yes, most men were out working/farming to provide for their families. Because during the first time when we talked about woman’s health, we stressed the need for women to be supported to find resources and food to maintain their health. So, others could say chart with my wife as I go and search for money to help in providing for my wife and family so they can be healthy. | | | | | | | | | | | | | |  |
|  | | |  | | | | | | | | | | | | | |  |
|  | | | | | | | | | | | | 3 |  | MTK |  | 6/30/2021 9:34 PM |  |
|  | | | I: okay you said you gave them assignments, I would like to know, were they able to do the work you gave them.  P: The work was just shown to them because of shortage of books, some wanted to have books left to them so that they can mark the assignments together with the husband, so books were not left but I could show them the tasks and also what will be discussed in the next session. | | | | | | | | | | | | | |  |
|  | | |  | | | | | | | | | | | | | |  |
|  | | | **Codes\\THPP delivery experience\Elements\Difficult to deliver** | | | | | | | | | | | | | |  |
|  |  |  |  |  | No |  | 0.0317 |  | 3 |  | | | | | | |  |
|  | | |  |  |  |  |  |  |  |  | | | | | | | |
|  | | | | | | | | | | | | 1 |  | MTK |  | 6/30/2021 9:35 PM |  |
|  | | | I: Meaning that you could leave the work but was difficult for them to work on the tasks because they had no books  P: Yes because during the training they said every counsellor should have three books and was visiting three women, so because of books a counsellor was forced to mark the woman’s work the same day but during the training we were told to leave the books but this was not done because we had one book. | | | | | | | | | | | | | |  |
|  | | |  | | | | | | | | | | | | | |  |
|  | | | | | | | | | | | | 2 |  | MTK |  | 6/30/2021 9:35 PM |  |
|  | | | I: So you are telling me the other problem was the shortage of books in addition to what you told me earlier?  P: Yes, the books were few. | | | | | | | | | | | | | |  |
|  | | |  | | | | | | | | | | | | | |  |
|  | | | | | | | | | | | | 3 |  | MTK |  | 6/30/2021 9:49 PM |  |
|  | | | I: Did you see anything that you can say made counselling difficult?  P: There is nothing | | | | | | | | | | | | | |  |
|  | | |  | | | | | | | | | | | | | |  |
|  | | | **Codes\\THPP delivery experience\Elements\Easy to deliver** | | | | | | | | | | | | | |  |
|  |  |  |  |  | No |  | 0.0595 |  | 2 |  | | | | | | |  |
|  | | |  |  |  |  |  |  |  |  | | | | | | | |
|  | | | | | | | | | | | | 1 |  | MTK |  | 6/30/2021 9:26 PM |  |
|  | | | I: Thank you, now I would like know what happened when you reach a woman’s home, how were you welcomed and were other people present like husband, mother-in laws, did you had chance of talking to these people, involving them and hear their problems?  P: When we visited the woman for the first time/the introductory session we invited the husband, mother-in law, or the family member who she trusts to take part in the programme and on other days I could find either the husband or the mother in-law depending on whoever was free. Because when we were introducing the programme we explained everything, what the programme is about and that we are counselors that will be helping the woman and the family deal with issues causing stress and that the counselling is confidential. | | | | | | | | | | | | | |  |
|  | | |  | | | | | | | | | | | | | |  |
|  | | | | | | | | | | | | 2 |  | MTK |  | 6/30/2021 9:49 PM |  |
|  | | | I: What parts were easy for you to do/teach when doing counselling sessions?  P: I would like the giving of soap to continue  I: I meant in counselling which were the easy areas in counselling  P: The work itself was easy because everything was in the books and we were taught during the training and the first times we were reading each page but later stopped using a book because I was used and memorized the content by heart. | | | | | | | | | | | | | |  |
|  | | |  | | | | | | | | | | | | | |  |
|  | | | | | | | | | | | | | | | | | |
| Formatted Reports\\Coding Summary by File Formatted Report | | | | | | | | | | | Page 7 of 53 | | | | | | |
| 7/5/2021 9:43 AM | | | | | | | | | | | | | | | | | |
|  | | | **Classification** |  | **Aggregate** |  | **Coverage** |  | **Number Of Coding References** |  | | **Reference Number** |  | **Coded By Initials** |  | **Modified On** |  |
|  | | | **Codes\\THPP delivery experience\Elements\Facilitators** | | | | | | | | | | | | | |  |
|  |  |  |  |  | No |  | 0.0726 |  | 3 |  | | | | | | |  |
|  | | |  |  |  |  |  |  |  |  | | | | | | | |
|  | | | | | | | | | | | | 1 |  | MTK |  | 6/30/2021 9:20 PM |  |
|  | | | I: I thank you very much, we are continuing with the questions, now regarding the women you were visiting giving them the services at their homes, how can you describe the process, what were your experiences in reaching the women in their homes.  P: There were no problems for me only that I wish there was some assistance given to the women like the other time they started giving us soap to give the women, one time when I went to visit the woman and gave her soap the next visit after the soap the women welcomed us cheerfully. Maybe they had hope of getting assistance again from the study through sessions as well as other material support like soap, otherwise there were no problems for me but I wish there was some material support to the women. | | | | | | | | | | | | | |  |
|  | | |  | | | | | | | | | | | | | |  |
|  | | | | | | | | | | | | 2 |  | MTK |  | 6/30/2021 9:20 PM |  |
|  | | | I: So you are saying that when you were given soap to deliver to the women, the next visit you were welcomed more cheerfully?  P: Yes they thought they are going to be helped again this time because we were visiting women with a lot of problems in the villages, there are a lot of problems, I mean real problems. | | | | | | | | | | | | | |  |
|  | | |  | | | | | | | | | | | | | |  |
|  | | | | | | | | | | | | 3 |  | MTK |  | 6/30/2021 9:21 PM |  |
|  | | | I: Like what can you explain?  P: There are problems in the villages, financial problems, sometimes women were having stress and some unhelpful thoughts due to poverty, they would be sad and sometimes they would tell us the problems they face like husbands not providing them with household needs like soap. So, once we started giving them soap, we saw that the women were freer to talk about their issues. | | | | | | | | | | | | | |  |
|  | | |  | | | | | | | | | | | | | |  |
|  | | | **Codes\\THPP training\Content** | | | | | | | | | | | | | |  |
|  |  |  |  |  | No |  | 0.0279 |  | 1 |  | | | | | | |  |
|  | | |  |  |  |  |  |  |  |  | | | | | | | |
|  | | | | | | | | | | | | 1 |  | MTK |  | 6/30/2021 9:13 PM |  |
|  | | | I: My next question is about the women whom you were visiting, did you had adequate training before starting so that when going you would do your work perfectly?  P: Yes we had proper training, it went well but I feel the duration was short. I started understanding the issues very well/getting the issues once we started visiting these women whilst in the study with the help of reference manuals. The other thing is that we had no books the first days of the training to help us refer to what was being told, the books came later on the second day. | | | | | | | | | | | | | |  |
|  | | |  | | | | | | | | | | | | | |  |
|  | | | **Codes\\THPP training\Duration** | | | | | | | | | | | | | |  |
|  |  |  |  |  | No |  | 0.0434 |  | 2 |  | | | | | | |  |
|  | | |  |  |  |  |  |  |  |  | | | | | | | |
|  | | | | | | | | | | | | 1 |  | MTK |  | 6/30/2021 9:13 PM |  |
|  | | | I: You said the days for the training were inadequate, how many days did you do the training?  P: 5 days  I: How many days could have considered enough for the training?  P: 7 or 8 days, we would have mastered everything, because the time we were on training and doing role plays it was hard for some of us to get everything that time but we got used when we started visiting the women it is when I could recall what was learnt and what we were doing during the training by reading the books. | | | | | | | | | | | | | |  |
|  | | |  | | | | | | | | | | | | | |  |
|  | | | | | | | | | | | | 2 |  | MTK |  | 6/30/2021 9:53 PM |  |
|  | | | I: So what you are saying right away from the beginning is the duration of the training?  P: Because the time we started the training they told us that it was not hard and they told us not to write for they will provide us with books but we were not given the books in time. And now we are used, had it been we were given the books at the beginning things would not have been difficult. | | | | | | | | | | | | | |  |
|  | | |  | | | | | | | | | | | | | |  |
|  | | | | | | | | | | | | | | | | | |
| Formatted Reports\\Coding Summary by File Formatted Report | | | | | | | | | | | Page 8 of 53 | | | | | | |
| 7/5/2021 9:43 AM | | | | | | | | | | | | | | | | | |
|  | | | **Classification** |  | **Aggregate** |  | **Coverage** |  | **Number Of Coding References** |  | | **Reference Number** |  | **Coded By Initials** |  | **Modified On** |  |
|  | | | **Codes\\THPP training\Preparedness** | | | | | | | | | | | | | |  |
|  |  |  |  |  | No |  | 0.0452 |  | 2 |  | | | | | | |  |
|  | | |  |  |  |  |  |  |  |  | | | | | | | |
|  | | | | | | | | | | | | 1 |  | MTK |  | 6/30/2021 9:14 PM |  |
|  | | | I: How many days could have considered enough for the training?  P: 7 or 8 days, we would have mastered everything, because the time we were on training and doing role plays it was hard for some of us to get everything that time but we got used when we started visiting the women it is when I could recall what was learnt and what we were doing during the training by reading the books. | | | | | | | | | | | | | |  |
|  | | |  | | | | | | | | | | | | | |  |
|  | | | | | | | | | | | | 2 |  | MTK |  | 6/30/2021 9:14 PM |  |
|  | | | I: okay although you have said that the training was inadequate, do you think it was good for you to be trained before going to work with the women in the village?  P: Yes the training was needed.  I: How useful was it?  P: The training was helpful when visiting the woman because though I was one of those giving help to the women I also benefited because I had the same problems when we had training and was visiting the women had it been that I was also pregnant I could have been help myself with the information I gained. | | | | | | | | | | | | | |  |
|  | | |  |  |  |  |  |  |  |  |  |  |  |  |  |  |  |
|  | | |  | | | | | | | | | | | | | |  |
|  | **Files\\THPP PV_#04** | | | | | | | | | | | | | | | |  |
|  | | **Code** | | | | | | | | | | | | | | |  |
|  | | | **Codes\\Health workers\Relationship** | | | | | | | | | | | | | |  |
|  |  |  |  |  | No |  | 0.0546 |  | 1 |  | | | | | | |  |
|  | | |  |  |  |  |  |  |  |  | | | | | | | |
|  | | | | | | | | | | | | 1 |  | MTK |  | 7/2/2021 9:35 AM |  |
|  | | | I: Moving forward, I understand that you were working with the women as well as the health workers…  R: True.  I: How have you worked with the health workers? What were the challenges you faced working with them and what were the successes?  R: There was a very good relationship between us and the doctors. You know it is not easy to feel free with people you have met for the first time but it was easy for us to be free with them because they were free. There were no major challenges. The only challenge was that they were changing somethings in the programme at short notice. For instance, the agreement was that we were going to make ten follow up sessions with the mothers and four of them were to be done while she was pregnant and six of them after delivery. It was after we had started delivering the counseling intervention when they started changing things. For example if you were to follow up the woman in ten visits, only eight follow up visits were done, they told us to terminate the sessions at visit number 8 following screening of the women. | | | | | | | | | | | | | |  |
|  | | |  |  |  |  |  |  |  |  |  |  |  |  |  |  |  |
|  | | |  | | | | | | | | | | | | | |  |
|  | | | **Codes\\Involvement of family members** | | | | | | | | | | | | | |  |
|  |  |  |  |  | No |  | 0.0679 |  | 3 |  | | | | | | |  |
|  | | |  |  |  |  |  |  |  |  | | | | | | | |
|  | | | | | | | | | | | | 1 |  | MTK |  | 7/2/2021 9:28 AM |  |
|  | | | I: So you have said that when you went to the woman’s home you were also involving other people around the woman such as mother in-law, sister in-law if she was at her partner’s home or her mother and relatives if she was at her home. How was the interaction during the counseling session with the other family members?  R: The interaction was very good and successful. The families I was visiting appreciated the program and most were saying it’s unfortunate that the programme is starting now as they are having the last child etc. Most families thought the programme was useful and there was no family that thought it was not useful, they accepted this programme and we have seen changes in families after going through the sessions. The most interesting part is that they all agreed that it is important that there should be good relationship between the mother and people surrounding her to help her live a healthy life. The relatives were able to support the woman in various aspects after the counseling sessions. I have an experience of one woman whose partner was not assisting the woman, but after participating in the sessions, I found him washing during the next visit. | | | | | | | | | | | | | |  |
|  | | |  |  |  |  |  |  |  |  |  |  |  |  |  |  |  |
|  | | |  | | | | | | | | | | | | | |  |
| Formatted Reports\\Coding Summary by File Formatted Report | | | | | | | | | | | Page 9 of 53 | | | | | | |
| 7/5/2021 9:43 AM | | | | | | | | | | | | | | | | | |
|  | | | **Classification** |  | **Aggregate** |  | **Coverage** |  | **Number Of Coding References** |  | | **Reference Number** |  | **Coded By Initials** |  | **Modified On** |  |
|  | | | | | | | | | | | | | | | | | |
|  | | | | | | | | | | | | 2 |  | MTK |  | 7/2/2021 9:29 AM |  |
|  | | | I: So you found the partner washing…?  R: Surely he was washing. | | | | | | | | | | | | | |  |
|  | | |  | | | | | | | | | | | | | |  |
|  | | | | | | | | | | | | 3 |  | MTK |  | 7/2/2021 9:29 AM |  |
|  | | | I: After you had conducted 2 sessions…  R: After the second session when he was involved…  I: And you think it was because you had involved him during the counseling session…  R: Yes he had bought fruits for the woman and he was washing clothes. The woman was very happy and said that “your coming has really made my home to improve. What is happening now has never happened before.” | | | | | | | | | | | | | |  |
|  | | |  | | | | | | | | | | | | | |  |
|  | | | **Codes\\Personal history\Age** | | | | | | | | | | | | | |  |
|  |  |  |  |  | No |  | 0.0309 |  | 1 |  | | | | | | |  |
|  | | |  |  |  |  |  |  |  |  | | | | | | | |
|  | | | | | | | | | | | | 1 |  | MTK |  | 7/2/2021 9:17 AM |  |
|  | | | I: So feel free because this is a confidential discussion and the information that we collect in this study will be used for study purposes only. So feel free to answer any questions that we want to have information from you. This afternoon, you are with me ………. for the Principal Investigator of this study. In our discussion today, we are going to cover three sections. The first part we want to know about your demographic information, the other parts are about the study in which you have been taking part. Firstly, how old are you? You may tell me your birth date, month year if that is possible.  R: I was born on 25th December 1980.  I: Thanks on Christmas day?  R: Sure.  I: What is your age now?  R: I am 40 years old. | | | | | | | | | | | | | |  |
|  | | |  |  |  |  |  |  |  |  |  |  |  |  |  |  |  |
|  | | |  | | | | | | | | | | | | | |  |
|  | | | **Codes\\Personal history\Contact information** | | | | | | | | | | | | | |  |
|  |  |  |  |  | No |  | 0.0066 |  | 1 |  | | | | | | |  |
|  | | |  |  |  |  |  |  |  |  | | | | | | | |
|  | | | | | | | | | | | | 1 |  | MTK |  | 7/2/2021 9:19 AM |  |
|  | | | I: Do you have a mail address?  R: ………………………………………………. Lilongwe.  I: Do you have a phone and what is your number?  R: I have it. | | | | | | | | | | | | | |  |
|  | | |  | | | | | | | | | | | | | |  |
|  | | | **Codes\\Personal history\Level of education** | | | | | | | | | | | | | |  |
|  |  |  |  |  | No |  | 0.0083 |  | 1 |  | | | | | | |  |
|  | | |  |  |  |  |  |  |  |  | | | | | | | |
|  | | | | | | | | | | | | 1 |  | MTK |  | 7/2/2021 9:17 AM |  |
|  | | | I: How far did you go with your education?  R: I did my primary school at ……….. my secondary school at ………….. form 1 up to form 4. I did not do well in form 4 so I use JCE certificate | | | | | | | | | | | | | |  |
|  | | |  | | | | | | | | | | | | | |  |
|  | | | | | | | | | | | | | | | | | |
| Formatted Reports\\Coding Summary by File Formatted Report | | | | | | | | | | | Page 10 of 53 | | | | | | |
| 7/5/2021 9:43 AM | | | | | | | | | | | | | | | | | |
|  | | | **Classification** |  | **Aggregate** |  | **Coverage** |  | **Number Of Coding References** |  | | **Reference Number** |  | **Coded By Initials** |  | **Modified On** |  |
|  | | | **Codes\\Personal history\Location** | | | | | | | | | | | | | |  |
|  |  |  |  |  | No |  | 0.0099 |  | 1 |  | | | | | | |  |
|  | | |  |  |  |  |  |  |  |  | | | | | | | |
|  | | | | | | | | | | | | 1 |  | MTK |  | 7/2/2021 9:19 AM |  |
|  | | | I: Thank you very much. Where are you currently staying?  R: I live at …………………….. and that is my partner’s home but my home district is ………………….  I: Thank you very much. in which TA?  R: TA Kabudula. | | | | | | | | | | | | | |  |
|  | | |  | | | | | | | | | | | | | |  |
|  | | | **Codes\\Personal history\Marital status** | | | | | | | | | | | | | |  |
|  |  |  |  |  | No |  | 0.0029 |  | 1 |  | | | | | | |  |
|  | | |  |  |  |  |  |  |  |  | | | | | | | |
|  | | | | | | | | | | | | 1 |  | MTK |  | 7/2/2021 9:18 AM |  |
|  | | | I: Are you married?  R: I am married with six children; 4 girls 2 boys. | | | | | | | | | | | | | |  |
|  | | |  | | | | | | | | | | | | | |  |
|  | | | **Codes\\Personal history\Number of children** | | | | | | | | | | | | | |  |
|  |  |  |  |  | No |  | 0.0029 |  | 1 |  | | | | | | |  |
|  | | |  |  |  |  |  |  |  |  | | | | | | | |
|  | | | | | | | | | | | | 1 |  | MTK |  | 7/2/2021 9:18 AM |  |
|  | | | I: Are you married?  R: I am married with six children; 4 girls 2 boys. | | | | | | | | | | | | | |  |
|  | | |  | | | | | | | | | | | | | |  |
|  | | | **Codes\\Previous work experience\Volunteer experience** | | | | | | | | | | | | | |  |
|  |  |  |  |  | No |  | 0.0343 |  | 1 |  | | | | | | |  |
|  | | |  |  |  |  |  |  |  |  | | | | | | | |
|  | | | | | | | | | | | | 1 |  | MTK |  | 7/2/2021 9:20 AM |  |
|  | | | I: What were the good things and challenges of this volunteer work?  R: The good thing was that as a volunteer, I had the opportunity to learn many things because I was also able to benefit from the program to enhance nutrition for my children because I also have children. The challenge was that usually people in the communities have difficulties in understanding some concepts. It was happening that when we were promoting eating of six food groups, most of them were saying that they cannot manage the food because they have no money but overtime, they realized that the foods are locally available in the communities and a lot of them were able to provide six food groups to their families. We were encouraging them to use locally available foods in the communities because they cannot manage to buy some of the foods. | | | | | | | | | | | | | |  |
|  | | |  |  |  |  |  |  |  |  |  |  |  |  |  |  |  |
|  | | |  | | | | | | | | | | | | | |  |
|  | | | **Codes\\Previous work experience\Volunteer experience\Activities** | | | | | | | | | | | | | |  |
|  |  |  |  |  | No |  | 0.0139 |  | 1 |  | | | | | | |  |
|  | | |  |  |  |  |  |  |  |  | | | | | | | |
|  | | | | | | | | | | | | 1 |  | MTK |  | 7/2/2021 9:20 AM |  |
|  | | | I: What were you doing there?  R: I was working with lactating mothers of children not older than five years to promote nutrition among children because it showed that in our area of TA Kabudula there were more malnourished children. So World relief was promoting Early Childhood Development by enhancing elimination of malnutrition. | | | | | | | | | | | | | |  |
|  | | |  | | | | | | | | | | | | | |  |
| Formatted Reports\\Coding Summary by File Formatted Report | | | | | | | | | | | Page 11 of 53 | | | | | | |
| 7/5/2021 9:43 AM | | | | | | | | | | | | | | | | | |
|  | | | **Classification** |  | **Aggregate** |  | **Coverage** |  | **Number Of Coding References** |  | | **Reference Number** |  | **Coded By Initials** |  | **Modified On** |  |
|  | | | **Codes\\Previous work experience\Volunteer experience\Length of volunteer experience** | | | | | | | | | | | | | |  |
|  |  |  |  |  | No |  | 0.0047 |  | 1 |  | | | | | | |  |
|  | | |  |  |  |  |  |  |  |  | | | | | | | |
|  | | | | | | | | | | | | 1 |  | MTK |  | 7/2/2021 9:21 AM |  |
|  | | | I: Did you stop working on this?  R: No I am still working.  I: Which organization did you say…?  R: World Relief. | | | | | | | | | | | | | |  |
|  | | |  | | | | | | | | | | | | | |  |
|  | | | **Codes\\Previous work experience\Volunteer experience\Name of organization** | | | | | | | | | | | | | |  |
|  |  |  |  |  | No |  | 0.0169 |  | 2 |  | | | | | | |  |
|  | | |  |  |  |  |  |  |  |  | | | | | | | |
|  | | | | | | | | | | | | 1 |  | MTK |  | 7/2/2021 9:20 AM |  |
|  | | | I: Thank you very much. That’s it on your demographics. Now I want us to go into details of the study in which you were taking part but before that, have you ever been a health volunteer in health related field?  R: I was a volunteer working as a promoter of nutrition for World Relief. Sure. | | | | | | | | | | | | | |  |
|  | | |  | | | | | | | | | | | | | |  |
|  | | | | | | | | | | | | 2 |  | MTK |  | 7/2/2021 9:21 AM |  |
|  | | | I: Did you stop working on this?  R: No I am still working.  I: Which organization did you say…?  R: World Relief. | | | | | | | | | | | | | |  |
|  | | |  | | | | | | | | | | | | | |  |
|  | | | **Codes\\Recommendations** | | | | | | | | | | | | | |  |
|  |  |  |  |  | No |  | 0.2463 |  | 4 |  | | | | | | |  |
|  | | |  |  |  |  |  |  |  |  | | | | | | | |
|  | | | | | | | | | | | | 1 |  | MTK |  | 7/2/2021 9:42 AM |  |
|  | | | I: Thank you very much. So where do you think the intervention needs improvement in general besides what you already mentioned?  R: Firstly, I would like if there was specific area that a counselor can be responsible for in this intervention, a catchment area. Just as the Health Surveillance Assistants work to deliver under five services in their respective catchment areas we also need to be given a catchment area where we can be making follow ups on the pregnant women. Secondly as I said earlier on this programme seem to be curative, we were delivering it to women who had depression already, but when you look at it closely it can help to prevent depression, so if a volunteer is given a catchment area, then they can be able to find pregnant women in their catchment area and start delivering the intervention to prevent depression so that the women do not get to the point of getting sick with depression. That is my opinion.  I: What do you think are the areas of success in this study and which ones are the challenges?  R: The successes are that we have managed to help the women have healthy feelings, thoughts and behavior as opposed to their previous experiences where they were not able to deal with their depression. There were some women who were not able to sleep and maybe they were just crying but after the counseling, they live happy and healthy lives and when we meet they say “You helped me a lot to live a positive life because in the past, I did not feel good about myself and sometimes I could just be crying or I could think about eliminating my life through hanging. But counseling helped me to have self-confidence and I regret that this program came late”. The intervention has helped women change their way of thinking. This is what went very well because lives of people have been saved, lives of mothers and babies. | | | | | | | | | | | | | |  |
|  | | |  |  |  |  |  |  |  |  |  |  |  |  |  |  |  |
|  | | |  |  |  |  |  |  |  |  |  |  |  |  |  |  |  |
|  | | |  | | | | | | | | | | | | | |  |
|  | | | | | | | | | | | | 2 |  | MTK |  | 7/2/2021 9:43 AM |  |
|  | | | I: Okay. Thank you. Now I would like us to touch base on some of the things that you have already tackled that need improvement. For example, if I remember very well, you mentioned that the training period was short… how would you like it to be done?  R: I would like it to be extended by one week or more so that we should feel confident enough to deliver the intervention because what happened was that even the EPDS, the women were screened when we were not there but then we saw the tool towards termination of the counseling intervention sessions. They were doing the second screening using our phones and we had to tell the participant some of the aspects of the EPDS but we did not have enough knowledge. We would have wished to be trained in EPDS so that we can be able to know that a woman is depressed or not because this counseling has not stopped with this project. After this study we are going to continue with counseling because some women have already started approaching us and we cannot just leave them like that. We provide the counseling.  I: So you would like to be trained in conducting the screening with EPDS…So you want EPDS to be included in the training agenda?  R: Yes it should be included so that we should be able to screen any pregnant woman in her community so that we cannot only concentrate on providing counseling treatment but we should also be able to prevent it. Before the woman start showing signs of depression, we should counsel them. | | | | | | | | | | | | | |  |
|  | | |  |  |  |  |  |  |  |  |  |  |  |  |  |  |  |
|  | | |  | | | | | | | | | | | | | |  |
| Formatted Reports\\Coding Summary by File Formatted Report | | | | | | | | | | | Page 12 of 53 | | | | | | |
| 7/5/2021 9:43 AM | | | | | | | | | | | | | | | | | |
|  | | | **Classification** |  | **Aggregate** |  | **Coverage** |  | **Number Of Coding References** |  | | **Reference Number** |  | **Coded By Initials** |  | **Modified On** |  |
|  | | | | | | | | | | | | | | | | | |
|  | | | | | | | | | | | | 3 |  | MTK |  | 7/2/2021 9:44 AM |  |
|  | | | I: If I understand very well, you mentioned about gadgets… such as phone. Can you tell me more?  R: I said that we need phones that can be able to audio record the counseling session so that we can be able to improve our counseling approach after reviewing our recorded sessions.  I: You also said that you need to approach the women before they are depressed… can you tell me more?  R: I said that prevention is better than cure. Much as counseling is part of treatment, it would also be helpful to prevent the problems that pregnant women go through. This will minimize cases of depression up to the extent of wanting to kill themselves because some of the women we were counseling had suicidal ideations and we need to prevent this by providing counseling before the women reach that extent and that was why I said that counselors need to be given a catchment area to assess every woman who may require counseling. | | | | | | | | | | | | | |  |
|  | | |  |  |  |  |  |  |  |  |  |  |  |  |  |  |  |
|  | | |  | | | | | | | | | | | | | |  |
|  | | | | | | | | | | | | 4 |  | MTK |  | 7/2/2021 9:46 AM |  |
|  | | | I: Thank you very much. What I know is that every study has its procedures but your views are very helpful because they will advise on future intervention counseling sessions. This marks the end of the interview but before we close, do you have final comments?  R: Mine is appreciation for this program and comment to the PI, this programme is very good and helpful. It is unfortunate that it found me after I have stopped bearing children. I have learnt a lot through this programme. I can say that had it been that I was still bearing children, especially my third pregnancy, I would have been one of the participants. I want to encourage researchers, that once they complete their study, they need to roll out this programme, it should not just die down, because the programme has been well received and appreciated in the community, it is very helpful, a lot of women are willing to be part of it because it has improved lives of pregnant women. Because if we just leave the pregnant women, they way they are, we might lose two lives. So once the study is done, I just encourage them to roll it out so that we improve the lives of pregnant women before they are depressed.  I: Thank you very much. So you have also said that the study helped you as a person?  R: Yes.  I: Meaning that it can also assist those people who are not pregnant?  R: Yes, other people may benefit from this intervention because anyone can be depressed at any time, just like I have benefitted. There are people who have approached me that I think can benefit from such an intervention, that’s why I was saying this intervention is not only curative but preventative as well. | | | | | | | | | | | | | |  |
|  | | |  |  |  |  |  |  |  |  |  |  |  |  |  |  |  |
|  | | |  |  |  |  |  |  |  |  |  |  |  |  |  |  |  |
|  | | |  | | | | | | | | | | | | | |  |
|  | | | **Codes\\Session termination\Barriers** | | | | | | | | | | | | | |  |
|  |  |  |  |  | No |  | 0.0350 |  | 1 |  | | | | | | |  |
|  | | |  |  |  |  |  |  |  |  | | | | | | | |
|  | | | | | | | | | | | | 1 |  | MTK |  | 7/2/2021 9:25 AM |  |
|  | | | The other challenge was that during the training, we were told that we would be giving the women soap while they are pregnant and then after delivery when terminating the session. However, we gave the women soap once, and they said they will give them the second batch after group sessions. the other challenge was that we the volunteers, we were traveling long distances to find the participants and that required us to use transport. The money that we were given was not enough for the transport and this money was given to us after we have already conducted the visits. My suggestion is that they should assign us a catchment area whereby we can easily move from one house to the other. Those are some of the challenges we have gone through. | | | | | | | | | | | | | |  |
|  | | |  |  |  |  |  |  |  |  |  |  |  |  |  |  |  |
|  | | |  | | | | | | | | | | | | | |  |
|  | | | **Codes\\THPP delivery experience** | | | | | | | | | | | | | |  |
|  |  |  |  |  | No |  | 0.0504 |  | 1 |  | | | | | | |  |
|  | | |  |  |  |  |  |  |  |  | | | | | | | |
|  | | | | | | | | | | | | 1 |  | MTK |  | 7/2/2021 9:35 AM |  |
|  | | | I: Did you have the same experiences in terms of delivery of the counseling intervention session?  R: No they are different. I had similar experiences with two women and for the other woman I had a different experience. This one seemed to have problems; she did not change much because of the situation she was in.  I: Could this be because she did not understand the intervention?  R: No.  I: What was her problem?  R: I was not supposed to say it here but I will say it for the sake of the interview. This woman had relationship problems, because you can have a session with her today and during the session all would be well, she was able to say that she is feeling much better and is really improving. When I go back next session, because she was in a polygamous relationship you would find that she is back experiencing the same relationship problems she was stressed with. When I went for the last session, I found that her problems were beyond what she was experiencing when she started receiving the counseling intervention. This time, I found that she was no longer at her partner’s house but she went to stay with her parents and she seemed depressed again but at the beginning everything was okay. | | | | | | | | | | | | | |  |
|  | | |  |  |  |  |  |  |  |  |  |  |  |  |  |  |  |
|  | | |  | | | | | | | | | | | | | |  |
| Formatted Reports\\Coding Summary by File Formatted Report | | | | | | | | | | | Page 13 of 53 | | | | | | |
| 7/5/2021 9:43 AM | | | | | | | | | | | | | | | | | |
|  | | | **Classification** |  | **Aggregate** |  | **Coverage** |  | **Number Of Coding References** |  | | **Reference Number** |  | **Coded By Initials** |  | **Modified On** |  |
|  | | | **Codes\\THPP delivery experience\Elements** | | | | | | | | | | | | | |  |
|  |  |  |  |  | No |  | 0.0091 |  | 1 |  | | | | | | |  |
|  | | |  |  |  |  |  |  |  |  | | | | | | | |
|  | | | | | | | | | | | | 1 |  | MTK |  | 7/2/2021 9:26 AM |  |
|  | | | I: So what have been your experiences visiting the mothers in their homes?  R: When we were going to the communities to help pregnant women and lactating mothers on thinking healthy program, the work went on very well. | | | | | | | | | | | | | |  |
|  | | |  | | | | | | | | | | | | | |  |
|  | | | **Codes\\THPP delivery experience\Elements\Barriers** | | | | | | | | | | | | | |  |
|  |  |  |  |  | No |  | 0.0405 |  | 1 |  | | | | | | |  |
|  | | |  |  |  |  |  |  |  |  | | | | | | | |
|  | | | | | | | | | | | | 1 |  | MTK |  | 7/2/2021 9:33 AM |  |
|  | | | I: That is a very good point. Did you have enough resources to conduct the counseling intervention session or there is an area where there can be an improvement?  R: I can say that the resources were not enough. For example, I can say that it was difficult to review the session because after the session, we had nowhere to refer so that we could know if the session went well or not and where it needed improvement. Had it been that we had some gadgets, it would be easier to go through the whole session at home and see where we were supposed to probe or add something and that would help us to improve during the next session.  I: So what gadgets are you referring to?  R: I am referring to phones that can audio record or the audio recorders to help us record the whole session so that we could listen to it all and when the Principal Investigator comes for supervision, she could simply listen to the whole conversation and see where there was need for improvement. | | | | | | | | | | | | | |  |
|  | | |  |  |  |  |  |  |  |  |  |  |  |  |  |  |  |
|  | | |  | | | | | | | | | | | | | |  |
|  | | | **Codes\\THPP delivery experience\Elements\Challenges** | | | | | | | | | | | | | |  |
|  |  |  |  |  | No |  | 0.1473 |  | 3 |  | | | | | | |  |
|  | | |  |  |  |  |  |  |  |  | | | | | | | |
|  | | | | | | | | | | | | 1 |  | MTK |  | 7/2/2021 9:28 AM |  |
|  | | | I: So what have been your experiences visiting the mothers in their homes?  R: When we were going to the communities to help pregnant women and lactating mothers on thinking healthy program, the work went on very well. One thing that was a challenge during the visits was that there were many pregnant and lactating women in the community but it was difficult for us to visit them all because the program focused on the women who were already screened for depression here at the clinic and were found to be depressed. for the rest of the women, it was not possible for us to provide the intervention. But most women once they knew about this programme, the were asking to be involved the programme. the other challenge was that we the volunteers, we were traveling long distances to find the participants and that required us to use transport. The money that we were given was not enough for the transport and this money was given to us after we have already conducted the visits. My suggestion is that they should assign us a catchment area whereby we can easily move from one house to the other. Those are some of the challenges we have gone through.  I: Thank you very much. So besides that, how easy or difficult was it for you to deliver the counseling intervention session to the women?  R: Counseling is not easy. For example in my case, I had difficulties with the first and the second sessions. For the rest of the sessions, it was easy because I was now used and I had formed good relationship with the mothers. The contributing factor to this challenge was that previously, women were visited on HIV and AIDS issues and during the first sessions, it was difficult for the woman to feel free because she was thinking that people will think that she went to the hospital and was found HIV positive. So after I leave, she was thinking that people in the community will start discriminating against her. So, because this programme was good, in that I was also involving the partner, relatives and other family members around her, they were able to understand that my visits were not related to HIV and AIDs but on thinking healthy program whereby I was helping the women in thinking healthy. So that was the challenges I experienced during the first and second sessions but for the third and all the following sessions, it was easy, even the participants started enjoying the intervention because there was nothing bad the community could talk about because the same people around the woman were able to understand the program. | | | | | | | | | | | | | |  |
|  | | |  |  |  |  |  |  |  |  |  |  |  |  |  |  |  |
|  | | |  |  |  |  |  |  |  |  |  |  |  |  |  |  |  |
|  | | |  |  |  |  |  |  |  |  |  |  |  |  |  |  |  |
|  | | |  | | | | | | | | | | | | | |  |
|  | | | | | | | | | | | | 2 |  | MTK |  | 7/2/2021 9:28 AM |  |
|  | | | I: Do you think that was the only challenge or you had challenges during the first and second sessions because you were anxious?  R: Yes I had a little bit of anxiety because it was my first experience but when I got used to it, everything was going on well. | | | | | | | | | | | | | |  |
|  | | |  | | | | | | | | | | | | | |  |
| Formatted Reports\\Coding Summary by File Formatted Report | | | | | | | | | | | Page 14 of 53 | | | | | | |
| 7/5/2021 9:43 AM | | | | | | | | | | | | | | | | | |
|  | | | **Classification** |  | **Aggregate** |  | **Coverage** |  | **Number Of Coding References** |  | | **Reference Number** |  | **Coded By Initials** |  | **Modified On** |  |
|  | | | | | | | | | | | | | | | | | |
|  | | | | | | | | | | | | 3 |  | MTK |  | 7/2/2021 9:42 AM |  |
|  | | | I: What have been the challenges?  R: The challenges are as I said that when I started delivering the intervention, I was not properly following the steps and sometimes I could combine the sessions because I was not used to the counseling content but with time, I became an expert and I was able to help the women change their ways of thinking. | | | | | | | | | | | | | |  |
|  | | |  | | | | | | | | | | | | | |  |
|  | | | **Codes\\THPP delivery experience\Elements\Easy to deliver** | | | | | | | | | | | | | |  |
|  |  |  |  |  | No |  | 0.0257 |  | 1 |  | | | | | | |  |
|  | | |  |  |  |  |  |  |  |  | | | | | | | |
|  | | | | | | | | | | | | 1 |  | MTK |  | 7/2/2021 9:37 AM |  |
|  | | | I: What were the easiest and difficult aspects of the counseling intervention that you were providing? I would like you to do a self-assessment.  R: The aspects that went well, the areas I felt more comfortable with were lessons about one’s feelings, thoughts and behavior. I was feeling good when delivering the intervention in this area because I started realizing that for a behavior to happen, a person has to think about it and there were feelings as well. What I found difficult was when a participant had lots pf problems, it became difficult to move from one step to the other. This took time for me to understand and successfully deliver to the high need participants. | | | | | | | | | | | | | |  |
|  | | |  | | | | | | | | | | | | | |  |
|  | | | **Codes\\THPP delivery experience\Elements\Facilitators** | | | | | | | | | | | | | |  |
|  |  |  |  |  | No |  | 0.1222 |  | 3 |  | | | | | | |  |
|  | | |  |  |  |  |  |  |  |  | | | | | | | |
|  | | | | | | | | | | | | 1 |  | MTK |  | 7/2/2021 9:33 AM |  |
|  | | | I: Sure, thank you very much. So we were looking at relationships but now let us look at the woman you were providing the counseling intervention to… How was the willingness of the women that you were visiting in their homes to participate in the programme?  R: The women showed willingness, they were very welcoming because when we went to them for the first time, we were asking if they were willing to take part in the programme and they said they were willing. So upon agreement, we then discussed the rules/norms that we were going to follow during the programme, we were suggesting some and they were also suggesting others, showing their participation for example they suggested time and days of our meetings. What was happening was that on the set dates despite the fact that we were meeting during the rainy season whereby people were busy working in their gardens, when I arrived at their homes, I was able to find them and they seemed prepared for the session. The other interesting thing was that we were using a health calendar during the counseling intervention and I was giving them assignment to work on to be reviewed during the next visit. I was asking them “from this list, what do you think you can manage?” When I go during the next visit, she was able to say “I managed to do this and this.” This meant that even after the session, they were able to practice what we discussed during the counseling session. | | | | | | | | | | | | | |  |
|  | | |  |  |  |  |  |  |  |  |  |  |  |  |  |  |  |
|  | | |  | | | | | | | | | | | | | |  |
|  | | | | | | | | | | | | 2 |  | MTK |  | 7/2/2021 9:34 AM |  |
|  | | | I: So you have said that they were also suggesting some rules for the discussion. Can you mention some of these rules?  R: On my part, the only additional rule was that the discussion was going to be confidential but for the time and date, they were the ones who proposed. For example, one proposed nine o’clock the other 2 pm and when I went there, I was finding out that they were ready for the session.  I: How many women were you following?  R: Three. | | | | | | | | | | | | | |  |
|  | | |  | | | | | | | | | | | | | |  |
|  | | | | | | | | | | | | 3 |  | MTK |  | 7/2/2021 9:36 AM |  |
|  | | | I: Or I should say what were the barriers and facilitators on your provision of counseling intervention session to the women? How easy or difficult was it?  R: On one part I can say it was easy because it helped me as a person to develop psychologically as much as I was helping the women.  I: Can you clarify?  R: What I am trying to say is that when we are providing counseling, we also learn from the experiences. Only that the project had enrolled specific women, but I would have loved if the programme was delivered to all pregnant women in the community because the issues that were affecting the participants were the same for the other pregnant women in the community. This programme can also help as a preventive measure not just a curative measure because it’s a programme that does not involve giving medication, but counseling from one mother to the other, so am saying it was easier because we were visiting people in our villages, our people, we speak the same language and we were interacting freely, so it wasn’t difficult for us. | | | | | | | | | | | | | |  |
|  | | |  |  |  |  |  |  |  |  |  |  |  |  |  |  |  |
|  | | |  | | | | | | | | | | | | | |  |
| Formatted Reports\\Coding Summary by File Formatted Report | | | | | | | | | | | Page 15 of 53 | | | | | | |
| 7/5/2021 9:43 AM | | | | | | | | | | | | | | | | | |
|  | | | **Classification** |  | **Aggregate** |  | **Coverage** |  | **Number Of Coding References** |  | | **Reference Number** |  | **Coded By Initials** |  | **Modified On** |  |
|  | | | **Codes\\THPP training** | | | | | | | | | | | | | |  |
|  |  |  |  |  | No |  | 0.0350 |  | 1 |  | | | | | | |  |
|  | | |  |  |  |  |  |  |  |  | | | | | | | |
|  | | | | | | | | | | | | 1 |  | MTK |  | 7/2/2021 9:25 AM |  |
|  | | | The other challenge was that we the volunteers, we were traveling long distances to find the participants and that required us to use transport. The money that we were given was not enough for the transport and this money was given to us after we have already conducted the visits. My suggestion is that they should assign us a catchment area whereby we can easily move from one house to the other. Those are some of the challenges we have gone through. | | | | | | | | | | | | | |  |
|  | | |  |  |  |  |  |  |  |  |  |  |  |  |  |  |  |
|  | | |  | | | | | | | | | | | | | |  |
|  | | | **Codes\\THPP training\Content** | | | | | | | | | | | | | |  |
|  |  |  |  |  | No |  | 0.0396 |  | 1 |  | | | | | | |  |
|  | | |  |  |  |  |  |  |  |  | | | | | | | |
|  | | | | | | | | | | | | 1 |  | MTK |  | 7/2/2021 9:22 AM |  |
|  | | | I: World Relief… okay. Now let us focus on the study that you have been taking part as a volunteer counselor. Were you trained?  R: We were really trained although for few days.  I: Can you clarify, did you say the training was not enough?  R: It was not enough training because this is a new concept here in our community but we were only trained for five days. It was essential for us to be properly trained because as volunteers, we were supposed to be equipped with the necessary skills to deliver the intervention and we needed to have the information. On the contrary, we were given a lot of information within a short period of time and we were learning some of the things while the program was being implemented because during the training those areas were not fully covered but had it been that we had more days for training, we would have learnt everything during the training. And we would be having some extra sessions within the programme | | | | | | | | | | | | | |  |
|  | | |  |  |  |  |  |  |  |  |  |  |  |  |  |  |  |
|  | | |  | | | | | | | | | | | | | |  |
|  | | | **Codes\\THPP training\Duration** | | | | | | | | | | | | | |  |
|  |  |  |  |  | No |  | 0.0396 |  | 1 |  | | | | | | |  |
|  | | |  |  |  |  |  |  |  |  | | | | | | | |
|  | | | | | | | | | | | | 1 |  | MTK |  | 7/2/2021 9:22 AM |  |
|  | | | I: World Relief… okay. Now let us focus on the study that you have been taking part as a volunteer counselor. Were you trained?  R: We were really trained although for few days.  I: Can you clarify, did you say the training was not enough?  R: It was not enough training because this is a new concept here in our community but we were only trained for five days. It was essential for us to be properly trained because as volunteers, we were supposed to be equipped with the necessary skills to deliver the intervention and we needed to have the information. On the contrary, we were given a lot of information within a short period of time and we were learning some of the things while the program was being implemented because during the training those areas were not fully covered but had it been that we had more days for training, we would have learnt everything during the training. And we would be having some extra sessions within the programme | | | | | | | | | | | | | |  |
|  | | |  |  |  |  |  |  |  |  |  |  |  |  |  |  |  |
|  | | |  | | | | | | | | | | | | | |  |
|  | | | **Codes\\THPP training\Preparedness** | | | | | | | | | | | | | |  |
|  |  |  |  |  | No |  | 0.0136 |  | 1 |  | | | | | | |  |
|  | | |  |  |  |  |  |  |  |  | | | | | | | |
|  | | | | | | | | | | | | 1 |  | MTK |  | 7/2/2021 9:22 AM |  |
|  | | | I: Did you think this training was important?  R: it was a very useful training because it equipped us with the necessary skills but at the same time it helped me as a person, it changed me. Much as we were going out there to counsel the women, we were also able to counsel ourselves because of the training that we received. | | | | | | | | | | | | | |  |
|  | | |  | | | | | | | | | | | | | |  |
|  | | | | | | | | | | | | | | | | | |
| Formatted Reports\\Coding Summary by File Formatted Report | | | | | | | | | | | Page 16 of 53 | | | | | | |
| 7/5/2021 9:43 AM | | | | | | | | | | | | | | | | | |
|  | | | **Classification** |  | **Aggregate** |  | **Coverage** |  | **Number Of Coding References** |  | | **Reference Number** |  | **Coded By Initials** |  | **Modified On** |  |
|  | **Files\\THPP PV_#05** | | | | | | | | | | | | | | | |  |
|  | | **Code** | | | | | | | | | | | | | | |  |
|  | | | **Codes\\Health workers** | | | | | | | | | | | | | |  |
|  |  |  |  |  | No |  | 0.0101 |  | 1 |  | | | | | | |  |
|  | | |  |  |  |  |  |  |  |  | | | | | | | |
|  | | | | | | | | | | | | 1 |  | MTK |  | 7/4/2021 2:39 PM |  |
|  | | | I: Can you say that there were no problems with the doctors?  R: There were no problems between them and us because they taught us things that we did not know. | | | | | | | | | | | | | |  |
|  | | |  | | | | | | | | | | | | | |  |
|  | | | **Codes\\Health workers\Relationship** | | | | | | | | | | | | | |  |
|  |  |  |  |  | No |  | 0.0141 |  | 1 |  | | | | | | |  |
|  | | |  |  |  |  |  |  |  |  | | | | | | | |
|  | | | | | | | | | | | | 1 |  | MTK |  | 7/4/2021 2:39 PM |  |
|  | | | I: How were you working with the hospital staff/doctors. How was your relationship like?  R: Our relationship with doctors/hospital staff was good, we have learnt a lot from them which has also changed our lives as well. | | | | | | | | | | | | | |  |
|  | | |  | | | | | | | | | | | | | |  |
|  | | | **Codes\\Personal history** | | | | | | | | | | | | | |  |
|  |  |  |  |  | No |  | 0.0124 |  | 1 |  | | | | | | |  |
|  | | |  |  |  |  |  |  |  |  | | | | | | | |
|  | | | | | | | | | | | | 1 |  | MTK |  | 7/4/2021 2:25 PM |  |
|  | | | I: Can you tell me about this marriage, is it the first marriage or …… how many wives does your husband have, are you the first one?  R: I am the first and only wife, this is my first marriage. | | | | | | | | | | | | | |  |
|  | | |  | | | | | | | | | | | | | |  |
|  | | | **Codes\\Personal history\Age** | | | | | | | | | | | | | |  |
|  |  |  |  |  | No |  | 0.0061 |  | 1 |  | | | | | | |  |
|  | | |  |  |  |  |  |  |  |  | | | | | | | |
|  | | | | | | | | | | | | 1 |  | MTK |  | 7/4/2021 2:08 PM |  |
|  | | | R: I was born in 1989 on 9th September.  I: Thank you, so how old are you now?  R: I am 32 years | | | | | | | | | | | | | |  |
|  | | |  | | | | | | | | | | | | | |  |
|  | | | **Codes\\Personal history\Contact information** | | | | | | | | | | | | | |  |
|  |  |  |  |  | No |  | 0.0117 |  | 1 |  | | | | | | |  |
|  | | |  |  |  |  |  |  |  |  | | | | | | | |
|  | | | | | | | | | | | | 1 |  | MTK |  | 7/4/2021 2:26 PM |  |
|  | | | I: Ok, what is the postal address?  R: No, I have never asked about that.  I: Do you have a phone, what is the number?  R: Yes, ………..  I: So you memorized it?  R: Yes | | | | | | | | | | | | | |  |
|  | | |  | | | | | | | | | | | | | |  |
| Formatted Reports\\Coding Summary by File Formatted Report | | | | | | | | | | | Page 17 of 53 | | | | | | |
| 7/5/2021 9:43 AM | | | | | | | | | | | | | | | | | |
|  | | | **Classification** |  | **Aggregate** |  | **Coverage** |  | **Number Of Coding References** |  | | **Reference Number** |  | **Coded By Initials** |  | **Modified On** |  |
|  | | | **Codes\\Personal history\Level of education** | | | | | | | | | | | | | |  |
|  |  |  |  |  | No |  | 0.0190 |  | 1 |  | | | | | | |  |
|  | | |  |  |  |  |  |  |  |  | | | | | | | |
|  | | | | | | | | | | | | 1 |  | MTK |  | 7/4/2021 2:23 PM |  |
|  | | | I: Ok thank you. How far did you go with your education?  R: Like the training we had?  I: School  R: I went up to standard eight  I: What happened for you to stop on the way?  R: I had no money to pay at the school but I passed class 8 and I stopped. I am able to read and write. | | | | | | | | | | | | | |  |
|  | | |  | | | | | | | | | | | | | |  |
|  | | | **Codes\\Personal history\Location** | | | | | | | | | | | | | |  |
|  |  |  |  |  | No |  | 0.0083 |  | 1 |  | | | | | | |  |
|  | | |  |  |  |  |  |  |  |  | | | | | | | |
|  | | | | | | | | | | | | 1 |  | MTK |  | 7/4/2021 2:25 PM |  |
|  | | | I: Ok thank you. Where do you live?  R: I live in ……………. village,  I: Which traditional authority?  R: Kabudula | | | | | | | | | | | | | |  |
|  | | |  | | | | | | | | | | | | | |  |
|  | | | **Codes\\Personal history\Marital status** | | | | | | | | | | | | | |  |
|  |  |  |  |  | No |  | 0.0040 |  | 1 |  | | | | | | |  |
|  | | |  |  |  |  |  |  |  |  | | | | | | | |
|  | | | | | | | | | | | | 1 |  | MTK |  | 7/4/2021 2:24 PM |  |
|  | | | I: I would like to know, are you married?  R: Yes, I am married | | | | | | | | | | | | | |  |
|  | | |  | | | | | | | | | | | | | |  |
|  | | | **Codes\\Personal history\Number of children** | | | | | | | | | | | | | |  |
|  |  |  |  |  | No |  | 0.0060 |  | 1 |  | | | | | | |  |
|  | | |  |  |  |  |  |  |  |  | | | | | | | |
|  | | | | | | | | | | | | 1 |  | MTK |  | 7/4/2021 2:25 PM |  |
|  | | | I: Ok, how many children did God bless you with?  R: We have three children, 1 girl and 2 boys. | | | | | | | | | | | | | |  |
|  | | |  | | | | | | | | | | | | | |  |
|  | | | **Codes\\Previous work experience\Volunteer experience** | | | | | | | | | | | | | |  |
|  |  |  |  |  | No |  | 0.0152 |  | 1 |  | | | | | | |  |
|  | | |  |  |  |  |  |  |  |  | | | | | | | |
|  | | | | | | | | | | | | 1 |  | MTK |  | 7/4/2021 2:26 PM |  |
|  | | | I: Now, the next part of the discussion. I want to know if you have worked as a health volunteer with any programme before taking part in this study?  R: No, this is my first time  I: So, this is your first time being a volunteer?  R: Yes | | | | | | | | | | | | | |  |
|  | | |  | | | | | | | | | | | | | |  |
| Formatted Reports\\Coding Summary by File Formatted Report | | | | | | | | | | | Page 18 of 53 | | | | | | |
| 7/5/2021 9:43 AM | | | | | | | | | | | | | | | | | |
|  | | | **Classification** |  | **Aggregate** |  | **Coverage** |  | **Number Of Coding References** |  | | **Reference Number** |  | **Coded By Initials** |  | **Modified On** |  |
|  | | | **Codes\\Recommendations** | | | | | | | | | | | | | |  |
|  |  |  |  |  | No |  | 0.1506 |  | 2 |  | | | | | | |  |
|  | | |  |  |  |  |  |  |  |  | | | | | | | |
|  | | | | | | | | | | | | 1 |  | MTK |  | 7/4/2021 2:45 PM |  |
|  | | | I: Thank you very much we are getting to the end of our discussion this afternoon, I would like to hear from you as a person who participated in the study of the thinking healthy, if the study is to continue in future which are the things you feel need to be changed in this intervention.  R: We would like to be given books in time during the training and also consider transportation since we cover long distances  I: On transport what should be done?  R: To look into transport issues.  I: Can you elaborate on that, You are saying that the problem is transport, so what can be done to solve the problem?  R: Provide bicycles in order to reach the woman in goodtime because when we walk on foot we could be late and not keep time because we walk long distances.,  I: Thank you. So you have talked about books and bicycles….. What are the other things?  R: Ooh should I continue [laughing]?  I: Yes things that should be changed to provide quality services?  R: The other thing is changing the venue for training to a different area than where we were. People were asking where we were going, it was difficult to explain because we were taught not to disclose the study before commencement. So the training need to be conducted in a different location.  I: You are saying in your area people know you?  R: Yes they were asking why we have not registered them in the programme, but this was difficult for us since we were not the ones responsible for enrolling people.  I: So you do not want to be asked what you were doing.  R: Yes it should be known when the programme has started.  I: Thank you is there any reason you want the training to be conducted outside the area if any?  R: No, that is all.  I: Is there anything you think need to be changed in the programme apart from what you have already said?  R: If we can be given a little something on top of transport money so that we can be helped to sort out some issues at our homes, so we ask if they can add up some money. | | | | | | | | | | | | | |  |
|  | | |  |  |  |  |  |  |  |  |  |  |  |  |  |  |  |
|  | | |  |  |  |  |  |  |  |  |  |  |  |  |  |  |  |
|  | | |  |  |  |  |  |  |  |  |  |  |  |  |  |  |  |
|  | | |  | | | | | | | | | | | | | |  |
|  | | | | | | | | | | | | 2 |  | MTK |  | 7/4/2021 2:45 PM |  |
|  | | | I: Reaching this far we have come to the end of our discussion; I have been asking you questions, maybe there is something I haven’t asked that you want to talk about?  R: For me, I just want to ask that this programme should continue, because we have learnt a lot in this study, if it continues it can help us a lot and they should continue involving us. | | | | | | | | | | | | | |  |
|  | | |  | | | | | | | | | | | | | |  |
|  | | | **Codes\\THPP delivery experience** | | | | | | | | | | | | | |  |
|  |  |  |  |  | No |  | 0.1326 |  | 4 |  | | | | | | |  |
|  | | |  |  |  |  |  |  |  |  | | | | | | | |
|  | | | | | | | | | | | | 1 |  | MTK |  | 7/4/2021 2:28 PM |  |
|  | | | I: Thank you very much. Now after the training, when you started delivering the intervention what were your experiences in giving the services to the women?  R: I had no problems during the home visits to the women and the mothers were very grateful, although the intervention has just started and hasn’t reached all the women/mothers. Others are asking if the programme can continue so that they can also be helped. Because looking at our area [T/A Kabudula] it is one quarter of the people who have benefited from the programme, and most people wanted the same help looking at how people have seen the programme working. Most people have seen that this intervention is improving the lives of the women and their families. | | | | | | | | | | | | | |  |
|  | | |  |  |  |  |  |  |  |  |  |  |  |  |  |  |  |
|  | | |  | | | | | | | | | | | | | |  |
|  | | | | | | | | | | | | 2 |  | MTK |  | 7/4/2021 2:31 PM |  |
|  | | | I: How were you welcomed in the homes of the participants? can you explain how things were like?  R: We were welcomed by the women and could sit at the place arranged by them and sometimes we would agree on where to sit. At the beginning of each session after greetings we would always assess the woman’s mood, we would ask and hear about her feelings and thoughts, then start that day’s work basing on the woman’s response. | | | | | | | | | | | | | |  |
|  | | |  | | | | | | | | | | | | | |  |
|  | | | | | | | | | | | | 3 |  | MTK |  | 7/4/2021 2:36 PM |  |
|  | | | I: How many women were you visiting?  R: I visited three women. | | | | | | | | | | | | | |  |
|  | | |  | | | | | | | | | | | | | |  |
| Formatted Reports\\Coding Summary by File Formatted Report | | | | | | | | | | | Page 19 of 53 | | | | | | |
| 7/5/2021 9:43 AM | | | | | | | | | | | | | | | | | |
|  | | | **Classification** |  | **Aggregate** |  | **Coverage** |  | **Number Of Coding References** |  | | **Reference Number** |  | **Coded By Initials** |  | **Modified On** |  |
|  | | | | | | | | | | | | | | | | | |
|  | | | | | | | | | | | | 4 |  | MTK |  | 7/4/2021 2:40 PM |  |
|  | | | I: Now I want to hear your views regarding issues you were meeting while providing the thinking healthy programme?  R: During the time I was providing the service, things have also changed for me in my family. because now I know things that I did not know. Because with the help that we were giving the mothers although we were not providing money or other material support but it has helped to improve my life and that of my family as well and would love to still be in the programme if it is to continue.  I: Ok apart from you benefiting from the programme, what were your other experiences in providing the intervention to the women?  R: It has also taught me that not only pregnant women have depression problems, but other people can have anxiety and depression problems too and they can be helped using information from the books we use, even myself. | | | | | | | | | | | | | |  |
|  | | |  |  |  |  |  |  |  |  |  |  |  |  |  |  |  |
|  | | |  | | | | | | | | | | | | | |  |
|  | | | **Codes\\THPP delivery experience\Elements** | | | | | | | | | | | | | |  |
|  |  |  |  |  | No |  | 0.1513 |  | 3 |  | | | | | | |  |
|  | | |  |  |  |  |  |  |  |  | | | | | | | |
|  | | | | | | | | | | | | 1 |  | MTK |  | 7/4/2021 2:35 PM |  |
|  | | | I: What issues were you facing considering that you were meeting the woman at her home together with other family members, her husband, mother, inlaws and other relatives, can you tell me how things were?  R: Upon arrival and greetings we were telling the woman that if the husband is around or the mother/mother in-law should take part in the sessions or other people like in-laws whom she confides in that can keep the confidentiality of the discussion. We were giving the others [the husband, the mother/mother in-law] chance to discuss issues bothering them including discussing what the mother is going through so that we can assist them. Especially issues that the mother shares with her husband. We also explained to them how the intervention will be delivered. We openly told them that we will not be giving monetary help or medication in this programme, but helps in thinking healthy that one should have helpful thinking. | | | | | | | | | | | | | |  |
|  | | |  |  |  |  |  |  |  |  |  |  |  |  |  |  |  |
|  | | |  | | | | | | | | | | | | | |  |
|  | | | | | | | | | | | | 2 |  | MTK |  | 7/4/2021 2:35 PM |  |
|  | | | I: What were the materials that helped you in delivering services?  R: Materials that helped us delivering the services are; we had a health calendar and a book/manual for us counsellors used in teaching the women. When teaching the woman, we used pictures of unhelpful thoughts and behaviors and discussed how these can affect her. We would then show the woman pictures of helpful thoughts and behavior and emphasized that the purpose is to help woman change her way of thinking and behavior through discussing issues and worries she is having. After such discussions the woman was able to see how things are changing in her life. | | | | | | | | | | | | | |  |
|  | | |  | | | | | | | | | | | | | |  |
|  | | | | | | | | | | | | 3 |  | MTK |  | 7/4/2021 2:38 PM |  |
|  | | | I: OK, I would like to know if the women were able to do assignments given and work on them before the next meeting or were there any problems?  R: I could find all the assignments done because once we are done with a particular session, once we are sure that they have understood, we would give them an assignment on the topics covered like time for resting, eating the six food groups. Before starting the next session, we would ask for the assignment, she would give us feedback on assignments/homework she had done, like testifying that she has enough resting time than before and trying to eat the six food groups with support from the husband although it was a difficult season to find everything. But she was able to differentiate the support she was getting from husband now than previously. | | | | | | | | | | | | | |  |
|  | | |  |  |  |  |  |  |  |  |  |  |  |  |  |  |  |
|  | | |  | | | | | | | | | | | | | |  |
|  | | | **Codes\\THPP delivery experience\Elements\Barriers** | | | | | | | | | | | | | |  |
|  |  |  |  |  | No |  | 0.0314 |  | 1 |  | | | | | | |  |
|  | | |  |  |  |  |  |  |  |  | | | | | | | |
|  | | | | | | | | | | | | 1 |  | MTK |  | 7/4/2021 2:42 PM |  |
|  | | | I: Thank you very much, you said the session where you were laying foundation of the intervention was a bit difficult for you, what do you think was the problem?  R: The introduction session difficult because the intervention was new and we were meeting the family for the first time. I didn’t know where and how to start, but the rest of the sessions were easy. | | | | | | | | | | | | | |  |
|  | | |  | | | | | | | | | | | | | |  |
|  | | | | | | | | | | | | | | | | | |
| Formatted Reports\\Coding Summary by File Formatted Report | | | | | | | | | | | Page 20 of 53 | | | | | | |
| 7/5/2021 9:43 AM | | | | | | | | | | | | | | | | | |
|  | | | **Classification** |  | **Aggregate** |  | **Coverage** |  | **Number Of Coding References** |  | | **Reference Number** |  | **Coded By Initials** |  | **Modified On** |  |
|  | | | **Codes\\THPP delivery experience\Elements\Challenges** | | | | | | | | | | | | | |  |
|  |  |  |  |  | No |  | 0.0524 |  | 2 |  | | | | | | |  |
|  | | |  |  |  |  |  |  |  |  | | | | | | | |
|  | | | | | | | | | | | | 1 |  | MTK |  | 7/4/2021 2:29 PM |  |
|  | | | I: Ok what were the problems that you experienced in giving this service?  R: When starting the programme there were no problems only that the intervention was new to us and the books to use when teaching were brought to on the second day of training, so it took time for us to master what is in the books/manuals to teach the women. we were learning how to use it first and due to good organization we managed to do the work. | | | | | | | | | | | | | |  |
|  | | |  | | | | | | | | | | | | | |  |
|  | | | | | | | | | | | | 2 |  | MTK |  | 7/4/2021 2:30 PM |  |
|  | | | I: During the time you were providing the service, was there anything that you lacked to do your job like the materials?  R: We lacked plastic carrier bags in the rainy season to protect the materials and mode of transport because most of us do not have bicycles to cover long distances and we depended on hired bicycles and when you do not have money you could walk on foot and reach the area late. | | | | | | | | | | | | | |  |
|  | | |  | | | | | | | | | | | | | |  |
|  | | | **Codes\\THPP delivery experience\Elements\Difficult to deliver** | | | | | | | | | | | | | |  |
|  |  |  |  |  | No |  | 0.0666 |  | 2 |  | | | | | | |  |
|  | | |  |  |  |  |  |  |  |  | | | | | | | |
|  | | | | | | | | | | | | 1 |  | MTK |  | 7/4/2021 2:42 PM |  |
|  | | | I: From your experience, which areas were easy to deliver in this intervention and which areas were difficult?  R: I had problems on laying foundation of the programme, the first session was a bit difficult for me. But when reading through the book, I learned a lot and when I proceeded providing sessions about relationship of mother and the people surrounding her, it became easier helping the woman because we realized that when a person lives alone, she has unhelpful behavior. | | | | | | | | | | | | | |  |
|  | | |  | | | | | | | | | | | | | |  |
|  | | | | | | | | | | | | 2 |  | MTK |  | 7/4/2021 2:43 PM |  |
|  | | | I: Meaning that the only problem was on laying the foundation?  R: Yes.  I: Were the problems on laying foundation the same among the three women or it differed?  R: The problems differed, when we went to the first woman, i was accompanied by the doctors/ our counsellors to help each other in initiating the programme and when I went to the second woman, I saw that I had some problems, but the woman was able to understand although I had problems following through the process. I would realize that I missed something, but I could repeat where necessary. | | | | | | | | | | | | | |  |
|  | | |  |  |  |  |  |  |  |  |  |  |  |  |  |  |  |
|  | | |  | | | | | | | | | | | | | |  |
|  | | | **Codes\\THPP delivery experience\Elements\Easy to deliver** | | | | | | | | | | | | | |  |
|  |  |  |  |  | No |  | 0.0428 |  | 2 |  | | | | | | |  |
|  | | |  |  |  |  |  |  |  |  | | | | | | | |
|  | | | | | | | | | | | | 1 |  | MTK |  | 7/4/2021 2:42 PM |  |
|  | | | I: Ok but I want to know which areas were easy for the women to understand, or which areas brought a lot of questions showing that they were unable to understand?  R: No, they were helped, they could listen properly and when I asked them, they understood and would do the homework very well.  I: Was there any difficult part for the women to learn or you to teach?  R: No there was no problem. | | | | | | | | | | | | | |  |
|  | | |  | | | | | | | | | | | | | |  |
|  | | | | | | | | | | | | 2 |  | MTK |  | 7/4/2021 2:44 PM |  |
|  | | | I: So you went alone to the second woman?  R: Yes  I: To the third woman?  R: This time I had no problems because I mastered the book and was used without any problems.  I: If given chance to go to the fourth woman or more, it could be easier?  R: Yes it would be helpful. | | | | | | | | | | | | | |  |
|  | | |  | | | | | | | | | | | | | |  |
|  | | | | | | | | | | | | | | | | | |
| Formatted Reports\\Coding Summary by File Formatted Report | | | | | | | | | | | Page 21 of 53 | | | | | | |
| 7/5/2021 9:43 AM | | | | | | | | | | | | | | | | | |
|  | | | **Classification** |  | **Aggregate** |  | **Coverage** |  | **Number Of Coding References** |  | | **Reference Number** |  | **Coded By Initials** |  | **Modified On** |  |
|  | | | **Codes\\THPP delivery experience\Elements\Facilitators** | | | | | | | | | | | | | |  |
|  |  |  |  |  | No |  | 0.0409 |  | 2 |  | | | | | | |  |
|  | | |  |  |  |  |  |  |  |  | | | | | | | |
|  | | | | | | | | | | | | 1 |  | MTK |  | 7/4/2021 2:30 PM |  |
|  | | | I: What can you explain about the time required to give counselling sessions per day? Or what can be the changes as you have seen it.  R: The time for the meeting was good and there were no changes agreed with the women. | | | | | | | | | | | | | |  |
|  | | |  | | | | | | | | | | | | | |  |
|  | | | | | | | | | | | | 2 |  | MTK |  | 7/4/2021 2:43 PM |  |
|  | | | I: Which part can you say went very well?  R: The areas that went on well are; teaching from session 2 up to 7, I had no problems because I was able to read through the sessions, guide the woman, give her time to internalize the content and ask questions. the woman could be seen understanding the topics and felt that I have really taught the lessons very well, there was no topic that gave me problems from the book. | | | | | | | | | | | | | |  |
|  | | |  | | | | | | | | | | | | | |  |
|  | | | **Codes\\THPP training** | | | | | | | | | | | | | |  |
|  |  |  |  |  | No |  | 0.0340 |  | 1 |  | | | | | | |  |
|  | | |  |  |  |  |  |  |  |  | | | | | | | |
|  | | | | | | | | | | | | 1 |  | MTK |  | 7/4/2021 2:27 PM |  |
|  | | | I: Thank you, now back to the study that you participated in, before commencement of this study did you have any training to equip you with knowledge and skills to deliver the intervention?  R: Yes, we had a five days training, we were just called to go for the training about pregnant woman’s health and those who has babies. We had a five days training but the challenge was that some of the materials came late, this is what seemed to be a challenge. | | | | | | | | | | | | | |  |
|  | | |  | | | | | | | | | | | | | |  |
|  | | | **Codes\\THPP training\Preparedness** | | | | | | | | | | | | | |  |
|  |  |  |  |  | No |  | 0.0204 |  | 1 |  | | | | | | |  |
|  | | |  |  |  |  |  |  |  |  | | | | | | | |
|  | | | | | | | | | | | | 1 |  | MTK |  | 7/4/2021 2:28 PM |  |
|  | | | I: Ok, we will come to that but I would like to know about the training itself despite what you have said can you say that the training was helpful and adequate?  R: The training was adequate and….. [I have forgotten a little bit]  I: Was it helpful?  R: It was very helpful because we knew what we did not know before. | | | | | | | | | | | | | |  |
|  | | |  | | | | | | | | | | | | | |  |
|  | **Files\\THPP PV_#06** | | | | | | | | | | | | | | | |  |
|  | | **Code** | | | | | | | | | | | | | | |  |
|  | | | **Codes\\Health workers\Interaction** | | | | | | | | | | | | | |  |
|  |  |  |  |  | No |  | 0.0292 |  | 1 |  | | | | | | |  |
|  | | |  |  |  |  |  |  |  |  | | | | | | | |
|  | | | | | | | | | | | | 1 |  | MTK |  | 7/4/2021 3:08 PM |  |
|  | | | I: Ok thank you, let us turn to another section a bit. You worked with the women and the doctors at the hospital, how did you work with the doctors/hospital staff.  R: We worked with the doctors together as one without looking at qualification differences, that even when we visited the women they could not see the difference that this is more educated than this one and the doctors/hospital staff referred the women to us for counselling and because of that women were happy that they told us everything knowing that this person is from our community and the others are doctors from the hospital, meaning this programme is recognized and very important. | | | | | | | | | | | | | |  |
|  | | |  | | | | | | | | | | | | | |  |
| Formatted Reports\\Coding Summary by File Formatted Report | | | | | | | | | | | Page 22 of 53 | | | | | | |
| 7/5/2021 9:43 AM | | | | | | | | | | | | | | | | | |
|  | | | **Classification** |  | **Aggregate** |  | **Coverage** |  | **Number Of Coding References** |  | | **Reference Number** |  | **Coded By Initials** |  | **Modified On** |  |
|  | | | **Codes\\Health workers\Relationship** | | | | | | | | | | | | | |  |
|  |  |  |  |  | No |  | 0.0292 |  | 1 |  | | | | | | |  |
|  | | |  |  |  |  |  |  |  |  | | | | | | | |
|  | | | | | | | | | | | | 1 |  | MTK |  | 7/4/2021 3:08 PM |  |
|  | | | I: Ok thank you, let us turn to another section a bit. You worked with the women and the doctors at the hospital, how did you work with the doctors/hospital staff.  R: We worked with the doctors together as one without looking at qualification differences, that even when we visited the women they could not see the difference that this is more educated than this one and the doctors/hospital staff referred the women to us for counselling and because of that women were happy that they told us everything knowing that this person is from our community and the others are doctors from the hospital, meaning this programme is recognized and very important. | | | | | | | | | | | | | |  |
|  | | |  | | | | | | | | | | | | | |  |
|  | | | **Codes\\Involvement of family members** | | | | | | | | | | | | | |  |
|  |  |  |  |  | No |  | 0.0379 |  | 1 |  | | | | | | |  |
|  | | |  |  |  |  |  |  |  |  | | | | | | | |
|  | | | | | | | | | | | | 1 |  | MTK |  | 7/4/2021 3:04 PM |  |
|  | | | I: Can you tell me what used to happen when you get to the home of a woman, where you have also met other family members, her mother-in law or the husband how were things like?  R: Let me explain how it went. During the first visit we went to the house humbly, knock and they welcomed us, I would usually be seated facing the patient and asked if she was ready for the session and ask if they are willing to be together with husband or mother in-law, if she says yes we could have the discussions together. All the women I was following up accepted to have discussions with husband or mother-in law which helped them to be close to the woman and support the woman with her problems then find solutions. Sometimes we found that the in-laws were not very free with the mother, but through the sessions, things changed | | | | | | | | | | | | | |  |
|  | | |  |  |  |  |  |  |  |  |  |  |  |  |  |  |  |
|  | | |  | | | | | | | | | | | | | |  |
|  | | | **Codes\\Personal history** | | | | | | | | | | | | | |  |
|  |  |  |  |  | No |  | 0.0313 |  | 2 |  | | | | | | |  |
|  | | |  |  |  |  |  |  |  |  | | | | | | | |
|  | | | | | | | | | | | | 1 |  | MTK |  | 7/4/2021 2:51 PM |  |
|  | | | I: The marriage you have, is it your first marriage or …….?  R: No I have never changed men this is my first marriage with my husband and we have 4 children.  I: Ok how many wives does your husband have or are you the only wife?  R: My husband has three wives and I am the first one.  I: Meaning has married two times after you?  R: Yes it is like that. | | | | | | | | | | | | | |  |
|  | | |  | | | | | | | | | | | | | |  |
|  | | | | | | | | | | | | 2 |  | MTK |  | 7/4/2021 2:53 PM |  |
|  | | | I: Thank you. You have said you have four children, how many times have you given birth?  R: Three children died, the first, third and fourth born. The second, fifth, sixth and seventh are alive.  I: So you gave birth 7 times.  R: Yes  I: Thank you. So can we say you stopped bearing children?  R: Yes I stoped in 2004. | | | | | | | | | | | | | |  |
|  | | |  | | | | | | | | | | | | | |  |
|  | | | **Codes\\Personal history\Age** | | | | | | | | | | | | | |  |
|  |  |  |  |  | No |  | 0.0189 |  | 1 |  | | | | | | |  |
|  | | |  |  |  |  |  |  |  |  | | | | | | | |
|  | | | | | | | | | | | | 1 |  | MTK |  | 7/4/2021 2:50 PM |  |
|  | | | I: We are starting our discussion; the first section is that I would like to know more about your demographic information. When were you born if you can remember the date and year?  R: I was born on 27 October 1970.  I: Thank you, if you calculate, how many years do you have?  R: I have not done the mathematics…… (Laughter)  I: Ok (laughter) this October you will be 51 years, according to my calculation. | | | | | | | | | | | | | |  |
|  | | |  | | | | | | | | | | | | | |  |
| Formatted Reports\\Coding Summary by File Formatted Report | | | | | | | | | | | Page 23 of 53 | | | | | | |
| 7/5/2021 9:43 AM | | | | | | | | | | | | | | | | | |
|  | | | **Classification** |  | **Aggregate** |  | **Coverage** |  | **Number Of Coding References** |  | | **Reference Number** |  | **Coded By Initials** |  | **Modified On** |  |
|  | | | **Codes\\Personal history\Contact information** | | | | | | | | | | | | | |  |
|  |  |  |  |  | No |  | 0.0220 |  | 1 |  | | | | | | |  |
|  | | |  |  |  |  |  |  |  |  | | | | | | | |
|  | | | | | | | | | | | | 1 |  | MTK |  | 7/4/2021 2:54 PM |  |
|  | | | I: Do you have a contact address used in receiving letters?  R: Since the postal mail stopped, I get communications through phone.  I: Yes it is true that nowadays people use phones for communication. Do you have a phone?  R: Yes I have it.  I: what is the number?  R: …………………….  I: I can see that you memorized it.  R: Yes, we used to do this at school even the addresses we could say by heart…. I went to …………………… primary school. (Laughter) | | | | | | | | | | | | | |  |
|  | | |  |  |  |  |  |  |  |  |  |  |  |  |  |  |  |
|  | | |  | | | | | | | | | | | | | |  |
|  | | | **Codes\\Personal history\Level of education** | | | | | | | | | | | | | |  |
|  |  |  |  |  | No |  | 0.0186 |  | 1 |  | | | | | | |  |
|  | | |  |  |  |  |  |  |  |  | | | | | | | |
|  | | | | | | | | | | | | 1 |  | MTK |  | 7/4/2021 2:50 PM |  |
|  | | | I would like to know about your education.  R: I did standard 8 in 1988.  I: We hear that at that time standard 8 was very powerful  R: Yes it is true because at that time a student could be in the class for many years without chance of selection to secondary which is totally different to what is happening now where they are day secondary schools to help in students in education to reach form four. | | | | | | | | | | | | | |  |
|  | | |  | | | | | | | | | | | | | |  |
|  | | | **Codes\\Personal history\Location** | | | | | | | | | | | | | |  |
|  |  |  |  |  | No |  | 0.0034 |  | 1 |  | | | | | | |  |
|  | | |  |  |  |  |  |  |  |  | | | | | | | |
|  | | | | | | | | | | | | 1 |  | MTK |  | 7/4/2021 2:53 PM |  |
|  | | | I: Thank you. Where do you stay?  R: I live in ………….. village T/A Kabudula. | | | | | | | | | | | | | |  |
|  | | |  | | | | | | | | | | | | | |  |
|  | | | **Codes\\Personal history\Marital status** | | | | | | | | | | | | | |  |
|  |  |  |  |  | No |  | 0.0047 |  | 1 |  | | | | | | |  |
|  | | |  |  |  |  |  |  |  |  | | | | | | | |
|  | | | | | | | | | | | | 1 |  | MTK |  | 7/4/2021 2:50 PM |  |
|  | | | I: Ok thank you. I would like to know about your marriage life, are you married?  R: Yes I am married. | | | | | | | | | | | | | |  |
|  | | |  | | | | | | | | | | | | | |  |
|  | | | **Codes\\Personal history\Number of children** | | | | | | | | | | | | | |  |
|  |  |  |  |  | No |  | 0.0072 |  | 1 |  | | | | | | |  |
|  | | |  |  |  |  |  |  |  |  | | | | | | | |
|  | | | | | | | | | | | | 1 |  | MTK |  | 7/4/2021 2:52 PM |  |
|  | | | I: The marriage you have, is it your first marriage or …….?  R: No I have never changed men this is my first marriage with my husband and we have 4 children. | | | | | | | | | | | | | |  |
|  | | |  | | | | | | | | | | | | | |  |
| Formatted Reports\\Coding Summary by File Formatted Report | | | | | | | | | | | Page 24 of 53 | | | | | | |
| 7/5/2021 9:43 AM | | | | | | | | | | | | | | | | | |
|  | | | **Classification** |  | **Aggregate** |  | **Coverage** |  | **Number Of Coding References** |  | | **Reference Number** |  | **Coded By Initials** |  | **Modified On** |  |
|  | | | **Codes\\Previous work experience\Volunteer experience** | | | | | | | | | | | | | |  |
|  |  |  |  |  | No |  | 0.0428 |  | 3 |  | | | | | | |  |
|  | | |  |  |  |  |  |  |  |  | | | | | | | |
|  | | | | | | | | | | | | 1 |  | MTK |  | 7/4/2021 2:54 PM |  |
|  | | | I: Ok we are done with the first part on demographics, let us go to the study part but before that I would like to know if you have ever worked as a volunteer in any health-related area.  R: Yes, I worked as a volunteer for UNICEF doing follow ups on malnourished children by using the MUAC measurements. | | | | | | | | | | | | | |  |
|  | | |  | | | | | | | | | | | | | |  |
|  | | | | | | | | | | | | 2 |  | MTK |  | 7/4/2021 2:55 PM |  |
|  | | | I: At that time what were the facilitators or challenges you were meeting?  R: Everyone needs to be trained on doing things, in that time because we were trained, most people were trained on child feeding and benefited because their children were saved from malnutrition related deaths.  I: Ok any challenges that you met in doing your job that time?  R: At that time there were no problems because when people talk to someone from the same community they relax, understand each other better and open up. There were no problems, that time I covered Khongoni area. | | | | | | | | | | | | | |  |
|  | | |  | | | | | | | | | | | | | |  |
|  | | | | | | | | | | | | 3 |  | MTK |  | 7/4/2021 2:56 PM |  |
|  | | | I: Which area was this?  R: It was within Khongoni area. | | | | | | | | | | | | | |  |
|  | | |  | | | | | | | | | | | | | |  |
|  | | | **Codes\\Previous work experience\Volunteer experience\Activities** | | | | | | | | | | | | | |  |
|  |  |  |  |  | No |  | 0.0141 |  | 1 |  | | | | | | |  |
|  | | |  |  |  |  |  |  |  |  | | | | | | | |
|  | | | | | | | | | | | | 1 |  | MTK |  | 7/4/2021 2:56 PM |  |
|  | | | I: Ok we are done with the first part on demographics, let us go to the study part but before that I would like to know if you have ever worked as a volunteer in any health-related area.  R: Yes, I worked as a volunteer for UNICEF doing follow ups on malnourished children by using the MUAC measurements. | | | | | | | | | | | | | |  |
|  | | |  | | | | | | | | | | | | | |  |
|  | | | **Codes\\Previous work experience\Volunteer experience\Name of organization** | | | | | | | | | | | | | |  |
|  |  |  |  |  | No |  | 0.0141 |  | 1 |  | | | | | | |  |
|  | | |  |  |  |  |  |  |  |  | | | | | | | |
|  | | | | | | | | | | | | 1 |  | MTK |  | 7/4/2021 2:55 PM |  |
|  | | | I: Ok we are done with the first part on demographics, let us go to the study part but before that I would like to know if you have ever worked as a volunteer in any health-related area.  R: Yes, I worked as a volunteer for UNICEF doing follow ups on malnourished children by using the MUAC measurements. | | | | | | | | | | | | | |  |
|  | | |  | | | | | | | | | | | | | |  |
|  | | | **Codes\\Recommendations** | | | | | | | | | | | | | |  |
|  |  |  |  |  | No |  | 0.1608 |  | 3 |  | | | | | | |  |
|  | | |  |  |  |  |  |  |  |  | | | | | | | |
|  | | | | | | | | | | | | 1 |  | MTK |  | 7/4/2021 3:15 PM |  |
|  | | | I: Ok we are approaching the end of our discussions, my last question is according to how you have seen the study, which part needs to be reviewed whether the training or provision of counselling so that we can improve and do better than this in case the programme comes back?  R: When the programme comes again it should consider having more beneficiaries selected from all the villages surrounding Kabudula catchment health area for example I come from ………… which is also a VDC on its own and our area is vast, but there were no beneficiaries from ………... Maybe because Kabudula area is vast. But in future the programme should involve more beneficiaries from all VDCs surrounding the hospital. Most people just heard of this programme as news for it came from the chiefs but even some chiefs do not have beneficiaries from their village. So if this programme will be rolled out consider selecting a woman or nurse special for the programme to work with health staff in recruiting more women from all the VDC and other areas surrounding the health facility. Yes, this was a study but this programme is very powerful and helpful. It has brought peace in the families and in the minds of people who had anxieties and depression. It has helped them think healthily. There are still others who does not know helpful actions from unhelpful actions. If the programme is to continue, they should add some more people in this community. | | | | | | | | | | | | | |  |
|  | | |  |  |  |  |  |  |  |  |  |  |  |  |  |  |  |
|  | | |  | | | | | | | | | | | | | |  |
| Formatted Reports\\Coding Summary by File Formatted Report | | | | | | | | | | | Page 25 of 53 | | | | | | |
| 7/5/2021 9:43 AM | | | | | | | | | | | | | | | | | |
|  | | | **Classification** |  | **Aggregate** |  | **Coverage** |  | **Number Of Coding References** |  | | **Reference Number** |  | **Coded By Initials** |  | **Modified On** |  |
|  | | | | | | | | | | | | | | | | | |
|  | | | | | | | | | | | | 2 |  | MTK |  | 7/4/2021 3:17 PM |  |
|  | | | I: Thank you. In your earlier explanation you said time for the training was short, how was it to be done?  R: would like to consider additional time for the training so that we can do our work properly together with the doctors  I: What exactly would you love to be added?  R: Adding material help to the women, something like giving soap incentive, when we were giving them gifts of soap to help them the women were very happy looking at the poverty levels in the villages.  I: Ok you said the training was not enough, what do you want to be added for you said the training was for five days, I would like to know from you as a counsellor what was to be done?  R: If there can be a chance next time the training to be conducted at a neutral place outside the community to ensure that everyone concentrated on the training. And also, although we were doing voluntary work, they should have considered a little something for soap apart from transport provision because even a volunteer want to be clean and smart. | | | | | | | | | | | | | |  |
|  | | |  |  |  |  |  |  |  |  |  |  |  |  |  |  |  |
|  | | |  |  |  |  |  |  |  |  |  |  |  |  |  |  |  |
|  | | |  | | | | | | | | | | | | | |  |
|  | | | | | | | | | | | | 3 |  | MTK |  | 7/4/2021 3:17 PM |  |
|  | | | I: Reaching this far we can end our discussion but before doing that is there anything that has not been asked and you want to talk about it regarding the study?  R: Thank you the other need for the study is to look in to transportation issues since the work is done in villages by giving bicycles to counsellors according to the roads, we have here at kabudula for the visited women are not along the tarmac road, others live very far like from here to Kang’ombe, Kadala, chikho or Nsanama all these surround Kabudula hospital. | | | | | | | | | | | | | |  |
|  | | |  | | | | | | | | | | | | | |  |
|  | | | **Codes\\Supervision** | | | | | | | | | | | | | |  |
|  |  |  |  |  | No |  | 0.0539 |  | 2 |  | | | | | | |  |
|  | | |  |  |  |  |  |  |  |  | | | | | | | |
|  | | | | | | | | | | | | 1 |  | MTK |  | 7/4/2021 3:09 PM |  |
|  | | | I: So you can say that there were no misunderstandings between you and the doctors from the hospital?  R: No there were no misunderstandings because their work was to remind us if there were some problems after training and make follow up on how the home visits are going. We joined hands in doing this job. | | | | | | | | | | | | | |  |
|  | | |  | | | | | | | | | | | | | |  |
|  | | | | | | | | | | | | 2 |  | MTK |  | 7/4/2021 3:15 PM |  |
|  | | | I: Ok but I still wanted to know according to what you were taught, which parts were difficult or not difficult for you to do, For example the first session was introduction were you able to explain it or had some difficulties? Did you feel that you have delivered the session fully as expected or missed something because you had problems, please explain if there was something like that?  R: I did introduction session very well. The good part was we were not alone in the programme we could meet the doctors often who were helping us how to go about the sessions, how to deal with issues, even if we forget they would remind us how to start session up to the end. There was good coordination with the doctors. So, incase this programme comes again it will be good to add on skills because education and skills helps in the development of the community. | | | | | | | | | | | | | |  |
|  | | |  |  |  |  |  |  |  |  |  |  |  |  |  |  |  |
|  | | |  | | | | | | | | | | | | | |  |
|  | | | **Codes\\THPP delivery experience** | | | | | | | | | | | | | |  |
|  |  |  |  |  | No |  | 0.1683 |  | 6 |  | | | | | | |  |
|  | | |  |  |  |  |  |  |  |  | | | | | | | |
|  | | | | | | | | | | | | 1 |  | MTK |  | 7/4/2021 2:58 PM |  |
|  | | | I: Let us proceed, as you were visiting the women helping them in the thinking healthy programme what were your experiences?  R: when we were teaching the women thinking healthy, helpful thinking and behavior, and knowing helpful and unhelpful actions it was found that they were helped because there were some changes noticed in how they were before and after the programme. Although the programme found them when they were not expecting it but they had the interest to even help their relatives but since this was not part of the programme, we stopped where we were supposed to stop. When at home we are asked questions and help others who are not in this program so that they can also have helpful thinking. | | | | | | | | | | | | | |  |
|  | | |  | | | | | | | | | | | | | |  |
| Formatted Reports\\Coding Summary by File Formatted Report | | | | | | | | | | | Page 26 of 53 | | | | | | |
| 7/5/2021 9:43 AM | | | | | | | | | | | | | | | | | |
|  | | | **Classification** |  | **Aggregate** |  | **Coverage** |  | **Number Of Coding References** |  | | **Reference Number** |  | **Coded By Initials** |  | **Modified On** |  |
|  | | | | | | | | | | | | | | | | | |
|  | | | | | | | | | | | | 2 |  | MTK |  | 7/4/2021 2:59 PM |  |
|  | | | I: Ok, what were the difficulties in providing this program?  R: The problem in giving out help in doing home visits is that some people wanted to be part of the programme but it was not possible because we were getting only those referred to us by the doctors/hospital staff. This happened because women shared with others about the services given to them and they also wanted to benefit and we told them that they will be included next time to avoid disappointing them. | | | | | | | | | | | | | |  |
|  | | |  | | | | | | | | | | | | | |  |
|  | | | | | | | | | | | | 3 |  | MTK |  | 7/4/2021 3:05 PM |  |
|  | | | I: You said were facing the patient, were you calling them patients… (Laughter)  R: Yes according to the condition they were in because stress is a big health problem more than malaria then when recovering we could see that there is improvement.  I: So they were patients who recovered, so you were a doctor? (Laughter)  R: We were almost there because when coming they would say the counsellor is coming and we felt honored. | | | | | | | | | | | | | |  |
|  | | |  | | | | | | | | | | | | | |  |
|  | | | | | | | | | | | | 4 |  | MTK |  | 7/4/2021 3:10 PM |  |
|  | | | I: Ok can you tell me the challenges in providing the service as expected?  R: There was no challenge with the woman in the programme but there were others who were interested and waiting outside the house for us so that we can also deliver the intervention in their homes. This was difficult for us because these women were referred from the hospital with depression problems and we could not just include anyone in the program. We just told them to wait for another time. | | | | | | | | | | | | | |  |
|  | | |  | | | | | | | | | | | | | |  |
|  | | | | | | | | | | | | 5 |  | MTK |  | 7/4/2021 3:11 PM |  |
|  | | | I: So you mean there was no problem with the woman in the programme but may be those not in the study who wanted to be helped as well but was not possible.  R: Yes they saw this new programme, they were interested and wanted us to be their doctors at their home but we told them that we are doing a study, if possible they will be included next time. | | | | | | | | | | | | | |  |
|  | | |  | | | | | | | | | | | | | |  |
|  | | | | | | | | | | | | 6 |  | MTK |  | 7/4/2021 3:12 PM |  |
|  | | | I: Ok so after discussing all these I would like to know about the study, what part of the study do you think is going well or not going well?  R: What went well was the encouragement we got and then, sharing of ideas with the women we were helping. The women were been encouraged by us counsellors chosen by the doctors and they benefited a lot. It was also very prestigious for us to be known as counselors in the community. We are also proud to see that there are healthy children born in the program/after our counselling. The only problem is that the program has run for a short period and it helped a small group of people while there are many women with health problems in our community and the whole country in need of help. So, this needed to be a big programme not just a study to benefit a lot of people in our community. It’s a good programme because we are saving newly born babies’ lives, emotionally stable children and have healthy thinking citizens because children born from depressed families would also be affected emotionally, they would be stunted and dull. This programme helped to shape good future for children once parents dedicate themselves to this programme. | | | | | | | | | | | | | |  |
|  | | |  |  |  |  |  |  |  |  |  |  |  |  |  |  |  |
|  | | |  | | | | | | | | | | | | | |  |
|  | | | **Codes\\THPP delivery experience\Elements** | | | | | | | | | | | | | |  |
|  |  |  |  |  | No |  | 0.1225 |  | 4 |  | | | | | | |  |
|  | | |  |  |  |  |  |  |  |  | | | | | | | |
|  | | | | | | | | | | | | 1 |  | MTK |  | 7/4/2021 3:05 PM |  |
|  | | | I: Okay thank you very much, I would like to know things that helped you in providing counselling like books and other materials were they enough or what can you say?  R: The books were adequate though at first, during training some had Indian pictures but later we got traditional ones. And with what we learnt we were able to help the mothers with explanations following the pictures. and we helped explain to them with what was in the pictures and ask them what they see, they could answer accordingly. At first they saw it as strange but later they got used all was well. | | | | | | | | | | | | | |  |
|  | | |  | | | | | | | | | | | | | |  |
|  | | | | | | | | | | | | 2 |  | MTK |  | 7/4/2021 3:07 PM |  |
|  | | | I: How many women did you have?  R: I visited three women. | | | | | | | | | | | | | |  |
|  | | |  | | | | | | | | | | | | | |  |
|  | | | | | | | | | | | | | | | | | |
| Formatted Reports\\Coding Summary by File Formatted Report | | | | | | | | | | | Page 27 of 53 | | | | | | |
| 7/5/2021 9:43 AM | | | | | | | | | | | | | | | | | |
|  | | | **Classification** |  | **Aggregate** |  | **Coverage** |  | **Number Of Coding References** |  | | **Reference Number** |  | **Coded By Initials** |  | **Modified On** |  |
|  | | | | | | | | | | | | | | | | | |
|  | | | | | | | | | | | | 3 |  | MTK |  | 7/4/2021 3:12 PM |  |
|  | | | I: Ok so after discussing all these I would like to know about the study, what part of the study do you think is going well or not going well?  R: What went well was the encouragement we got and then, sharing of ideas with the women we were helping. The women were been encouraged by us counsellors chosen by the doctors and they benefited a lot. It was also very prestigious for us to be known as counselors in the community. We are also proud to see that there are healthy children born in the program/after our counselling. The only problem is that the program has run for a short period and it helped a small group of people while there are many women with health problems in our community and the whole country in need of help. So, this needed to be a big programme not just a study to benefit a lot of people in our community. It’s a good programme because we are saving newly born babies’ lives, emotionally stable children and have healthy thinking citizens because children born from depressed families would also be affected emotionally, they would be stunted and dull. This programme helped to shape good future for children once parents dedicate themselves to this programme. | | | | | | | | | | | | | |  |
|  | | |  |  |  |  |  |  |  |  |  |  |  |  |  |  |  |
|  | | |  | | | | | | | | | | | | | |  |
|  | | | | | | | | | | | | 4 |  | MTK |  | 7/4/2021 3:13 PM |  |
|  | | | I: Thank you, so you are saying you have reached few people and could have loved to reach more. Out of the few eligible ones, what were the difficulties or what went on well as you were delivering the intervention.  R: For those eligible what went well is that after getting counselling they could show some improvement and when given tasks they were able to do the tasks. When you assess their mood, when you asked them where they are by marking on the pictures related to them you could see that the programme is helping them. But there was one woman who was not really improving because of the problems she had in her marriage, we did our part but we did not want to be involved in other issues that were beyond us, we referred her to the chief. However, we helped her to have helpful thinking as counselors. | | | | | | | | | | | | | |  |
|  | | |  |  |  |  |  |  |  |  |  |  |  |  |  |  |  |
|  | | |  | | | | | | | | | | | | | |  |
|  | | | **Codes\\THPP delivery experience\Elements\Challenges** | | | | | | | | | | | | | |  |
|  |  |  |  |  | No |  | 0.0663 |  | 2 |  | | | | | | |  |
|  | | |  |  |  |  |  |  |  |  | | | | | | | |
|  | | | | | | | | | | | | 1 |  | MTK |  | 7/4/2021 3:09 PM |  |
|  | | | I: Now let us talk about you as a person and counsellor who took the intervention to the woman at the village. What have you come across in conducting your services especially delivering the thinking healthy programme to the women?  R: We had a problem of transportation since we could reach far distances and you do not have enough money but because we agreed to work voluntarily to help these women we made it possible to reach and help her for free at no cost and without exchanging with anything to help her life and the baby. Even in times of rain we could still go and did not bother her to prepare something for us, no……..what we do was counselling as taught then she was left to do her daily chores. We reminded her that our work is to help her to have healthy life regarding stress. | | | | | | | | | | | | | |  |
|  | | |  |  |  |  |  |  |  |  |  |  |  |  |  |  |  |
|  | | |  | | | | | | | | | | | | | |  |
|  | | | | | | | | | | | | 2 |  | MTK |  | 7/4/2021 3:14 PM |  |
|  | | | I: Thank you, now I would like to know what part was not difficult for you to deliver and what part was difficult and if there was something that you failed to deliver to the women?  R: The easy part was providing counselling while partner was present, especially if their relationship was good, for he took part in helping the woman to make her decision…. what was difficult was when we were dealing with women who had no partners. For example, I was visiting one woman who was alone, the husband rejected her and the baby because he said he was not the father of this baby. This was difficult for us to involve him in the intervention. | | | | | | | | | | | | | |  |
|  | | |  | | | | | | | | | | | | | |  |
|  | | | **Codes\\THPP delivery experience\Elements\Easy to deliver** | | | | | | | | | | | | | |  |
|  |  |  |  |  | No |  | 0.0699 |  | 3 |  | | | | | | |  |
|  | | |  |  |  |  |  |  |  |  | | | | | | | |
|  | | | | | | | | | | | | 1 |  | MTK |  | 7/4/2021 3:07 PM |  |
|  | | | I: I would like to know how these women managed to work on the assignments given to them were they managing to the assignments in time?  R: They were able to do the assignments because were eager to learn more and we could help them look into the pictures and give support and they took it as a great plessure to be in the programme. | | | | | | | | | | | | | |  |
|  | | |  | | | | | | | | | | | | | |  |
|  | | | | | | | | | | | | 2 |  | MTK |  | 7/4/2021 3:10 PM |  |
|  | | | I: What do you think were the easy ways to be learnt or done?  R: These were easy to be done because of the materials we were given from the hospital especially pictures, after getting them we studied, understood them and was easy to teach the women through pictures. It was easy to teach, even those who did not go to school got it, even the old mother in-laws that haven’t been to school were able to understand lessons through the pictures. The pictures could tell the mood like happy, angry, and sad so that made our job easier…… | | | | | | | | | | | | | |  |
|  | | |  | | | | | | | | | | | | | |  |
| Formatted Reports\\Coding Summary by File Formatted Report | | | | | | | | | | | Page 28 of 53 | | | | | | |
| 7/5/2021 9:43 AM | | | | | | | | | | | | | | | | | |
|  | | | **Classification** |  | **Aggregate** |  | **Coverage** |  | **Number Of Coding References** |  | | **Reference Number** |  | **Coded By Initials** |  | **Modified On** |  |
|  | | | | | | | | | | | | | | | | | |
|  | | | | | | | | | | | | 3 |  | MTK |  | 7/4/2021 3:14 PM |  |
|  | | | I: Thank you, now I would like to know what part was not difficult for you to deliver and what part was difficult and if there was something that you failed to deliver to the women?  R: The easy part was providing counselling while partner was present, especially if their relationship was good, for he took part in helping the woman to make her decision…. what was difficult was when we were dealing with women who had no partners. For example, I was visiting one woman who was alone, the husband rejected her and the baby because he said he was not the father of this baby. This was difficult for us to involve him in the intervention. | | | | | | | | | | | | | |  |
|  | | |  | | | | | | | | | | | | | |  |
|  | | | **Codes\\THPP delivery experience\Elements\Facilitators** | | | | | | | | | | | | | |  |
|  |  |  |  |  | No |  | 0.0890 |  | 4 |  | | | | | | |  |
|  | | |  |  |  |  |  |  |  |  | | | | | | | |
|  | | | | | | | | | | | | 1 |  | MTK |  | 7/4/2021 2:59 PM |  |
|  | | | I: Ok for those in the programme, were there any challenges in giving the service?  R: No, there were no problems because they welcomed it very well according to the problems found and had passion to change their lives. We were also seeing that the first time they were not open enough but the second time were relaxed and we could tell though it is counselling only without pills but the person is getting better. | | | | | | | | | | | | | |  |
|  | | |  | | | | | | | | | | | | | |  |
|  | | | | | | | | | | | | 2 |  | MTK |  | 7/4/2021 3:00 PM |  |
|  | | | I: What about in terms of time for you in giving help, did you see that it was okay or had some problems?  R: It was alright for we kept time for visits as trained like we should be finishing but they still wanted to continue the discussions.  I: So they still wanted you to continue?  R: Yes they asked if we can continue because of excitement with the program. But I said we are done for the day. | | | | | | | | | | | | | |  |
|  | | |  | | | | | | | | | | | | | |  |
|  | | | | | | | | | | | | 3 |  | MTK |  | 7/4/2021 3:06 PM |  |
|  | | | I: Thank you, let us talk about the woman you visited were they interested in the intervention, considering that you came long way to visit her?  R: Since the woman was referred to us by the doctor from hospital, she was told that a volunteer counselor will be visiting you at your home and upon arrival we were warmly welcomed. There were no problems from the women.  I: Ok so they had passion to receive counselling?  R: They received the program well that when we reached the end we could not tell them that we have stopped to avoid being upset instead we said for now let me stop here will continue next time. The programme was well received even by the chiefs around the community also welcomed this programme. | | | | | | | | | | | | | |  |
|  | | |  |  |  |  |  |  |  |  |  |  |  |  |  |  |  |
|  | | |  | | | | | | | | | | | | | |  |
|  | | | | | | | | | | | | 4 |  | MTK |  | 7/4/2021 3:07 PM |  |
|  | | | I: How was your experience regarding time keeping during the discussions, were they mobile or stayed to the end of the session?  R: Frankly speaking these women were listening attentively because it was the first time to have such a programme in our area even around Kabudula hospital so they wanted to really understand and benefit from the information that was being given to them. | | | | | | | | | | | | | |  |
|  | | |  | | | | | | | | | | | | | |  |
|  | | | **Codes\\THPP training\Content** | | | | | | | | | | | | | |  |
|  |  |  |  |  | No |  | 0.0208 |  | 1 |  | | | | | | |  |
|  | | |  |  |  |  |  |  |  |  | | | | | | | |
|  | | | | | | | | | | | | 1 |  | MTK |  | 7/4/2021 2:57 PM |  |
|  | | | I: Thank you. Let us talk about the study which you are taking part in now, I would like to know if you had training before commencement of the study to be equipped in conducting the duties?  R: Yes, we had a training although the days were not enough as compared to the work to be done. but in the study, it was found that we and the trainees were moving together until we got used to the intervention and we were able to share with them properly. | | | | | | | | | | | | | |  |
|  | | |  | | | | | | | | | | | | | |  |
|  | | | | | | | | | | | | | | | | | |
| Formatted Reports\\Coding Summary by File Formatted Report | | | | | | | | | | | Page 29 of 53 | | | | | | |
| 7/5/2021 9:43 AM | | | | | | | | | | | | | | | | | |
|  | | | **Classification** |  | **Aggregate** |  | **Coverage** |  | **Number Of Coding References** |  | | **Reference Number** |  | **Coded By Initials** |  | **Modified On** |  |
|  | | | **Codes\\THPP training\Duration** | | | | | | | | | | | | | |  |
|  |  |  |  |  | No |  | 0.0541 |  | 2 |  | | | | | | |  |
|  | | |  |  |  |  |  |  |  |  | | | | | | | |
|  | | | | | | | | | | | | 1 |  | MTK |  | 7/4/2021 2:57 PM |  |
|  | | | I: Thank you. Let us talk about the study which you are taking part in now, I would like to know if you had training before commencement of the study to be equipped in conducting the duties?  R: Yes, we had a training although the days were not enough as compared to the work to be done. but in the study, it was found that we and the trainees were moving together until we got used to the intervention and we were able to share with them properly. | | | | | | | | | | | | | |  |
|  | | |  | | | | | | | | | | | | | |  |
|  | | | | | | | | | | | | 2 |  | MTK |  | 7/4/2021 2:57 PM |  |
|  | | | I: So, you mean the training duration was short or …. Can you explain what happened?  R: The days were not enough, few training days so it was difficult to connect what we were learning, time was not enough. The training was done in our community, we were operating from our homes during the cultivation time so after the training we would just drop the papers and go back to the fields or doing other chores and early in the morning browse a little bit before going back to the training without having enough time to go through the books on what we learnt but later we were able to catch up. Had it been the training was a bit longer and done somewhere else with added time it would have been nice in relation to the job we were doing. | | | | | | | | | | | | | |  |
|  | | |  | | | | | | | | | | | | | |  |
|  | | | **Codes\\THPP training\Preparedness** | | | | | | | | | | | | | |  |
|  |  |  |  |  | No |  | 0.0049 |  | 1 |  | | | | | | |  |
|  | | |  |  |  |  |  |  |  |  | | | | | | | |
|  | | | | | | | | | | | | 1 |  | MTK |  | 7/4/2021 2:58 PM |  |
|  | | | I: What can you say about the training you had, was it important?  R: Yes, it is very important and useful. | | | | | | | | | | | | | |  |
|  | | |  | | | | | | | | | | | | | |  |
|  | **Files\\THPP PV_#07** | | | | | | | | | | | | | | | |  |
|  | | **Code** | | | | | | | | | | | | | | |  |
|  | | | **Codes\\Health workers\Relationship** | | | | | | | | | | | | | |  |
|  |  |  |  |  | No |  | 0.0325 |  | 1 |  | | | | | | |  |
|  | | |  |  |  |  |  |  |  |  | | | | | | | |
|  | | | | | | | | | | | | 1 |  | MTK |  | 7/4/2021 3:33 PM |  |
|  | | | I: Now, let us look at another section, you worked like a mediator between the woman and the health workers. I would like to know how your cooperation with health workers was.  R: Our cooperation with health workers was very good, because when the woman is not feeling well, we referred her to the doctors and we followed up on the next visit to inquire if she went to the hospital and how she is feeling at that time.  I: So, you can confirm that the relationship with the doctors/hospital staff was good that there is no time the woman received care that was different from the doctors?  R: We have a good relationship with hospital staff. | | | | | | | | | | | | | |  |
|  | | |  |  |  |  |  |  |  |  |  |  |  |  |  |  |  |
|  | | |  | | | | | | | | | | | | | |  |
|  | | | | | | | | | | | | | | | | | |
| Formatted Reports\\Coding Summary by File Formatted Report | | | | | | | | | | | Page 30 of 53 | | | | | | |
| 7/5/2021 9:43 AM | | | | | | | | | | | | | | | | | |
|  | | | **Classification** |  | **Aggregate** |  | **Coverage** |  | **Number Of Coding References** |  | | **Reference Number** |  | **Coded By Initials** |  | **Modified On** |  |
|  | | | **Codes\\Involvement of family members** | | | | | | | | | | | | | |  |
|  |  |  |  |  | No |  | 0.1014 |  | 3 |  | | | | | | |  |
|  | | |  |  |  |  |  |  |  |  | | | | | | | |
|  | | | | | | | | | | | | 1 |  | MTK |  | 7/4/2021 3:29 PM |  |
|  | | | I: Let us now talk about the woman you visited. Upon reaching the woman’s home, you said that sometimes the husband and other relatives participated?  R: Yes  I: Tell me what used to happen?  R: During the first session where we were laying the foundation of the programme, we could tell the woman the purpose of our visit and the aim of the programme even if the husband or other relatives were not around, since it was the first visit but we still emphasized that this programme encourages involvement of other family members especially the husband. So that they should be aware of the programme but also what is in this programme need to be known by the whole family so they can be able to support the woman through the problem she meets. | | | | | | | | | | | | | |  |
|  | | |  |  |  |  |  |  |  |  |  |  |  |  |  |  |  |
|  | | |  | | | | | | | | | | | | | |  |
|  | | | | | | | | | | | | 2 |  | MTK |  | 7/4/2021 3:31 PM |  |
|  | | | I: Ok, can you tell me how comfortable was the woman when she was receiving the intervention together with the husband and other family members to express her feelings and also what part did the mother-in-law or other family members take?  R: The woman was relaxed as she was told that everything in the programme will be kept confidential and that we will not share their concerns/issues with other people apart from these that are part of the intervention. We even emphasized that with all family members involved. Sometimes we observed that the man could help the woman talk about issues that she is not disclosing or when she was not open enough. | | | | | | | | | | | | | |  |
|  | | |  | | | | | | | | | | | | | |  |
|  | | | | | | | | | | | | 3 |  | MTK |  | 7/4/2021 3:34 PM |  |
|  | | | I: If I get you clearly, you are saying the programme has also benefited other women, your friends apart from those enrolled in the programme?  R: Yes, they have been assisted, because when they complain I tell them not to be bothered by issues that will not help them. There was one woman who found me at home, she was not even pregnant, when she told me about her problems, I talked her through the unhelpful thoughts and behavior that we learned versus helpful thoughts and behavior. I encouraged her to change her way of thinking ……. she understood and even now she appreciates that she was helped. | | | | | | | | | | | | | |  |
|  | | |  | | | | | | | | | | | | | |  |
|  | | | **Codes\\Personal history** | | | | | | | | | | | | | |  |
|  |  |  |  |  | No |  | 0.0442 |  | 2 |  | | | | | | |  |
|  | | |  |  |  |  |  |  |  |  | | | | | | | |
|  | | | | | | | | | | | | 1 |  | MTK |  | 7/4/2021 3:21 PM |  |
|  | | | I: Ok can you tell me about your marriage, whether your husband has other wives besides you?  R: This is my first marriage with my husband, we have three children two girls and one boy. | | | | | | | | | | | | | |  |
|  | | |  | | | | | | | | | | | | | |  |
|  | | | | | | | | | | | | 2 |  | MTK |  | 7/4/2021 3:22 PM |  |
|  | | | I: Have you stopped bearing children or you plan to have some in future?  R: I have not stopped only that I use family planning methods, if possible in future I will have another.  I: You have not discussed on the number of children to have?  R: Yes… (Laughter) | | | | | | | | | | | | | |  |
|  | | |  |  |  |  |  |  |  |  |  |  |  |  |  |  |  |
|  | | |  | | | | | | | | | | | | | |  |
|  | | | | | | | | | | | | | | | | | |
| Formatted Reports\\Coding Summary by File Formatted Report | | | | | | | | | | | Page 31 of 53 | | | | | | |
| 7/5/2021 9:43 AM | | | | | | | | | | | | | | | | | |
|  | | | **Classification** |  | **Aggregate** |  | **Coverage** |  | **Number Of Coding References** |  | | **Reference Number** |  | **Coded By Initials** |  | **Modified On** |  |
|  | | | **Codes\\Personal history\Age** | | | | | | | | | | | | | |  |
|  |  |  |  |  | No |  | 0.0373 |  | 1 |  | | | | | | |  |
|  | | |  |  |  |  |  |  |  |  | | | | | | | |
|  | | | | | | | | | | | | 1 |  | MTK |  | 7/4/2021 3:19 PM |  |
|  | | | I: Thank you, so as already said feel free. Our discussion will be based on what I have explained, this is a confidential discussion, your opinions will be used for study purposes only, so feel free to tell us your experiences this morning. Our discussion will be in three sections, the first section is about your demographic background and the two sections will be about this study. Starting with the first section I would like to know how old you are now?  R: Thank you, I was born in 1984 on 26 December and I am 34 years old.  I: So you were born in 1984 and you are 34?  R: Yes  I: Ok may be if we can do some calculations, you should be 36 or37……  R: I think 36 years will turn 37 this December (Laughter) I just missed a bit. | | | | | | | | | | | | | |  |
|  | | |  |  |  |  |  |  |  |  |  |  |  |  |  |  |  |
|  | | |  | | | | | | | | | | | | | |  |
|  | | | **Codes\\Personal history\Contact information** | | | | | | | | | | | | | |  |
|  |  |  |  |  | No |  | 0.0114 |  | 1 |  | | | | | | |  |
|  | | |  |  |  |  |  |  |  |  | | | | | | | |
|  | | | | | | | | | | | | 1 |  | MTK |  | 7/4/2021 3:23 PM |  |
|  | | | I: Ok do you have postal address which you use?  R: No  I: Ok I would like to know if you have a phone.  R: Yes I have a phone  I: Can you tell your number by heart?  R: Yes I can say it. ……….. | | | | | | | | | | | | | |  |
|  | | |  | | | | | | | | | | | | | |  |
|  | | | **Codes\\Personal history\Level of education** | | | | | | | | | | | | | |  |
|  |  |  |  |  | No |  | 0.0144 |  | 1 |  | | | | | | |  |
|  | | |  |  |  |  |  |  |  |  | | | | | | | |
|  | | | | | | | | | | | | 1 |  | MTK |  | 7/4/2021 3:19 PM |  |
|  | | | I: Ok can you tell me about your education?  R:I did my primary education from standard 1 up to 8 then went to secondary school up to form 2.  I: Were there any reasons why you stopped in form 2?  R: I stopped in form 2 because my parents could no longer afford paying my school fees. | | | | | | | | | | | | | |  |
|  | | |  | | | | | | | | | | | | | |  |
|  | | | **Codes\\Personal history\Location** | | | | | | | | | | | | | |  |
|  |  |  |  |  | No |  | 0.0057 |  | 1 |  | | | | | | |  |
|  | | |  |  |  |  |  |  |  |  | | | | | | | |
|  | | | | | | | | | | | | 1 |  | MTK |  | 7/4/2021 3:22 PM |  |
|  | | | I: Laughter…ok thank you, I would like to know where you stay now?  R: Now I stay in ………….. village T/A Kabudula. | | | | | | | | | | | | | |  |
|  | | |  | | | | | | | | | | | | | |  |
|  | | | **Codes\\Personal history\Marital status** | | | | | | | | | | | | | |  |
|  |  |  |  |  | No |  | 0.0026 |  | 1 |  | | | | | | |  |
|  | | |  |  |  |  |  |  |  |  | | | | | | | |
|  | | | | | | | | | | | | 1 |  | MTK |  | 7/4/2021 3:19 PM |  |
|  | | | I: Thank you. Are you married?  R: Yes I am married. | | | | | | | | | | | | | |  |
|  | | |  | | | | | | | | | | | | | |  |
| Formatted Reports\\Coding Summary by File Formatted Report | | | | | | | | | | | Page 32 of 53 | | | | | | |
| 7/5/2021 9:43 AM | | | | | | | | | | | | | | | | | |
|  | | | **Classification** |  | **Aggregate** |  | **Coverage** |  | **Number Of Coding References** |  | | **Reference Number** |  | **Coded By Initials** |  | **Modified On** |  |
|  | | | **Codes\\Personal history\Number of children** | | | | | | | | | | | | | |  |
|  |  |  |  |  | No |  | 0.0063 |  | 1 |  | | | | | | |  |
|  | | |  |  |  |  |  |  |  |  | | | | | | | |
|  | | | | | | | | | | | | 1 |  | MTK |  | 7/4/2021 3:20 PM |  |
|  | | | I: Thank you. You said you have three children, are these the only child you gave birth to?  R: Yes I gave birth three times. | | | | | | | | | | | | | |  |
|  | | |  | | | | | | | | | | | | | |  |
|  | | | **Codes\\Previous work experience\Volunteer experience** | | | | | | | | | | | | | |  |
|  |  |  |  |  | No |  | 0.0383 |  | 1 |  | | | | | | |  |
|  | | |  |  |  |  |  |  |  |  | | | | | | | |
|  | | | | | | | | | | | | 1 |  | MTK |  | 7/4/2021 3:25 PM |  |
|  | | | I: In your previous voluntary work what has been your experience?  R: I sometimes had challenges in doing my job other people would say that I am just moving around but I was not discouraged, I jus want to do the job am assigned.  I: What did they mean by ‘moving around’?  R: They said I have started prostitution but I paid a deaf ear to this and keep on working as a volunteer.  I: So they thought that when going for home visits you were meeting other men?  R: Yes and others were jealousy of me they wanted if it were them doing the job.  I: So meaning they were jealousy?  R: Yes they were jealousy.  I: Is this still happening during this voluntary work?  R: No it stopped because the chief talked about it, that they choose those who are hard working. | | | | | | | | | | | | | |  |
|  | | |  |  |  |  |  |  |  |  |  |  |  |  |  |  |  |
|  | | |  | | | | | | | | | | | | | |  |
|  | | | **Codes\\Previous work experience\Volunteer experience\Name of organization** | | | | | | | | | | | | | |  |
|  |  |  |  |  | No |  | 0.0171 |  | 1 |  | | | | | | |  |
|  | | |  |  |  |  |  |  |  |  | | | | | | | |
|  | | | | | | | | | | | | 1 |  | MTK |  | 7/4/2021 3:23 PM |  |
|  | | | I: Ok I can see that you memorized it. We are going to the second section of our discussion; in this part I would like to know if you have worked as a health volunteer before this study?  R: I worked as a volunteer and I am a promoter of world relief (chiyambi chabwino), I am also a village livestock advisor trained by Concern worldwide. | | | | | | | | | | | | | |  |
|  | | |  | | | | | | | | | | | | | |  |
|  | | | **Codes\\Recommendations** | | | | | | | | | | | | | |  |
|  |  |  |  |  | No |  | 0.2133 |  | 3 |  | | | | | | |  |
|  | | |  |  |  |  |  |  |  |  | | | | | | | |
|  | | | | | | | | | | | | 1 |  | MTK |  | 7/4/2021 3:38 PM |  |
|  | | | I: Ok thank you very much, now I would like to ask you as a counsellor providing the intervention from the time of training up to the completion of counselling services which areas do you think need to be reviewed in this programme?  R: Thank you very much the part that needs to be reviewed, firstly is training if the programme is to continue there is need for additional training so that we can internalize everything without reading from books when delivering sessions. The second thing is the number of women, it needs to be increased. Most people wanted to be part of the programme. They were complaining, that they were not selected but we just reassured them that since this is the first time, we are implementing this programme, we couldn’t include every pregnant woman, they might be included in future if this programme will continue. | | | | | | | | | | | | | |  |
|  | | |  |  |  |  |  |  |  |  |  |  |  |  |  |  |  |
|  | | |  | | | | | | | | | | | | | |  |
|  | | | | | | | | | | | | | | | | | |
| Formatted Reports\\Coding Summary by File Formatted Report | | | | | | | | | | | Page 33 of 53 | | | | | | |
| 7/5/2021 9:43 AM | | | | | | | | | | | | | | | | | |
|  | | | **Classification** |  | **Aggregate** |  | **Coverage** |  | **Number Of Coding References** |  | | **Reference Number** |  | **Coded By Initials** |  | **Modified On** |  |
|  | | | | | | | | | | | | | | | | | |
|  | | | | | | | | | | | | 2 |  | MTK |  | 7/4/2021 3:40 PM |  |
|  | | | I: Ok you said there is need for additional training, how can this be done?  R: If we can have additional training and also if there can be enough books and the health calendar so that women can mark what they have eaten, the time she had for rest or how she felt that week. That is why a phone is needed so that we can use for the sessions. The health calendar to be given to the woman for reference then the counselor should use phone.  I: You talked about the phone several times…….  R: Yes it is needed so much, we admire our friends (Laughter)  I: Do you just want it or it is needed?  R: (Laughter) I want to use it for the programme because I have mine but this programme needs a phone.  I: You also wanted the materials to be added like books and calendars….. What can be the other things?  R: The other thing we want to be assisted on is transportation, because sometimes we were walking on foot if we weren’t able to find bicycle taxis, or in times when our bicycle had a breakdown. They should consider us in this area if possible.  I: Ok what about the materials used in providing counselling to the women, is there anything to be added?  R: May be if they can add the health calendars for women to keep because I was carrying the calendars for the four women, it was difficult to use at their free time since I couldn’t leave it with the women. instead, I was moving with it and mark the woman’s mood and what she managed to do during the session, so it was difficult to remember what was done for the whole week.  I: Thank you now we are approaching the end of our discussion, is there anything that you would want to talk about, something that I might have forgotten to ask or something you think can help to improve the intervention?  R: This study is good and has helped us and the women we were visiting, although there were few. My request is that this programme should continue so that more women are assisted in our community. Because there are a lot of pregnant women with problems and only few were involved in this programme. So, I wish these women were considered, more women should be involved maybe we can change lives of pregnant women who have a lot of emotional problems in pregnancy. We can even help to reduce maternal deaths and still births. Even reducing high blood pressure that affect a lot of women when they are about to deliver. This programme has also benefited us as counselors. I, as a counselor who was visiting the women. I have benefitted a lot.  I ask for the continuity of the programme to benefit a lot of the people in the community pregnant women and those with health problems are many for this targeted only a few and if they can look into this and help them have helpful thinking because if someone is pregnant usually has a lot of stress and this can prevent maternal deaths, miscarriages and high blood pressure………I have also benefited from the programme as a counsellor providing this service. | | | | | | | | | | | | | |  |
|  | | |  |  |  |  |  |  |  |  |  |  |  |  |  |  |  |
|  | | |  |  |  |  |  |  |  |  |  |  |  |  |  |  |  |
|  | | |  |  |  |  |  |  |  |  |  |  |  |  |  |  |  |
|  | | |  | | | | | | | | | | | | | |  |
|  | | | | | | | | | | | | 3 |  | MTK |  | 7/4/2021 3:41 PM |  |
|  | | | R: I want to thank you for taking part in this intervention and although this programme is coming to an end but the skills you acquired can still be used, am I right?  I: Yes you are right.  R: So you will still be helping people even when the study has reached the end?  I: Yes will be still helping people, when a woman approaches me will be helped because they know that I was doing the job….. (Phone rings) | | | | | | | | | | | | | |  |
|  | | |  | | | | | | | | | | | | | |  |
|  | | | **Codes\\THPP delivery experience** | | | | | | | | | | | | | |  |
|  |  |  |  |  | No |  | 0.0463 |  | 1 |  | | | | | | |  |
|  | | |  |  |  |  |  |  |  |  | | | | | | | |
|  | | | | | | | | | | | | 1 |  | MTK |  | 7/4/2021 3:34 PM |  |
|  | | | I: Ok we are continuing with our discussion, tell me more on your experiences as a counsellor delivering the intervention. Firstly I would like to know what you have gone through in providing this thinking health counselling to the women.  R: I have also learnt a lot in the time I was providing counselling to these women because some problems these women had I also experienced them. But now each time I go through some stressful situation am able to help myself deal with the issue and do things that will help me and my family. I have benefited a lot  I: So, you would like to say that the counselling helped you besides helping others?  R: Yes, the counselling has helped me whilst I was helping others because I also had emotional problems and doing unhelpful things. Even when some of my friends came to complain to me am able to direct them in thinking healthy, to focus on the things that will help them. | | | | | | | | | | | | | |  |
|  | | |  |  |  |  |  |  |  |  |  |  |  |  |  |  |  |
|  | | |  | | | | | | | | | | | | | |  |
|  | | | | | | | | | | | | | | | | | |
| Formatted Reports\\Coding Summary by File Formatted Report | | | | | | | | | | | Page 34 of 53 | | | | | | |
| 7/5/2021 9:43 AM | | | | | | | | | | | | | | | | | |
|  | | | **Classification** |  | **Aggregate** |  | **Coverage** |  | **Number Of Coding References** |  | | **Reference Number** |  | **Coded By Initials** |  | **Modified On** |  |
|  | | | **Codes\\THPP delivery experience\Elements** | | | | | | | | | | | | | |  |
|  |  |  |  |  | No |  | 0.0334 |  | 2 |  | | | | | | |  |
|  | | |  |  |  |  |  |  |  |  | | | | | | | |
|  | | | | | | | | | | | | 1 |  | MTK |  | 7/4/2021 3:28 PM |  |
|  | | | I: How much time were you spending with the women?  R: We were meeting for one hour.  I: Can you explain what happened during that one hour was it enough or more than enough?  R: The time was enough in relation to the topic for that day, we were able to finish the discussions, ask some questions then bid farewell, it was enough. | | | | | | | | | | | | | |  |
|  | | |  | | | | | | | | | | | | | |  |
|  | | | | | | | | | | | | 2 |  | MTK |  | 7/4/2021 3:37 PM |  |
|  | | | I: Is there any part that you see went well like the training or may be what you feel did not go well?  R: On the part of training and delivering the intervention, I think I did very well, I was able to provide counseling to the people I was visiting. The people I was visiting are happy with the intervention they were provided. | | | | | | | | | | | | | |  |
|  | | |  | | | | | | | | | | | | | |  |
|  | | | **Codes\\THPP delivery experience\Elements\Barriers** | | | | | | | | | | | | | |  |
|  |  |  |  |  | No |  | 0.0272 |  | 1 |  | | | | | | |  |
|  | | |  |  |  |  |  |  |  |  | | | | | | | |
|  | | | | | | | | | | | | 1 |  | MTK |  | 7/4/2021 3:29 PM |  |
|  | | | I: Ok what about the resources given to you as people providing counselling, was it enough or you lacked something to help you provide the service?  R: The resources we received were not enough because we were walking long distances and sometimes, we used to hire bicycle and we were charged different prices. Also, other materials like books used in counselling, books only were not enough, we needed things like phones, these days people use phones to store information, most things are done through phone before going for a home visit. | | | | | | | | | | | | | |  |
|  | | |  | | | | | | | | | | | | | |  |
|  | | | **Codes\\THPP delivery experience\Elements\Challenges** | | | | | | | | | | | | | |  |
|  |  |  |  |  | No |  | 0.0392 |  | 3 |  | | | | | | |  |
|  | | |  |  |  |  |  |  |  |  | | | | | | | |
|  | | | | | | | | | | | | 1 |  | MTK |  | 7/4/2021 3:27 PM |  |
|  | | | I: If you say that they were surprised at the first time, why was it so did they not expect you to visit them?  R: They knew but when we visited for the first time to lay the foundation for the programme they were not sure of what we will be discussing with them going forward. | | | | | | | | | | | | | |  |
|  | | |  | | | | | | | | | | | | | |  |
|  | | | | | | | | | | | | 2 |  | MTK |  | 7/4/2021 3:28 PM |  |
|  | | | I: Ok I want you to explain more on this that how difficult was it to provide counselling if at all they were challenges.  R: It was difficult to make a person understand what we meant because they heard that they will be visited and have lessons but did not know how exactly this will be done and others thought that if someone visits you from hospital it means you have been found with some disease but in our study, it was not like this. | | | | | | | | | | | | | |  |
|  | | |  | | | | | | | | | | | | | |  |
|  | | | | | | | | | | | | 3 |  | MTK |  | 7/4/2021 3:36 PM |  |
|  | | | I: Were there any challenge?  R: No there was nothing. | | | | | | | | | | | | | |  |
|  | | |  | | | | | | | | | | | | | |  |
|  | | | **Codes\\THPP delivery experience\Elements\Easy to deliver** | | | | | | | | | | | | | |  |
|  |  |  |  |  | No |  | 0.0914 |  | 3 |  | | | | | | |  |
|  | | |  |  |  |  |  |  |  |  | | | | | | | |
|  | | | | | | | | | | | | 1 |  | MTK |  | 7/4/2021 3:32 PM |  |
|  | | | I: Thank you. I want to know about materials that you were given to provide counselling, were they enough and how did they help you?  R: The materials were helping a lot. We were given books, health calendars that the women could record how they have been, how they were feeling that week and even recording home work. Women would record the type of food they managed to eat, rest etc. But I think the materials were not enough. There were difficulties in carrying the books during rainy season. The health calendar was given to a woman while we use a book, had it been we used a phone, people would not know that we are reading from a book instead they would think we are getting all the information from the head. | | | | | | | | | | | | | |  |
|  | | |  | | | | | | | | | | | | | |  |
| Formatted Reports\\Coding Summary by File Formatted Report | | | | | | | | | | | Page 35 of 53 | | | | | | |
| 7/5/2021 9:43 AM | | | | | | | | | | | | | | | | | |
|  | | | **Classification** |  | **Aggregate** |  | **Coverage** |  | **Number Of Coding References** |  | | **Reference Number** |  | **Coded By Initials** |  | **Modified On** |  |
|  | | | | | | | | | | | | | | | | | |
|  | | | | | | | | | | | | 2 |  | MTK |  | 7/4/2021 3:35 PM |  |
|  | | | I: Thank you, now I would like to know the areas that were easy to learn and deliver/do in the intervention?  R: Thank you what I saw that was easy to be done is behavior change from unhelpful behavior to helpful behavior but also changing someone’s mind or the way of thinking, I found that easier to do in this programme. | | | | | | | | | | | | | |  |
|  | | |  | | | | | | | | | | | | | |  |
|  | | | | | | | | | | | | 3 |  | MTK |  | 7/4/2021 3:37 PM |  |
|  | | | I: From all the sessions, session I to 10 given to the woman, which were the easiest to deliver, if you can evaluate yourself?  R: The lesson that at first had problems with was on woman’s health because it was first time meeting the woman on my own, I had problems to interpreted the session and explain well to the woman, but in the middle of the session I got used.  I: What were the easy lessons for you to teach?  R: Session numbers 2, 3 and 4 to the pregnant woman and when the woman had delivered session 5, 6 and 7 were easy because they were similar, we were repeating the same process the only difference was that in the first four sessions you discuss the topics when woman is still pregnant and later you focus on her and the baby after delivery. | | | | | | | | | | | | | |  |
|  | | |  |  |  |  |  |  |  |  |  |  |  |  |  |  |  |
|  | | |  | | | | | | | | | | | | | |  |
|  | | | **Codes\\THPP delivery experience\Elements\Facilitators** | | | | | | | | | | | | | |  |
|  |  |  |  |  | No |  | 0.1549 |  | 6 |  | | | | | | |  |
|  | | |  |  |  |  |  |  |  |  | | | | | | | |
|  | | | | | | | | | | | | 1 |  | MTK |  | 7/4/2021 3:27 PM |  |
|  | | | I: Now, as you were taking these counselling services to the women through home visits, I would like to know what you have gone through.  R: some of the experiences we had is that when going for the first time some women were surprised/not sure of what the programme was about but from the second visit things were different especially since we were encouraging other family members to be involved especially the husband and that improved their understanding of the programme. They discovered that this programme was very helpful even now they still invite us to go and visit them | | | | | | | | | | | | | |  |
|  | | |  | | | | | | | | | | | | | |  |
|  | | | | | | | | | | | | 2 |  | MTK |  | 7/4/2021 3:31 PM |  |
|  | | | I: Thank you. I want to know about materials that you were given to provide counselling, were they enough and how did they help you?  R: The materials were helping a lot. We were given books, health calendars that the women could record how they have been, how they were feeling that week and even recording home work. Women would record the type of food they managed to eat, rest etc. But I think the materials were not enough. There were difficulties in carrying the books during rainy season. The health calendar was given to a woman while we use a book, had it been we used a phone, people would not know that we are reading from a book instead they would think we are getting all the information from the head. | | | | | | | | | | | | | |  |
|  | | |  | | | | | | | | | | | | | |  |
|  | | | | | | | | | | | | 3 |  | MTK |  | 7/4/2021 3:32 PM |  |
|  | | | I: Ok so I would like to know, the interest the women had in the intervention, when visited did they have interest to get counselling from you as their counsellor?  R: These women had interest and we developed a relationship through this programme and i had nice welcome each time I visited them even now when we meet, they give us a lot of respect.  I: So, you can strongly say that your relationship is very good?  R: Yes our relationship is very good that each time after a session, they would want to walk us out/ escort us. | | | | | | | | | | | | | |  |
|  | | |  | | | | | | | | | | | | | |  |
|  | | | | | | | | | | | | 4 |  | MTK |  | 7/4/2021 3:33 PM |  |
|  | | | I: OK I would like to know what used to happened when you were conducting a session, were these women present throughout the session or they would go to do other things and join later. Were they active during the entire one hour session?  R: They were dedicating the agreed time for the sessions; other household chores were done after counselling sessions. | | | | | | | | | | | | | |  |
|  | | |  | | | | | | | | | | | | | |  |
|  | | | | | | | | | | | | 5 |  | MTK |  | 7/4/2021 3:35 PM |  |
|  | | | I: What can you say are the things that facilitated/helped you in delivering this intervention and what were the barriers?  R: Things that made the service to continue or fail?  I: Yes  R: I think the books helped us, It would have been difficult for us to teach if we were not given books because they trained us for few days and the books helped us in delivering counselling to the women when we forgot something we would refer to the books. | | | | | | | | | | | | | |  |
|  | | |  | | | | | | | | | | | | | |  |
|  | | | | | | | | | | | | 6 |  | MTK |  | 7/4/2021 3:36 PM |  |
|  | | | I: I thank you because you are answering openly, we are approaching the end of our discussion I would like to know which part of the programme went well and what part was not going well?  R: The part that is going well according to the programme is the transport money that is provided to us. It is somehow good although we cover long distances and sometimes it is not enough but we appreciate because it eases our movement. | | | | | | | | | | | | | |  |
|  | | |  | | | | | | | | | | | | | |  |
| Formatted Reports\\Coding Summary by File Formatted Report | | | | | | | | | | | Page 36 of 53 | | | | | | |
| 7/5/2021 9:43 AM | | | | | | | | | | | | | | | | | |
|  | | | **Classification** |  | **Aggregate** |  | **Coverage** |  | **Number Of Coding References** |  | | **Reference Number** |  | **Coded By Initials** |  | **Modified On** |  |
|  | | | **Codes\\THPP training\Duration** | | | | | | | | | | | | | |  |
|  |  |  |  |  | No |  | 0.0279 |  | 1 |  | | | | | | |  |
|  | | |  |  |  |  |  |  |  |  | | | | | | | |
|  | | | | | | | | | | | | 1 |  | MTK |  | 7/4/2021 3:25 PM |  |
|  | | | I: How can you describe the training, was it enough or lacked something?  R: According to me I feel the training was not enough because the training duration was short against the training content. I wish it was about two weeks or so. I also wish the training was done at a different place outside the community, where we would reside there during the period of training to concentrate on the training and prevent disturbances from home because when we were operating from home, it was difficult to go through what you learnt that day in the evenings. | | | | | | | | | | | | | |  |
|  | | |  | | | | | | | | | | | | | |  |
|  | | | **Codes\\THPP training\Preparedness** | | | | | | | | | | | | | |  |
|  |  |  |  |  | No |  | 0.0095 |  | 1 |  | | | | | | |  |
|  | | |  |  |  |  |  |  |  |  | | | | | | | |
|  | | | | | | | | | | | | 1 |  | MTK |  | 7/4/2021 3:26 PM |  |
|  | | | I: Okay, we will come back to that later. You said the days were not enough, but was the training important for your work?  R: Yes, the training was very useful and other people admire it. | | | | | | | | | | | | | |  |
|  | | |  | | | | | | | | | | | | | |  |
|  | **Files\\THPP PV_#2** | | | | | | | | | | | | | | | |  |
|  | | **Code** | | | | | | | | | | | | | | |  |
|  | | | **Codes\\Health workers\Relationship** | | | | | | | | | | | | | |  |
|  |  |  |  |  | No |  | 0.0315 |  | 1 |  | | | | | | |  |
|  | | |  |  |  |  |  |  |  |  | | | | | | | |
|  | | | | | | | | | | | | 1 |  | MTK |  | 7/1/2021 1:32 PM |  |
|  | | | I: Okay. To continue with the discussion, I would like to know; you were working with these women and at the same time you were working with health workers. How was your work with the health workers? What were the barriers and facilitators?  R: To say the truth, we were doing this work in two fold; working with the women and with the health workers. We have worked very well with the health workers and they were very understanding considering our different education levels. Where we were facing challenges, we were able to ask them and they were not tired to repeatedly assist us so that we understand and provide the quality service. We were in very good relationship with them and if there were some challenges, we are human beings and no one is 100% perfect. We are grateful that we had a good relationship and we worked very well, for other challenges we simply need to understand and forgive each other. | | | | | | | | | | | | | |  |
|  | | |  |  |  |  |  |  |  |  |  |  |  |  |  |  |  |
|  | | |  | | | | | | | | | | | | | |  |
|  | | | | | | | | | | | | | | | | | |
|  | | | | | | | | | | | | | | | | | |
| Formatted Reports\\Coding Summary by File Formatted Report | | | | | | | | | | | Page 37 of 53 | | | | | | |
| 7/5/2021 9:43 AM | | | | | | | | | | | | | | | | | |
|  | | | **Classification** |  | **Aggregate** |  | **Coverage** |  | **Number Of Coding References** |  | | **Reference Number** |  | **Coded By Initials** |  | **Modified On** |  |
|  | | | **Codes\\Involvement of family members** | | | | | | | | | | | | | |  |
|  |  |  |  |  | No |  | 0.1100 |  | 2 |  | | | | | | |  |
|  | | |  |  |  |  |  |  |  |  | | | | | | | |
|  | | | | | | | | | | | | 1 |  | MTK |  | 7/1/2021 1:27 PM |  |
|  | | | I: So how was the counseling session conducted? Were there other people available such as mother in-laws, husband and other family members. What was the reception like by the people surrounding the woman?  R: Because when we went there for the first time, we introduced the program that we are looking at the health of the woman, relationship between the woman and the child and the relationship between the woman and people around her. So this program was successful because we made sure that we have people around the woman available during the first session so that if they attend all the sessions, they should be able to know what is required of them to support the pregnant woman. The mother in-law should not think that the pregnancy is for her son and her daughter in-law but it is the responsibility of every person around the pregnant woman. In other homes, it was happening that mother in-laws were not able to come when invited to the session but the most interesting part is that other members of the family were able to attend. The other interesting thing is that it was happening that when we went to the participant’s home, we could find that she is not there maybe she went to fetch water but other family members or friends were able to warmly welcome us and gave us a place to sit while waiting for the participant. They could go to call the participant that her visitors have come. It was a strange and extraordinary intervention that I have never experienced in my life. | | | | | | | | | | | | | |  |
|  | | |  |  |  |  |  |  |  |  |  |  |  |  |  |  |  |
|  | | |  | | | | | | | | | | | | | |  |
|  | | | | | | | | | | | | 2 |  | MTK |  | 7/1/2021 1:28 PM |  |
|  | | | I: Regarding the partners, what roles did they play during the counseling interventions? Were they available for session? What were their views?  R: I had a very wonderful experience with one participant. At first, the woman thought that if she was to complain while her partner was present, it would not be a good thing. There was a time when we were discussing about her health and when I asked about her mood, how she was feelings, she just said that everything was okay. Then the partner jumped in and said “why are you responding like this? Do you mean to say there are no issues, that you don’t expect me to be buying fish or eggs for you to eat? These people have brought in a very good program and it is going to help improve our families, look at the way we have been living in this family before and now that this intervention is here. Please madame, whenever you come for a session, if you find that I am not available, I mostly spend my time at the dimba garden. Do not start the sessions until when I come. Send a child to call me. This is a very good program because my wife has been living unhappy life and with this intervention, I know things are going to improve.” So there was positive feedback from some of the partners.  However, there were some partners who seemed to be very busy and never attended the sessions, we were just having other family members present. For most of them, they were in polygamous marriages and they feared that the other partner may not be happy if she hears that the husband was involved in the counseling sessions with the other wife. We inquired indirectly about this from the communities and this is some of the information that we gathered. | | | | | | | | | | | | | |  |
|  | | |  |  |  |  |  |  |  |  |  |  |  |  |  |  |  |
|  | | |  |  |  |  |  |  |  |  |  |  |  |  |  |  |  |
|  | | |  | | | | | | | | | | | | | |  |
|  | | | **Codes\\Personal history** | | | | | | | | | | | | | |  |
|  |  |  |  |  | No |  | 0.0283 |  | 2 |  | | | | | | |  |
|  | | |  |  |  |  |  |  |  |  | | | | | | | |
|  | | | | | | | | | | | | 1 |  | MTK |  | 6/30/2021 11:05 PM |  |
|  | | | I: Can you describe the lost child?  R: I can say it was a miscarriage. | | | | | | | | | | | | | |  |
|  | | |  | | | | | | | | | | | | | |  |
|  | | | | | | | | | | | | 2 |  | MTK |  | 6/30/2021 11:05 PM |  |
|  | | | I: Thank you. Can you describe your marriage?  R: We are three in a polygamous family. I am in the middle because he left another one before me and he married another one after me.  I: So you are like the first wife now?  R: Yes.  I: Is this your first marriage?  R: It is my first marriage but at first I was impregnated and had the boy I am talking about and this one is the step son for my current husband. | | | | | | | | | | | | | |  |
|  | | |  |  |  |  |  |  |  |  |  |  |  |  |  |  |  |
|  | | |  | | | | | | | | | | | | | |  |
|  | | | | | | | | | | | | | | | | | |
| Formatted Reports\\Coding Summary by File Formatted Report | | | | | | | | | | | Page 38 of 53 | | | | | | |
| 7/5/2021 9:43 AM | | | | | | | | | | | | | | | | | |
|  | | | **Classification** |  | **Aggregate** |  | **Coverage** |  | **Number Of Coding References** |  | | **Reference Number** |  | **Coded By Initials** |  | **Modified On** |  |
|  | | | **Codes\\Personal history\Age** | | | | | | | | | | | | | |  |
|  |  |  |  |  | No |  | 0.0148 |  | 1 |  | | | | | | |  |
|  | | |  |  |  |  |  |  |  |  | | | | | | | |
|  | | | | | | | | | | | | 1 |  | MTK |  | 6/30/2021 11:03 PM |  |
|  | | | I: Thank you very much. Our discussion will cover three sections. The first part is about your demographic information, the remaining two parts are about the study in which you have been taking part. So to start with, how old are you? If you are free you can tell us the date and month of your birthday.  R: I was born in 1976 on 22 July.  I: Thank you very much. How old do you think you are now?  R: I believe I am 45 years old. | | | | | | | | | | | | | |  |
|  | | |  | | | | | | | | | | | | | |  |
|  | | | **Codes\\Personal history\Contact information** | | | | | | | | | | | | | |  |
|  |  |  |  |  | No |  | 0.0078 |  | 1 |  | | | | | | |  |
|  | | |  |  |  |  |  |  |  |  | | | | | | | |
|  | | | | | | | | | | | | 1 |  | MTK |  | 6/30/2021 11:06 PM |  |
|  | | | I: Are you comfortable to tell me your address?  R: Yes. Kabudula FP School, P.O. Box 20, Nsaru, Lilongwe.  I: Do you have a phone number, and if you have what is your phone number?  R: I have a phone. The number is …………. | | | | | | | | | | | | | |  |
|  | | |  | | | | | | | | | | | | | |  |
|  | | | **Codes\\Personal history\Level of education** | | | | | | | | | | | | | |  |
|  |  |  |  |  | No |  | 0.0119 |  | 1 |  | | | | | | |  |
|  | | |  |  |  |  |  |  |  |  | | | | | | | |
|  | | | | | | | | | | | | 1 |  | MTK |  | 6/30/2021 11:03 PM |  |
|  | | | I: Thank you very much. How far did you go with your education?  R: I only did Junior Certificate of Education. I was not able to continue further because of lack of school fees.  I: So you would have wished to continue?  R: Very much so and up to now, I am not happy when I see my friends whom I was with at school who completed their education. | | | | | | | | | | | | | |  |
|  | | |  | | | | | | | | | | | | | |  |
|  | | | **Codes\\Personal history\Location** | | | | | | | | | | | | | |  |
|  |  |  |  |  | No |  | 0.0022 |  | 1 |  | | | | | | |  |
|  | | |  |  |  |  |  |  |  |  | | | | | | | |
|  | | | | | | | | | | | | 1 |  | MTK |  | 6/30/2021 11:06 PM |  |
|  | | | I: Where do you stay?  R: I stay in ………….. village in TA Kabudula. | | | | | | | | | | | | | |  |
|  | | |  | | | | | | | | | | | | | |  |
|  | | | **Codes\\Personal history\Marital status** | | | | | | | | | | | | | |  |
|  |  |  |  |  | No |  | 0.0029 |  | 1 |  | | | | | | |  |
|  | | |  |  |  |  |  |  |  |  | | | | | | | |
|  | | | | | | | | | | | | 1 |  | MTK |  | 6/30/2021 11:04 PM |  |
|  | | | I: Are you married?  R: Yes I am married with three children; two girls and one boy. | | | | | | | | | | | | | |  |
|  | | |  | | | | | | | | | | | | | |  |
|  | | | | | | | | | | | | | | | | | |
| Formatted Reports\\Coding Summary by File Formatted Report | | | | | | | | | | | Page 39 of 53 | | | | | | |
| 7/5/2021 9:43 AM | | | | | | | | | | | | | | | | | |
|  | | | **Classification** |  | **Aggregate** |  | **Coverage** |  | **Number Of Coding References** |  | | **Reference Number** |  | **Coded By Initials** |  | **Modified On** |  |
|  | | | **Codes\\Personal history\Number of children** | | | | | | | | | | | | | |  |
|  |  |  |  |  | No |  | 0.0072 |  | 1 |  | | | | | | |  |
|  | | |  |  |  |  |  |  |  |  | | | | | | | |
|  | | | | | | | | | | | | 1 |  | MTK |  | 6/30/2021 11:04 PM |  |
|  | | | R: Yes I am married with three children; two girls and one boy.  I: Thank you. Are these the only pregnancies you have had?  R: I have been pregnant four times, one was still birth. I have three live children. | | | | | | | | | | | | | |  |
|  | | |  | | | | | | | | | | | | | |  |
|  | | | **Codes\\Previous work experience\Volunteer experience** | | | | | | | | | | | | | |  |
|  |  |  |  |  | No |  | 0.0602 |  | 2 |  | | | | | | |  |
|  | | |  |  |  |  |  |  |  |  | | | | | | | |
|  | | | | | | | | | | | | 1 |  | MTK |  | 6/30/2021 11:07 PM |  |
|  | | | I: Thanks. You memorized it… [Laughter]… So that was our first part. For the second part, I am going to ask you some questions as earlier said. So apart from your taking part in this thinking health program for pregnant women and lactating mothers, have you been a volunteer in health related fields before?  R: Very much so. I have been a volunteer since 2006 whereby I was bringing family planning methods to the communities as a CBDA. The aim is to help women live healthy lives. So with this thinking healthy program that involved the pregnant women, it is like an add on to the activities that I was already doing. | | | | | | | | | | | | | |  |
|  | | |  | | | | | | | | | | | | | |  |
|  | | | | | | | | | | | | 2 |  | MTK |  | 6/30/2021 11:08 PM |  |
|  | | | I: We will discuss more about that. Before that, what have been your experiences with mobile family planning services be?  R: There were challenges that we were experiencing during the time when we were providing these services because it was not easy for women to understand the family planning concept. We had a task to convince the women that if they are on family planning methods, they live happy lives and have a happy family.  I: What made it difficult for the women not to understand family planning since 2006 when you started providing the services?  R: The challenge is that people in the communities are resistant to change. They cling to the old beliefs that if their women are on family planning methods, they would not have been born and so they would wish to give birth to as many children as possible so that every child who was supposed to be born out of them should see the world. The second misconception is that people in the communities think that family planning methods such as the oral pills cause cancer. So they did not take it positively but they thought we were bringing strange beliefs to them. | | | | | | | | | | | | | |  |
|  | | |  |  |  |  |  |  |  |  |  |  |  |  |  |  |  |
|  | | |  | | | | | | | | | | | | | |  |
|  | | | **Codes\\Previous work experience\Volunteer experience\Activities** | | | | | | | | | | | | | |  |
|  |  |  |  |  | No |  | 0.0214 |  | 1 |  | | | | | | |  |
|  | | |  |  |  |  |  |  |  |  | | | | | | | |
|  | | | | | | | | | | | | 1 |  | MTK |  | 6/30/2021 11:07 PM |  |
|  | | | I: Thanks. You memorized it… [Laughter]… So that was our first part. For the second part, I am going to ask you some questions as earlier said. So apart from your taking part in this thinking health program for pregnant women and lactating mothers, have you been a volunteers in health related fields before?  R: Very much so. I have been a volunteer since 2006 whereby I was bringing family planning methods to the communities as a CBDA. The aim is to help women live healthy lives. So with this thinking healthy program that involved the pregnant women, it is like an add on to the activities that I was already doing. | | | | | | | | | | | | | |  |
|  | | |  | | | | | | | | | | | | | |  |
|  | | | **Codes\\Previous work experience\Volunteer experience\Length of volunteer experience** | | | | | | | | | | | | | |  |
|  |  |  |  |  | No |  | 0.0214 |  | 1 |  | | | | | | |  |
|  | | |  |  |  |  |  |  |  |  | | | | | | | |
|  | | | | | | | | | | | | 1 |  | MTK |  | 6/30/2021 11:09 PM |  |
|  | | | I: Thanks. You memorized it… [Laughter]… So that was our first part. For the second part, I am going to ask you some questions as earlier said. So apart from your taking part in this thinking health program for pregnant women and lactating mothers, have you been a volunteers in health related fields before?  R: Very much so. I have been a volunteer since 2006 whereby I was bringing family planning methods to the communities as a CBDA. The aim is to help women live healthy lives. So with this thinking healthy program that involved the pregnant women, it is like an add on to the activities that I was already doing. | | | | | | | | | | | | | |  |
|  | | |  | | | | | | | | | | | | | |  |
| Formatted Reports\\Coding Summary by File Formatted Report | | | | | | | | | | | Page 40 of 53 | | | | | | |
| 7/5/2021 9:43 AM | | | | | | | | | | | | | | | | | |
|  | | | **Classification** |  | **Aggregate** |  | **Coverage** |  | **Number Of Coding References** |  | | **Reference Number** |  | **Coded By Initials** |  | **Modified On** |  |
|  | | | **Codes\\Recommendations** | | | | | | | | | | | | | |  |
|  |  |  |  |  | No |  | 0.1059 |  | 3 |  | | | | | | |  |
|  | | |  |  |  |  |  |  |  |  | | | | | | | |
|  | | | | | | | | | | | | 1 |  | MTK |  | 7/2/2021 8:16 AM |  |
|  | | | I: So my last question is that you should review the service that you were providing. Is there any area of improvement in this program and why do you think there should be improvement?  R: Okay. So, as I said the programme was very good. However, the initial plan was that every participant should have ten sessions but it happened that the plan changed after assessing the women, some participants were having eight sessions others ten. So we wished if all the participants had completed all the ten counseling sessions, as in the modules so we could easily follow how the participant is changing. Even us as counselors we should see for ourselfves how the women are improving, not only senior counselors to see the growth of the participants but we should also be able to evaluate our participants and decide on way forward. [ refering to the screening that determined numbers of sessions following child birth].This will help to make this program uniform by not differentiating the services. The other thing is that this program needs to be extended to other people so that when we complete sessions with one participant we should be able to start sessions with new participant. In so doing, this program will benefit every member of the community. It is true that it is a study but there should be an increased number of people to benefit from the service. | | | | | | | | | | | | | |  |
|  | | |  |  |  |  |  |  |  |  |  |  |  |  |  |  |  |
|  | | |  | | | | | | | | | | | | | |  |
|  | | | | | | | | | | | | 2 |  | MTK |  | 7/2/2021 8:12 AM |  |
|  | | | I: What else needs improvement?  R: They need to consider that a counselor is a human being who has a home to manage. We need to have necessary resources. We need to be trained in COVID preventive measure. Also, we working during the rainy season but we did not have any protective garment and sometimes we could come home late because of rainfall. If they could provide us with umbrellas, raincoats or bags to carry our books, we would appreciate because we were coming home late in order to protect the program documents. We can appreciate it this can improve. | | | | | | | | | | | | | |  |
|  | | |  | | | | | | | | | | | | | |  |
|  | | | | | | | | | | | | 3 |  | MTK |  | 7/2/2021 8:12 AM |  |
|  | | | I: We are at the end of our discussion but do you have anything to add?  R: I don’t have much to say but to appreciate this program, and how it has been conducted and that we had the opportunity to take part in this program. I understand that whenever a new thing is starting, there are a lot of lessons that are learnt. We thank those who have brought this programme to continue as it is one way that can help to prevent or reduce deaths of mothers and infants which is a big problem in this community So, if possible, they may consider to increase the numbers of participants to be involved. The other thing is that they should train us to screen women so that we can be able to identify women who are depressed in the community. We don’t want to sit idle after the study, we want to continue helping women. My last word is to thank this program for the vital information that we have got and we have been changed by this intervention, we able to live healthy lives because of what we were teaching the women, although we were not pregnant, we used the information and skills to deal with some anxieties/stress that we have. | | | | | | | | | | | | | |  |
|  | | |  |  |  |  |  |  |  |  |  |  |  |  |  |  |  |
|  | | |  | | | | | | | | | | | | | |  |
|  | | | **Codes\\Supervision\Additional support** | | | | | | | | | | | | | |  |
|  |  |  |  |  | No |  | 0.0234 |  | 1 |  | | | | | | |  |
|  | | |  |  |  |  |  |  |  |  | | | | | | | |
|  | | | | | | | | | | | | 1 |  | MTK |  | 7/1/2021 12:41 PM |  |
|  | | | I: What support would you appreciate?  R: It was not possible to conduct a home visit while dirty. We were supposed to wash and bathe with soap. It would be ideal for them to consider us when they were planning for this program, so that we can also improve our lives, be motivated and happy as we go help the women.  I: So that you would be able to buy soap…  R: Yes, we needed soap to bath and body lotion, plus shoes as well. | | | | | | | | | | | | | |  |
|  | | |  |  |  |  |  |  |  |  |  |  |  |  |  |  |  |
|  | | |  | | | | | | | | | | | | | |  |
|  | | | | | | | | | | | | | | | | | |
|  | | | | | | | | | | | | | | | | | |
| Formatted Reports\\Coding Summary by File Formatted Report | | | | | | | | | | | Page 41 of 53 | | | | | | |
| 7/5/2021 9:43 AM | | | | | | | | | | | | | | | | | |
|  | | | **Classification** |  | **Aggregate** |  | **Coverage** |  | **Number Of Coding References** |  | | **Reference Number** |  | **Coded By Initials** |  | **Modified On** |  |
|  | | | **Codes\\THPP delivery experience** | | | | | | | | | | | | | |  |
|  |  |  |  |  | No |  | 0.1192 |  | 4 |  | | | | | | |  |
|  | | |  |  |  |  |  |  |  |  | | | | | | | |
|  | | | | | | | | | | | | 1 |  | MTK |  | 7/1/2021 12:38 PM |  |
|  | | | I: To continue, I would like to know [Interruption; phone ringing and respondent responding to phone call and audio was paused]. Okay, so I was explaining… I would like to know your general experiences taking part as a volunteer counselor in the thinking healthy program… for example the challenges that you were experiencing when you were providing the counseling to the women, in all areas, were there problems related to the time taken to provide counseling etc… What have been your experiences?  R: Thank you very much. The experience of providing counseling to the women went on well. For the challenges they were there. It was difficult for the women to understand that we were going there for counseling services. They thought we were going there with financial support or medication. Despite the fact that the objectives of the study were made explicit to these women, they were not able to understand that we went to them in the communities just to provide counseling. The major challenge being that this program was implemented during the difficult season of the year, the rainy season where people had no food or soap in the families and to have someone from the health facility to visit them, they had high expectation that they would be given financial support. For us, the time this study was implemented was a challenge as well because this is the time when we are busy working in the garden but instead of going to the garden on that particular day, it was happening that we are going to the participant’s house to conduct the counseling session. Much as we are volunteers who are not expecting to receive anything, we were supposed to be considered with a certain amount of money, which would at least have contributed towards the cost of farming or the activities that we were supposed to do in the gardens. Yes, we understand that we are aiming at improving our communities but we are human beings, we need to be considered. | | | | | | | | | | | | | |  |
|  | | |  |  |  |  |  |  |  |  |  |  |  |  |  |  |  |
|  | | |  |  |  |  |  |  |  |  |  |  |  |  |  |  |  |
|  | | |  | | | | | | | | | | | | | |  |
|  | | | | | | | | | | | | 2 |  | MTK |  | 7/1/2021 1:26 PM |  |
|  | | | I: Much as the women had those thoughts, did you not introduce the program to them before commencement of the counseling intervention?  R: We did explain to them that “we are community volunteers who are bringing counseling to them and not money or soap or medication. So we did understand each other but sometimes because of poverty, people tend to forget what we discussed when we were introducing the program to them. However, although these were the problems but we were receiving warm welcome and the counseling sessions were going on very well and we could observe psychological growth in the participants to the extent that we have become friends with the study participants. | | | | | | | | | | | | | |  |
|  | | |  |  |  |  |  |  |  |  |  |  |  |  |  |  |  |
|  | | |  | | | | | | | | | | | | | |  |
|  | | | | | | | | | | | | 3 |  | MTK |  | 7/1/2021 1:28 PM |  |
|  | | | I: How many women were you following up?  R: I had three women. | | | | | | | | | | | | | |  |
|  | | |  | | | | | | | | | | | | | |  |
|  | | | | | | | | | | | | 4 |  | MTK |  | 7/1/2021 1:31 PM |  |
|  | | | I: I understand you were giving them home work for the next session. I want to understand how this homework was done by the women?  R: These women were able to do their homework although at times they had some barriers to the implementation of their homework. For example if we gave them homework on nutrition, they could not manage everything because of lack of resources. We have done this study yes, but it is during the bad time of the year when most of the households have no food. It might seem as if the woman is not able to implement what was agree but it was the situation she was in. they were trying in some areas where they could afford and we knew that they were able to do what they were capable of in relation to maintaining their health in their relation to the child they are expecting. | | | | | | | | | | | | | |  |
|  | | |  |  |  |  |  |  |  |  |  |  |  |  |  |  |  |
|  | | |  | | | | | | | | | | | | | |  |
|  | | | **Codes\\THPP delivery experience\Elements\Barriers** | | | | | | | | | | | | | |  |
|  |  |  |  |  | No |  | 0.0980 |  | 3 |  | | | | | | |  |
|  | | |  |  |  |  |  |  |  |  | | | | | | | |
|  | | | | | | | | | | | | 1 |  | MTK |  | 7/1/2021 1:26 PM |  |
|  | | | I: Much as the women had those thoughts, did you not introduce the program to them before commencement of the counseling intervention?  R: We did explain to them that “we are community volunteers who are bringing counseling to them and not money or soap or medication. So we did understand each other but sometimes because of poverty, people tend to forget what we discussed when we were introducing the program to them. However, although these were the problems but we were receiving warm welcome and the counseling sessions were going on very well and we could observe psychological growth in the participants to the extent that we have become friends with the study participants. | | | | | | | | | | | | | |  |
|  | | |  |  |  |  |  |  |  |  |  |  |  |  |  |  |  |
|  | | |  | | | | | | | | | | | | | |  |
| Formatted Reports\\Coding Summary by File Formatted Report | | | | | | | | | | | Page 42 of 53 | | | | | | |
| 7/5/2021 9:43 AM | | | | | | | | | | | | | | | | | |
|  | | | **Classification** |  | **Aggregate** |  | **Coverage** |  | **Number Of Coding References** |  | | **Reference Number** |  | **Coded By Initials** |  | **Modified On** |  |
|  | | | | | | | | | | | | | | | | | |
|  | | | | | | | | | | | | 2 |  | MTK |  | 7/2/2021 8:18 AM |  |
|  | | | I: You have said that there were tools that you were using when providing the counseling sessions… You mentioned about the transport reimbursement… Can you mention some of the barriers for you to provide the counseling sessions?  R: It is true that transport could be a barrier because we were meeting every two weeks for supervision and this means that we were reimbursed transport for the visits that were already conducted. So we had to be committed to conduct the counseling session because it meant that we had to find transport on our own to go and conduct the session. For the tools, as I said, the woman was not given a comprehensive package because we were supposed to give her the book so that she could work on it for the whole week and by the time we go for the visit, she should have answers. Because we were not leaving the book with her, when we came for the next visit, it was as if the woman was doing the assignment on the same day much as it was in the form of homework because she had nowhere to refer to. | | | | | | | | | | | | | |  |
|  | | |  |  |  |  |  |  |  |  |  |  |  |  |  |  |  |
|  | | |  | | | | | | | | | | | | | |  |
|  | | | | | | | | | | | |  |  |  |  |  |  |
|  | | |  |  |  |  |  |  |  |  | | | | | | | |
|  | | | | | | | | | | | |  |  |  |  |  |  |
|  | | |  | | | | | | | | | | | | | |  |
|  | | |  |  |  |  |  |  |  |  |  |  |  |  |  |  |  |
|  | | |  | | | | | | | | | | | | | |  |
|  | | | **Codes\\THPP delivery experience\Elements\Difficult to deliver** | | | | | | | | | | | | | |  |
|  |  |  |  |  | No |  | 0.0278 |  | 1 |  | | | | | | |  |
|  | | |  |  |  |  |  |  |  |  | | | | | | | |
|  | | | | | | | | | | | | 1 |  | MTK |  | 7/1/2021 1:29 PM |  |
|  | | | I: My last question on this section is about the resources to provide the counseling intervention. Did you have enough resources to provide the counseling session or did you have some challenges?  R: We had adequate resources although we did not have enough books whereby the participant was supposed to tick on pictures after doing her homework and we had to leave the book with her but because they were not enough, we were not leaving the book with the participant. However, we had a new skill that we were taught whereby we were documenting somewhere in the book with a pencil the information that the participant gave us and when we went to another participant, we were able to delete the later information and write information for the next participant. So we successfully did the required activity. | | | | | | | | | | | | | |  |
|  | | |  |  |  |  |  |  |  |  |  |  |  |  |  |  |  |
|  | | |  | | | | | | | | | | | | | |  |
|  | | | | | | | | | | | | | | | | | |
| Formatted Reports\\Coding Summary by File Formatted Report | | | | | | | | | | | Page 43 of 53 | | | | | | |
| 7/5/2021 9:43 AM | | | | | | | | | | | | | | | | | |
|  | | | **Classification** |  | **Aggregate** |  | **Coverage** |  | **Number Of Coding References** |  | | **Reference Number** |  | **Coded By Initials** |  | **Modified On** |  |
|  | | | **Codes\\THPP delivery experience\Elements\Easy to deliver** | | | | | | | | | | | | | |  |
|  |  |  |  |  | No |  | 0.0712 |  | 2 |  | | | | | | |  |
|  | | |  |  |  |  |  |  |  |  | | | | | | | |
|  | | | | | | | | | | | | 1 |  | MTK |  | 7/2/2021 8:23 AM |  |
|  | | | I: We are going to the last section as I said that we have three sections. Having looked at the woman, I want you now to look at yourself as the counselor. Were there things that you feel were easy to do when you were providing the counseling session?  R: It was easy to implement because we were providing the service to the woman who live within our community, we were helping women within our community, we know where these people live and we were able to make follow up visits to this woman, it was easy to go to and from the women’s homes. The other thing is that it was easy to conduct the session because we were using the same content that we learnt during the training. There were no additions, so it was easy. | | | | | | | | | | | | | |  |
|  | | |  | | | | | | | | | | | | | |  |
|  | | | | | | | | | | | | 2 |  | MTK |  | 7/2/2021 8:18 AM |  |
|  | | | I: As we are going towards the end, in your perception, which areas do you think have gone very well and which areas have not gone well?  R: The areas that have gone well are that the counseling sessions were successfully done and most of the women have recovered from perinatal depression because for example in my case, I had three women whereby each one of them has low EPDS as compared to her scores during screening. This shows that counseling has really helped them and they all appreciate the service that it has improved their lives. It would be better to extend this service to a larger group of women because the study participants are not the only depressed women in the community. It was happening that when we went to the community, the women were inviting other women in the community to join the session and at the end of the session we could realize that the invited pregnant woman is more depressed than the study participant but we did not know what we could do. In terms of transport, they should set aside a fixed amount of money for the counselors for the activity so that they can be able to plan whether to use it for transport or other things.  I: So you mean that the number of women who were visited is small as compared to those who are sick?  R: Yes we observed that they were seriously sick than our participants. | | | | | | | | | | | | | |  |
|  | | |  |  |  |  |  |  |  |  |  |  |  |  |  |  |  |
|  | | |  | | | | | | | | | | | | | |  |
|  | | | **Codes\\THPP delivery experience\Elements\Facilitators** | | | | | | | | | | | | | |  |
|  |  |  |  |  | No |  | 0.0948 |  | 3 |  | | | | | | |  |
|  | | |  |  |  |  |  |  |  |  | | | | | | | |
|  | | | | | | | | | | | | 1 |  | MTK |  | 7/1/2021 12:53 PM |  |
|  | | | I: That is well explained. Now as you were going to the house of the women for the intervention. What was it that was happening, were you meeting any problems?  R: Thanks. As I said, most of the families were expecting that we were going there with financial or material support besides the counseling services. There was a time when we were giving them soap and the women were very excited. All in all, people in the communities were excited with our service. Much as we did not have the material support but the communities received it [the intervention] with high morale and people loved us in the communities. The women we were counseling have delivered but they are so motivated and they are saying that they are well equipped with counseling skills that they can even help other women in the communities because of the counseling sessions that we were having with them. | | | | | | | | | | | | | |  |
|  | | |  |  |  |  |  |  |  |  |  |  |  |  |  |  |  |
|  | | |  | | | | | | | | | | | | | |  |
|  | | | | | | | | | | | | 2 |  | MTK |  | 7/1/2021 12:56 PM |  |
|  | | | I: What do you mean by requirements?  R: As I said, people in the communities, the mother and her family did not expect us just to go there with counseling and come back. They expected material support, as with the soap that we gave them they were even expecting to get some financial support each time we were visiting. At times they could think we were given the financial support but we were not extending it to them. However, as people from the same communities, we tried explaining and assisting them and they eventually were able to understand us and understand the objective of the program and we were able to help them replace unhelpful thoughts with helpful ones. | | | | | | | | | | | | | |  |
|  | | |  | | | | | | | | | | | | | |  |
|  | | | | | | | | | | | | 3 |  | MTK |  | 7/1/2021 1:30 PM |  |
|  | | | I: Now we want to look at the woman whom you were following up… What was the willingness of the women considering that the counseling was specifically for them? Were they willing? You have told me that sometimes it was happening that the woman went to fetch water during the time of her scheduled visit and you were not able to find her at home, how was the adherence to the scheduled visits? What was happening? Was it because you did not keep the time to arrive at her home? How was this happening?  R: A woman is supposed to do various household chores during the day. It was happening that the woman has just moved few steps but she is within the community keeping in mind her scheduled home visit. She was not going to take long time drawing water. She would come back within a short period of time. It never happened that a woman missed her scheduled home visit because she went away no. unless there was a funeral in the community where we had no control but otherwise the women were adherent to their visits. We were conducting the visits on the agreed time and date. Her going to draw water was also part of the implementation of living healthy program because that was part of exercise. | | | | | | | | | | | | | |  |
|  | | |  |  |  |  |  |  |  |  |  |  |  |  |  |  |  |
|  | | |  | | | | | | | | | | | | | |  |
| Formatted Reports\\Coding Summary by File Formatted Report | | | | | | | | | | | Page 44 of 53 | | | | | | |
| 7/5/2021 9:43 AM | | | | | | | | | | | | | | | | | |
|  | | | **Classification** |  | **Aggregate** |  | **Coverage** |  | **Number Of Coding References** |  | | **Reference Number** |  | **Coded By Initials** |  | **Modified On** |  |
|  | | | **Codes\\THPP training** | | | | | | | | | | | | | |  |
|  |  |  |  |  | No |  | 0.0115 |  | 1 |  | | | | | | |  |
|  | | |  |  |  |  |  |  |  |  | | | | | | | |
|  | | | | | | | | | | | | 1 |  | MTK |  | 7/1/2021 12:35 PM |  |
|  | | | I: You have mentioned about time… how many days were you trained?  R: We were trained for five days. However, within this period, we were not able to cover some of the topics. | | | | | | | | | | | | | |  |
|  | | |  | | | | | | | | | | | | | |  |
|  | | | **Codes\\THPP training\Content** | | | | | | | | | | | | | |  |
|  |  |  |  |  | No |  | 0.0267 |  | 2 |  | | | | | | |  |
|  | | |  |  |  |  |  |  |  |  | | | | | | | |
|  | | | | | | | | | | | | 1 |  | MTK |  | 7/1/2021 12:36 PM |  |
|  | | | I: How long would you like the training to be done?  R: It should have for one week (7 days) so that we could be able to have a face to face interaction and then we could do revision on all the topics and then go for field work. In so doing, the volunteer counselors would be well equipped with the training content. | | | | | | | | | | | | | |  |
|  | | |  | | | | | | | | | | | | | |  |
|  | | | | | | | | | | | | 2 |  | MTK |  | 7/2/2021 8:19 AM |  |
|  | | | I: So you have explained very well that distance to the women was not an issue. Was it easy to use the knowledge you got from the training sessions to conduct the counseling session with the women?  R: As I said earlier on, the time for the training was short and we seemed to forget some of the content. In short, we were not supposed to commute from home during the training sessions. We were supposed to go somewhere else where we would have maximum concentration. | | | | | | | | | | | | | |  |
|  | | |  | | | | | | | | | | | | | |  |
|  | | | **Codes\\THPP training\Duration** | | | | | | | | | | | | | |  |
|  |  |  |  |  | No |  | 0.0267 |  | 2 |  | | | | | | |  |
|  | | |  |  |  |  |  |  |  |  | | | | | | | |
|  | | | | | | | | | | | | 1 |  | MTK |  | 7/1/2021 12:36 PM |  |
|  | | | I: How long would you like the training to be done?  R: It should have for one week (7 days) so that we could be able to have a face to face interaction and then we could do revision on all the topics and then go for field work. In so doing, the volunteer counselors would be well equipped with the training content. | | | | | | | | | | | | | |  |
|  | | |  | | | | | | | | | | | | | |  |
|  | | | | | | | | | | | | 2 |  | MTK |  | 7/2/2021 8:19 AM |  |
|  | | | I: So you have explained very well that distance to the women was not an issue. Was it easy to use the knowledge you got from the training sessions to conduct the counseling session with the women?  R: As I said earlier on, the time for the training was short and we seemed to forget some of the content. In short, we were not supposed to commute from home during the training sessions. We were supposed to go somewhere else where we would have maximum concentration. | | | | | | | | | | | | | |  |
|  | | |  | | | | | | | | | | | | | |  |
|  | | | **Codes\\THPP training\Preparedness** | | | | | | | | | | | | | |  |
|  |  |  |  |  | No |  | 0.0183 |  | 1 |  | | | | | | |  |
|  | | |  |  |  |  |  |  |  |  | | | | | | | |
|  | | | | | | | | | | | | 1 |  | MTK |  | 7/2/2021 8:17 AM |  |
|  | | | I: Okay. Now I want to ask you your personal question. You were trained to provide the counseling and when you were going there in the community to meet the women, how did you perceive your capability to provide the counseling session?  R: I was well equipped and I feel I was very capable to provide the counseling session and I am very committed to this work considering that we are developing our area of TA Kabudula. After the training, I was eager to go and provide the counseling session knowing that we will improve our area. | | | | | | | | | | | | | |  |
|  | | |  | | | | | | | | | | | | | |  |
|  | | | | | | | | | | | | | | | | | |
| Formatted Reports\\Coding Summary by File Formatted Report | | | | | | | | | | | Page 45 of 53 | | | | | | |
| 7/5/2021 9:43 AM | | | | | | | | | | | | | | | | | |
|  | | | **Classification** |  | **Aggregate** |  | **Coverage** |  | **Number Of Coding References** |  | | **Reference Number** |  | **Coded By Initials** |  | **Modified On** |  |
|  | **Files\\THPP PV_#3** | | | | | | | | | | | | | | | |  |
|  | | **Code** | | | | | | | | | | | | | | |  |
|  | | | **Codes\\Health workers** | | | | | | | | | | | | | |  |
|  |  |  |  |  | No |  | 0.0183 |  | 1 |  | | | | | | |  |
|  | | |  |  |  |  |  |  |  |  | | | | | | | |
|  | | | | | | | | | | | | 1 |  | MTK |  | 7/2/2021 8:59 AM |  |
|  | | | I: So you were like a liaison person for the health workers and the study participants. How have you worked with the health workers who were also providing service to the women that you were counseling.  R: I can say that the health workers provided medication while we were providing counseling intervention. I can say, our roles were different, but….. | | | | | | | | | | | | | |  |
|  | | |  | | | | | | | | | | | | | |  |
|  | | | **Codes\\Health workers\Relationship** | | | | | | | | | | | | | |  |
|  |  |  |  |  | No |  | 0.0032 |  | 1 |  | | | | | | |  |
|  | | |  |  |  |  |  |  |  |  | | | | | | | |
|  | | | | | | | | | | | | 1 |  | MTK |  | 7/2/2021 8:59 AM |  |
|  | | | I: There were no challenges…  R: No. our relationship was good | | | | | | | | | | | | | |  |
|  | | |  | | | | | | | | | | | | | |  |
|  | | | **Codes\\Involvement of family members** | | | | | | | | | | | | | |  |
|  |  |  |  |  | No |  | 0.0438 |  | 1 |  | | | | | | |  |
|  | | |  |  |  |  |  |  |  |  | | | | | | | |
|  | | | | | | | | | | | | 1 |  | MTK |  | 7/2/2021 8:46 AM |  |
|  | | | I: Were you able to meet relatives of the woman when you visited their homes? It could happen that you met a mother, mother in-law, children, other family members… how were your interactions with the family of the woman and what role were they playing in the intervention?  R: All the women I had sessions with, their partners were present during the first four visits before they delivered. After delivery, they were with other relatives. For example, the relatives and mother in-law. For example, I was encouraging the mother in-law to participate in the discussion and they were really participating in the intervention.  I: That is a very good point. You have said that you conducted the first, second, third and fourth sessions with the two women you had sessions with…  R: Yes.  I: Were partners available in all these sessions?  R: Yes. | | | | | | | | | | | | | |  |
|  | | |  |  |  |  |  |  |  |  |  |  |  |  |  |  |  |
|  | | |  | | | | | | | | | | | | | |  |
|  | | | **Codes\\Personal history** | | | | | | | | | | | | | |  |
|  |  |  |  |  | No |  | 0.0112 |  | 1 |  | | | | | | |  |
|  | | |  |  |  |  |  |  |  |  | | | | | | | |
|  | | | | | | | | | | | | 1 |  | MTK |  | 7/2/2021 8:30 AM |  |
|  | | | : What type of marriage are you in?  R: I am the only wife to my partner.  I: Is he your first husband and are you the first wife?  R: He is my first husband and I am his first wife. We got married as a boy and girl. | | | | | | | | | | | | | |  |
|  | | |  | | | | | | | | | | | | | |  |
|  | | | | | | | | | | | | | | | | | |
| Formatted Reports\\Coding Summary by File Formatted Report | | | | | | | | | | | Page 46 of 53 | | | | | | |
| 7/5/2021 9:43 AM | | | | | | | | | | | | | | | | | |
|  | | | **Classification** |  | **Aggregate** |  | **Coverage** |  | **Number Of Coding References** |  | | **Reference Number** |  | **Coded By Initials** |  | **Modified On** |  |
|  | | | **Codes\\Personal history\Age** | | | | | | | | | | | | | |  |
|  |  |  |  |  | No |  | 0.0153 |  | 1 |  | | | | | | |  |
|  | | |  |  |  |  |  |  |  |  | | | | | | | |
|  | | | | | | | | | | | | 1 |  | MTK |  | 7/2/2021 8:28 AM |  |
|  | | | I: So, our discussion will cover three sections. The first part is about your demographic information, the remaining two parts are about the study in general in which you have been taking part in. So to start with, how old are you?  R: I was born on 25 February in 1988 and I am 34 years old. | | | | | | | | | | | | | |  |
|  | | |  | | | | | | | | | | | | | |  |
|  | | | **Codes\\Personal history\Contact information** | | | | | | | | | | | | | |  |
|  |  |  |  |  | No |  | 0.0141 |  | 1 |  | | | | | | |  |
|  | | |  |  |  |  |  |  |  |  | | | | | | | |
|  | | | | | | | | | | | | 1 |  | MTK |  | 7/2/2021 8:32 AM |  |
|  | | | I: Nowadays people are mostly using phones. Do you have one?  R: Yes.  I: Do you need to check of the phone number or you memorized it?  R: I have to check for it.  I: Okay where did you write it?  R: I saved it somewhere…  I: Can you tell me the number?  R: ………. | | | | | | | | | | | | | |  |
|  | | |  |  |  |  |  |  |  |  |  |  |  |  |  |  |  |
|  | | |  | | | | | | | | | | | | | |  |
|  | | | **Codes\\Personal history\Level of education** | | | | | | | | | | | | | |  |
|  |  |  |  |  | No |  | 0.0254 |  | 1 |  | | | | | | |  |
|  | | |  |  |  |  |  |  |  |  | | | | | | | |
|  | | | | | | | | | | | | 1 |  | MTK |  | 7/2/2021 8:29 AM |  |
|  | | | I: How far did you go with your education?  R: I went up to form 3.  I: What happened for you stop at form 3?  R: It was not because of lack of school fees but I was sick and when I came back home, my friends were far with the syllabus and I thought I could not catch up. When I came here they tried to send me to Kabudula Community Secondary School and I was sick again. I had sores in my eyes and had two operations. After the two operations it happened that I could not be able to read. | | | | | | | | | | | | | |  |
|  | | |  | | | | | | | | | | | | | |  |
|  | | | **Codes\\Personal history\Location** | | | | | | | | | | | | | |  |
|  |  |  |  |  | No |  | 0.0073 |  | 1 |  | | | | | | |  |
|  | | |  |  |  |  |  |  |  |  | | | | | | | |
|  | | | | | | | | | | | | 1 |  | MTK |  | 7/2/2021 8:31 AM |  |
|  | | | I: Where are you currently staying?  R: I am staying in ………………...  I: Is …………………….. a village or a trading centre?  R: It is a village. | | | | | | | | | | | | | |  |
|  | | |  | | | | | | | | | | | | | |  |
|  | | | | | | | | | | | | | | | | | |
|  | | | | | | | | | | | | | | | | | |
| Formatted Reports\\Coding Summary by File Formatted Report | | | | | | | | | | | Page 47 of 53 | | | | | | |
| 7/5/2021 9:43 AM | | | | | | | | | | | | | | | | | |
|  | | | **Classification** |  | **Aggregate** |  | **Coverage** |  | **Number Of Coding References** |  | | **Reference Number** |  | **Coded By Initials** |  | **Modified On** |  |
|  | | | **Codes\\Personal history\Marital status** | | | | | | | | | | | | | |  |
|  |  |  |  |  | No |  | 0.0264 |  | 1 |  | | | | | | |  |
|  | | |  |  |  |  |  |  |  |  | | | | | | | |
|  | | | | | | | | | | | | 1 |  | MTK |  | 7/2/2021 8:29 AM |  |
|  | | | I: Sorry for that. Are you married?  R: I am married with two children; one boy and one girl.  R: Yes but I have not stopped bearing…  I: Meaning that given an opportunity you can have another child?  R: Yes, but not soon.  I: What are you discussing with your partner on child bearing?  R: We are discussing that it is good to have children but we agreed that we need to have a number of children that we can be able to take care of. We should be able to send the children to school. | | | | | | | | | | | | | |  |
|  | | |  |  |  |  |  |  |  |  |  |  |  |  |  |  |  |
|  | | |  | | | | | | | | | | | | | |  |
|  | | | **Codes\\Previous work experience\Volunteer experience** | | | | | | | | | | | | | |  |
|  |  |  |  |  | No |  | 0.0162 |  | 1 |  | | | | | | |  |
|  | | |  |  |  |  |  |  |  |  | | | | | | | |
|  | | | | | | | | | | | | 1 |  | MTK |  | 7/2/2021 8:33 AM |  |
|  | | | : So that was our first part. For the second part, I am going to ask the next set of questions. So apart from your part in this thinking healthy program for pregnant women and lactating mothers, have you been a volunteers in health related fields before?  R: I worked as a community volunteer in area of health. | | | | | | | | | | | | | |  |
|  | | |  | | | | | | | | | | | | | |  |
|  | | | **Codes\\Previous work experience\Volunteer experience\Activities** | | | | | | | | | | | | | |  |
|  |  |  |  |  | No |  | 0.0410 |  | 2 |  | | | | | | |  |
|  | | |  |  |  |  |  |  |  |  | | | | | | | |
|  | | | | | | | | | | | | 1 |  | MTK |  | 7/2/2021 8:34 AM |  |
|  | | | I: What was involved?  R: We were looking at water and sanitation in the communities. | | | | | | | | | | | | | |  |
|  | | |  | | | | | | | | | | | | | |  |
|  | | | | | | | | | | | | 2 |  | MTK |  | 7/2/2021 8:34 AM |  |
|  | | | I: Can you describe your roles in the water and sanitation program?  R: We were helping people to know that we are supposed to drink water from boreholes and not wells. On sanitation, we were educating them on the importance of having pit latrines, rubbish pits, lines for drying clothes and a bathroom. We ensured everyone in the community has these things.  I: Were people able to do what you were teaching them?  R: Because some people are different and some were able to understand but others were not able to understand. However, most of the households in my community were able to implement the activities and it happened that our VDC became the first in successful implementation of the program. | | | | | | | | | | | | | |  |
|  | | |  |  |  |  |  |  |  |  |  |  |  |  |  |  |  |
|  | | |  | | | | | | | | | | | | | |  |
|  | | | **Codes\\Recommendations** | | | | | | | | | | | | | |  |
|  |  |  |  |  | No |  | 0.1286 |  | 5 |  | | | | | | |  |
|  | | |  |  |  |  |  |  |  |  | | | | | | | |
|  | | | | | | | | | | | | 1 |  | MTK |  | 7/2/2021 9:04 AM |  |
|  | | | I: You have said that the introduction session was difficult. What do you think would have happened in order to make this session easy?  R: What was supposed to happen was that we were supposed to be prepared psychologically for the session so that we should not be stressed… | | | | | | | | | | | | | |  |
|  | | |  | | | | | | | | | | | | | |  |
|  | | | | | | | | | | | | | | | | | |
| Formatted Reports\\Coding Summary by File Formatted Report | | | | | | | | | | | Page 48 of 53 | | | | | | |
| 7/5/2021 9:43 AM | | | | | | | | | | | | | | | | | |
|  | | | **Classification** |  | **Aggregate** |  | **Coverage** |  | **Number Of Coding References** |  | | **Reference Number** |  | **Coded By Initials** |  | **Modified On** |  |
|  | | | | | | | | | | | | | | | | | |
|  | | | | | | | | | | | | 2 |  | MTK |  | 7/2/2021 9:04 AM |  |
|  | | | I: So we are moving towards the end of the interview. We understand that for you to be able to deliver the counseling intervention in this program, you were trained and given all the resources before you went to the woman to start delivering the intervention. What do you think should be done to improve the program?  R: The training for this study was supposed to be done at a place where we had to be accommodated right there because what was happening was that we were coming from different locations and the training content was difficult. So because we had to switch from training activities to household chores, most of the days, we could hardly remember what we learnt the previous day during recap and this concern was raised. | | | | | | | | | | | | | |  |
|  | | |  |  |  |  |  |  |  |  |  |  |  |  |  |  |  |
|  | | |  | | | | | | | | | | | | | |  |
|  | | | | | | | | | | | | 3 |  | MTK |  | 7/2/2021 9:04 AM |  |
|  | | | I: So you did not like the commuting?  R: No. If we can all be there, we could be discussing after the training to remind each other what we learnt and no wonder our colleague [Name of volunteer] was not able to perform successfully during the training because she was forgetting everything that was learnt during the training and it was difficult for her to deliver the intervention. | | | | | | | | | | | | | |  |
|  | | |  | | | | | | | | | | | | | |  |
|  | | | | | | | | | | | | 4 |  | MTK |  | 7/2/2021 9:05 AM |  |
|  | | | I: What else should be improved apart from that?  R: The other thing to be improved is to give us transport prior to the visit and not giving us after we have visited the mother already.  I: How should this be done?  R: What did you say?  I: What should we do to improve the issue of transport?  R: There are volunteers in the communities and they have their mode of transportation. This makes it easy for them to conduct the home visits. For example, [Name of volunteer] is able to conduct his home based visits because he was given transport to use. As for us, we did not have arranged transport and the money was reimbursed after the visit had already been conducted. This made life hard for us because we had to hire a motor cyclist to take us to and from the participant on credit so that when the researcher gives us we should give him. Some motor cyclists were accepting it but others were refusing. | | | | | | | | | | | | | |  |
|  | | |  |  |  |  |  |  |  |  |  |  |  |  |  |  |  |
|  | | |  | | | | | | | | | | | | | |  |
|  | | | | | | | | | | | | 5 |  | MTK |  | 7/2/2021 9:06 AM |  |
|  | | | I: Perhaps would you like to be given transport prior to the home visit so that you do not go there on credit?  R: Yes because no many people accept to be hired on credit. | | | | | | | | | | | | | |  |
|  | | |  | | | | | | | | | | | | | |  |
|  | | | **Codes\\THPP delivery experience** | | | | | | | | | | | | | |  |
|  |  |  |  |  | No |  | 0.0462 |  | 2 |  | | | | | | |  |
|  | | |  |  |  |  |  |  |  |  | | | | | | | |
|  | | | | | | | | | | | | 1 |  | MTK |  | 7/2/2021 8:41 AM |  |
|  | | | I: Like how long was it taking?  R: More than one hour.  I: Like how many minutes?  R: One and half hours. | | | | | | | | | | | | | |  |
|  | | |  | | | | | | | | | | | | | |  |
|  | | | | | | | | | | | | 2 |  | MTK |  | 7/2/2021 8:57 AM |  |
|  | | | I: Was the woman you were visiting showing interest in the counseling that you were providing. What was your experience with the two women?  R: Each woman was interested because they were attentive when I was speaking and the interaction was participatory. This showed that they were committed to the counseling sessions and they were looking into my face when interacting. They were really interested in sessions  I: Thank you very much. How was the compliance of the women to the sessions. Were you able to find them during the scheduled visits?  R: We were agreeing on the next scheduled dates and they were available. Sometimes before I start off, she could call me or the motor cyclist could find me at the agreed time so that we can go. They were always ready for the session. | | | | | | | | | | | | | |  |
|  | | |  |  |  |  |  |  |  |  |  |  |  |  |  |  |  |
|  | | |  | | | | | | | | | | | | | |  |
|  | | | | | | | | | | | | | | | | | |
| Formatted Reports\\Coding Summary by File Formatted Report | | | | | | | | | | | Page 49 of 53 | | | | | | |
| 7/5/2021 9:43 AM | | | | | | | | | | | | | | | | | |
|  | | | **Classification** |  | **Aggregate** |  | **Coverage** |  | **Number Of Coding References** |  | | **Reference Number** |  | **Coded By Initials** |  | **Modified On** |  |
|  | | | **Codes\\THPP delivery experience\Elements** | | | | | | | | | | | | | |  |
|  |  |  |  |  | No |  | 0.0534 |  | 2 |  | | | | | | |  |
|  | | |  |  |  |  |  |  |  |  | | | | | | | |
|  | | | | | | | | | | | | 1 |  | MTK |  | 7/2/2021 9:00 AM |  |
|  | | | I: We are moving to the last part of the interview this afternoon. I would like to hear from you regarding your experience providing thinking healthy intervention counseling session.  R: What was happening was that sometimes the women were telling us problems that were also affecting us and were beyond my capabilities but because we learnt how to deal with the problems, we could replace unhelpful thoughts with helpful thought, the modules helped us to deal with stress we were also having. So we were able to counsel ourselves so that we are not affected by the issues of the participants. However, I can say that there were no challenges that I experienced during my counseling sessions with the women. | | | | | | | | | | | | | |  |
|  | | |  |  |  |  |  |  |  |  |  |  |  |  |  |  |  |
|  | | |  | | | | | | | | | | | | | |  |
|  | | | | | | | | | | | | 2 |  | MTK |  | 7/2/2021 9:02 AM |  |
|  | | | In addition to that, this study has also helped me and family, it has helped us to be healthy and live a healthy life. I was able to assist my children and husband to replace unhelpful thoughts and behaviors with helpful thoughts and behaviors.  I: Meaning that the study has also improved your family health?  R: Yes. | | | | | | | | | | | | | |  |
|  | | |  | | | | | | | | | | | | | |  |
|  | | | **Codes\\THPP delivery experience\Elements\Barriers** | | | | | | | | | | | | | |  |
|  |  |  |  |  | No |  | 0.0589 |  | 2 |  | | | | | | |  |
|  | | |  |  |  |  |  |  |  |  | | | | | | | |
|  | | | | | | | | | | | | 1 |  | MTK |  | 7/2/2021 8:58 AM |  |
|  | | | I: I understand you were giving them assignment?  R: Yes.  I: How was it going?  R: For some women they were doing their assignment but for some, they were doing it the same day when I come for the next session and they were asking me to mark their scores in the book.  I: So were they completing the home work?  R: In some cases they were not completing as you know that here in the community, it is not possible for the women to eat the six food groups all the time. So they were completing the part where they were able to implement but for the tasks that they did have the resources, they were not doing it. | | | | | | | | | | | | | |  |
|  | | |  |  |  |  |  |  |  |  |  |  |  |  |  |  |  |
|  | | |  | | | | | | | | | | | | | |  |
|  | | | | | | | | | | | | 2 |  | MTK |  | 7/2/2021 9:01 AM |  |
|  | | | I: Continue…  R: Sometimes it was happening that when we go to visit the woman, we could forget some of the things that we learnt in class, that were not in the modules, things we didn’t write down. Our friends, the facilitators were better off because they had laptops with all the information to teach us. So, for us before we went to the participants, we had to read the module to see how we are going to handle the session. So it was okay because, yes we had problems but were able to control the sessions on our own. | | | | | | | | | | | | | |  |
|  | | |  | | | | | | | | | | | | | |  |
|  | | | **Codes\\THPP delivery experience\Elements\Challenges** | | | | | | | | | | | | | |  |
|  |  |  |  |  | No |  | 0.1021 |  | 4 |  | | | | | | |  |
|  | | |  |  |  |  |  |  |  |  | | | | | | | |
|  | | | | | | | | | | | | 1 |  | MTK |  | 7/2/2021 8:37 AM |  |
|  | | | I: What are the other gaps?  R: We were supposed to be given uniforms so that we could easily be identified in the communities as we were going to visit the women in their homes. We were also affected by COVID but we were never given enough masks. The other gap was that we were not given incentives as volunteers. What they were giving us was transport money to and from the homes of the study participants during follow up visits. It was not on because when we were leaving home, partners knew that we were going to do research work and they were expecting that when coming back, we should be able to buy soap and other things but that was not the case. The transport money was equivalent to the money that we were paying to the motorists who were taking us to and from the participants’ homes. | | | | | | | | | | | | | |  |
|  | | |  |  |  |  |  |  |  |  |  |  |  |  |  |  |  |
|  | | |  | | | | | | | | | | | | | |  |
|  | | | | | | | | | | | | 2 |  | MTK |  | 7/2/2021 8:40 AM |  |
|  | | | I: Was the time to conduct the session enough or was it less than that?  R: What happened was that we were trained to conduct a one hour session with the woman but that was not the case on the ground. It was happening that when you start discussing a topic with the participant had a lot to issues/problems to explain and I had to listen to her so that I understand what she was saying. So it was happening that the one hour was not enough. | | | | | | | | | | | | | |  |
|  | | |  | | | | | | | | | | | | | |  |
| Formatted Reports\\Coding Summary by File Formatted Report | | | | | | | | | | | Page 50 of 53 | | | | | | |
| 7/5/2021 9:43 AM | | | | | | | | | | | | | | | | | |
|  | | | **Classification** |  | **Aggregate** |  | **Coverage** |  | **Number Of Coding References** |  | | **Reference Number** |  | **Coded By Initials** |  | **Modified On** |  |
|  | | | | | | | | | | | | | | | | | |
|  | | | | | | | | | | | | 3 |  | MTK |  | 7/2/2021 8:41 AM |  |
|  | | | I: So one hour was not enough…  R: It was not enough. Besides that, they women thought that when we visited them, we went there to give them help in form of food, money especially with the problems people have during the growing season, they expected soap or medicine. So when we went there, they were explaining a lot of issues and for us to follow the counseling procedures, it was taking a long time. | | | | | | | | | | | | | |  |
|  | | |  | | | | | | | | | | | | | |  |
|  | | | | | | | | | | | | 4 |  | MTK |  | 7/2/2021 9:04 AM |  |
|  | | | I: Did you have challenges with the first participant or all the introduction sessions with all the participants were challenging?  R: No, the challenge was with the first participant. For the second and the rest, it was not difficult because I had the experience with the first participant. | | | | | | | | | | | | | |  |
|  | | |  | | | | | | | | | | | | | |  |
|  | | | **Codes\\THPP delivery experience\Elements\Difficult to deliver** | | | | | | | | | | | | | |  |
|  |  |  |  |  | No |  | 0.0984 |  | 4 |  | | | | | | |  |
|  | | |  |  |  |  |  |  |  |  | | | | | | | |
|  | | | | | | | | | | | | 1 |  | MTK |  | 7/2/2021 8:40 AM |  |
|  | | | I: As we continue with our discussion, I would like to know; what has been your experience delivering the counseling intervention sessions to the women?  R: The first session was the introduction and you know that it is difficult most of the times to start a new thing. Before the actual introduction session, I was very worried if I was going to successfully do the intervention as per the training protocol. However, I gathered courage that although I am going to start this new thing, I will be able to deliver. So the introduction session went on well. Then I went home. During the second session, I was not worried because I was used to. | | | | | | | | | | | | | |  |
|  | | |  | | | | | | | | | | | | | |  |
|  | | | | | | | | | | | | 2 |  | MTK |  | 7/2/2021 9:01 AM |  |
|  | | | I: When you say that the participants’ issues were beyond your control… what do you mean? You also said the intervention has also helped you, so I want you to explain more about this?  R: It was happening that the participant was telling us issues that were not in line with our program, problems beyond the programme, for example other disease conditions… We were focusing on thinking healthy program but it was happening that she was telling me other strange health problems that are not related to the study. This meant that we had to think about it and it was consuming a lot of time to address that and move from her topic and go back to the counseling session. | | | | | | | | | | | | | |  |
|  | | |  | | | | | | | | | | | | | |  |
|  | | | | | | | | | | | | 3 |  | MTK |  | 7/2/2021 9:02 AM |  |
|  | | | I: Was there any aspect of the counseling procedure in this study that was easy to deliver? Perhaps were there any barriers to your delivery of the counseling intervention in the thinking healthy program?  R: The difficult session was the introduction session was difficult because it was meeting for the first time for lessons 2 to 8 they were simple because I was used and we were repeating the same format, so they were easy. | | | | | | | | | | | | | |  |
|  | | |  | | | | | | | | | | | | | |  |
|  | | | | | | | | | | | | 4 |  | MTK |  | 7/2/2021 9:02 AM |  |
|  | | | I: So the most difficult one was the introduction session?  R: It was very difficult because I did not know where and how to start but the rest were easy. | | | | | | | | | | | | | |  |
|  | | |  | | | | | | | | | | | | | |  |
|  | | | **Codes\\THPP delivery experience\Elements\Easy to deliver** | | | | | | | | | | | | | |  |
|  |  |  |  |  | No |  | 0.0222 |  | 1 |  | | | | | | |  |
|  | | |  |  |  |  |  |  |  |  | | | | | | | |
|  | | | | | | | | | | | | 1 |  | MTK |  | 7/2/2021 9:02 AM |  |
|  | | | I: Was there any aspect of the counseling procedure in this study that was easy to deliver? Perhaps were there any barriers to your delivery of the counseling intervention in the thinking healthy program?  R: The difficult session was the introduction session was difficult because it was meeting for the first time for lessons 2 to 8 they were simple because I was used and we were repeating the same format, so they were easy. | | | | | | | | | | | | | |  |
|  | | |  | | | | | | | | | | | | | |  |
|  | | | | | | | | | | | | | | | | | |
| Formatted Reports\\Coding Summary by File Formatted Report | | | | | | | | | | | Page 51 of 53 | | | | | | |
| 7/5/2021 9:43 AM | | | | | | | | | | | | | | | | | |
|  | | | **Classification** |  | **Aggregate** |  | **Coverage** |  | **Number Of Coding References** |  | | **Reference Number** |  | **Coded By Initials** |  | **Modified On** |  |
|  | | | **Codes\\THPP delivery experience\Elements\Facilitators** | | | | | | | | | | | | | |  |
|  |  |  |  |  | No |  | 0.0784 |  | 2 |  | | | | | | |  |
|  | | |  |  |  |  |  |  |  |  | | | | | | | |
|  | | | | | | | | | | | | 1 |  | MTK |  | 7/2/2021 8:43 AM |  |
|  | | | I: Okay. So what was your experience at the woman’s home?  R: My first experience is that all the women who I visited warmly welcomed me and no one showed no interest. On the same note, all the participants expected that I was going to give them something, and I used to feel bad when leaving them without anything. There were times when we were given soap to give to the participants and they were very appreciative because of the situations they were in. the soap really assisted them. And they appreciated that the programme helped them | | | | | | | | | | | | | |  |
|  | | |  | | | | | | | | | | | | | |  |
|  | | | | | | | | | | | | 2 |  | MTK |  | 7/2/2021 8:46 AM |  |
|  | | | I: So how come that they were expecting to be given something?  R: I supposed they believed that after being screened at the hospital, and told that volunteers will be visiting them at their homes. They might have believed that if a volunteer from the hospital goes to visit them in their homes, she should bring something for them. However, during the introductory session, we emphasized to them that the counseling sessions are to help them replace unhelpful behaviors with helpful behaviors. Little by little, they started realizing that the counseling was really helping them to have healthy lives but they did not say it that they wanted to be given something but their actions showed that they were expecting something. I am saying this because the times we gave them soap, their appreciation showed that they were really expecting something from us because it is our culture to give something to the person we are visiting.  I: It is cultural to give?  R: Yes. | | | | | | | | | | | | | |  |
|  | | |  |  |  |  |  |  |  |  |  |  |  |  |  |  |  |
|  | | |  | | | | | | | | | | | | | |  |
|  | | | **Codes\\THPP training** | | | | | | | | | | | | | |  |
|  |  |  |  |  | No |  | 0.0758 |  | 3 |  | | | | | | |  |
|  | | |  |  |  |  |  |  |  |  | | | | | | | |
|  | | | | | | | | | | | | 1 |  | MTK |  | 7/2/2021 8:35 AM |  |
|  | | | I: Thank you very much. Now let us look at the study that you have been taking part. Before the study commencement, were you trained?  R: [No response]  I: Before the study commencement, were you trained?  R: No they did not train us.  I: So you were not trained?  R: No, we were trained.  I: Now you can remember?  R: Yes. | | | | | | | | | | | | | |  |
|  | | |  |  |  |  |  |  |  |  |  |  |  |  |  |  |  |
|  | | |  | | | | | | | | | | | | | |  |
|  | | | | | | | | | | | | 2 |  | MTK |  | 7/2/2021 8:38 AM |  |
|  | | | I: What are the other gaps?  R: We were supposed to be given uniforms so that we could easily be identified in the communities as we were going to visit the women in their homes. The other gap was that we were not given incentives as volunteers. What they were giving us was transport money to and from the homes of the study participants during follow up visits. It was not on because when we were leaving home, partners knew that we were going to do research work and they were expecting that when coming back, we should be able to buy soap and other things but that was not the case. The transport money was equivalent to the money that we were paying to the motorists who were taking us to and from the participants’ homes. | | | | | | | | | | | | | |  |
|  | | |  |  |  |  |  |  |  |  |  |  |  |  |  |  |  |
|  | | |  | | | | | | | | | | | | | |  |
|  | | | | | | | | | | | | 3 |  | MTK |  | 7/2/2021 8:50 AM |  |
|  | | | I: That is good news, thank you. In terms of tools that you were using during the counseling sessions.. were they enough?  R: They were not enough. | | | | | | | | | | | | | |  |
|  | | |  | | | | | | | | | | | | | |  |
|  | | | | | | | | | | | | | | | | | |
| Formatted Reports\\Coding Summary by File Formatted Report | | | | | | | | | | | Page 52 of 53 | | | | | | |
| 7/5/2021 9:43 AM | | | | | | | | | | | | | | | | | |
|  | | | **Classification** |  | **Aggregate** |  | **Coverage** |  | **Number Of Coding References** |  | | **Reference Number** |  | **Coded By Initials** |  | **Modified On** |  |
|  | | | **Codes\\THPP training\Content** | | | | | | | | | | | | | |  |
|  |  |  |  |  | No |  | 0.0228 |  | 1 |  | | | | | | |  |
|  | | |  |  |  |  |  |  |  |  | | | | | | | |
|  | | | | | | | | | | | | 1 |  | MTK |  | 7/2/2021 8:36 AM |  |
|  | | | I: Was the training enough to equip you to conduct the study?  R: It was enough and it equipped us with the necessary skills to conduct the study. However, there were some tools such as some books which we were supposed to use during the training, they were not available in time and we accessed the last book towards the end of the training. So I saw that there were some gaps during the training. | | | | | | | | | | | | | |  |
|  | | |  | | | | | | | | | | | | | |  |
|  | | | **Codes\\THPP training\Preparedness** | | | | | | | | | | | | | |  |
|  |  |  |  |  | No |  | 0.0533 |  | 3 |  | | | | | | |  |
|  | | |  |  |  |  |  |  |  |  | | | | | | | |
|  | | | | | | | | | | | | 1 |  | MTK |  | 7/2/2021 8:36 AM |  |
|  | | | I: Was the training enough to equip you to conduct the study?  R: It was enough and it equipped us with the necessary skills to conduct the study. However, there were some tools such as some books which we were supposed to use during the training, they were not available in time and we accessed the last book towards the end of the training. So I saw that there were some gaps during the training. | | | | | | | | | | | | | |  |
|  | | |  | | | | | | | | | | | | | |  |
|  | | | | | | | | | | | | 2 |  | MTK |  | 7/2/2021 8:38 AM |  |
|  | | | I: Thank you. Despite those gaps, did the training help you to successfully deliver the counseling intervention sessions?  R: The training was very helpful and it has helped both the participants and us volunteers to change. Even our families have changed because of that training. | | | | | | | | | | | | | |  |
|  | | |  | | | | | | | | | | | | | |  |
|  | | | | | | | | | | | |  |  |  |  |  |  |
|  | | |  | | | | | | | | | | | | | |  |
|  | | |  | | | | | | | | | | | | | |  |
|  | | | | | | | | | | | | | | | | | |
|  | | | | | | | | | | | | | | | | | |
|  | | | | | | | | | | | | | | | | | |
|  | | | | | | | | | | | | | | | | | |
| Formatted Reports\\Coding Summary by File Formatted Report | | | | | | | | | | | Page 53 of 53 | | | | | | |
